# Supplementary material for: HFE genotypes, haemochromatosis diagnosis and clinical outcomes at age 80 years: a prospective cohort study in the UK Biobank
Source: BMJ Open. 2024 Mar 13;14(3):e081926. doi: 10.1136/bmjopen-2023-081926 (PMC10936495; doi:10.1136/bmjopen-2023-081926)
Supplement: Supplementary data [file bmjopen-2023-081926supp001.pdf]

Supplementary Material

eTable 1. Incident hospital diagnosed outcomes or procedures with associated ICD-10/OPCS codes

Disease	ICD-10 code
Arrhythmia	I49
Cardiomyopathy	I42
CHD	I20; I21; I22; I23; I24; I25
Cholecystitis	K800; K804; K81
COVID-19	U07.1; U07.2
Delirium	F05
Dementia	F00; F01; F02; F03; G30
Alzheimer's disease	G30
Non-Alzheimer's dementia	F00; F01; F02; F03
Depression	F32; F33; F34.1
Fractures (any)	S02; S12; S22; S32; S42; S52; S62; S72; S82; S92; T02; T08; T10; T12; T14.2
Fragility fractures	S220; S32; S325; S328; S422; S423; S424; S524; S525; S720; S721; S722; S582; S5823; T08
Haemochromatosis	E83.1
Heart failure	I50; J81
Liver disease (any)	K70; K71; K72; K73; K74; K75; K76; K77
Alcoholic liver disease	K70
Fibrosis & Cirrhosis	K74
Hepatic failure	K72
Liver cancer	C22
Lower respiratory tract infection	J20; J21; J22
Osteoarthritis	M15.0; M15.1; M15.2; M15.9; M16.0; M16.1; M17.0; M17.1; M18.0; M18.1; M19.0
Osteoporosis	M80; M81; M811; M812; M813; M814; M815; M816; M818; M819
Parkinson's disease	G20; F02.3
Pneumonia	J13; J14; J15; J16; J17; J18
Prostate cancer	C61
Rheumatoid arthritis	M05; M06
Sepsis	A021; A039; A207; A241; A217; A227; A239; A267; A282; A327; A392; A393; A394; A40; A41; A427; A548; B007; B377; H440; J950; N390; O85; P36; R651; T814; T880
Skin soft tissue infection	L00; L01; L02; L03; L04; L05; L06; L07; L08
Type 1 or Type 2 diabetes	E10; E11
Urinary tract infection	N30; N34; N39
Upper respiratory tract infection	J39; J06; J04

Procedure	OPCS Code
Ankle replacement	O32; O320; O321; O322; O323; O324; O325
Hip replacement	W37;W370;W371;W372;W373;W374;W38;W380;W381;W382;W383;W384; W46;W460;W461;W462;W463;W47;W470;W471;W472;W473;W93;W930; W931;W932;W933;W94;W940;W941;W942;W943;O171;O172;O173;W580; W581;W582
Knee replacement	O18*; W40*; W41*; W42*
Shoulder replacement	O06; +A11O060; O061; O062; O063; O068; O069; O07; O070; O071; O072; O073; O078; O079; O08; O080; O081; O082; O083; O084; O088; O089; O09; O091; O098; O099; O10; O101; O108; O109

Lucas MR, *et al. BMJ Open* 2024; 14:e081926. doi: 10.1136/bmjopen-2023-081926

ICD-10 = International Classification of Diseases 10th revision codes; OPCS-4 = OPCS Classification of Interventions and Procedures version 4. Joint replacement surgery variable includes a diagnosis of hip, knee, ankle, or shoulder replacement. Any brain outcome variable includes a diagnosis of dementia, delirium, or Parkinson's disease.

eTable 2. Baseline characteristics of male UK Biobank participants by p.C282Y/H63D genotypes

	No mutations	H63D+/-	H63D+/+	C282Y+/H63D+	C282Y+/-	C282Y +/+	Total
**Total participants**	122,841	47,983	4,673	4,959	24,636	1,298	206,390
Mean age, years (SD)	56.99 (8.1)	57.02 (8.1)	56.99 (8.1)	56.97 (8.1)	57.02 (8.1)	56.84 (8.2)	57.00 (8.1)
Haemochromatosis diagnosis, n (%)	29 (0.02)	17 (0.04)	8 (0.2)	29 (0.6)	27 (0.1)	157 (12.1)	267 (0.1)
Self-reported fatigue, n (%)	12,094 (10.1)	4,865 (10.4)	451 (9.9)	523 (10.9)	2,476 (10.3)	148 (11.8)	20,557 (10.2)
Self-reported fatigue (60+ years), n (%),	4,475 (8.2)	1,858 (8.6)	173 (8.2)	177 (8.0)	924 (8.4)	68 (11.8)	7,675 (8.3)
Depression diagnosis, n (%)	5,883 (4.8)	2,232 (4.7)	220 (4.7)	188 (3.8)	1,146 (4.7)	71 (5.5)	9,740 (4.7)

A total of 206,390 male participants genetically similar to the 1000 Genomes project European reference population with *HFE* genotypic data available in the UK Biobank. Numbers presented are mean (SD) for continuous variables and n (%) for categorical variables. Fatigue = tiredness in more than half the days in past 2 weeks.

Lucas MR, *et al. BMJ Open* 2024; 14:e081926. doi: 10.1136/bmjopen-2023-081926

eTable 3. Baseline characteristics of female UK Biobank participants by p.C282Y/H63D genotypes

	No mutations	H63D+/-	H63D+/+	C282Y+/H63D+	C282Y+/-	C282Y+/+	Total
**Total participants**	145,694	57,021	5,580	5,760	29,221	1,604	244,880
Mean age, years (SD)	56.62 (7.9)	56.58 (7.9)	56.58 (8.1)	56.46 (7.9)	56.49 (8.0)	56.92 (8.0)	56.60 (7.9)
Haemochromatosis diagnosis, n (%)	8 (0.01)	6 (0.01)	<5	12 (0.2)	5 (0.02)	54 (3.4)	87 (0.04)
Self-reported fatigue, n (%)	19,110 (13.5)	7,449 (13.5)	760 (14.1)	785 (14.1)	3,802 (13.4)	220 (14.4)	32,126 (13.5)
Self-reported fatigue (60+ years), n (%),	6,055 (10.0)	2,417 (10.2)	253 (10.9)	229 (9.9)	1,157 (9.6)	77 (11.4)	10,188 (10.0)
Depression diagnosis, n (%)	10,734 (7.4)	4,266 (7.5)	387 (6.9)	432 (7.5)	2,271 (7.8)	123 (7.7)	18,213 (7.4)

A total of 244,880 female participants genetically similar to the 1000 Genomes project European reference population with *HFE* genotypic data available in the UK Biobank. Numbers presented are mean (SD) for continuous variables and n (%) for categorical variables. Fatigue = tiredness in more than half the days in past 2 weeks.

Lucas MR, *et al. BMJ Open* 2024; 14:e081926. doi: 10.1136/bmjopen-2023-081926

eTable 4. Incident hospital diagnoses in male UK Biobank participants by p.C282Y/H63D genotypes

Males	No mutations	H63D+/-	H63D+/+	C282Y+/ H36D+	C282Y+/-	C282Y+/+
Haemochromatosis	85 (0.1)	51 (0.1)	29 (0.6)	95 (1.9)	42 (0.2)	288 (25.2)
All-cause mortality	13,976 (11.4)	5,658 (11.8)	540 (11.6)	591 (11.9)	2,996 (12.2)	194 (15.0)
**Liver**						
Liver disease (any)	3,969 (3.3)	1,621 (3.4)	160 (3.5)	171 (3.5)	803 (3.3)	102 (8.1)
Alcoholic liver disease	608 (0.5)	227 (0.5)	25 (0.5)	28 (0.6)	150 (0.6)	14 (1.1)
Fibrosis & Cirrhosis	618 (0.5)	252 (0.5)	24 (0.5)	26 (0.5)	142 (0.6)	36 (2.8)
Hepatic failure	355 (0.3)	137 (0.3)	10 (0.2)	15 (0.3)	54 (0.2)	<5
**Cancer**						
Liver cancer	363 (0.3)	167 (0.4)	12 (0.3)	20 (0.4)	80 (0.3)	31 (2.4)
Prostate cancer	7,723 (6.4)	3,025 (6.4)	283 (6.2)	319 (6.5)	1,613 (6.7)	104 (8.1)
**Musculoskeletal**						
Joint replacement surgery (any)	8,429 (7.1)	3,379 (7.3)	315 (7.0)	376 (7.8)	1,801 (7.5)	144 (11.8)
Osteoarthritis	2,873 (2.5)	1,155 (2.6)	97 (2.2)	120 (2.6)	601 (2.6)	61 (5.5)
Fractures (any)	6,092 (5.2)	2,298 (5.1)	212 (4.8)	231 (4.9)	1,243 (5.3)	75 (6.2)
Fragility fractures	2,666 (2.2)	963 (2.0)	79 (1.7)	98 (2.0)	551 (2.3)	44 (3.4)
Osteoporosis	1,522 (1.3)	602 (1.3)	42 (0.9)	64 (1.3)	319 (1.3)	27 (2.1)
Rheumatoid arthritis	1,131 (0.9)	479 (1.0)	38 (0.8)	33 (0.7)	238 (1.0)	15 (1.2)
**Brain**						
Any brain outcome (dementia, delirium, or Parkinson's disease)	4,619 (3.8)	1,852 (3.9)	177 (3.8)	201 (4.1)	993 (4.0)	76 (5.9)
Dementia	2,306 (1.9)	916 (1.9)	92 (2.0)	97 (2.0)	487 (2.0)	40 (3.1)
Alzheimer's disease	1,026 (0.8)	380 (0.8)	38 (0.8)	50 (1.0)	189 (0.8)	13 (1.0)
Non-Alzheimer's dementia	1,299 (1.1)	540 (1.1)	54 (1.2)	47 (1.0)	303 (1.2)	27 (2.1)
Delirium	2,621 (2.1)	1,028 (2.1)	95 (2.0)	112 (2.3)	603 (2.5)	45 (3.5)
Parkinson's disease	1,116 (0.9)	454 (1.0)	46 (1.0)	43 (0.9)	202 (0.8)	21 (1.6)
**Pancreas**						
T1 or T2 diabetes	9,515 (8.0)	3,665 (7.9)	368 (8.1)	367 (7.6)	1,822 (7.7)	119 (9.6)
**Infection**						
Covid-19	2,332 (1.9)	956 (2.0)	96 (2.1)	77 (1.6)	522 (2.1)	35 (2.7)
Cholecystitis	1,624 (1.3)	613 (1.3)	55 (1.2)	73 (1.5)	318 (1.3)	24 (1.9)

Lucas MR, *et al. BMJ Open* 2024; 14:e081926. doi: 10.1136/bmjopen-2023-081926

Pneumonia	8,019 (6.7)	3,128 (6.7)	273 (6.0)	342 (7.1)	1,651 (6.9)	95 (7.6)
Sepsis	10,081 (8.4)	3,955 (8.4)	374 (8.2)	416 (8.6)	2,065 (8.6)	122 (9.7)
LRTI	4,113 (3.4)	1,694 (3.6)	148 (3.2)	164 (3.3)	914 (3.7)	50 (3.9)
URTI	588 (0.5)	226 (0.5)	21 (0.5)	17 (0.3)	109 (0.4)	<5
UTI	7,099 (5.9)	2,773 (5.9)	263 (5.7)	290 (6.0)	1,472 (6.1)	96 (7.6)
SSTI	4,604 (3.8)	1,792 (3.8)	176 (3.8)	216 (4.5)	1,053 (4.4)	66 (5.2)
**Cardiovascular**						
Arrhythmia	2,283 (1.9)	871 (1.8)	86 (1.9)	77 (1.6)	445 (1.8)	23 (1.8)
Cardiomyopathy	855 (0.7)	330 (0.7)	25 (0.5)	34 (0.7)	179 (0.7)	6 (0.5)
CHD	11,796 (10.5)	4,626 (10.5)	412 (9.6)	485 (10.5)	2,422 (10.7)	133 (11.0)
Heart failure	5,779 (4.7)	2,289 (4.8)	211 (4.6)	229 (4.7)	1,256 (5.1)	68 (5.3)
**Mental Health**						
Depression	3,929 (3.4)	1,615 (3.5)	132 (3.0)	152 (3.2)	772 (3.3)	46 (3.8)

Incident disease numbers exclude prevalent disease at baseline. Numbers presented are n (%). Abbreviations: CHD, coronary heart disease; T1, type 1; T2, type 2; LRTI, lower respiratory tract infection; URTI, upper respiratory tract infection; UTI, urinary tract infection; SSTI, skin and soft tissue infection. Joint replacement surgery variable includes a diagnosis of hip, knee, ankle, or shoulder replacement. Any brain outcome variable includes a diagnosis of dementia, delirium, or Parkinson's disease.

Lucas MR, *et al. BMJ Open* 2024; 14:e081926. doi: 10.1136/bmjopen-2023-081926

eTable 5. Hazard ratios of incident disease outcomes in p.C282Y/H63D genotypes in males

Males	No mutations	H63D +/-		H63D+/+		C282Y+/H36D +		C282Y+/-		C282Y+/+	
		HR (95% CI)	P	HR (95% CI)	P	HR (95% CI)	P	HR (95% CI)	P	HR (95% CI)	P
Haemochromatosis	1	1.53 (1.08-2.17)	0.02	8.95 (5.87-13.64)	$2.20*10^{-24}$	27.47 (20.49-36.84)	$1.00*10^{-108}$	2.40 (1.66-3.47)	$3.70*10^{-06}$	405.29 (317.06-518.11)	$2.97*10^{-501}$
All-cause mortality	1	1.03 (1.00-1.06)	0.05	1.01 (0.93-1.11)	0.75	1.02 (0.94-1.11)	0.58	1.05 (1.00-1.09)	0.03	1.29 (1.12-1.48)	$4.70*10^{-04}$
**Liver**											
Liver disease (any)	1	1.05 (0.99-1.11)	0.12	1.06 (0.90-1.24)	0.48	1.06 (0.91-1.23)	0.46	1.00 (0.93-1.78)	0.96	2.56 (2.10-3.12)	$8.70*10^{-21}$
Alcoholic liver disease	1	0.95 (0.81-1.11)	0.50	1.07 (0.71-1.59)	0.76	1.07 (0.73-1.57)	0.72	1.17 (0.98-1.40)	0.09	1.97 (1.16-3.35)	0.01
Fibrosis & Cirrhosis	1	1.04 (0.90-1.21)	0.58	1.02 (0.68-1.53)	0.93	1.00 (0.68-1.48)	0.99	1.11 (0.92-1.33)	0.28	5.36 (3.83-7.52)	$2.00*10^{-22}$
Hepatic failure	1	0.99 (0.81-1.21)	0.92	0.74 (0.40-1.40)	0.36	1.04 (0.62-1.74)	0.88	0.75 (0.56-1.00)	0.05	0.81 (0.26-2.52)	0.72
**Cancer**											
Liver cancer	1	1.17 (0.98-1.41)	0.09	0.86 (0.48-1.53)	0.61	1.32 (0.84-2.08)	0.22	1.07 (0.84-1.36)	0.61	7.90 (5.46-11.43)	$5.50*10^{-28}$
Prostate cancer	1	1.00 (0.96-1.05)	0.86	0.96 (0.86-1.09)	0.56	1.03 (0.93-1.16)	0.55	1.05 (1.00-1.11)	0.09	1.33 (1.09-1.61)	0.004
**Musculoskeletal**											
Joint replacement surgery (any)	1	1.03 (0.99-1.07)	0.21	0.98 (0.88-1.10)	0.74	1.10 (1.00-1.22)	0.08	1.06 (1.01-1.12)	0.02	1.78 (1.51-2.10)	$6.40*10^{-12}$
Osteoarthritis	1	1.03 (0.96-1.10)	0.44	0.89 (0.72-1.09)	0.25	1.01 (0.84-1.21)	0.99	1.03 (0.94-1.12)	0.55	2.10 (1.63-2.71)	$1.10*10^{-08}$
Fractures (any)	1	0.96 (0.92-1.01)	0.13	0.92 (0.80-1.05)	0.23	0.93 (0.82-1.06)	0.29	1.01 (0.95-1.07)	0.85	1.18 (0.94-1.48)	0.16
Fragility fractures	1	0.92 (0.86-0.99)	0.03	0.78 (0.62-0.98)	0.03	0.90 (0.74-1.10)	0.31	1.02 (0.93-1.12)	0.68	1.59 (1.18-2.14)	0.002
Osteoporosis	1	1.01 (0.92-1.11)	0.85	0.73 (0.53-0.99)	0.04	1.03 (0.80-1.32)	0.82	1.02 (0.91-1.16)	0.70	1.70 (1.16-2.48)	0.007
Rheumatoid arthritis	1	1.08 (0.97-1.21)	0.13	0.89 (0.64-1.23)	0.48	0.73 (0.51-1.03)	0.07	1.05 (0.91-1.20)	0.53	1.30 (0.78-2.16)	0.31
**Brain**											
Any brain outcome (dementia, delirium, or Parkinson's disease)	1	1.02 (0.97-1.08)	0.39	1.02 (0.88-1.18)	0.83	1.07 (0.93-1.24)	0.32	1.06 (0.99-1.13)	0.11	1.65 (1.31-2.06)	$1.70*10^{-05}$
Dementia	1	1.01 (0.94-1.09)	0.76	1.06 (0.86-1.31)	0.58	1.04 (0.85-1.27)	0.73	1.04 (0.94-1.14)	0.48	1.72 (1.26-2.35)	0.001
Alzheimer's disease	1	0.94 (0.84-1.06)	0.32	0.98 (0.71-1.36)	0.91	1.21 (0.91-1.60)	0.19	0.90 (0.77-1.05)	0.20	1.26 (0.73-2.17)	0.42
Non-Alzheimer's dementia	1	1.06 (0.96-1.17)	0.25	1.11 (0.84-1.45)	0.46	0.89 (0.66-1.19)	0.43	1.14 (1.01-1.30)	0.04	2.05 (1.40-3.00)	$2.40*10^{-04}$
Delirium	1	1.00 (0.93-1.08)	0.98	0.96 (0.78-1.18)	0.69	1.06 (0.87-1.28)	0.57	1.13 (1.04-1.24)	0.006	1.69 (1.26-2.27)	$4.80*10^{-04}$
Parkinson's disease	1	1.04 (0.93-1.16)	0.47	1.09 (0.81-1.47)	0.55	0.96 (0.71-1.30)	0.77	0.90 (0.77-1.04)	0.16	1.86 (1.21-2.87)	0.005
**Pancreas**											

T1 or T2 diabetes	1	0.98 (0.95-1.02)	0.44	1.02 (0.92-1.13)	0.69	0.95 (0.86-1.06)	0.35	0.95 (0.91-1.00)	0.06	1.25 (1.05-1.50)	0.01
**Infection**											
COVID-19	1	1.05 (0.97-1.13)	0.21	1.09 (0.89-1.34)	0.40	0.81 (0.65-1.02)	0.07	1.11 (1.01-1.22)	0.03	1.51 (1.08-2.11)	0.02
Cholecystitis	1	0.96 (0.88-1.06)	0.45	0.89 (0.68-1.16)	0.39	1.11 (0.87-1.40)	0.40	0.96 (0.86-1.09)	0.56	1.41 (0.94-2.11)	0.09
Pneumonia	1	1.00 (0.96-1.04)	0.82	0.90 (0.80-1.02)	0.09	1.04 (0.93-1.16)	0.51	1.01 (0.96-1.07)	0.68	1.14 (0.93-1.40)	0.21
Sepsis	1	1.01 (0.97-1.04)	0.78	0.98 (0.88-1.08)	0.66	1.02 (0.92-1.12)	0.70	1.01 (0.96-1.06)	0.70	1.17 (0.98-1.40)	0.09
LRTI	1	1.05 (0.99-1.11)	0.10	0.94 (0.80-1.11)	0.46	0.96 (0.82-1.12)	0.58	1.08 (1.00-1.16)	0.48	1.11 (0.84-1.46)	0.48
URTI	1	0.99 (0.84-1.15)	0.85	0.94 (0.61-1.45)	0.77	0.71 (0.44-1.15)	0.16	0.92 (0.75-1.13)	0.41	0.64 (0.24-1.71)	0.38
UTI	1	1.00 (0.95-1.04)	0.90	0.98 (0.86-1.10)	0.69	1.00 (0.89-1.13)	0.98	1.02 (0.96-1.08)	0.51	1.31 (1.07-1.61)	0.008
SSTI	1	1.00 (0.94-1.05)	0.88	1.01 (0.87-1.17)	0.90	1.16 (1.01-1.33)	0.03	1.14 (1.06-1.22)	$1.80*10^{-04}$	1.39 (1.09-1.77)	0.008
**Cardiovascular**											
Arrhythmia	1	0.98 (0.90-1.06)	0.57	1.00 (0.81-1.24)	0.99	0.84 (0.67-1.05)	0.13	0.96 (0.87-1.07)	0.49	0.97 (0.64-1.47)	0.89
Cardiomyopathy	1	0.99 (0.87-1.12)	0.88	0.77 (0.52-1.15)	0.21	0.99 (0.70-1.39)	0.94	1.04 (0.89-1.23)	0.60	0.69 (0.31-1.53)	0.36
CHD	1	1.01 (0.97-1.04)	0.77	0.91 (0.83-1.01)	0.08	1.00 (0.91-1.10)	0.99	1.02 (0.97-1.06)	0.45	1.06 (0.90-1.26)	0.49
Heart failure	1	1.01 (0.96-1.06)	0.67	0.96 (0.84-1.10)	0.59	0.97 (0.85-1.11)	0.65	1.07 (1.01-1.14)	0.03	1.14 (0.89-1.44)	0.29
**Mental Health**											
Depression	1	1.05 (0.99-1.12)	0.07	0.88 (0.74-1.05)	0.16	0.96 (0.81-1.13)	0.60	0.98 (0.91-1.06)	0.59	1.15 (0.86-1.54)	0.34

HR (Hazard ratio) compared to those with neither *HFE* mutation. Cox proportional hazards regression models adjusted for age, assessment centre, and genetic principal components 1–10. Abbreviations: CHD, coronary heart disease; T1, type 1; T2, type 2; LRTI, lower respiratory tract infection; URTI, upper respiratory tract infection; UTI, urinary tract infection; SSTI, skin and soft tissue infection; CI, confidence interval. Joint replacement surgery variable includes a diagnosis of hip, knee, ankle, or shoulder replacement. Any brain outcome variable includes a diagnosis of dementia, delirium, or Parkinson's disease.

Lucas MR, *et al. BMJ Open* 2024; 14:e081926. doi: 10.1136/bmjopen-2023-081926

eTable 6. Incident hospital diagnoses in male UK Biobank participants by p.C282Y/H63D genotypes, excluding a diagnosis of haemochromatosis at baseline

**Males**	**No mutations**	**H63D+/-**	**H63D+/+**	**C282Y+/H36D+**	**C282Y+/-**	**C282Y+/+**
Haemochromatosis	85 (0.1)	51 (0.1)	29 (0.6)	95 (1.9)	42 (0.2)	288 (25.2)
All-cause mortality	13,959 (11.4)	5,653 (11.8)	539 (11.6)	587 (11.9)	2,990 (12.2)	160 (14.0)
**Liver**						
Liver disease (any)	3,969 (3.3)	1,619 (3.4)	158 (3.4)	167 (3.4)	800 (3.3)	85 (7.5)
Alcoholic liver disease	607 (0.5)	226 (0.5)	25 (0.5)	27 (0.6)	148 (0.6)	10 (0.9)
Fibrosis & Cirrhosis	618 (0.5)	250 (0.5)	23 (0.5)	25 (0.5)	140 (0.6)	27 (2.4)
Hepatic failure	355 (0.3)	137 (0.3)	10 (0.2)	15 (0.3)	54 (0.2)	<5
**Cancer**						
Liver cancer	361 (0.3)	166 (0.4)	12 (0.3)	19 (0.4)	78 (0.3)	17 (1.5)
Prostate cancer	7,722 (6.4)	3,023 (6.4)	283 (6.2)	318 (6.6)	1,610 (6.7)	87 (7.7)
**Musculoskeletal**						
Joint replacement surgery (any)	8,427 (7.1)	3,377 (7.3)	315 (7.0)	375 (7.8)	1,801 (7.5)	126 (11.6)
Osteoarthritis	2,872 (2.5)	1,153 (2.6)	97 (2.2)	120 (2.7)	600 (2.6)	51 (5.1)
Fractures (any)	6,090 (5.2)	2,296 (5.1)	212 (4.8)	231 (5.0)	1,243 (5.3)	66 (6.2)
Fragility fractures	2,663 (2.2)	963 (2.0)	79 (1.7)	98 (2.0)	550 (2.3)	39 (3.5)
Osteoporosis	1,521 (1.3)	601 (1.3)	42 (0.9)	63 (1.3)	319 (1.3)	16 (1.4)
Rheumatoid arthritis	1,130 (0.9)	479 (1.0)	38 (0.8)	33 (0.7)	238 (1.0)	12 (1.1)
**Brain**						
Any brain outcome (dementia, delirium, or Parkinson's disease)	4,616 (3.8)	1,849 (3.9)	177 (3.8)	200 (4.1)	992 (4.1)	66 (5.8)
Dementia	2,305 (1.9)	915 (1.9)	92 (2.0)	97 (2.0)	487 (2.0)	33 (2.9)
Alzheimer's disease	1,026 (0.8)	380 (0.8)	38 (0.8)	50 (1.0)	189 (0.8)	12 (1.1)
Non-Alzheimer's dementia	1,298 (1.1)	539 (1.1)	54 (1.2)	47 (1.0)	303 (1.2)	21 (1.9)
Delirium	2,619 (2.1)	1,025 (2.1)	95 (2.0)	111 (2.3)	602 (2.5)	41 (3.6)
Parkinson's disease	1,115 (0.9)	454 (1.0)	46 (1.0)	43 (0.9)	202 (0.8)	19 (1.7)
**Pancreas**						
T1 or T2 diabetes	9,510 (8.0)	3,663 (7.9)	368 (8.1)	368 (7.6)	1,820 (7.7)	99 (9.0)
**Infection**						

Lucas MR, *et al. BMJ Open* 2024; 14:e081926. doi: 10.1136/bmjopen-2023-081926

Covid-19	2,331 (1.9)	953 (2.0)	96 (2.1)	76 (1.5)	522 (2.1)	31 (2.7)
Cholecystitis	1,623 (1.3)	612 (1.3)	55 (1.2)	73 (1.5)	316 (1.3)	23 (2.0)
Pneumonia	8,014 (6.7)	3,124 (6.7)	273 (6.0)	340 (7.1)	1,649 (6.9)	83 (7.5)
Sepsis	10,075 (8.4)	3,951 (8.4)	374 (8.2)	412 (8.5)	2,063 (8.6)	103 (9.3)
LRTI	4,110 (3.4)	1,691 (3.6)	148 (3.2)	164 (3.4)	913 (3.8)	45 (4.0)
URTI	588 (0.5)	226 (0.5)	21 (0.5)	17 (0.4)	108 (0.4)	<5
UTI	7,097 (5.9)	2,769 (5.9)	263 (5.7)	286 (5.9)	1,470 (6.1)	85 (7.6)
SSTI	4,599 (3.8)	1,790 (3.8)	176 (3.8)	215 (4.5)	1,053 (4.4)	59 (5.3)
**Cardiovascular**						
Arrhythmia	2,282 (1.9)	870 (1.8)	85 (1.8)	77 (1.6)	444 (1.8)	20 (1.8)
Cardiomyopathy	853 (0.7)	330 (0.7)	25 (0.5)	34 (0.7)	178 (0.7)	5 (0.4)
CHD	11,793 (10.5)	4,622 (10.5)	411 (9.6)	481 (10.5)	2,414 (10.7)	115 (10.7)
Heart failure	5,775 (4.7)	2,285 (4.8)	211 (4.6)	227 (4.6)	1,253 (5.1)	59 (5.2)
**Mental Health**						
Depression	3,925 (3.4)	1,614 (3.5)	132 (3.0)	150 (3.2)	771 (3.3)	40 (3.7)

Incident disease numbers exclude prevalent disease at baseline. Numbers presented are n (%). Abbreviations: CHD, coronary heart disease; T1, type 1; T2, type 2; LRTI, lower respiratory tract infection; URTI, upper respiratory tract infection; UTI, urinary tract infection; SSTI, skin and soft tissue infection. Joint replacement surgery variable includes a diagnosis of hip, knee, ankle, or shoulder replacement. Any brain outcome variable includes a diagnosis of dementia, delirium, or Parkinson’s disease.

Lucas MR, *et al. BMJ Open* 2024; 14:e081926. doi: 10.1136/bmjopen-2023-081926

eTable 7. Hazard ratios of incident disease outcomes in p.C282Y/H63D genotypes in males, excluding a diagnosis of haemochromatosis at baseline

**Males**	**No mutations (Ref group)**	**H63D+/-**		**H63D+/+**		**C282Y+/H36D+**		**C282Y+/-**		**C282Y+/+**	
		HR (95% CI)	P	HR (95% CI)	P	HR (95% CI)	P	HR (95% CI)	P	HR (95% CI)	P
Haemochromatosis	1	1.53 (1.08-2.17)	0.02	8.95 (5.87-13.64)	$2.2*10^{-24}$	27.49 (20.50-36.86)	$1.0*10^{-108}$	2.40 (1.66-3.47)	$3.6*10^{-06}$	405.79 (317.34-518.75)	0.00E+00
All-cause mortality	1	1.03 (1.00-1.07)	0.04	1.01 (0.93-1.11)	0.74	1.02 (0.94-1.11)	0.58	1.05 (1.00-1.09)	0.03	1.22 (1.05-1.43)	0.01
**Liver**											
Liver disease (any)	1	1.05 (0.99-1.11)	0.13	1.05 (0.89-1.23)	0.57	1.04 (0.89-1.21)	0.63	1.00 (0.92-1.07)	0.90	2.36 (1.90-2.93)	$5.3*10^{-15}$
Alcoholic liver disease	1	0.95 (0.12-1.10)	0.48	1.07 (0.72-1.60)	0.74	1.04 (0.71-1.53)	0.84	1.15 (0.96-1.38)	0.12	1.59 (0.85-2.97)	0.15
Fibrosis & Cirrhosis	1	1.03 (0.89-1.20)	0.66	0.98 (0.64-1.48)	0.91	0.97 (0.65-1.44)	0.87	1.09 (0.91-1.31)	0.35	4.52 (3.07-6.66)	$2.1*10^{-14}$
Hepatic failure	1	0.99 (0.81-1.21)	0.92	0.75 (0.40-1.40)	0.36	1.05 (0.62-1.76)	0.86	0.75 (0.56-1.00)	0.05	0.31 (0.04-2.19)	0.24
**Cancer**											
Liver cancer	1	1.17 (0.98-1.41)	0.09	0.87 (0.49-1.54)	0.63	1.27 (0.80-2.02)	0.30	1.05 (0.82-1.34)	0.71	4.97 (3.05-8.11)	$1.2*10^{-10}$
Prostate cancer	1	1.00 (0.96-1.05)	0.87	0.97 (0.86-1.09)	0.57	1.04 (0.93-1.16)	0.51	1.05 (0.99-1.11)	0.09	1.27 (1.03-1.57)	0.03
**Musculoskeletal**											
Joint replacement surgery (any)	1	1.03 (0.99-1.07)	0.22	0.98 (0.88-1.10)	0.75	1.10 (0.99-1.22)	0.06	1.06 (1.01-1.12)	0.02	1.75 (1.47-2.09)	$4.8*10^{-10}$
Osteoarthritis	1	1.03 (0.96-1.10)	0.46	0.89 (0.73-1.09)	0.26	1.02 (0.85-1.22)	0.87	1.03 (0.94-1.12)	0.56	1.97 (1.50-2.60)	$1.5*10^{-06}$
Fractures (any)	1	0.96 (0.92-1.01)	0.12	0.92 (0.80-1.06)	0.24	0.94 (0.82-1.07)	0.34	1.01 (0.95-1.07)	0.82	1.17 (0.91-1.49)	0.22
Fragility fractures	1	0.92 (0.86-0.99)	0.03	0.78 (0.63-0.98)	0.03	0.91 (0.74-1.11)	0.34	1.02 (0.93-1.12)	0.68	1.60 (1.17-2.20)	$3.6*10^{-03}$
Osteoporosis	1	1.01 (0.92-1.11)	0.86	0.73 (0.54-0.99)	0.04	1.02 (0.79-1.31)	0.89	1.03 (0.91-1.16)	0.68	1.14 (0.69-1.86)	0.61
Rheumatoid arthritis	1	1.09 (0.98-1.21)	0.13	0.89 (0.64-1.23)	0.48	0.73 (0.52-1.03)	0.07	1.05 (0.91-1.20)	0.52	1.17 (0.66-2.07)	0.58
**Brain**											
Any brain outcome (dementia, delirium, or Parkinson's disease)	1	1.02 (0.97-1.08)	0.40	1.02 (0.88-1.18)	0.80	1.08 (0.93-1.24)	0.31	1.06 (0.99-1.13)	0.10	1.62 (1.27-2.07)	$1.0*10^{-04}$
Dementia	1	1.01 (0.94-1.09)	0.76	1.06 (0.86-1.31)	0.57	1.04 (0.85-1.28)	0.69	1.04 (0.94-1.14)	0.46	1.60 (1.14-2.26)	0.01

Alzheimer's disease	1	0.94 (0.84-1.06)	0.32	0.98 (0.71-1.36)	0.92	1.12 (0.91-1.61)	0.18	0.90 (0.77-1.06)	0.20	1.31 (0.74-2.31)	0.35
Non-Alzheimer's dementia	1	1.06 (0.96-1.17)	0.26	1.11 (0.85-1.46)	0.45	0.89 (0.67-1.20)	0.45	1.14 (1.01-1.30)	0.04	1.81 (1.17-2.78)	0.01
Delirium	1	1.00 (0.93-1.07)	0.98	0.96 (0.78-1.18)	0.70	1.05 (0.87-1.27)	0.59	1.13 (1.04-1.24)	0.01	1.75 (1.28-2.38)	$3.9*10^{-04}$
Parkinson's disease	1	1.07 (0.98-1.16)	0.13	1.05 (0.84-1.31)	0.67	1.06 (0.85-1.33)	0.58	1.01 (0.91-1.13)	0.79	1.44 (1.02-2.03)	0.04
**Pancreas**											
T1 or T2 diabetes	1	0.99 (0.95-1.02)	0.45	1.02 (0.92-1.14)	0.66	0.94 (0.85-1.05)	0.28	0.95 (0.91-1.00)	0.06	1.17 (0.96-1.43)	0.11
**Infection**											
COVID-19	1	1.05 (0.97-1.13)	0.23	1.09 (0.89-1.34)	0.39	0.81 (0.64-1.01)	0.07	1.11 (1.01-1.23)	0.03	1.51 (1.06-2.15)	0.02
Cholecystitis	1	0.96 (0.88-1.06)	0.44	0.89 (0.68-1.17)	0.40	1.11 (0.88-1.41)	0.37	0.96 (0.85-1.08)	0.51	1.54 (1.02-2.32)	0.04
Pneumonia	1	0.99 (0.95-1.04)	0.80	0.90 (0.80-1.02)	0.09	1.04 (0.93-1.16)	0.52	1.01 (0.96-1.07)	0.66	1.13 (0.91-1.40)	0.27
Sepsis	1	1.01 (0.97-1.04)	0.79	0.98 (0.88-1.09)	0.69	1.01 (0.92-1.12)	0.82	1.01 (0.96-1.06)	0.68	1.12 (0.92-1.36)	0.26
LRTI	1	1.05 (0.99-1.11)	0.10	0.94 (0.80-1.11)	0.48	0.96 (0.82-1.13)	0.63	1.08 (1.00-1.16)	0.04	1.14 (0.85-1.52)	0.40
URTI	1	0.99 (0.85-1.15)	0.85	0.94 (0.61-1.45)	0.78	0.71 (0.44-1.16)	0.17	0.91 (0.74-1.12)	0.37	0.55 (0.18-1.70)	0.30
UTI	1	1.00 (0.95-1.04)	0.86	0.98 (0.86-1.11)	0.71	1.00 (0.89-1.12)	0.96	1.02 (0.96-1.08)	0.51	1.32 (1.07-1.64)	0.01
SSTI	1	1.00 (0.94-1.05)	0.89	1.01 (0.87-1.18)	0.87	1.16 (1.01-1.33)	0.03	1.14 (1.07-1.22)	$1.4*10^{-04}$	1.40 (1.09-1.82)	0.01
**Cardiovascular**											
Arrhythmia	1	0.98 (0.90-1.06)	0.56	0.99 (0.80-1.23)	0.92	0.84 (0.67-1.06)	0.15	0.96 (0.87-1.07)	0.48	0.96 (0.62-1.49)	0.86
Cardiomyopathy	1	0.99 (0.87-1.13)	0.91	0.78 (0.52-1.16)	0.21	0.99 (0.71-1.40)	0.98	1.04 (0.89-1.22)	0.62	0.65 (0.27-1.57)	0.34
CHD	1	1.00 (0.97-1.04)	0.80	0.91 (0.83-1.01)	0.71	1.00 (0.91-1.09)	0.97	1.01 (0.97-1.06)	0.52	1.04 (0.87-1.25)	0.66
Heart failure	1	1.01 (0.96-1.06)	0.69	0.96 (0.84-1.11)	0.61	0.97 (0.85-1.10)	0.62	1.07 (1.01-1.14)	0.03	1.12 (0.87-1.45)	0.38
**Mental Health**											
Depression	1	1.05 (1.00-1.12)	0.07	0.88 (0.74-1.05)	0.17	0.95 (0.81-1.12)	0.55	0.98 (0.91-1.06)	0.60	1.13 (0.82-1.54)	0.46

HR (Hazard ratio) compared to those with neither *HFE* mutation. Cox proportional hazards regression models adjusted for age, assessment centre, and genetic principal components 1–10. Abbreviations: CHD, coronary heart disease; T1, type 1; T2, type 2; LRTI, lower respiratory tract infection; URTI, upper respiratory tract infection; UTI, urinary tract infection SSTI, skin and soft tissue infection; CI, confidence interval. Joint replacement surgery variable includes a diagnosis of hip, knee, ankle, or shoulder replacement. Any brain outcome variable includes a diagnosis of dementia, delirium, or Parkinson's disease.

eTable 8. Cumulative incidence of haemochromatosis from ages 40-80 years by HFE genotypes

	Total Cohort (n=451,270)			No mutations			H63D+/-			H63D+/+			C282Y+/H36D+			C282Y+/-			C282Y+/+		
	Incident diagnosis			Incident diagnosis			Incident diagnosis			Incident diagnosis			Incident diagnosis			Incident diagnosis			Incident diagnosis		
	%	(95% CI)		%	(95% CI)		%	(95% CI)		%	(95% CI)		%	(95% CI)		%	(95% CI)		%	(95% CI)	
**Male age group (years)**																					
**40 - 45**	0.1	0.0	0.2	0.0	0.0	0.2	0.0	0.0	0.0	0.0	0.0	0.0	0.3	0.0	1.9	0.0	0.0	0.0	6.8	2.8	16.1
**46 - 50**	0.1	0.1	0.2	0.0	0.0	0.2	0.0	0.0	0.1	0.4	0.1	1.3	0.7	0.2	2.0	0.0	0.0	0.0	12.5	7.3	20.8
**51 - 55**	0.2	0.2	0.3	0.0	0.0	0.2	0.0	0.0	0.1	0.6	0.3	1.5	1.5	0.8	2.6	0.0	0.0	0.1	20.4	14.7	27.9
**56 - 60**	0.3	0.3	0.4	0.1	0.0	0.2	0.1	0.0	0.1	0.8	0.4	1.6	2.1	1.3	3.2	0.1	0.1	0.2	28.4	22.7	35.3
**61 - 65**	0.4	0.3	0.5	0.1	0.0	0.2	0.1	0.1	0.2	1.0	0.6	1.8	2.6	1.8	3.8	0.2	0.1	0.3	36.1	30.5	42.4
**66 - 70**	0.5	0.5	0.6	0.1	0.1	0.2	0.2	0.1	0.2	1.2	0.7	2.1	3.6	2.7	4.8	0.3	0.2	0.4	43.0	37.6	48.7
**71 - 75**	0.6	0.6	0.7	0.2	0.1	0.2	0.2	0.1	0.3	1.4	0.9	2.2	4.3	3.4	5.5	0.3	0.2	0.4	48.9	43.8	54.2
**76 - 80**	0.8	0.7	0.9	0.2	0.1	0.3	0.3	0.2	0.4	1.9	1.3	2.9	5.4	4.3	6.8	0.4	0.3	0.6	56.4	51.4	61.6
**Female age group (years)**																					
**40 - 45**	0.0	0.0	0.0	0.0	0.0	0.0	0.0	0.0	0.0	0.0	0.0	0.0	0.2	0.0	1.5	0.0	0.0	0.0	0.0	0.0	0.0
**46 - 50**	0.0	0.0	0.1	0.0	0.0	0.0	0.0	0.0	0.1	0.2	0.1	0.9	0.3	0.1	1.3	0.0	0.0	0.0	3.4	1.7	6.7
**51 - 55**	0.1	0.1	0.1	0.0	0.0	0.0	0.0	0.0	0.1	0.3	0.1	1.0	0.5	0.2	1.3	0.0	0.0	0.1	8.2	5.7	11.7
**56 - 60**	0.1	0.1	0.2	0.0	0.0	0.0	0.1	0.0	0.1	0.4	0.2	1.0	0.7	0.4	1.5	0.1	0.1	0.2	14.5	11.5	18.1
**61 - 65**	0.2	0.2	0.3	0.0	0.0	0.0	0.1	0.0	0.1	0.5	0.2	1.1	1.1	0.6	1.8	0.1	0.1	0.2	21.2	17.9	24.9
**66 - 70**	0.3	0.3	0.3	0.0	0.0	0.1	0.1	0.1	0.2	0.5	0.2	1.1	1.5	1.0	2.2	0.2	0.1	0.3	27.6	24.2	31.3
**71 - 75**	0.4	0.3	0.4	0.1	0.0	0.1	0.1	0.1	0.2	0.6	0.3	1.2	1.9	1.4	2.7	0.2	0.2	0.3	33.5	30.0	37.2
**76 - 80**	0.5	0.5	0.6	0.1	0.1	0.1	0.1	0.1	0.2	0.6	0.3	1.3	2.7	2.0	3.6	0.3	0.2	0.5	40.5	36.7	44.5

Lucas MR, *et al. BMJ Open* 2024; 14:e081926. doi: 10.1136/bmjopen-2023-081926

eTable 9. Incident hospital diagnoses in female UK Biobank participants by p.C282Y/H63D genotypes

**Females**	**No mutations**	**H63D+/-**	**H63D+/+**	**C282Y+/H36D+**	**C282Y+/-**	**C282Y+/+**
Haemochromatosis	44 (0.03)	35 (0.1)	12 (0.2)	57 (1.0)	37 (0.1)	291 (18.8)
All-cause mortality	9,849 (6.8)	3,810 (6.7)	363 (6.5)	393 (6.8)	2,020 (6.9)	129 (8.0)
**Liver**						
Liver disease (any)	3,822 (2.6)	1,512 (2.7)	146 (2.6)	179 (3.1)	825 (2.9)	69 (4.3)
Alcoholic liver disease	181 (0.1)	78 (0.1)	11 (0.2)	6 (0.1)	59 (0.2)	7 (0.4)
Fibrosis & Cirrhosis	475 (0.3)	163 (0.3)	16 (0.3)	21 (0.4)	89 (0.3)	14 (0.9)
Hepatic failure	200 (0.1)	60 (0.1)	7 (0.1)	<5	35 (0.1)	5 (0.3)
**Cancer**						
Liver cancer	232 (0.2)	114 (0.2)	10 (0.2)	8 (0.1)	49 (0.2)	<5
**Musculoskeletal**						
Joint replacement surgery (any)	12,356 (8.7)	4,772 (8.6)	454 (8.4)	501 (8.9)	2,415 (8.5)	158 (10.3)
Osteoarthritis	4,344 (3.3)	1,681 (3.3)	177 (3.6)	190 (3.7)	893 (3.4)	70 (5.1)
Fractures (any)	10,666 (7.6)	4,204 (7.7)	376 (7.0)	430 (7.7)	2,189 (7.8)	124 (8.0)
Fragility fractures	6,308 (4.4)	2,517 (4.5)	214 (3.9)	275 (4.8)	1,265 (4.4)	63 (4.0)
Osteoporosis	6,990 (5.0)	2,580 (4.7)	254 (4.7)	245 (4.4)	1,390 (4.9)	103 (6.7)
Rheumatoid arthritis	2,189 (1.5)	819 (1.5)	79 (1.4)	87 (1.5)	391 (1.4)	26 (1.6)
**Brain**						
Any brain outcome (dementia, delirium, or Parkinson's disease)	3,876 (2.7)	1,547 (2.7)	142 (2.6)	155 (2.7)	777 (2.7)	61 (3.8)
Dementia	2,159 (1.5)	837 (1.5)	68 (1.2)	84 (1.5)	440 (1.5)	31 (1.9)
Alzheimer's disease	1,072 (0.7)	450 (0.8)	37 (0.7)	51 (0.9)	220 (0.8)	14 (0.9)
Non-Alzheimer's dementia	1,099 (0.8)	395 (0.7)	31 (0.6)	34 (0.6)	227 (0.8)	17 (1.1)
Delirium	1,983 (1.4)	823 (1.4)	81 (1.5)	82 (1.4)	406 (1.4)	36 (2.2)
Parkinson's disease	691 (0.5)	249 (0.4)	26 (0.5)	30 (0.5)	134 (0.5)	8 (0.5)
**Pancreas**						
T1 or T2 diabetes	6,532 (4.6)	2,583 (4.6)	245 (4.5)	278 (4.9)	1,327 (4.6)	87 (5.5)
**Infection**						
Covid-19	1,809 (1.2)	720 (1.3)	72 (1.3)	72 (1.3)	383 (1.3)	21 (1.3)
Cholecystitis	1,749 (1.2)	675 (1.2)	62 (1.1)	69 (1.2)	351 (1.2)	30 (1.9)
Pneumonia	6,130 (4.3)	2,408 (4.3)	242 (4.4)	255 (4.5)	1,276 (4.5)	75 (4.8)

Lucas MR, *et al. BMJ Open* 2024; 14:e081926. doi: 10.1136/bmjopen-2023-081926

Sepsis	10,026 (7.0)	3,840 (6.9)	379 (7.0)	414 (7.4)	2,103 (7.4)	133 (8.5)
LRTI	3,875 (2.7)	1,439 (2.5)	139 (2.5)	144 (2.5)	821 (2.8)	53 (3.3)
URTI	636 (0.4)	279 (0.5)	21 (0.4)	21 (0.4)	142 (0.5)	<5
UTI	9,140 (6.5)	3,493 (6.4)	351 (6.6)	366 (6.7)	1,905 (6.8)	126 (8.2)
SSTI	3,900 (2.7)	1,534 (2.7)	158 (2.9)	153 (2.7)	799 (2.8)	53 (3.3)
**Cardiovascular**						
Arrhythmia	1,655 (1.1)	656 (1.2)	48 (0.9)	67 (1.2)	323 (1.1)	20 (1.3)
Cardiomyopathy	608 (0.4)	230 (0.4)	19 (0.3)	26 (0.5)	130 (0.5)	<5
CHD	7,402 (5.3)	2,877 (5.2)	294 (5.4)	293 (5.3)	1,502 (5.3)	82 (5.3)
Heart failure	3,551 (2.4)	1,431 (2.5)	127 (2.3)	155 (2.7)	753 (2.6)	56 (3.5)
**Mental Health**						
Depression	6,415 (4.8)	2,430 (4.6)	269 (5.2)	279 (5.2)	1,317 (4.9)	64 (4.3)

Incident disease numbers exclude prevalent disease at baseline. Numbers presented are n (%). Abbreviations: CHD, coronary heart disease; T1, type 1; T2, type 2; LRTI, lower respiratory tract infection; URTI, upper respiratory tract infection; UTI, urinary tract infection; SSTI, skin and soft tissue infection. Joint replacement surgery variable includes a diagnosis of hip, knee, ankle, or shoulder replacement. Any brain outcome variable includes a diagnosis of dementia, delirium, or Parkinson's disease.

eTable 10. Hazard ratios of incident disease outcomes in p.C282Y/H63D genotypes in females

**Females**	**No mutations**	**H63D+/-**		**H63D+/+**		**C282Y+/H36D+**		**C282Y+/-**		**C282Y+/+**	
		HR (95% CI)	P	HR (95% CI)	P	HR (95% CI)	P	HR (95% CI)	P	HR (95% CI)	P
Haemochromatosis	1	2.04 (1.31-3.17)	$1.70*10^{-03}$	7.10 (3.75-13.45)	$1.80*10^{-09}$	32.78 (22.10-48.63)	$2.20*10^{-67}$	4.15 (2.68-6.44)	$1.80*10^{-10}$	674.10 (489.11-929.05)	$2.47*10^{-346}$
All-cause mortality	1	0.99 (0.96-1.03)	0.68	0.95 (0.85-1.05)	0.30	1.01 (0.91-1.11)	0.90	1.01 (0.96-1.06)	0.62	1.10 (0.92-1.31)	0.28
**Liver**											
Liver disease (any)	1	1.01 (0.95-1.07)	0.72	1.00 (0.84-1.17)	0.96	1.19 (1.02-1.38)	0.03	1.08 (1.00-1.16)	0.06	1.62 (1.27-2.05)	$7.80*10^{-05}$
Alcoholic liver disease	1	1.09 (0.84-1.43)	0.51	1.56 (0.85-2.87)	0.15	0.78 (0.35-1.77)	0.56	1.53 (1.14-2.05)	0.005	3.07 (1.44-6.54)	0.004
Fibrosis & Cirrhosis	1	0.87 (0.73-1.04)	0.13	0.87 (0.53-1.43)	0.58	1.10 (0.71-1.71)	0.67	0.92 (0.74-1.16)	0.49	2.56 (1.50-4.36)	0.001
Hepatic failure	1	0.77 (0.58-1.02)	0.07	0.92 (0.43-1.94)	0.82	0.49 (0.18-1.33)	0.16	0.85 (0.59-1.21)	0.37	2.09 (0.86-5.08)	0.10
**Cancer**											
Liver cancer	1	1.26 (1.01-1.58)	0.04	1.13 (0.60-2.13)	0.71	0.89 (0.44-1.81)	0.75	1.06 (0.78-1.45)	0.69	1.17 (0.37-3.66)	0.79
**Musculoskeletal**											
Joint replacement surgery (any)	1	0.99 (0.96-1.02)	0.55	0.97 (0.88-1.06)	0.47	1.04 (0.95-1.14)	0.34	0.99 (0.94-1.03)	0.51	1.18 (1.01-1.38)	0.04
Osteoarthritis	1	0.99 (0.94-1.05)	0.71	1.06 (0.91-1.23)	0.46	1.10 (0.95-1.27)	0.21	1.02 (0.95-1.10)	0.60	1.44 (1.14-1.82)	0.002
Fractures (any)	1	1.01 (0.97-1.04)	0.65	0.91 (0.82-1.01)	0.07	1.01 (0.92-1.12)	0.79	1.02 (0.97-1.07)	0.42	1.00 (0.84-1.20)	0.96
Fragility fractures	1	1.02 (0.98-1.07)	0.38	0.87 (0.76-1.00)	0.05	1.10 (0.97-1.24)	0.13	0.99 (0.93-1.05)	0.74	0.85 (0.66-1.09)	0.19
Osteoporosis	1	0.94 (0.90-0.99)	0.01	0.94 (0.83-1.07)	0.34	0.89 (0.79-1.01)	0.08	0.99 (0.94-1.05)	0.75	1.28 (1.06-1.56)	0.01
Rheumatoid arthritis	1	0.96 (0.88-1.04)	0.28	0.94 (0.75-1.17)	0.58	1.01 (0.82-1.26)	0.90	0.89 (0.80-0.99)	0.03	1.04 (0.71-1.53)	0.84
**Brain**											
Any brain outcome (dementia, delirium, or Parkinson's disease)	1	1.02 (0.97-1.09)	0.43	0.93 (0.79-1.11)	0.43	1.03 (0.88-1.21)	0.72	1.00 (0.92-1.08)	0.90	1.30 (1.01-1.68)	0.04
Dementia	1	1.00 (0.92-1.08)	0.94	0.80 (0.63-1.02)	0.07	1.00 (0.81-1.25)	0.98	1.01 (0.91-1.12)	0.86	1.16 (0.81-1.66)	0.41
Alzheimer's disease	1	1.08 (0.97-1.20)	0.18	0.87 (0.63-1.20)	0.40	1.22 (0.92-1.61)	0.17	1.02 (0.88-1.17)	0.84	1.04 (0.62-1.77)	0.88

Non-Alzheimer's dementia	1	0.93 (0.83-1.04)	0.20	0.72 (0.50-1.03)	0.07	0.81 (0.57-1.13)	0.21	1.03 (0.89-1.18)	0.73	1.27 (0.76-2.05)	0.33
Delirium	1	1.06 (0.98-1.15)	0.13	1.05 (0.84-1.31)	0.67	1.06 (0.85-1.32)	0.60	1.01 (0.91-1.13)	0.80	1.50 (1.08-2.08)	0.02
Parkinson's disease	1	0.93 (0.80-1.07)	0.29	0.97 (0.66-1.44)	0.89	1.14 (0.79-1.65)	0.48	0.98 (0.81-1.18)	0.84	1.00 (0.50-2.00)	0.99
**Pancreas**											
T1 or T2 diabetes	1	1.01 (0.97-1.06)	0.55	0.98 (0.86-1.11)	0.77	1.09 (0.97-1.23)	0.17	1.01 (0.96-1.08)	0.60	1.18 (0.95-1.46)	0.13
**Infection**											
COVID-19	1	1.02 (0.94-1.11)	0.66	1.03 (0.81-1.30)	0.83	1.02 (0.80-1.29)	0.90	1.06 (0.94-1.18)	0.34	1.00 (0.65-1.54)	0.99
Cholecystitis	1	0.98 (0.90-1.08)	0.73	0.92 (0.71-1.18)	0.52	0.99 (0.78-1.26)	0.92	0.99 (0.88-1.11)	0.86	1.49 (1.04-2.13)	0.03
Pneumonia	1	1.01 (0.96-1.05)	0.83	1.02 (0.90-1.16)	0.72	1.06 (0.93-1.20)	0.40	1.03 (0.97-1.10)	0.29	1.05 (0.83-1.32)	0.69
Sepsis	1	0.98 (0.94-1.01)	0.23	0.97 (0.88-1.08)	0.61	1.05 (0.95-1.15)	0.36	1.04 (0.99-1.09)	0.12	1.13 (0.96-1.35)	0.15
LRTI	1	0.95 (0.89-1.01)	0.08	0.92 (0.78-1.09)	0.34	0.93 (0.79-1.10)	0.39	1.04 (0.97-1.12)	0.30	1.16 (0.89-1.52)	0.28
URTI	1	1.12 (0.98-1.30)	0.10	0.86 (0.56-1.33)	0.51	0.84 (0.54-1.30)	0.43	1.12 (0.93-1.34)	0.24	0.28 (0.07-1.12)	0.07
UTI	1	0.97 (0.94-1.01)	0.18	0.99 (0.89-1.10)	0.82	1.01 (0.91-1.13)	0.80	1.03 (0.98-1.08)	0.23	1.19 (0.99-1.41)	0.06
SSTI	1	1.01 (0.95-1.07)	0.79	1.05 (0.90-1.23)	0.54	1.00 (0.85-1.18)	0.97	1.03 (0.95-1.11)	0.52	1.20 (0.92-1.58)	0.18
**Cardiovascular**											
Arrhythmia	1	1.02 (0.93-1.11)	0.69	0.76 (0.57-1.01)	0.06	1.04 (0.81-1.33)	0.75	0.98 (0.87-1.11)	0.78	1.07 (0.69-1.67)	0.75
Cardiomyopathy	1	0.97 (0.83-1.13)	0.71	0.82 (0.52-1.30)	0.41	1.11 (0.75-1.64)	0.61	1.08 (0.90-1.31)	0.41	0.44 (0.14-1.37)	0.16
CHD	1	1.00 (0.95-1.04)	0.82	1.03 (0.92-1.16)	0.61	1.01 (0.90-1.14)	0.86	1.01 (0.96-1.07)	0.71	0.94 (0.76-1.17)	0.60
Heart failure	1	1.04 (0.97-1.10)	0.27	0.92 (0.77-1.10)	0.35	1.12 (0.96-1.32)	0.15	1.06 (0.98-1.15)	0.16	1.34 (1.03-1.75)	0.03
**Mental Health**											
Depression	1	0.97 (0.93-1.02)	0.20	1.10 (0.97-1.24)	0.14	1.11 (0.99-1.25)	0.09	1.03 (0.97-1.09)	0.38	0.90 (0.70-1.15)	0.39

HR (Hazard ratio) compared to those with neither *HFE* mutation. Cox proportional hazards regression models adjusted for age, assessment centre, and genetic principal components 1–10. Abbreviations: CHD, coronary heart disease; T1, type 1; T2, type 2; LRTI, lower respiratory tract infection; URTI, upper respiratory tract infection; UTI, urinary tract infection SSTI, skin and soft tissue infection; CI, confidence interval. Joint replacement surgery variable includes a diagnosis of hip, knee, ankle, or shoulder replacement. Any brain outcome variable includes a diagnosis of dementia, delirium, or Parkinson's disease.

eTable 11. Incident hospital diagnoses in female UK Biobank participants by p.C282Y/H63D genotypes, excluding a diagnosis of haemochromatosis at baseline

Females	No mutations	H63D+/-	H63D+/+	C282Y+/H36D+	C282Y+/-	C282Y+/+
Haemochromatosis	44 (0.03)	35 (0.1)	12 (0.2)	57 (1.0)	37 (0.1)	291 (18.8)
All-cause mortality	9,847 (6.8)	3,810 (6.7)	363 (6.5)	390 (6.8)	2,020 (6.9)	127 (8.2)
**Liver**						
Liver disease (any)	3,821 (2.6)	1,511 (2.7)	146 (2.6)	177 (3.1)	825 (2.9)	65 (4.2)
Alcoholic liver disease	180 (0.1)	78 (0.1)	11 (0.2)	6 (0.1)	59 (0.2)	6 (0.4)
Fibrosis & Cirrhosis	473 (0.3)	162 (0.3)	16 (0.3)	21 (0.4)	89 (0.3)	14 (0.9)
Hepatic failure	199 (0.1)	60 (0.1)	7 (0.1)	<5	35 (0.1)	<5
**Cancer**						
Liver cancer	232 (0.2)	114 (0.2)	10 (0.2)	8 (0.1)	49 (0.2)	<5
**Musculoskeletal**						
Joint replacement surgery (any)	12,355 (8.7)	4,771 (8.6)	454 (8.4)	500 (8.9)	2,415 (8.5)	150 (10.1)
Osteoarthritis	4,343 (3.3)	1,681 (3.3)	177 (3.6)	189 (3.7)	893 (3.4)	68 (5.1)
Fractures (any)	10,665 (7.6)	4,204 (7.7)	375 (7.0)	430 (7.7)	2,188 (7.8)	119 (8.0)
Fragility fractures	6,308 (4.4)	2,517 (4.5)	214 (3.9)	275 (4.8)	1,264 (4.4)	61 (4.0)
Osteoporosis	6,990 (5.0)	2,580 (4.7)	254 (4.7)	244 (4.4)	1,389 (4.9)	97 (6.5)
Rheumatoid arthritis	2,188 (1.5)	818 (1.5)	79 (1.4)	86 (1.5)	390 (1.4)	23 (1.5)
**Brain**						
Any brain outcome (dementia, delirium, or Parkinson's disease)	3,875 (2.7)	1,547 (2.7)	142 (2.6)	154 (2.7)	777 (2.7)	55 (3.6)
Dementia	2,159 (1.5)	837 (1.5)	68 (1.2)	83 (1.4)	440 (1.5)	28 (1.8)
Alzheimer's disease	1,072 (0.7)	450 (0.8)	37 (0.7)	50 (0.9)	220 (0.8)	13 (0.8)
Non-Alzheimer's dementia	1,099 (0.8)	395 (0.7)	31 (0.6)	34 (0.6)	227 (0.8)	15 (1.0)
Delirium	1,982 (1.4)	823 (1.4)	81 (1.5)	82 (1.4)	406 (1.4)	33 (2.1)
Parkinson's disease	691 (0.5)	249 (0.4)	26 (0.5)	30 (0.5)	134 (0.5)	8 (0.5)
**Pancreas**						
T1 or T2 diabetes	6,531 (4.6)	2,583 (4.6)	244 (4.5)	276 (4.9)	1,327 (4.6)	80 (5.3)

**Infection**						
Covid-19	1,809 (1.2)	719 (1.3)	72 (1.3)	72 (1.3)	383 (1.3)	20 (1.3)
Cholecystitis	1,749 (1.2)	675 (1.2)	62 (1.1)	68 (1.2)	351 (1.2)	29 (1.9)
Pneumonia	6,127 (4.3)	2,406 (4.3)	242 (4.4)	254 (4.5)	1,275 (4.5)	70 (4.6)
Sepsis	10,024 (7.0)	3,837 (6.9)	379 (7.0)	414 (7.4)	2,103 (7.4)	128 (8.5)
LRTI	3,875 (2.7)	1,437 (2.5)	139 (2.5)	143 (2.5)	821 (2.8)	50 (3.3)
URTI	636 (0.4)	277 (0.5)	21 (0.4)	21 (0.4)	142 (0.5)	<5
UTI	9,137 (6.5)	3,490 (6.4)	351 (6.6)	366 (6.7)	1,905 (6.8)	119 (8.0)
SSTI	3,900 (2.7)	1,533 (2.7)	158 (2.9)	152 (2.7)	799 (2.8)	51 (3.3)
**Cardiovascular**						
Arrhythmia	1,654 (1.1)	656 (1.2)	48 (0.9)	67 (1.2)	323 (1.1)	20 (1.3)
Cardiomyopathy	608 (0.4)	230 (0.4)	19 (0.3)	25 (0.4)	130 (0.5)	<5
CHD	7,401 (5.3)	2,875 (5.2)	294 (5.4)	291 (5.2)	1,502 (5.3)	77 (5.1)
Heart failure	3,550 (2.4)	1,430 (2.5)	127 (2.3)	154 (2.7)	753 (2.6)	54 (3.5)
**Mental Health**						
Depression	6,414 (4.8)	2,430 (4.6)	269 (5.2)	278 (5.2)	1,317 (4.9)	57 (4.0)

Incident disease numbers exclude prevalent disease at baseline. Numbers presented are n (%). Abbreviations: CHD, coronary heart disease; T1, type 1; T2, type 2; LRTI, lower respiratory tract infection; URTI, upper respiratory tract infection; UTI, urinary tract infection; SSTI, skin and soft tissue infection. Joint replacement surgery variable includes a diagnosis of hip, knee, ankle, or shoulder replacement. Any brain outcome variable includes a diagnosis of dementia, delirium, or Parkinson’s disease.

Lucas MR, *et al. BMJ Open* 2024; 14:e081926. doi: 10.1136/bmjopen-2023-081926

eTable 12. Hazard ratios of incident disease outcomes in p.C282Y/H63D genotypes in females, excluding a diagnosis of haemochromatosis at baseline

Females	No mutations (Ref group)	H63D+/-		H63D +/+		C282Y+/ H36D +		C282Y+/-		C282Y+/+	
		HR (95% CI)	P	HR (95% CI)	P	HR (95% CI)	P	HR (95% CI)	P	HR (95% CI)	P
Haemochromatosis	1	2.04 (1.31-3.17)	1.70E-03	7.10 (3.75-13.45)	1.80E-09	32.79 (22.10-48.64)	2.10E-67	4.15 (2.68-6.44)	1.80E-10	675.18 (489.89-930.55)	0.00E+00
All-cause mortality	1	0.99 (0.96-1.03)	0.69	0.95 (0.85-1.05)	0.31	1.00 (0.90-1.11)	0.98	1.01 (0.97-1.06)	0.61	1.13 (0.95-1.35)	0.16
**Liver**											
Liver disease (any)	1	1.01 (0.95-1.07)	0.73	1.00 (0.84-1.18)	0.97	1.18 (1.01-1.37)	0.03	1.08 (1.00-1.16)	0.06	1.58 (1.23-2.02)	2.80E-04
Alcoholic liver disease	1	1.10 (0.84-1.43)	0.48	1.57 (0.85-2.88)	0.15	0.79 (0.35-1.78)	0.57	1.54 (1.14-2.06)	0.43	2.75 (1.21-6.20)	0.02
Fibrosis & Cirrhosis	1	0.87 (0.73-1.04)	0.13	0.87 (0.53-1.44)	0.60	1.11 (0.72-1.72)	0.65	0.93 (0.74-1.16)	0.51	2.67 (1.57-4.55)	3.00E-04
Hepatic failure	1	0.77 (0.58-1.03)	0.08	0.92 (0.43-1.95)	0.83	0.50 (0.18-1.34)	0.17	0.85 (0.59-1.22)	0.38	1.76 (0.65-4.74)	0.26
**Cancer**											
Liver cancer	1	1.26 (1.10-1.58)	0.04	1.13 (0.60-2.13)	0.71	0.89 (0.44-1.81)	0.75	1.06 (0.78-1.45)	0.69	1.22 (0.39-3.81)	0.73
**Musculoskeletal**											
Joint replacement surgery (any)	1	0.99 (0.96-1.02)	0.54	0.97 (0.88-1.06)	0.47	1.04 (0.95-1.14)	0.35	0.99 (0.94-1.03)	0.51	1.16 (0.99-1.37)	0.07
Osteoarthritis	1	0.99 (0.94-1.05)	0.72	1.06 (0.91-1.23)	0.46	1.09 (0.95-1.27)	0.22	1.02 (0.95-1.10)	0.59	1.46 (1.15-1.85)	2.10E-03
Fractures (any)	1	1.01 (0.97-1.05)	0.65	0.91 (0.82-1.01)	0.07	1.02 (0.92-1.12)	0.76	1.02 (0.97-1.07)	0.42	1.01 (0.84-1.20)	0.95
Fragility fractures	1	1.02 (0.98-1.07)	0.37	0.87 (0.76-1.00)	0.05	1.10 (0.98-1.24)	0.12	0.99 (0.93-1.05)	0.73	0.86 (0.67-1.10)	0.23
Osteoporosis	1	0.94 (0.90-0.99)	0.01	0.94 (0.83-1.07)	0.34	0.89 (0.78-1.01)	0.08	0.99 (0.93-1.05)	0.73	1.26 (1.03-1.54)	0.02
Rheumatoid arthritis	1	0.96 (0.88-1.04)	0.28	0.94 (0.75-1.17)	0.58	1.00 (0.81-1.25)	0.97	0.89 (0.80-0.99)	0.03	0.96 (0.63-1.44)	0.83
**Brain**											
Any brain outcome (dementia, delirium, or Parkinson's disease)	1	1.02 (0.97-1.09)	0.43	0.93 (0.79-1.11)	0.43	1.03 (0.87-1.20)	0.76	1.00 (0.92-1.08)	0.91	1.23 (0.94-1.61)	0.13
Dementia	1	1.00 (0.92-1.08)	0.94	0.80 (0.63-1.02)	0.07	0.99 (0.80-1.24)	0.95	1.01 (0.91- 1.12)	0.86	1.10 (0.76-1.60)	0.61
Alzheimer's disease	1	1.08 (0.97-1.20)	0.18	0.86 (0.63-1.21)	0.40	1.20 (0.90-1.59)	0.21	1.02 (0.88-1.17)	0.84	1.02 (0.59-1.76)	0.95

Non-Alzheimer's dementia	1	0.93 (0.83-1.04)	0.20	0.72 (0.50-1.03)	0.07	0.81 (0.57-1.14)	0.22	1.03 (0.89-1.18)	0.73	1.17 (0.71-1.96)	0.54
Delirium	1	1.07 (0.98-1.16)	0.13	1.05 (0.84-1.31)	0.67	1.06 (0.85-1.33)	0.58	1.01 (0.91-1.13)	0.79	1.44 (1.02-2.03)	0.04
Parkinson's disease	1	0.93 (0.80-1.07)	0.29	0.97 (0.66-1.44)	0.89	1.15 (0.79-1.65)	0.47	0.98 (0.81-1.18)	0.84	1.04 (0.52-2.10)	0.91
**Pancreas**											
T1 or T2 diabetes	1	1.01 (0.97-1.06)	0.54	0.98 (0.86-1.11)	0.72	1.08 (0.96-1.22)	0.20	1.02 (0.96-1.08)	0.59	1.13 (0.90-1.41)	0.28
**Infection**											
COVID-19	1	1.02 (0.93-1.11)	0.68	1.03 (0.81-1.30)	0.83	1.02 (0.80-1.29)	0.88	1.06 (0.94-1.18)	0.34	1.00 (0.64-1.56)	1.00
Cholecystitis	1	0.98 (0.90-1.08)	0.73	0.92 (0.71-1.19)	0.52	0.98 (0.77-1.24)	0.84	0.99 (0.88-1.11)	0.86	1.49 (1.03-2.16)	0.03
Pneumonia	1	1.00 (0.96-1.05)	0.84	1.02 (0.90-1.17)	0.71	1.05 (0.93-1.20)	0.41	1.03 (0.97-1.10)	0.29	1.02 (0.81-1.29)	0.85
Sepsis	1	0.98 (0.94-1.01)	0.22	0.97 (0.88-1.08)	0.62	1.05 (0.95-1.16)	0.34	1.04 (0.99-1.09)	0.11	1.14 (0.96-1.36)	0.14
LRTI	1	0.95 (0.89-1.01)	0.07	0.92 (0.78-1.09)	0.34	0.93 (0.78-1.09)	0.36	1.04 (0.97-1.12)	0.29	1.14 (0.86-1.51)	0.35
URTI	1	1.12 (0.97-1.29)	0.13	0.86 (0.56-1.33)	0.51	0.84 (0.54-1.30)	0.43	1.12 (0.93-1.34)	0.24	0.29 (0.07-1.17)	0.08
UTI	1	0.97 (0.94-1.01)	0.17	0.99 (0.89-1.10)	0.82	1.02 (0.92-1.13)	0.76	1.03 (0.98-1.08)	0.22	1.17 (0.97-1.40)	0.10
SSTI	1	1.01 (0.95-1.07)	0.81	1.05 (0.90-1.23)	0.54	1.00 (0.85-1.17)	0.98	1.03 (0.95-1.11)	0.52	1.20 (0.91-1.59)	0.19
**Cardiovascular**											
Arrhythmia	1	1.02 (0.93-1.12)	0.69	0.76 (0.57-1.01)	0.06	1.04 (0.82-1.33)	0.73	0.98 (0.87-1.11)	0.78	1.13 (0.72-1.75)	0.60
Cardiomyopathy	1	0.97 (0.83-1.13)	0.71	0.82 (0.52-1.30)	0.41	1.07 (0.72-1.59)	0.75	1.08 (0.90-1.31)	0.40	0.46 (0.15-1.43)	0.18
CHD	1	0.99 (0.95-1.04)	0.81	1.03 (0.92-1.16)	0.60	1.01 (0.89-1.13)	0.92	1.01 (0.96-1.07)	0.70	0.92 (0.74-1.15)	0.48
Heart failure	1	1.03 (0.97-1.10)	0.28	0.92 (0.77-1.10)	0.36	1.12 (0.95-1.32)	0.17	1.06 (0.98-1.15)	0.15	1.35 (1.03-1.77)	0.03
**Mental Health**											
Depression	1	0.97 (0.93-1.02)	0.20	1.10 (0.97-1.23)	0.14	1.11 (0.98-1.25)	0.09	1.03 (0.97-1.09)	0.37	0.83 (0.64-1.08)	0.16

HR (Hazard ratio) compared to those with neither *HFE* mutation. Cox proportional hazards regression models adjusted for age, assessment centre, and genetic principal components 1–10. Abbreviations: CHD, coronary heart disease; T1, type 1; T2, type 2; LRTI, lower respiratory tract infection; URTI, upper respiratory tract infection; UTI, urinary tract infection SSTI, skin and soft tissue infection; CI, confidence interval. Joint replacement surgery variable includes a diagnosis of hip, knee, ankle, or shoulder replacement. Any brain outcome variable includes a diagnosis of dementia, delirium, or Parkinson's disease

eFigure 1. Kaplan-Meier curves for the cumulative incidence of (a) mortality, (b) liver disease, (c) any joint replacement, and (d) any brain outcome, in male *HFE* p.C282Y homozygotes compared to those with no mutations, excluding a diagnosis of haemochromatosis at baseline

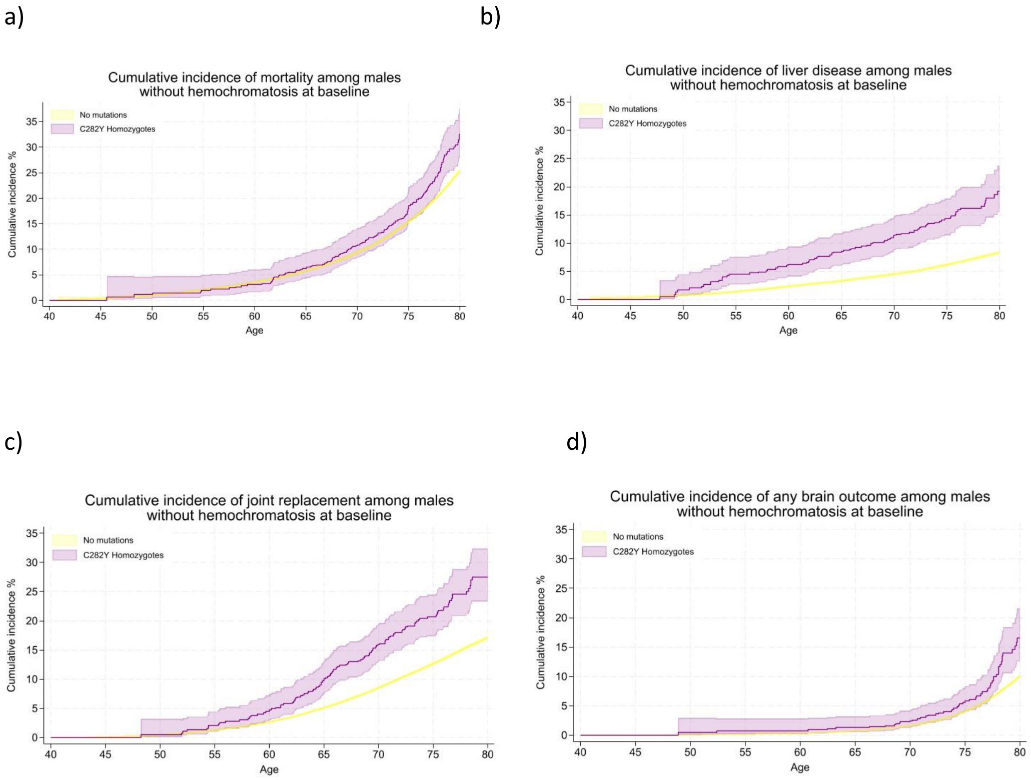

Cumulative incidence (estimated % diagnosed by age 80 years; 95% CIs). Joint replacement surgery includes a diagnosis of hip, knee, ankle, or shoulder replacement. Any brain outcome included a diagnosis of delirium, dementia, or Parkinson's disease.

eFigure 2. Kaplan-Meier curves for the cumulative incidence of (a) liver disease, and (b) any joint replacement, in female *HFE* p.C282Y homozygotes compared to those with no mutations, excluding a diagnosis of haemochromatosis at baseline

a) b)

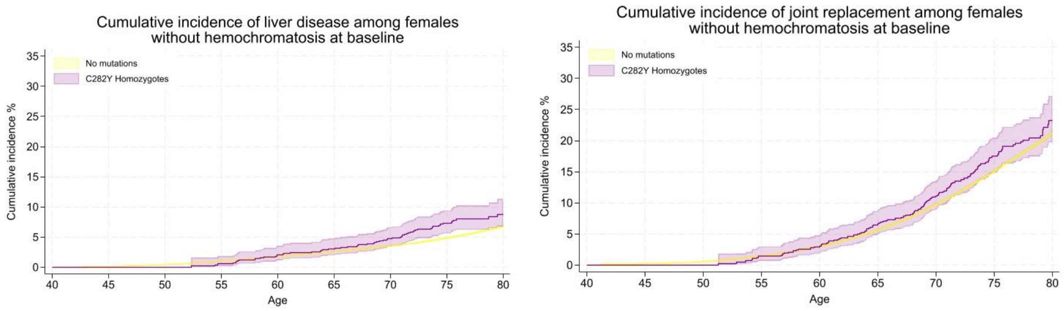

Cumulative incidence (estimated % diagnosed by age 80 years; 95% CIs). Joint replacement surgery includes a diagnosis of hip, knee, ankle, or shoulder replacement.
