# Supplementary material for: HFE genotypes, haemochromatosis diagnosis and clinical outcomes at age 80 years: a prospective cohort study in the UK Biobank
Source: BMJ Open. 2024 Mar 13;14(3):e081926. doi: 10.1136/bmjopen-2023-081926 (PMC10936495; doi:10.1136/bmjopen-2023-081926)
Supplement: Author's manuscript [file bmjopen-2023-081926.draft_revisions.pdf]

BMJ Open is committed to open peer review. As part of this commitment we make the peer review history of every article we publish publicly available.

When an article is published we post the peer reviewers' comments and the authors' responses online. We also post the versions of the paper that were used during peer review. These are the versions that the peer review comments apply to.

The versions of the paper that follow are the versions that were submitted during the peer review process. They are not the versions of record or the final published versions. They should not be cited or distributed as the published version of this manuscript.

BMJ Open is an open access journal and the full, final, typeset and author-corrected version of record of the manuscript is available on our site with no access controls, subscription charges or pay-per-view fees (http://bmjopen.bmj.com).

If you have any questions on BMJ Open's open peer review process please email info.bmjopen@bmj.com

BMJ Open

HFE genotypes, haemochromatosis diagnosis status and clinical penetrance to age 80 in the UK Biobank community cohort

Journal:	*BMJ Open*
Manuscript ID	bmjopen-2023-081926
Article Type:	Original research
Date Submitted by the Author:	09-Nov-2023

Complete List of Authors:	Lucas, Mitchell; University of Exeter<br>Atkins, Janice; University of Exeter<br>Pilling, Luke; University of Exeter, Epidemiology and Public Health<br>Shearman, Jeremy; South Warwickshire University NHS Foundation Trust<br>Melzer, David; University of Exeter

Keywords:	GENETICS, Other metabolic, e.g. iron, porphyria < DIABETES & ENDOCRINOLOGY, Mortality, Hepatology < INTERNAL MEDICINE

SCHOLARONE™
Manuscripts

I, the Submitting Author has the right to grant and does grant on behalf of all authors of the Work (as defined in the below author licence), an exclusive licence and/or a non-exclusive licence for contributions from authors who are: i) UK Crown employees; ii) where BMJ has agreed a CC-BY licence shall apply, and/or iii) in accordance with the terms applicable for US Federal Government officers or employees acting as part of their official duties; on a worldwide, perpetual, irrevocable, royalty-free basis to BMJ Publishing Group Ltd ("BMJ") its licensees and where the relevant Journal is co-owned by BMJ to the co-owners of the Journal, to publish the Work in this journal and any other BMJ products and to exploit all rights, as set out in our licence.

The Submitting Author accepts and understands that any supply made under these terms is made by BMJ to the Submitting Author unless you are acting as an employee on behalf of your employer or a postgraduate student of an affiliated institution which is paying any applicable article publishing charge ("APC") for Open Access articles. Where the Submitting Author wishes to make the Work available on an Open Access basis (and intends to pay the relevant APC), the terms of reuse of such Open Access shall be governed by a Creative Commons licence – details of these licences and which Creative Commons licence will apply to this Work are set out in our licence referred to above.

Other than as permitted in any relevant BMJ Author's Self Archiving Policies, I confirm this Work has not been accepted for publication elsewhere, is not being considered for publication elsewhere and does not duplicate material already published. I confirm all authors consent to publication of this Work and authorise the granting of this licence.

For peer review only

HFE genotypes, haemochromatosis diagnosis status and clinical penetrance to age 80 in the UK Biobank community cohort

Mitchell R Lucas[1]*, Janice L Atkins[1]*, Luke C Pilling[1], Jeremy Shearman[2], David Melzer[1]**

[1]Epidemiology and Public Health Group, Department of Clinical and Biomedical Sciences, Faculty of Health and Life Sciences, University of Exeter, UK

[2]Department of Gastroenterology, South Warwickshire University NHS Foundation Trust, UK

*Contributed equally to this study

Corresponding author: David Melzer, Professor of Epidemiology and Public Health, The Department of Clinical and Biomedical Sciences, Faculty of Health and Life Sciences, University of Exeter, St Luke's Campus, College House, Magdalen Road, Exeter, Devon, EX1 2LU, UK. E-mail: d.melzer@exeter.ac.uk

Keywords: Haemochromatosis; *HFE* p.C282Y / p.H63D genotypes; iron overload; morbidity; mortality; UK Biobank

Word count: 3,853

References: 43

Tables: 1

Figures: 4

For peer review only - http://bmjopen.bmj.com/site/about/guidelines.xhtml

Abstract

Objectives: Type-1 haemochromatosis *HFE* genetic variants have an uncertain clinical penetrance, especially to older ages and in undiagnosed groups. We estimated p.C282Y and p.H63D variant cumulative incidence of multiple clinical outcomes in a large community cohort.

Design: Prospective cohort study.

Setting:22 assessment centres across England, Scotland, and Wales in the UK Biobank (2006-2010).

Participants:451,270 participants genetically similar to the 1000-Genomes European reference population, with a mean 13.3-year follow-up through hospital inpatient, cancer registries and death certificate data.

Main outcome measures: Cox proportional hazard ratios of incident clinical outcomes and mortality in those with *HFE* p.C282Y-p.H63D mutations compared to those with no variants, stratified by sex and adjusted for age, assessment centre and genetic stratification. Cumulative incidences were estimated from age 40 to 80 years.

Results:12.1% of p.C282Y+/+ males had baseline (mean age 57) haemochromatosis diagnoses, with age 80 cumulative incidence of 56.4%. 33.1% died vs. 25.4% without *HFE* variants (Hazard Ratio [HR] 1.29, 95% CI: 1.12-1.48, p=4.7*10^{-4}); 27.9% vs 17.1% had joint replacements, 20.3% vs 8.3% had liver disease, and there was excess delirium, dementia, and Parkinson's disease, but not depression. Associations, including excess mortality, were similar in the group undiagnosed with haemochromatosis. 3.4% of p.C282Y+/+ females had baseline haemochromatosis diagnoses, with cumulative age 80 incidence of 40.5%. There was excess incident liver disease (8.9% vs 6.8%; HR 1.62, 95% CI: 1.27-2.05, p=7.8*10^{-5}), joint replacements and delirium, with similar results in the undiagnosed. p.C282Y/p.H63D and p.H63D+/+ men or women had no statistically significant excess fatigue or depression at baseline and no excess incident outcomes.

Conclusions: Male and female p.C282Y homozygotes experienced greater excess morbidity than previously documented, including those undiagnosed with haemochromatosis in the community. As haemochromatosis diagnosis rates were low at baseline despite treatment being considered effective, trials of screening to identify people with p.C282Y homozygosity early appear justified.

For peer review only - http://bmjopen.bmj.com/site/about/guidelines.xhtml

Strengths and limitations of this study

- We analyzed largescale data on community volunteers from the UK Biobank, one of the world's largest *HFE* genotyped cohorts.
- We have analyzed incident disease outcomes during an extended follow-up period of mean 13.3 years.
- We have provided the first clinical outcome data to age 80 years in those with haemochromatosis genotypes, including those undiagnosed with haemochromatosis at baseline, expanding the life-course evidence on *HFE* penetrance.
- UK Biobank participants were somewhat healthier than the general population, but *HFE* allele frequencies were similar to previous UK studies.
- Incident outcomes were from hospital inpatient and cancer registry follow-up, so did not rely on potentially biased patient self-reporting, but community diagnosed conditions may be underestimated.

For peer review only

For peer review only - http://bmjopen.bmj.com/site/about/guidelines.xhtml

Introduction

Adult-onset haemochromatosis (type 1) is defined by iron overload[1] [2] due to *HFE* gene variants, which dysregulate intestinal iron absorption. The p.C282Y+/+ (homozygous) group have markedly raised iron measures: e.g. median transferrin saturations were over 80% and near 60% in p.C282Y+/+ males and females in HEIRS study, but below 45% in compound heterozygotes (C282Y+/H36D+), and progressively lower across p.H63D+/+ (homozygote), p.C282Y+/- and p.H63D+/- carriers[3]. Women with each genotype have lower mean iron measures than men[4].

Clinical presentation of haemochromatosis is usually with fatigue, joint pain, raised iron measures, from family screening or direct-to-consumer genotyping. Symptoms usually present after age 40 years[6]. In severely affected p.C282Y+/+ patients (>90% of typical cases[7]), liver iron deposition can lead to liver fibrosis, cirrhosis and cancer[8], especially in the presence of other causes of liver disease. Arthropathy[9], diabetes[10], endocrine dysregulation[11], heart arrhythmias and cardiomyopathies[12], and pneumonia[10] have also been reported.

While clinical cohorts frequently have iron overload complications, disease penetrance in population genotyped groups is uncertain, especially at older ages and those not diagnosed with haemochromatosis[3] [13] [14]. Beutler et al found negligible haemochromatosis symptoms in 152 p.C282Y homozygotes from California health appraisal clinics (excluding diagnosed patients)[13] . The HEIRS Study reported excess liver disease in 299 p.C282Y+/+ males[15]. The Melbourne Collaborative Cohort Study[16] reported that 28.4% (95% Confidence Interval [95% CI], 18.8% to 40.2%) of p.C282Y+/+ males (n=95, mean age 65 at follow-up) had 'documented iron-overload-related disease', with 1.2% of p.C282Y+/+ females affected. Similarly, although excess mortality occurred in clinical patients (especially with liver disease)[17][18], no excess mortality was reported in community-identified p.C282Y+/+ males or other *HFE* genotype groups[16] [19]. Using UK Biobank, we previously examined data on European ancestry community participants with mean 7-year follow-up, finding the 1,294 male p.C282Y homozygotes had increased odds of liver disease and osteoarthritis compared to those without p.C282Y or p.H63D variants[10]. A UK Biobank 8.9-year follow-up quantified excess hepatic malignancies in male p.C282Y homozygotes (HR 10.5; 95% CI: 6.6-16.7; p<0.001 versus no *HFE* variant) and excess all-cause mortality (n=88 deaths; HR 1.2; 95% CI: 1.0-1.5; p=0.046)[8].

For p.C282Y/H63D compound heterozygotes, the Melbourne Collaborative Cohort Study[16] found only one male (of 242 studied) with documented iron overload-related disease, although alcohol was also a factor. Similarly, a study of community identified participants with p.H63D variants from the Busselton study (Australia) found none with clinically significant iron overloading[20].

For peer review only - http://bmjopen.bmj.com/site/about/guidelines.xhtml

Given the accumulating evidence of significant clinical penetrance with p.C282Y homozygosity (only) and the reported effectiveness of treatment (predominantly venesection), there is renewed interest in screening for those at high risk[1] [21] [22]. *HFE* p.C282Y homozygosity is recommended for reporting to patients when found incidentally[23].

The UK Biobank community cohort includes baseline questionnaires, plus hospital inpatient diagnoses during the mean 13.3-year follow-up. The cohort therefore provides a potential model for community based genetic screening. Participant consent did not allow genotype feedback (see Methods), so outcomes reflect normal clinical care. Here we aimed to estimate risks and cumulative outcomes to age 80 years by genotype and sex for relevant clinical outcomes, including (for the first time) analyses of those undiagnosed with haemochromatosis at baseline.

Methods

Study population

UK Biobank includes community volunteers aged 39 to 73 years at baseline assessments across England, Scotland and Wales, from 2006 to 2010. Participants were somewhat healthier than the general population[24], but *HFE* allele frequencies were similar to previous UK studies[8]. Data cover 451,270 participants, genetically similar to the 1000 Genomes project European reference population[25], with *HFE* p.C282Y (rs1800562) and *HFE* p.H63D (rs1799945) genotypes from whole exome sequencing (Whole Exome Sequence methods were by Regeneron[26]). Participants gave written informed consent and were informed of relevant health related findings at baseline, but consent excluded individual notification of subsequent findings including genotypes. North West Multi-Centre Research Ethics Committee (Research Ethics Committee reference 11/NW/0382) approved UK Biobank. All research was conducted in accordance with both the Declarations of Helsinki and Istanbul.

Baseline variables and incident health outcomes

Baseline questionnaires covered doctor-diagnosed conditions including haemochromatosis. Symptom questions included: “Over the past two weeks, how often have you felt tired or had little energy?”, and responses were coded as ‘fatigue’ combining “more than half the days” and “nearly every day”. Studied diagnoses were from *a priori* knowledge (see Supplementary eTable 1 for ascertainment codes for 34 outcomes). England, Wales, and Scotland hospital records were available from April 1996 to October 2022. Prevalent diagnoses were from baseline self-report plus hospital inpatient data from 1996 to baseline. Incident diagnoses and surgical procedures were from hospital inpatient data

For peer review only - http://bmjopen.bmj.com/site/about/guidelines.xhtml

(baseline to October 2022) plus cancer registries to December 2020 for England and Wales and November 2021 for Scotland. National death records were available to November 2022. Disease ascertainment used International Classification of Diseases 10th revision (ICD-10) codes. Surgical procedures were from OPCS Classification of Interventions and Procedures version 4 (OPCS-4). Having 'any joint replacement surgery' included hip, knee, ankle, or shoulder replacement surgery. The 'any brain outcome' included delirium, dementia, or Parkinson's disease.

Statistical analysis

Cox proportional hazards regression estimated genotype associations with incident outcomes. Models were stratified by sex and adjusted for age, assessment centre and 10 genetic principal components (accounting for population substructure). Main outcome proportional hazards assumptions (tested using 'estat phtest') were met in models adjusted for 5-year age bands. Given the extensive prior evidence for risks in p.C282Y+/+ groups, multiple testing corrections are discussed for lower risk genotypes, using Bonferroni correction of $p<0.001$ (0.05 / 34 disease outcomes). Kaplan-Meier survivor functions estimated the probabilities of cumulative incidence for associated outcomes from age 40 to 80 years within 5-year bands, by *HFE* genotype and by sex. We applied observed incidence rates in each age group to a notional cohort, estimating hypothetical cumulative incident case numbers from age 40 to 80 years. Sensitivity analysis repeated main analyses excluding participants with a haemochromatosis diagnosis at baseline. All analyses were performed in Stata 17.0.

Patient and public involvement

Patients and participants were and are extensively involved in the UK Biobank study itself. We used anonymised data that were already collected and therefore no patients were involved in developing the research question or the outcomes tested. UK Biobank notified participants of relevant health related findings in the baseline assessment, but there is no individual notification of subsequent findings, including genotypes.

Results

UK Biobank baseline characteristics were previously reported(10) (27) (Table 1, eTables 2-3): 451,270 participants genetically similar to the European 1000 Genomes reference population were followed for a mean 13.3 years. There were 1,298 p.C282Y+/+ (homozygotes), 4,959 p.C282Y/p.H63D compound heterozygote, and 4,673 p.H63D+/+ males: for females 1,604, 5,760 and 5,580 respectively. In the male p.C282Y+/+ group, 12.1% had haemochromatosis diagnoses at, with 6/1,000 diagnosed in p.C282Y/H63D and 2 per 1,000 in the p.H63D+/+ group: the respective rates in females were 3.4%, 2 per 1,000 and 4 per 10,000.

For peer review only - http://bmjopen.bmj.com/site/about/guidelines.xhtml

p.C282Y+/+ males aged 60 plus reported baseline excess fatigue (11.8% vs 8.2%; Odds Ratio (OR): 1.43, 95% CI: 1.11-1.85, p=0.01), but there was no statistically significant excess fatigue with other genotypes, except a marginal association in p.H63D+/- males (OR: 1.06, CI 1.00-1.12, p=0.05) which became non-significant after multiple testing correction. There were no differences in depression prevalence at baseline (Table 1, eTables 2-3).

Males with p.C282Y homozygote genotypes: excess mortality and morbidity versus without HFE variants

Figure 1 shows the Hazard ratios (HRs) for studied incident outcomes by sex and genotype, with cumulative incidence (with confidence intervals) to age 80 years presented in Figure 2 for those outcomes which had significant HRs. p.C282Y+/+ males had increased rates of mortality versus those without p.C282Y or p.H63D variants (HR 1.29, 95% CI: 1.12-1.48, $p=4.7*10^{-4}$; Figure 1; eTables 4 and 5). Cumulative incidence of death was 33.1% (95% CI: 28.9% to 37.8%) versus 25.4% (Figure 2 and 3a). Excess mortality in those undiagnosed with haemochromatosis at UK Biobank baseline (eTables 6 and 7) was similar (HR=1.22 95% CI: 1.05-1.43, $p=1.0*10^{-2}$) with a cumulative death rate of 32.5% (95% CI: 27.9 to 37.6%) (eFigure 1a).

Haemochromatosis diagnosis cumulative incidence at age 80 in p.C282Y+/+ males was 56.4% (Figure 2, eTable 8). Cumulative incidence of 'any liver disease' was 20.3% vs 8.3% without variants (HR 2.56, 95% CI: 2.10-3.12, $p=8.70*10^{-21}$) and 7.7% developed liver fibrosis or cirrhosis vs 1.3%. There was a raised HR for alcoholic liver disease (Figure 1) with cumulative incidence 3.3% of p.C282Y+/+ males vs 1.4%. Liver cancers cumulative incidence was 5.5% (95% CI: 3.8% to 8.0%, vs 0.8% without variants). p.C282Y+/+ males also had raised cumulative incidence of prostate cancer: 17.2% vs 14.8%. The cumulative incidence graphs show excess liver disease clearly apparent by age 55, but the excess mortality becoming significant at older ages (Figure 3a and 3b). Notably, 81.5% of deaths, 66.3% of 'any liver diseases', and 71.8% of joint replacements in p.C282Y+/+ males occurred after age 65.

Joint replacement cumulative incidence in p.C282Y+/+ males was 27.9% vs 17.1% without variants (Figure 3c): this remained after excluding those with fractures within 5 days before surgery (n=53; HR 1.74, 95% CI: 1.45-2.09; $p=1.6*10^{-9}$). p.C282Y+/+ males also had excess osteoarthritis, fragility fractures, and osteoporosis.

Brain outcome (dementia, delirium, or Parkinson's disease) cumulative incidence in p.C282Y+/+ males was 16.3% versus 10.0% without variants (HR 1.65 95% CI: 1.31-2.06, $p=1.70*10^{-5}$) (Figure 3d); for delirium 12.4% vs. 5.8% (HR 1.69, 95% CI: 1.26-2.27; $p=4.80*10^{-4}$); non-Alzheimer's dementia 6.0% vs 3.0% (HR 2.05, 95% CI: 1.40-3.00; $p=2.40*10^{-4}$), and Parkinson's disease (HR 1.86, 95% CI: 1.21-2.87 p=0.005).

For peer review only - http://bmjopen.bmj.com/site/about/guidelines.xhtml

Diabetes (type 1 or 2) cumulative incidence was 23.5% in p.C282Y+/+ males (vs. 19.1%). p.C282Y+/+ males had excess hospital diagnosed COVID-19 (8% vs 4.8%, HR 1.51, 95% CI: 1.08-2.11; p=0.02, missing significance in earlier follow-up data[(28)]), urinary tract infections (UTI) and skin and soft tissue infections (SSTI). Associations were not significant for cardiac outcomes (arrhythmia, cardiomyopathy, CHD, heart failure) or depression.

Estimates of excess morbidity in the male p.C282Y+/+ group without haemochromatosis diagnoses in the community at UK Biobank baseline (n=1,141) were mostly similar to those in the overall sample (Figures 2 and eFigure 1a-d, eTables 6 and 7). Notably, however, point HR estimates for liver fibrosis or cirrhosis, and liver cancers risk appeared marginally lower than in the whole group, but with wide confidence intervals including the whole study estimates HR point estimates (e.g. for liver fibrosis and cirrhosis HR 4.52, 95% CI: 3.07-6.66, $p=2.10*10^{-14}$ without diagnosis, versus HR 5.36 95% CI: 3.83-7.52; $p=2.00*10^{-22}$ in the whole p.C282Y+/+ group). In addition, alcoholic liver disease and osteoporosis lost statistical significance, but cholecystitis became nominally statistically significant (HR 1.54, 95% CI: 1.02-2.32, p=0.04).

Females with p.C282Y homozygote genotypes: excess morbidity versus without HFE variants

Diagnosed haemochromatosis cumulative incidence in p.C282Y+/+ females was 40.5% by age 80. There was no excess mortality, but this group experienced excess 'any liver disease' (8.9% vs 6.75%; HR 1.62, 95% CI: 1.27-2.05; $p=7.80*10^{-5}$) (Figure 4a), liver fibrosis or cirrhosis (1.9% vs 0.8%; HR 2.56, 95% CI: 1.50-4.36; p=0.001), alcoholic liver disease (1.0% vs 0.3%; HR 3.07, 95% CI: 1.44-6.54; p=0.004), plus cholecystitis, versus women without *HFE* variants (Figures 1 and 2; eTables 8, 9 and 10).

Joint replacement surgery was more common in female p.C282Y homozygotes (23.2% vs 21.1%) (Figure 4b), as were osteoarthritis and osteoporosis. Cumulative incidence of brain outcomes was raised (8.6% vs 7.4%; HR 1.30, 95% CI: 1.01-1.68; p=0.04), including delirium (5.9% vs 3.9%; HR 1.50. 95% CI: 1.08-2.08; p=0.02).

There was a nominally significant excess of heart failure (HR 1.34, 95% CI:1.03-1.75, p=0.03) but no associations with other studied cardiac outcomes. There were no associations with liver cancer, fragility fractures, diabetes, dementia, Parkinson's disease, or infections (SSTIs, UTIs).

Estimates of excess morbidity in the female p.C282Y+/+ group without haemochromatosis diagnoses in the community at UK Biobank baseline (n=1,550) were similar to those in the overall sample (Figures 2 and eFigure 2a-b, eTables 11 and 12).

Male and female p.C282Y/p.H63D compound heterozygotes versus without HFE variants

For peer review only - http://bmjopen.bmj.com/site/about/guidelines.xhtml

Haemochromatosis diagnosis cumulative incidence in p.C282Y/H63D males was 5.4% by age 80. However, there was no statistically significant increase in mortality, incidence of 'any liver disease' or liver cancers, joint replacements, or diabetes (Figure 1). p.C282Y/H63D males were modestly more likely to develop SSTIs (HR 1.16, 95% CI: 1.01-1.33, p=0.03) but this lost statistical significance after multiple testing correction.

Haemochromatosis cumulative incidence in p.C282Y/H63D females was 2.7% by age 80 but there were similarly no excess mortality, musculoskeletal diagnoses, diabetes, or brain outcomes (Figure 1). However, there was a modest association with 'any liver disease' (HR 1.19, 95% CI: 1.02-1.38, p=0.03), but associations with specific liver diagnoses were non-significant, including for liver fibrosis and cirrhosis, the most common form of liver disease with iron overload (Figures 1-2; eTables 4, 5, 8, 9, 10).

Males and females with p.H63D homozygote genotypes vs. no HFE variants

Diagnosed haemochromatosis cumulative incidence in p.H63D/H63D males was 1.9% by age 80, but with no excess mortality, liver disease diagnoses, liver cancers, joint replacement surgery, diabetes, or depression. There were protective associations between p.H63D homozygosity and fragility fractures (HR 0.78 95% CI: 0.62-0.98 p=0.03) and osteoporosis (HR 0.73 95% CI: 0.53-0.99, p=0.04) (eTables 4, 5 and 8).

Diagnosed haemochromatosis cumulative incidence p.H63D/H63D females was 0.6% by age 80. There was no excess mortality, liver disease or liver cancers, joint replacement surgery, diabetes, or depression. However, females also had a nominally significant (uncorrected p<0.05) protective association with fragility fractures (HR 0.87 95% CI: 0.76-1.00 p=0.05), echoing the similar protective associations in p.H63D/H63D males (eTables 8, 9 and 10).

Associations for p.C282Y+/- and p.H63D+/- (heterozygote carrier) males and females

There were a small number of nominally significant protective and risk associations in carrier groups (eTables 4 and 5), in p.C282Y+/- males including for excess delirium (HR 1.13 95% CI: 1.04-1.24 p=0.006) and skin and soft tissue infections (HR 1.14 95% CI: 1.06-1.22 p=1.80*10^{-4}) and for p.H63D+/- males a protective association with fragility fractures (HR 0.92 95% CI: 0.86-0.99 p=0.03). For p.C282Y+/- females there were no associations, but for p.H63D+/- females a nominally significant excess of liver cancers (HR 1.26 95% CI: 1.01-1.58 p=0.04) was present, plus a protective association for osteoporosis (eTables 9 and 10). Only the skin and soft tissue infection association was significant after multiple testing correction.

For peer review only - http://bmjopen.bmj.com/site/about/guidelines.xhtml

Discussion

Clinical penetrance by *HFE* genotypes in community samples has been uncertain, especially for those undiagnosed for haemochromatosis, the potential beneficiaries of population screening. Our results show greater excess morbidity and mortality in p.C282Y+/+ groups than previously documented, much occurring at older ages. p.C282Y+/+ males had marked excess mortality (33.1% dead by age 80 vs. 25.4% without p.C282Y or p.H63D variants), plus substantial excess joint replacements, liver disease, brain outcomes and some infections. Estimates of excess morbidity in p.C282Y+/+ groups undiagnosed for haemochromatosis were similar (eTables 7 and 12): perhaps most crucially, the p.C282Y homozygote male undiagnosed group had similar excess mortality, with an estimated cumulative death rate to age 80 of 32.5% (95% CI: 27.9 to 37.6%) versus 25.4% in those without *HFE* variants (eFigure 1a and eTable 7). Of p.C282Y+/+ females (n=1,604), diagnosed haemochromatosis cumulative incidence by 80 was 40.5%, with excess 'any liver disease', joint replacements and delirium present, suggesting more clinical penetrance than previously documented. In p.C282Y/p.H63D and H63D homozygote groups and carriers, we found no excess fatigue or depression at baseline and no excess mortality or major outcomes during follow-up.

Comparison to previous fatigue and depression studies

Baseline characteristics in UK Biobank have been reported before,[27], except for fatigue and depression. Fatigue and depression in haemochromatosis is important to patients and their carers, but the causal role of iron overload in these symptoms is unclear. p.C282Y+/+ males aged 60 plus reported excess fatigue (11.8% versus 8.2% without *HFE* variants) at baseline, but no excess depression at baseline or follow-up. Rates of both conditions were similar in the other studied genotype groups to those without *HFE* variants (except in p.H63D+/- males, with very modest excess fatigue, non-significant with multiple statistical testing correction). A blinded randomized trial of erythrocytapheresis[29] in p.C282Y+/+ groups with moderately raised ferritin levels (300 and 1000 µg/L) did find reduced fatigue scores with transferrin saturations falling from mean 63.5% to 45.4% in the treatment group, although the mean fatigue score was below diagnosis level, making clinical interpretation unclear. Also, recent observational studies have cast doubt on relationships between fatigue, quality of life measures and iron parameters in haemochromatosis patients[30] [31], and fatigue might also occur with venesection. A UK biobank analysis found tiredness genetically linked to factors including increased adiposity, blood lipids and inflammatory markers[32].

Comparison to previous mortality-and morbidity studies

Rates of haemochromatosis diagnosis at baseline (mean age 57) were low even in p.C282Y+/+ groups (12.1% in males, 3.4% in females), but cumulative estimates to age 80 indicated much higher diagnosis rates at older ages. These baseline rates are similar to cumulative rates from the US hospitals genetics

For peer review only - http://bmjopen.bmj.com/site/about/guidelines.xhtml

collaboration eMERGE study[33] for p.C282Y+/+ group at age 60. The eMERGE study[33] figures also show the majority of haemochromatosis diagnoses occurring later in life, reaching nearly 50% in males and 25% in females after age 80.

Earlier reports suggested no excess mortality in community-identified p.C282Y+/+[16] [19], despite higher death rates in clinical cohorts with liver disease[34), (16]. In the UK Biobank much larger sample, we found clearly increased all-cause mortality in p.C282Y+/+ males only (194 p.C282Y+/+ deaths, HR 1.29, 95% CI: 1.12 to 1.48, p=4.70*10^{-4}). A Swedish sample of 2,273 p.C282Y homozygotes found similar increased mortality (HR 1.30, 95% CI: 95%: 1.12-1.50, versus males with no mutations), with no excess in female homozygotes (HR 0.98, 95% CI: 0.82-1.18)[35]. These studies found no excess mortality in p.C282Y/p.H63D or p.H63D/p.H63D groups.

Estimates of disease penetrance in community genotyped groups differ widely [3][13] [14], with the highest estimate from The Melbourne Collaborative Cohort Study[16] , of 28.4% (95% CI: 18.8% to 40.2%) of p.C282Y+/+ males (n=95, mean age 65 at follow-up) having 'documented iron-overload-related disease', and 1.2% of female p.C282Y homozygotes affected. In UK Biobank we found that diagnosed haemochromatosis cumulative incidence was 36.1% in p.C282Y+/+ males and 21.2% of p.C282Y+/+ females by age 65 (eTable 8), suggesting a larger burden of iron overload disease especially in p.C282Y+/+ women. Liver cancers occurred in 5.5% (95% CI: 3.8% to 8.0%) of p.C282Y+/+ males by age 80, a slightly lower estimate than at age 75[8] (7.2% (95% CI: 3.9%-13.1%), but within the earlier confidence intervals. The estimate remains comparable to a meta-analysis[14] estimated lifetime incidence of severe liver disease (cirrhosis or hepatocellular carcinoma) of 9% (95% CI: 2.6%-15.3%) in untreated male *HFE* p.C282Y+/+ homozygotes.

Several studies have reported excess arthritis in p.C282Y homozygote men and to a lesser extent, in female homozygotes[10]. Our results extend a previous 11.5-year follow-up[36], now showing the full extent of later-life joint replacements in male p.C282Y homozygotes and providing a robust measure for severe joint damage in haemochromatosis.

Diabetes was associated with haemochromatosis in historical descriptions. However, we found a modest diabetes excess in p.C282Y+/+ individuals (Figures 1 and 2). Overall, as cumulative diabetes incidence was 23.5% in p.C282Y+/+ males vs. 19.1% without *HFE* variants, non-iron factors appear to now contribute the majority of diabetes even in p.C282Y+/+ males. Although cardiac complications are often noted haemochromatosis reviews[12][37], there is little evidence linking community-identified p.C282Y homozygosity to these outcomes[38] [39]: our finding of excess of heart failure in p.C282Y homozygote women only (HR 1.34 95% CI: 1.03-1.75, p=0.03) needs further study.

For peer review only - http://bmjopen.bmj.com/site/about/guidelines.xhtml

Our previous UK Biobank report on brain outcomes (mean 10.5 years follow-up) found p.C282Y+/+ males had excess dementia diagnoses and iron deposition in key brain areas on MRI(40). The current analysis also found higher incidence especially of non-Alzheimer's dementia in p.C282Y+/+ males plus more delirium. Loughnan et al[41] reported that p.C282Y+/+ males had more Parkinson's disease (OR 1.83; 95% CI: 1.19-2.80; p=0.006) in cross-sectional analyses of UK Biobank: our incident analyses provide a similar estimate for Parkinson's disease in p.C282Y+/+ males (HR 1.86, 95% CI: 1.21-2.87 p=0.005).

In the HEIRS study[3] community genotyped p.C282Y/p.H63D (compound heterozygote) males and females had substantially lower transferrin saturation levels compared to p.C282Y+/+ males or females. In line with this, cumulative incidence of haemochromatosis diagnosed by age 80 was relatively low (men 5.4%, women 2.7%), and no excess mortality, liver fibrosis and cirrhosis, joint replacements, diabetes, or depression in these groups. We did find that p.C282Y/p.H63D females had nominally significant small excess of any liver disease (HR 1.19, 95% CI: 1.02-1.38; p=0.03) although with no excess liver fibrosis and cirrhosis, no similar association in p.C282Y/p.H63D males, and with a non-significant multiple testing p-value. p.C282Y/p.H63D males had a small excess of skin and soft tissue infections (HR 1.16, 95% CI: 1.01-1.33, uncorrected p=0.03), again multiple testing non-significant.

Strengths and limitations

We analyzed largescale cohort data on community volunteers, providing the first outcome data to age 80. *HFE* allele frequencies were similar to other UK studies[8] but UK Biobank did recruit somewhat healthier participants than the general population[24], so we have focused on incident outcomes during the mean 13.3 year follow-up: we found no deviations from proportional hazards assumptions in Cox models. Outcomes for both the overall genotype groups and those undiagnosed with haemochromatosis at UK Biobank baseline are presented, but numbers with haemochromatosis diagnoses at baseline were small and there are no data available on the diverse routes to diagnosis, so modelling treated outcomes would likely be confounded. Most outcomes were from hospital inpatient care, so community diagnosed conditions may be underestimated, but findings are similar to analyses including primary care records (available to 2017 only)[8] and baseline data collected at interview[10].

Implications for early diagnosis and screening

We found greater excess morbidity than previously reported in both p.C282Y+/+ males and females, especially at older ages, and in those undiagnosed with haemochromatosis. Despite treatment (predominantly venesection) being considered effective for preventing liver disease

For peer review only - http://bmjopen.bmj.com/site/about/guidelines.xhtml

progression[1][42][43] rates of haemochromatosis diagnosis were low at baseline: for example, of 31 p.C282Y+/+ males with incident liver cancer, 17 were undiagnosed with haemochromatosis at baseline. Genotyping earlier in life could be a powerful preventive tool to identify p.C282Y+/+ individuals who are clearly at major risk of related excess disease. A recent cross-sectional genotyping study within a US health provider found 72% (144/201) of p.C282Y homozygous patients (mean age 62) had not been diagnosed with haemochromatosis, 36% of whom had iron overload [22]. As cumulative diagnosed haemochromatosis in UK Biobank cohort was estimated 56.4% in p.C282Y+/+ males and 40.5% females by age 80, p.C282Y+/+ genotyping with follow-up iron studies would likely have a high yield. However, the prevention potential following up p.C282Y/p.H63D, H63D+/+ groups will likely be very low, as there was no significant excess fatigue or depression at baseline, and no excess mortality, incident liver fibrosis or cirrhosis, joint replacements, or depression.

Conclusion

Male and female p.C282Y homozygotes in this community cohort experienced greater excess morbidity than previously documented. Much of this excess liver, musculoskeletal, diabetes, brain and morbidity occurred after age 60. In those not diagnosed with haemochromatosis at study baseline, risks were broadly similar to those in the overall group, notably including excess mortality in p.C282Y+/+ males. Trials of targeted or community genotyping to diagnose haemochromatosis earlier appear justified, especially to identify people with the p.C282Y homozygote variants. The potential for iron related preventive treatment in the p.C282Y/H63D, p.H63D+/+ and other *HFE* genotype groups appears very limited, as these genotype groups were associated with no statistically significant excess morbidity or mortality.

Data sharing

Data are available on application to the UK Biobank (www.ukbiobank.ac.uk/register-apply).

Funding

University of Exeter supports ML, LP and DM; JA has a National Institute for Health and Care Research (NIHR) Advanced Fellowship (NIHR301844).

Authors contributions

ML performed the analysis, interpreted results, created the figures, and drafted the manuscript. JA contributed to the design and analysis of the study, data interpretation and drafting of the manuscript. LP contributed to the design of the study, data interpretation, creation of figures and contributed to the manuscript. JS provided expert clinical interpretation of the data and contributed to the

For peer review only - http://bmjopen.bmj.com/site/about/guidelines.xhtml

manuscript. DM oversaw design of the study, data analysis, interpretation of results, and led the writing of the manuscript. All authors approved the final version of the manuscript.

Acknowledgements

This research was conducted using the UK Biobank resource, under application 14631. We thank the UK Biobank participants and coordinators. This work used data provided by patients and collected by the NHS as part of their care and support. Copyright © (2023), NHS England. Re-used with the permission of the NHS England and UK Biobank. All rights reserved. This research also used data assets made available by National Safe Haven as part of the Data and Connectivity National Core Study, led by Health Data Research UK in partnership with the Office for National Statistics and funded by UK Research and Innovation. This study was supported by the National Institute for Health and Care Research Exeter Biomedical Research Centre. The views expressed are those of the authors and not necessarily those of the NIHR or the Department of Health and Social Care.

Competing interests

None declared.

References

1. Adams PC, Jeffrey G, Ryan J. Haemochromatosis. Lancet. 2023;1811–21.
2. Katsarou MS, Papasavva M, Latsi R, Drakoulis N. Hemochromatosis: Hereditary hemochromatosis and HFE gene. Vitam Horm. 2019 Jan 1;110:201–22.
3. Adams PC, Reboussin DM, Barton JC, McLaren CE, Eckfeldt JH, McLaren GD, et al. Hemochromatosis and iron-overload screening in a racially diverse population. N Engl J Med. 2005 Apr 28;352(17):1769–78.
4. Bomford A. Genetics of haemochromatosis. Lancet. 2002 Nov 23;360(9346):1673–81.
5. Steinberg KK, Cogswell ME, Chang JC, Caudill SP, McQuillan GM, Bowman BA, et al. Prevalence of C282Y and H63D mutations in the hemochromatosis (HFE) gene in the United States. JAMA : the journal of the American Medical Association. 2001 May 2;285(17):2216–22.
6. Adams P. National Organization for Rare Disorders. 2019. p. 1–3 Classic Hereditary Hemochromatosis. Available from: https://rarediseases.org/rare-diseases/classic-hereditary-hemochromatosis/
7. Adams PC. Epidemiology and diagnostic testing for hemochromatosis and iron overload. Int J Lab Hematol. 2015;37(S1):25–30.

For peer review only - http://bmjopen.bmj.com/site/about/guidelines.xhtml

8. Atkins JL, Pilling LC, Masoli JAH, Kuo CL, Shearman JD, Adams PC, et al. Association of Hemochromatosis HFE p.C282Y Homozygosity with Hepatic Malignancy. JAMA - Journal of the American Medical Association. 2020 Nov 24;324(20):2048–57.

9. Wijarnpreecha K, Aby ES, Panjawatanan P, Kroner PT, Harnois DM, Palmer WC, et al. Hereditary hemochromatosis and risk of joint replacement surgery: A systematic review and meta-analysis. Eur J Gastroenterol Hepatol. 2020;33(1):96–101.

10. Pilling LC, Tamosauskaite J, Jones G, Wood AR, Jones L, Kuo C ling L, et al. Common conditions associated with hereditary haemochromatosis genetic variants: cohort study in UK Biobank. BMJ. 2019 Jan 16;364:k5222.

11. Pelusi C, Gasparini DI, Bianchi N, Pasquali R. Endocrine dysfunction in hereditary hemochromatosis. Vol. 39, Journal of Endocrinological Investigation. Springer International Publishing; 2016. p. 837–47.

12. Joshi PK, Patel SC, Shreya D, Zamora DI, Patel GS, Rodriguez K, et al. Hereditary Hemochromatosis : A Cardiac Perspective. 2021;13(11).

13. Beutler E, Felitti VJ, Koziol JA, Ho NJ, Gelbart T. Penetrance of 845G → A (C282Y) HFE hereditary haemochromatosis mutation in the USA. Lancet. 2002;359(9302):211–8.

14. Grosse SD, Gurrin LC, Bertalli NA, Allen KJ. Clinical penetrance in hereditary hemochromatosis: estimates of the cumulative incidence of severe liver disease among HFE C282Y homozygotes. Genetics in Medicine. 2017;

15. McLaren CE, Barton JC, Adams PC, Harris EL, Acton RT, Press N, et al. Hemochromatosis and Iron Overload Screening (HEIRS) study design for an evaluation of 100,000 primary care-based adults. Am J Med Sci. 2003 Feb;325(2):53–62.

16. Allen KJ, Gurrin LC, Constantine CC, Osborne NJ, Delatycki MB, Nicoll AJ, et al. Iron-Overload–Related Disease in *HFE* Hereditary Hemochromatosis. New England Journal of Medicine. 2008 Jan 17;358(3):221–30.

17. Niederau C, Fischer R, Purschel A, Purschel P, Stremmel W, Ha¨ussinger D, et al. Long-term Survival in Patients With Hereditary Hemochromatosis. Vol. 110, Gastroenterology. W.B. Saunders; 1996 Apr.

18. Adams PC, Richard L, Weir M, Speechley M. Survival and development of health conditions after iron depletion therapy in C282Y-linked hemochromatosis patients. Canadian Liver Journal. 2021;4(4):381–90.

19. Van Aken MO, De Craen AJM, Gussekloo J, Moghaddam PH, Vandenbroucke JP, Heijmans BT, et al. No increase in mortality and morbidity among carriers of the C282Y mutation of the hereditary haemochromatosis gene in the oldest old: The Leiden 85-plus Study. Eur J Clin Invest. 2002;32(10):750–4.

20. Gochee PA, Powell LW, Cullen DJ, Du Sart D, Rossi E, Olynyk JK. A population-based study of the biochemical and clinical expression of the H63D hemochromatosis mutation. Gastroenterology. 2002 Mar;122(3):646–51.

21. Schmidtke J. Twenty-Five Years of Contemplating Genotype-Based Hereditary Hemochromatosis Population Screening. Genes (Basel). 2022;13(9):1622.

For peer review only - http://bmjopen.bmj.com/site/about/guidelines.xhtml

22. Savatt JM, Johns A, Schwartz MLB, McDonald WS, Salvati ZM, Oritz NM, et al. Testing and Management of Iron Overload After Genetic Screening–Identified Hemochromatosis. JAMA Netw Open. 2023 Oct 23;6(10):e2338995.

23. Miller DT, Lee K, Chung WK, Gordon AS, Herman GE, Klein TE, et al. ACMG SF v3.0 list for reporting of secondary findings in clinical exome and genome sequencing: a policy statement of the American College of Medical Genetics and Genomics (ACMG). Genetics in Medicine. 2021;23(8):1381–90.

24. Fry A, Littlejohns TJ, Sudlow C, Doherty N, Adamska L, Sprosen T, et al. Comparison of Sociodemographic and Health-Related Characteristics of UK Biobank Participants With Those of the General Population. Am J Epidemiol. 2017 Nov 1;186(9):1026–34.

25. Committee on the Use of Race, Ethnicity, and Ancestry as Population Descriptors in Genomics Research, Board on Health Sciences Policy, Committee on Population, Health and Medicine Division, Division of Behavioral and Social Sciences and Education, Nationa [Internet]. National Academies Press, Washington, D.C; 2023. National Academies of Sciences, Engineering, and Medicine,. Available from: https://www.nap.edu/catalog/26902

26. Van Hout C V., Tachmazidou I, Backman JD, Hoffman JD, Liu D, Pandey AK, et al. Exome sequencing and characterization of 49,960 individuals in the UK Biobank. Nature. 2020;586(7831):749–56.

27. Tamosauskaite J, Atkins JL, Pilling LC, Kuo CL, Kuchel GA, Ferrucci L, et al. Hereditary Hemochromatosis Associations with Frailty, Sarcopenia and Chronic Pain: Evidence from 200,975 Older UK Biobank Participants. J Gerontol A Biol Sci Med Sci. 2019 Jan 15;74(3):337–42.

28. Atkins JL, Lucas M, Pilling LC, Melzer D. Letter to the editor regarding: “A haemochromatosis-causing HFE mutation is associated with SARS-CoV-2 susceptibility in the Czech population”. Clinica Chimica Acta 538 (2023) 211–215. Clinica Chimica Acta. 2023;542(February):117271.

29. Ong SY, Gurrin LC, Dolling L, Dixon J, Nicoll AJ, Wolthuizen M, et al. Reduction of body iron in HFE-related haemochromatosis and moderate iron overload (Mi-Iron): a multicentre, participant-blinded, randomised controlled trial. Lancet Haematol. 2017 Dec 1;4(12):e607–14.

30. Acevedo LAU, Alvarenga AM, Fonseca PFS, da Silva NK, Cançado RD, Naoum FA, et al. Quality of Life Scores Remained Different among the Genotypic Groups of Patients with Suspected Hemochromatosis, Even after Treatment Period. Genes (Basel). 2022 Jan 1;13(1).

31. Belhomme N, Morcet J, Lescoat A, Robin F, Deugnier Y, Bardou-Jacquet E, et al. Patient-reported outcomes and their relation with iron parameters in HFE haemochromatosis during maintenance therapy: A prospective cohort study. Liver Int. 2022 Jun 30;

32. Deary V, Hagenaars SP, Harris SE, Hill WD, Davies G, Liewald DCM, et al. Genetic contributions to self-reported tiredness. Mol Psychiatry. 2018 Mar;23(3):609–20.

33. Gallego CJ, Burt A, Sundaresan AS, Ye Z, Shaw C, Crosslin DR, et al. Penetrance of Hemochromatosis in HFE Genotypes Resulting in p.Cys282Tyr and p.[Cys282Tyr];[His63Asp] in the eMERGE Network. Am J Hum Genet. 2015 Oct 1;97(4):512–20.

For peer review only - http://bmjopen.bmj.com/site/about/guidelines.xhtml

34. Adams PC, Speechley M, Kertesz AE. Long-term survival analysis in hereditary hemochromatosis. Gastroenterology. 1991;101(2):368–72.

35. Hagström H, Ndegwa N, Jalmeus M, Ekstedt M, Posserud I, Rorsman F, et al. Morbidity, risk of cancer and mortality in 3645 HFE mutations carriers. Liver Int. 2021 Mar 1;41(3):545–53.

36. Banfield LR, Knapp KM, Pilling LC, Melzer D, Atkins JL. Hemochromatosis Genetic Variants and Musculoskeletal Outcomes: 11.5-Year Follow-Up in the UK Biobank Cohort Study. JBMR Plus. 2023;00(00):1–11.

37. Kowdley K V., Brown KE, Ahn J, Sundaram V. ACG Clinical Guideline. Am J Gastroenterol. 2019;114(August):1.

38. Fox CJ, Cullen DJ, Knuiman MW, Cumpston GN, Divitini ML, Rossi E, et al. Effects of body iron stores and haemochromatosis genotypes on coronary heart disease outcomes in the Busselton health study. J Cardiovasc Risk. 2002 Oct;9(5):287–93.

39. Waalen J, Felitti V, Gelbart T, Ho NJ, Beutler E. Prevalence of coronary heart disease associated with HFE mutations in adults attending a health appraisal center. Am J Med. 2002 Oct;113(6):472–9.

40. Atkins JL, Pilling LC, Heales CJ, Savage S, Kuo CL, Kuchel GA, et al. Hemochromatosis mutations, brain iron imaging, and dementia in the UK Biobank cohort. Journal of Alzheimer's Disease. 2021;79(3):1203–11.

41. Loughnan R, Ahern J, Tompkins C, Palmer CE, Iversen J, Thompson WK, et al. Association of Genetic Variant Linked to Hemochromatosis With Brain Magnetic Resonance Imaging Measures of Iron and Movement Disorders. 2022;74136:1–10.

42. Bardou-Jacquet E, Morandeau E, Anderson GJ, Ramm GA, Ramm LE, Morcet J, et al. Regression of Fibrosis Stage With Treatment Reduces Long-Term Risk of Liver Cancer in Patients With Hemochromatosis Caused by Mutation in HFE. Clin Gastroenterol Hepatol. 2020 Jul;18(8):1851–7.

43. Powell LW, Dixon JL, Ramm GA, Purdie DM, Lincoln DJ, Anderson GJ, et al. Screening for hemochromatosis in asymptomatic subjects with or without a family history. Arch Intern Med. 2006 Feb 13;166(3):294–301.

For peer review only

For peer review only - http://bmjopen.bmj.com/site/about/guidelines.xhtml

Table 1. Baseline characteristics of male and female UK Biobank participants by selected p.C282Y/H63D genotypes

	**No** p.C282Y or p.H63D **variants**	**H63D +/+**	**C282Y +/ H36D +**	**C282Y +/+**
**MALES**				
Total participants	122,841	4,673	4,959	1,298
Mean age, years (SD)	56.99 (8.1)	56.99 (8.1)	56.97 (8.1)	56.84 (8.2)
Hemochromatosis diagnosis, n (%)	29 (0.02)	8 (0.2)	29 (0.6)	157 (12.1)
Self-reported fatigue, n (%)	12,094 (10.1)	451 (9.9)	523 (10.9)	148 (11.8)
Self-reported fatigue (60+ years), n (%),	4,475 (8.2)	173 (8.2)	177 (8.0)	68 (11.8)
Prevalent depression, n (%)	5,883 (4.8)	220 (4.7)	188 (3.8)	71 (5.5)
**FEMALES**				
Total participants	145,694	5,580	5,760	1,604
Mean age, years (SD)	56.62 (7.9)	56.58 (8.1)	56.46 (7.9)	56.92 (8.0)
Hemochromatosis diagnosis, n (%)	8 (0.01)	2 (0.04)	12 (0.2)	54 (3.4)
Self-reported fatigue, n (%)	19,110 (13.5)	760 (14.1)	785 (14.1)	220 (14.4)
Self-reported fatigue (60+ years), n (%),	6,055 (10.0)	253 (10.9)	229 (9.9)	77 (11.4)
Prevalent depression, n (%)	10,734 (7.4)	387 (6.9)	432 (7.5)	123 (7.7)

A total of 451,270 male and female participants genetically-similar to the 1000 Genomes project European reference population with *HFE* genotypic data available in the UK Biobank. Numbers presented are mean (SD) for continuous variables and n (%) for categorical variables. Fatigue = tiredness in more than half the days in past 2 weeks. See supplementary eTable 2 and 3 for all p.C282Y/H63D genotype groups.

For peer review only

Figure 1. Hazard ratios of incident disease outcomes (95% CI) in selected p.C282Y/H63D genotypes compared to those with no mutations

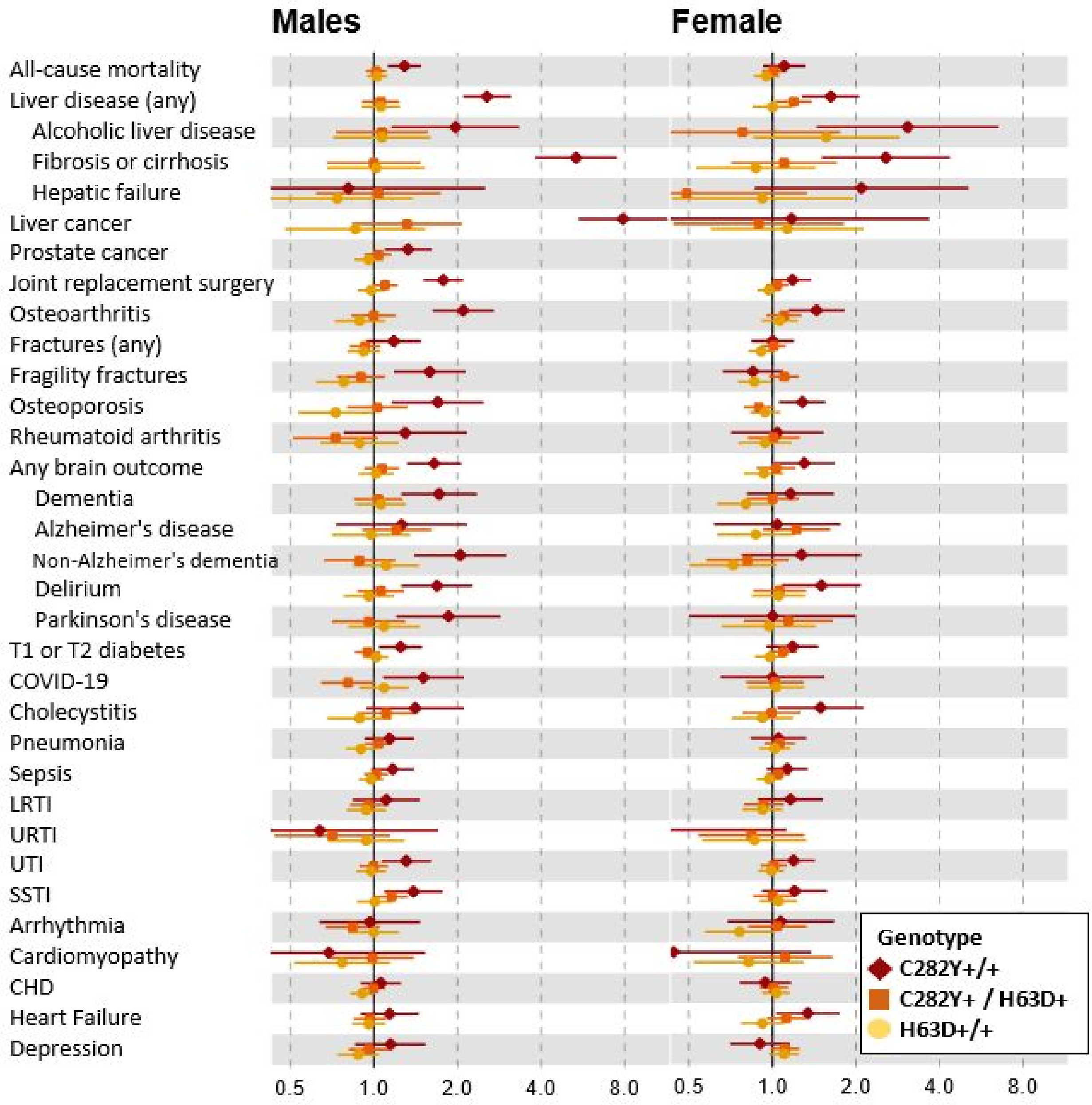

Hazard ratios compared to those with neither *HFE* mutation. Cox proportional hazards regression models adjusted for age, assessment centre, and genetic principal components 1–10. Abbreviations: UTI, urinary tract infection; SSTI, skin and soft tissue infection; CI, confidence interval. Joint replacement surgery includes a diagnosis of hip, knee, ankle, or shoulder replacement. Any brain outcome included a diagnosis of delirium, dementia, or Parkinson's disease. See supplementary eTables 4, 5, 9 and 10 for incident numbers and HRs for all p.C282Y/H63D genotype groups.

For peer review only - http://bmjopen.bmj.com/site/about/guidelines.xhtml

Figure 2. Cumulative incidence of outcomes from ages 40-80 years by *HFE* genotypes (no p.C282Y or p.H63D variants vs p.C282Y homozygotes and p.C282Y homozygotes undiagnosed with hemochromatosis at baseline)

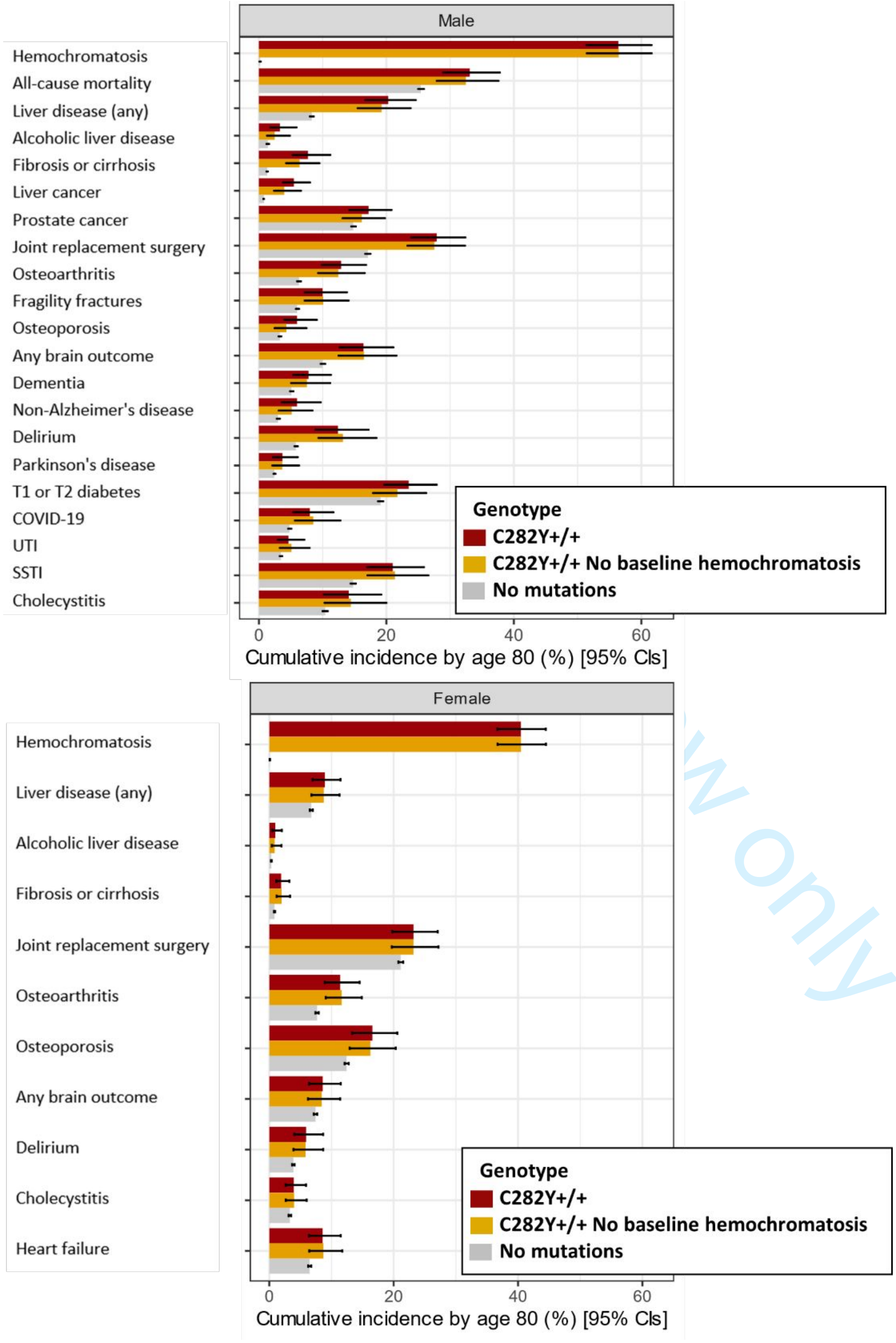

For peer review only - http://bmjopen.bmj.com/site/about/guidelines.xhtml

Cumulative incidence (estimated % diagnosed by age 80 years; 95% CIs) for significant outcomes ($p<0.05$) from Cox proportional hazards regression models (Figure 1; eTables 5, 7, 10 and 12). Abbreviations: UTI, urinary tract infection; SSTI, skin and soft tissue infection; CI, confidence interval. Joint replacement surgery includes a diagnosis of hip, knee, ankle, or shoulder replacement. Any brain outcome included a diagnosis of delirium, dementia, or Parkinson's disease.

For peer review only

Figure 3. Kaplan-Meier curves for the cumulative incidence of (a) mortality, (b) liver disease, (c) any joint replacement, and (d) any brain outcome, in male *HFE* p.C282Y homozygotes compared to those with no mutations

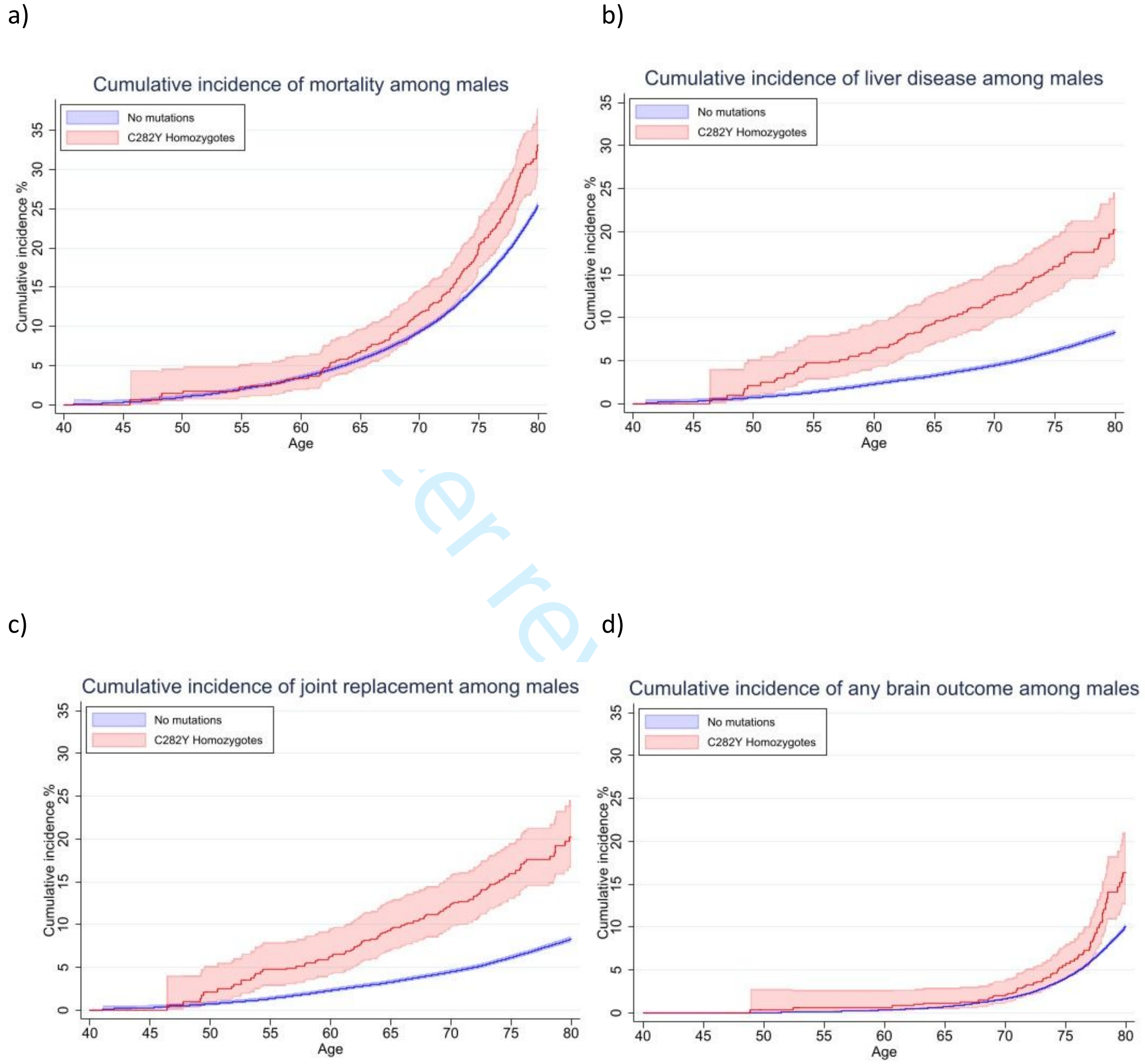

Cumulative incidence (estimated % diagnosed by age 80 years; 95% CIs). Joint replacement surgery includes a diagnosis of hip, knee, ankle, or shoulder replacement. Any brain outcome included a diagnosis of delirium, dementia, or Parkinson's disease.

For peer review only - http://bmjopen.bmj.com/site/about/guidelines.xhtml

Figure 4. Kaplan-Meier curves for the cumulative incidence of (a) liver disease, and (b) any joint replacement, in female *HFE* p.C282Y homozygotes compared to those with no mutations

a) b)

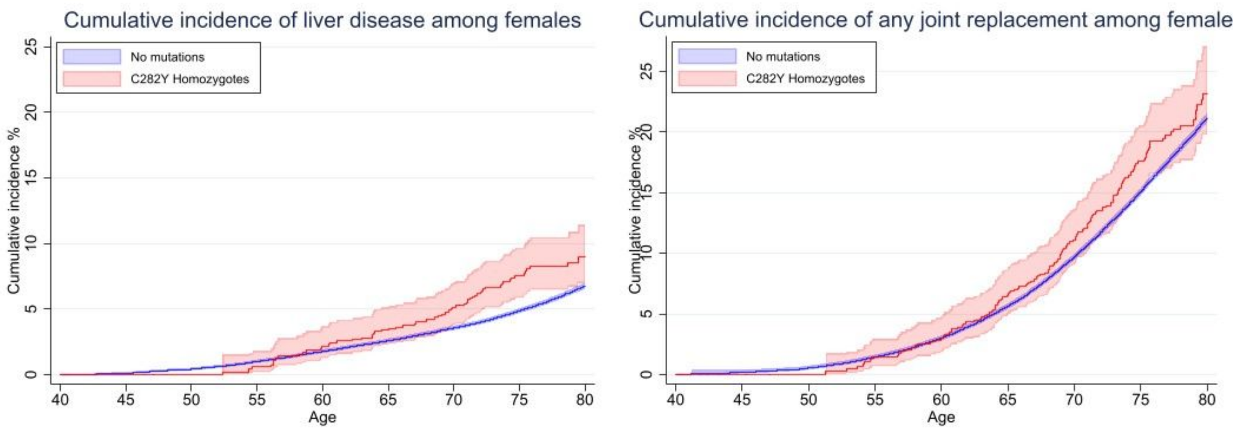

Cumulative incidence (estimated % diagnosed by age 80 years; 95% CIs). Joint replacement surgery includes a diagnosis of hip, knee, ankle, or shoulder replacement.

For peer review only - http://bmjopen.bmj.com/site/about/guidelines.xhtml

Supplementary Material

eTable 1. Incident hospital diagnosed outcomes or procedures with associated ICD-10/OPCS codes

Disease	ICD-10 code
Arrhythmia	I49
Cardiomyopathy	I42
CHD	I20; I21; I22; I23; I24; I25
Cholecystitis	K800; K804; K81
COVID-19	U07.1; U07.2
Delirium	F05
Dementia	F00; F01; F02; F03; G30
Alzheimer's disease	G30
Non-Alzheimer's dementia	F00; F01; F02; F03
Depression	F32; F33; F34.1
Fractures (any)	S02; S12; S22; S32; S42; S52; S62; S72; S82; S92; T02; T08; T10; T12; T14.2
Fragility fractures	S220; S32; S325; S328; S422; S423; S424; S524; S525; S720; S721; S722; S582; S5823; T08
Hemochromatosis	E83.1
Heart failure	I50; J81
Liver disease (any)	K70; K71; K72; K73; K74; K75; K76; K77
Alcoholic liver disease	K70
Fibrosis & Cirrhosis	K74
Hepatic failure	K72
Liver cancer	C22
Lower respiratory tract infection	J20; J21; J22
Osteoarthritis	M15.0; M15.1; M15.2; M15.9; M16.0; M16.1; M17.0; M17.1; M18.0; M18.1; M19.0
Osteoporosis	M80; M81; M811; M812; M813; M814; M815; M816; M818; M819
Parkinson's disease	G20; F02.3
Pneumonia	J13; J14; J15; J16; J17; J18
Prostate cancer	C61
Rheumatoid arthritis	M05; M06
Sepsis	A021; A039; A207; A241; A217; A227; A239; A267; A282; A327; A392; A393; A394; A40; A41; A427; A548; B007; B377; H440; J950; N390; O85; P36; R651; T814; T880
Skin soft tissue infection	L00; L01; L02; L03; L04; L05; L06; L07; L08
Type 1 or Type 2 diabetes	E10; E11
Urinary tract infection	N30; N34; N39
Upper respiratory tract infection	J39; J06; J04

Procedure	OPCS Code
Ankle replacement	O32; O320; O321; O322; O323; O324; O325
Hip replacement	W37;W370;W371;W372;W373;W374;W38;W380;W381;W382;W383;W384; W46;W460;W461;W462;W463;W47;W470;W471;W472;W473;W93;W930; W931;W932;W933;W94;W940;W941;W942;W943;O171;O172;O173;W580; W581;W582
Knee replacement	O18*; W40*; W41*; W42*
Shoulder replacement	O06; +A11O060; O061; O062; O063; O068; O069; O07; O070; O071; O072; O073; O078; O079; O08; O080; O081; O082; O083; O084; O088; O089; O09; O091; O098; O099; O10; O101; O108; O109

ICD-10 = International Classification of Diseases 10th revision codes; OPCS-4 = OPCS Classification of Interventions and Procedures version 4. Joint replacement surgery variable includes a diagnosis of hip, knee, ankle, or shoulder replacement. Any brain outcome variable includes a diagnosis of dementia, delirium, or Parkinson’s disease.

For peer review only

For peer review only - http://bmjopen.bmj.com/site/about/guidelines.xhtml

eTable 2. Baseline characteristics of male UK Biobank participants by p.C282Y/H63D genotypes

	No mutations	H63D+/-	H63D+/+	C282Y+/H63+	C282Y+/-	C282Y +/+	Total
**Total participants**	122,841	47,983	4,673	4,959	24,636	1,298	206,390
Mean age, years (SD)	56.99 (8.1)	57.02 (8.1)	56.99 (8.1)	56.97 (8.1)	57.02 (8.1)	56.84 (8.2)	57.00 (8.1)
Hemochromatosis diagnosis, n (%)	29 (0.02)	17 (0.04)	8 (0.2)	29 (0.6)	27 (0.1)	157 (12.1)	267 (0.1)
Self-reported fatigue, n (%)	12,094 (10.1)	4,865 (10.4)	451 (9.9)	523 (10.9)	2,476 (10.3)	148 (11.8)	20,557 (10.2)
Self-reported fatigue (60+ years), n (%),	4,475 (8.2)	1,858 (8.6)	173 (8.2)	177 (8.0)	924 (8.4)	68 (11.8)	7,675 (8.3)
Depression diagnosis, n (%)	5,883 (4.8)	2,232 (4.7)	220 (4.7)	188 (3.8)	1,146 (4.7)	71 (5.5)	9,740 (4.7)

A total of 206,390 male participants genetically similar to the 1000 Genomes project European reference population with *HFE* genotypic data available in the UK Biobank. Numbers presented are mean (SD) for continuous variables and n (%) for categorical variables. Fatigue = tiredness in more than half the days in past 2 weeks.

For peer review only - http://bmjopen.bmj.com/site/about/guidelines.xhtml

eTable 3. Baseline characteristics of female UK Biobank participants by p.C282Y/H63D genotypes

	No mutations	H63D+/-	H63D+/+	C282Y+/H63+	C282Y+/-	C282Y +/+	Total
**Total participants**	145,694	57,021	5,580	5,760	29,221	1,604	244,880
Mean age, years (SD)	56.62 (7.9)	56.58 (7.9)	56.58 (8.1)	56.46 (7.9)	56.49 (8.0)	56.92 (8.0)	56.60 (7.9)
Hemochromatosis diagnosis, n (%)	8 (0.01)	6 (0.01)	2 (0.04)	12 (0.2)	5 (0.02)	54 (3.4)	87 (0.04)
Self-reported fatigue, n (%)	19,110 (13.5)	7,449 (13.5)	760 (14.1)	785 (14.1)	3,802 (13.4)	220 (14.4)	32,126 (13.5)
Self-reported fatigue (60+ years), n (%),	6,055 (10.0)	2,417 (10.2)	253 (10.9)	229 (9.9)	1,157 (9.6)	77 (11.4)	10,188 (10.0)
Depression diagnosis, n (%)	10,734 (7.4)	4,266 (7.5)	387 (6.9)	432 (7.5)	2,271 (7.8)	123 (7.7)	18,213 (7.4)

A total of 244,880 female participants genetically similar to the 1000 Genomes project European reference population with *HFE* genotypic data available in the UK Biobank. Numbers presented are mean (SD) for continuous variables and n (%) for categorical variables. Fatigue = tiredness in more than half the days in past 2 weeks.

For peer review only - http://bmjopen.bmj.com/site/about/guidelines.xhtml

eTable 4. Incident hospital diagnoses in male UK Biobank participants by p.C282Y/H63D genotypes

Males	No mutations	H63D +/-	H63D +/+	C282Y +/ H36D +	C282Y +/-	C282Y +/+
Hemochromatosis	85 (0.1)	51 (0.1)	29 (0.6)	95 (1.9)	42 (0.2)	288 (25.2)
All-cause mortality	13,976 (11.4)	5,658 (11.8)	540 (11.6)	591 (11.9)	2,996 (12.2)	194 (15.0)
**Liver**						
Liver disease (any)	3,969 (3.3)	1,621 (3.4)	160 (3.5)	171 (3.5)	803 (3.3)	102 (8.1)
Alcoholic liver disease	608 (0.5)	227 (0.5)	25 (0.5)	28 (0.6)	150 (0.6)	14 (1.1)
Fibrosis & Cirrhosis	618 (0.5)	252 (0.5)	24 (0.5)	26 (0.5)	142 (0.6)	36 (2.8)
Hepatic failure	355 (0.3)	137 (0.3)	10 (0.2)	15 (0.3)	54 (0.2)	3 (0.2)
**Cancer**						
Liver cancer	363 (0.3)	167 (0.4)	12 (0.3)	20 (0.4)	80 (0.3)	31 (2.4)
Prostate cancer	7,723 (6.4)	3,025 (6.4)	283 (6.2)	319 (6.5)	1,613 (6.7)	104 (8.1)
**Musculoskeletal**						
Joint replacement surgery (any)	8,429 (7.1)	3,379 (7.3)	315 (7.0)	376 (7.8)	1,801 (7.5)	144 (11.8)
Osteoarthritis	2,873 (2.5)	1,155 (2.6)	97 (2.2)	120 (2.6)	601 (2.6)	61 (5.5)
Fractures (any)	6,092 (5.2)	2,298 (5.1)	212 (4.8)	231 (4.9)	1,243 (5.3)	75 (6.2)
Fragility fractures	2,666 (2.2)	963 (2.0)	79 (1.7)	98 (2.0)	551 (2.3)	44 (3.4)
Osteoporosis	1,522 (1.3)	602 (1.3)	42 (0.9)	64 (1.3)	319 (1.3)	27 (2.1)
Rheumatoid arthritis	1,131 (0.9)	479 (1.0)	38 (0.8)	33 (0.7)	238 (1.0)	15 (1.2)
**Brain**						
Any brain outcome (dementia, delirium, or Parkinson's disease)	4,619 (3.8)	1,852 (3.9)	177 (3.8)	201 (4.1)	993 (4.0)	76 (5.9)
Dementia	2,306 (1.9)	916 (1.9)	92 (2.0)	97 (2.0)	487 (2.0)	40 (3.1)
Alzheimer's disease	1,026 (0.8)	380 (0.8)	38 (0.8)	50 (1.0)	189 (0.8)	13 (1.0)
Non-Alzheimer's dementia	1,299 (1.1)	540 (1.1)	54 (1.2)	47 (1.0)	303 (1.2)	27 (2.1)
Delirium	2,621 (2.1)	1,028 (2.1)	95 (2.0)	112 (2.3)	603 (2.5)	45 (3.5)
Parkinson's disease	1,116 (0.9)	454 (1.0)	46 (1.0)	43 (0.9)	202 (0.8)	21 (1.6)
**Pancreas**						
T1 or T2 diabetes	9,515 (8.0)	3,665 (7.9)	368 (8.1)	367 (7.6)	1,822 (7.7)	119 (9.6)
**Infection**						
Covid-19	2,332 (1.9)	956 (2.0)	96 (2.1)	77 (1.6)	522 (2.1)	35 (2.7)
Cholecystitis	1,624 (1.3)	613 (1.3)	55 (1.2)	73 (1.5)	318 (1.3)	24 (1.9)

For peer review only - http://bmjopen.bmj.com/site/about/guidelines.xhtml

Pneumonia	8,019 (6.7)	3,128 (6.7)	273 (6.0)	342 (7.1)	1,651 (6.9)	95 (7.6)
Sepsis	10,081 (8.4)	3,955 (8.4)	374 (8.2)	416 (8.6)	2,065 (8.6)	122 (9.7)
LRTI	4,113 (3.4)	1,694 (3.6)	148 (3.2)	164 (3.3)	914 (3.7)	50 (3.9)
URTI	588 (0.5)	226 (0.5)	21 (0.5)	17 (0.3)	109 (0.4)	4 (0.3)
UTI	7,099 (5.9)	2,773 (5.9)	263 (5.7)	290 (6.0)	1,472 (6.1)	96 (7.6)
SSTI	4,604 (3.8)	1,792 (3.8)	176 (3.8)	216 (4.5)	1,053 (4.4)	66 (5.2)
**Cardiovascular**						
Arrhythmia	2,283 (1.9)	871 (1.8)	86 (1.9)	77 (1.6)	445 (1.8)	23 (1.8)
Cardiomyopathy	855 (0.7)	330 (0.7)	25 (0.5)	34 (0.7)	179 (0.7)	6 (0.5)
CHD	11,796 (10.5)	4,626 (10.5)	412 (9.6)	485 (10.5)	2,422 (10.7)	133 (11.0)
Heart failure	5,779 (4.7)	2,289 (4.8)	211 (4.6)	229 (4.7)	1,256 (5.1)	68 (5.3)
**Mental Health**						
Depression	3,929 (3.4)	1,615 (3.5)	132 (3.0)	152 (3.2)	772 (3.3)	46 (3.8)

Incident disease numbers exclude prevalent disease at baseline. Numbers presented are n (%). Abbreviations: CHD, coronary heart disease; T1, type 1; T2, type 2; LRTI, lower respiratory tract infection; URTI, upper respiratory tract infection; UTI, urinary tract infection; SSTI, skin and soft tissue infection. Joint replacement surgery variable includes a diagnosis of hip, knee, ankle, or shoulder replacement. Any brain outcome variable includes a diagnosis of dementia, delirium, or Parkinson's disease.

For peer review only - http://bmjopen.bmj.com/site/about/guidelines.xhtml

eTable 5. Hazard ratios of incident disease outcomes in p.C282Y/H63D genotypes in males

**Males**	**No mutations**	**H63D +/-**		**H63D +/+**		**C282Y +/ H36D +**		**C282Y +/-**		**C282Y +/+**	
		HR (95% CI)	P	HR (95% CI)	P	HR (95% CI)	P	HR (95% CI)	P	HR (95% CI)	P
Hemochromatosis	1	1.53 (1.08-2.17)	0.02	8.95 (5.87-13.64)	$2.20*10^{-24}$	27.47 (20.49-36.84)	$1.00*10^{-108}$	2.40 (1.66-3.47)	$3.70*10^{-06}$	405.29 (317.06-518.11)	$2.97*10^{-501}$
All-cause mortality	1	1.03 (1.00-1.06)	0.05	1.01 (0.93-1.11)	0.75	1.02 (0.94-1.11)	0.58	1.05 (1.00-1.09)	0.03	1.29 (1.12-1.48)	$4.70*10^{-04}$
**Liver**											
Liver disease (any)	1	1.05 (0.99-1.11)	0.12	1.06 (0.90-1.24)	0.48	1.06 (0.91-1.23)	0.46	1.00 (0.93-1.78)	0.96	2.56 (2.10-3.12)	$8.70*10^{-21}$
Alcoholic liver disease	1	0.95 (0.81-1.11)	0.50	1.07 (0.71-1.59)	0.76	1.07 (0.73-1.57)	0.72	1.17 (0.98-1.40)	0.09	1.97 (1.16-3.35)	0.01
Fibrosis & Cirrhosis	1	1.04 (0.90-1.21)	0.58	1.02 (0.68-1.53)	0.93	1.00 (0.68-1.48)	0.99	1.11 (0.92-1.33)	0.28	5.36 (3.83-7.52)	$2.00*10^{-22}$
Hepatic failure	1	0.99 (0.81-1.21)	0.92	0.74 (0.40-1.40)	0.36	1.04 (0.62-1.74)	0.88	0.75 (0.56-1.00)	0.05	0.81 (0.26-2.52)	0.72
**Cancer**											
Liver cancer	1	1.17 (0.98-1.41)	0.09	0.86 (0.48-1.53)	0.61	1.32 (0.84-2.08)	0.22	1.07 (0.84-1.36)	0.61	7.90 (5.46-11.43)	$5.50*10^{-28}$
Prostate cancer	1	1.00 (0.96-1.05)	0.86	0.96 (0.86-1.09)	0.56	1.03 (0.93-1.16)	0.55	1.05 (1.00-1.11)	0.09	1.33 (1.09-1.61)	0.004
**Musculoskeletal**											
Joint replacement surgery (any)	1	1.03 (0.99-1.07)	0.21	0.98 (0.88-1.10)	0.74	1.10 (1.00-1.22)	0.08	1.06 (1.01-1.12)	0.02	1.78 (1.51-2.10)	$6.40*10^{-12}$
Osteoarthritis	1	1.03 (0.96-1.10)	0.44	0.89 (0.72-1.09)	0.25	1.01 (0.84-1.21)	0.99	1.03 (0.94-1.12)	0.55	2.10 (1.63-2.71)	$1.10*10^{-08}$
Fractures (any)	1	0.96 (0.92-1.01)	0.13	0.92 (0.80-1.05)	0.23	0.93 (0.82-1.06)	0.29	1.01 (0.95-1.07)	0.85	1.18 (0.94-1.48)	0.16
Fragility fractures	1	0.92 (0.86-0.99)	0.03	0.78 (0.62-0.98)	0.03	0.90 (0.74-1.10)	0.31	1.02 (0.93-1.12)	0.68	1.59 (1.18-2.14)	0.002
Osteoporosis	1	1.01 (0.92-1.11)	0.85	0.73 (0.53-0.99)	0.04	1.03 (0.80-1.32)	0.82	1.02 (0.91-1.16)	0.70	1.70 (1.16-2.48)	0.007
Rheumatoid arthritis	1	1.08 (0.97-1.21)	0.13	0.89 (0.64-1.23)	0.48	0.73 (0.51-1.03)	0.07	1.05 (0.91-1.20)	0.53	1.30 (0.78-2.16)	0.31
**Brain**											
Any brain outcome (dementia, delirium, or Parkinson's disease)	1	1.02 (0.97-1.08)	0.39	1.02 (0.88-1.18)	0.83	1.07 (0.93-1.24)	0.32	1.06 (0.99-1.13)	0.11	1.65 (1.31-2.06)	$1.70*10^{-05}$
Dementia	1	1.01 (0.94-1.09)	0.76	1.06 (0.86-1.31)	0.58	1.04 (0.85-1.27)	0.73	1.04 (0.94-1.14)	0.48	1.72 (1.26-2.35)	0.001
Alzheimer's disease	1	0.94 (0.84-1.06)	0.32	0.98 (0.71-1.36)	0.91	1.21 (0.91-1.60)	0.19	0.90 (0.77-1.05)	0.20	1.26 (0.73-2.17)	0.42
Non-Alzheimer's dementia	1	1.06 (0.96-1.17)	0.25	1.11 (0.84-1.45)	0.46	0.89 (0.66-1.19)	0.43	1.14 (1.01-1.30)	0.04	2.05 (1.40-3.00)	$2.40*10^{-04}$
Delirium	1	1.00 (0.93-1.08)	0.98	0.96 (0.78-1.18)	0.69	1.06 (0.87-1.28)	0.57	1.13 (1.04-1.24)	0.006	1.69 (1.26-2.27)	$4.80*10^{-04}$
Parkinson's disease	1	1.04 (0.93-1.16)	0.47	1.09 (0.81-1.47)	0.55	0.96 (0.71-1.30)	0.77	0.90 (0.77-1.04)	0.16	1.86 (1.21-2.87)	0.005

For peer review only - http://bmjopen.bmj.com/site/about/guidelines.xhtml

**Pancreas**											
T1 or T2 diabetes	1	0.98 (0.95-1.02)	0.44	1.02 (0.92-1.13)	0.69	0.95 (0.86-1.06)	0.35	0.95 (0.91-1.00)	0.06	1.25 (1.05-1.50)	0.01
**Infection**											
COVID-19	1	1.05 (0.97-1.13)	0.21	1.09 (0.89-1.34)	0.4	0.81 (0.65-1.02)	0.07	1.11 (1.01-1.22)	0.03	1.51 (1.08-2.11)	0.02
Cholecystitis	1	0.96 (0.88-1.06)	0.45	0.89 (0.68-1.16)	0.39	1.11 (0.87-1.40)	0.4	0.96 (0.86-1.09)	0.56	1.41 (0.94-2.11)	0.09
Pneumonia	1	1.00 (0.96-1.04)	0.82	0.90 (0.80-1.02)	0.09	1.04 (0.93-1.16)	0.51	1.01 (0.96-1.07)	0.68	1.14 (0.93-1.40)	0.21
Sepsis	1	1.01 (0.97-1.04)	0.78	0.98 (0.88-1.08)	0.66	1.02 (0.92-1.12)	0.7	1.01 (0.96-1.06)	0.70	1.17 (0.98-1.40)	0.09
LRTI	1	1.05 (0.99-1.11)	0.10	0.94 (0.80-1.11)	0.46	0.96 (0.82-1.12)	0.58	1.08 (1.00-1.16)	0.48	1.11 (0.84-1.46)	0.48
URTI	1	0.99 (0.84-1.15)	0.85	0.94 (0.61-1.45)	0.77	0.71 (0.44-1.15)	0.16	0.92 (0.75-1.13)	0.41	0.64 (0.24-1.71)	0.38
UTI	1	1.00 (0.95-1.04)	0.90	0.98 (0.86-1.10)	0.69	1.00 (0.89-1.13)	0.98	1.02 (0.96-1.08)	0.51	1.31 (1.07-1.61)	0.008
SSTI	1	1.00 (0.94-1.05)	0.88	1.01 (0.87-1.17)	0.90	1.16 (1.01-1.33)	0.03	1.14 (1.06-1.22)	$1.80*10^{-04}$	1.39 (1.09-1.77)	0.008
**Cardiovascular**											
Arrhythmia	1	0.98 (0.90-1.06)	0.57	1.00 (0.81-1.24)	0.99	0.84 (0.67-1.05)	0.13	0.96 (0.87-1.07)	0.49	0.97 (0.64-1.47)	0.89
Cardiomyopathy	1	0.99 (0.87-1.12)	0.88	0.77 (0.52-1.15)	0.21	0.99 (0.70-1.39)	0.94	1.04 (0.89-1.23)	0.60	0.69 (0.31-1.53)	0.36
CHD	1	1.01 (0.97-1.04)	0.77	0.91 (0.83-1.01)	0.08	1.00 (0.91-1.10)	0.99	1.02 (0.97-1.06)	0.45	1.06 (0.90-1.26)	0.49
Heart failure	1	1.01 (0.96-1.06)	0.67	0.96 (0.84-1.10)	0.59	0.97 (0.85-1.11)	0.65	1.07 (1.01-1.14)	0.03	1.14 (0.89-1.44)	0.29
**Mental Health**											
Depression	1	1.05 (0.99-1.12)	0.07	0.88 (0.74-1.05)	0.16	0.96 (0.81-1.13)	0.60	0.98 (0.91-1.06)	0.59	1.15 (0.86-1.54)	0.34

HR (Hazard ratio) compared to those with neither *HFE* mutation. Cox proportional hazards regression models adjusted for age, assessment centre, and genetic principal components 1–10. Abbreviations: CHD, coronary heart disease; T1, type 1; T2, type 2; LRTI, lower respiratory tract infection; URTI, upper respiratory tract infection; UTI, urinary tract infection; SSTI, skin and soft tissue infection; CI, confidence interval. Joint replacement surgery variable includes a diagnosis of hip, knee, ankle, or shoulder replacement. Any brain outcome variable includes a diagnosis of dementia, delirium, or Parkinson's disease.

For peer review only - http://bmjopen.bmj.com/site/about/guidelines.xhtml

eTable 6. Incident hospital diagnoses in male UK Biobank participants by p.C282Y/H63D genotypes, excluding a diagnosis of hemochromatosis at baseline

**Males**	**No mutations**	**H63D +/-**	**H63D +/+**	**C282Y +/ H36D +**	**C282Y +/-**	**C282Y +/+**
Hemochromatosis	85 (14.41)	51 (8.64)	29 (4.92)	95 (16.10)	42 (7.12)	288 (48.81)
All-cause mortality	13959 (58.44)	5653 (23.66)	539 (2.26)	587 (2.46)	2990 (12.52)	160 (0.67)
**Liver**						
Liver disease (any)	3969 (58.38)	1619 (23.82)	158 (2.32)	167 (2.46)	800 (11.77)	85 (1.25)
Alcoholic liver disease	607 (58.20)	226 (21.67)	25 (2.40)	27 (2.59)	148 (14.19)	10 (0.96)
Fibrosis & Cirrhosis	618 (57.06)	250 (23.08)	23 (2.12)	25 (2.31)	140 (12.93)	27 (2.49)
Hepatic failure	355 (62.06)	137 (23.95)	10 (1.75)	15 (2.65)	54 (9.44)	1 (0.17)
**Cancer**						
Liver cancer	361 (55.28)	166 (25.42)	12 (1.84)	19 (2.91)	78 (11.94)	17 (2.60)
Prostate cancer	7722 (59.20)	3023 (23.18)	283 (2.17)	318 (2.44)	1610 (12.34)	87 (0.67)
**Musculoskeletal**						
Joint replacement surgery (any)	8427 (58.44)	3377 (23.42)	315 (2.18)	375 (2.60)	1801 (12.49)	126 (0.87)
Osteoarthritis	2872 (58.70)	1153 (23.56)	97 (1.98)	120 (2.45)	600 (12.26)	51 (1.04)
Fractures (any)	6090 (60.07)	2296 (22.65)	212 (2.09)	231 (2.28)	1243 (12.26)	66 (0.65)
Fragility fractures	2663 (60.63)	963 (21.93)	79 (1.80)	98 (2.23)	550 (12.52)	39 (0.89)
Osteoporosis	1521 (59.37)	601 (23.46)	42 (1.64)	63 (2.46)	319 (12.45)	16 (0.62)
Rheumatoid arthritis	1130 (58.55)	479 (24.82)	38 (1.97)	33 (1.71)	238 (12.33)	12 (0.62)
**Brain**						
Any brain outcome (dementia, delirium, or Parkinson's disease)	4616 (58.43)	1849 (23.41)	177 (2.24)	200 (2.53)	992 (12.56)	66 (0.84)
Dementia	2305 (58.67)	915 (23.29)	92 (2.34)	97 (2.47)	487 (12.40)	33 (0.84)
Alzheimer's disease	1026 (60.53)	380 (22.42)	38 (2.24)	50 (2.95)	189 (11.15)	12 (0.71)
Non-Alzheimer's dementia	1298 (57.38)	539 (23.83)	54 (2.39)	47 (2.08)	303 (13.40)	21 (0.93)
Delirium	2619 (58.29)	1025 (22.81)	95 (2.11)	111 (2.47)	602 (13.40)	41 (0.91)
Parkinson's disease	1115 (59.34)	454 (24.16)	46 (2.45)	43 (2.29)	202 (10.75)	19 (1.01)
**Pancreas**						

T2 diabetes	9570 (60.17)	3673 (23.09)	365 (2.30)	368 (2.31)	1831 (11.51)	97 (0.61)
**Infection**						
Covid-19	2331 (58.14)	953 (23.77)	96 (2.39)	76 (1.90)	522 (13.02)	31 (0.77)
Cholecystitis	1623 (60.07)	612 (22.65)	55 (2.04)	73 (2.70)	316 (11.70)	23 (0.85)
Pneumonia	8014 (59.44)	3124 (23.17)	273 (2.02)	340 (2.52)	1649 (12.23)	83 (0.62)
Sepsis	10075 (59.34)	3951 (23.27)	374 (2.20)	412 (2.43)	2063(12.15)	103 (0.61)
LRTI	4110 (58.12)	1691 (23.91)	148 (2.09)	164 (2.32)	913 (12.91)	45 (0.64)
URTI	588 (61.06)	226 (23.47)	21 (2.18)	17 (1.77)	108 (11.21)	3 (0.31)
UTI	7097 (59.29)	2769 (23.13)	263 (2.20)	286 (2.39)	1470 (12.28)	85 (0.71)
SSTI	4599 (58.27)	1790 (22.68)	176 (2.23)	215 (2.72)	1053 (13.34)	59 (0.75)
**Cardiovascular**						
Arrhythmia	2282 (60.40)	870 (23.03)	85 (2.25)	77 (2.04)	444 (11.75)	20 (0.53)
Cardiomyopathy	853 (59.86)	330 (23.16)	25 (1.75)	34 (2.39)	178 (12.49)	5 (0.35)
CHD	11793 (59.45)	4622 (23.30)	411 (2.07)	481 (2.42)	2414 (12.17)	115 (0.58)
Heart failure	5775 (58.87)	2285 (23.29)	211 (2.15)	227 (2.31)	1253 (12.77)	59 (0.60)
**Mental Health**						
Depression	3925 (59.18)	1614 (24.34)	132 (1.99)	150 (2.26)	771 (11.63)	40 (0.60)

Incident disease numbers exclude prevalent disease at baseline. Numbers presented are n (%). Abbreviations: CHD, coronary heart disease; T1, type 1; T2, type 2; LRTI, lower respiratory tract infection; URTI, upper respiratory tract infection; UTI, urinary tract infection; SSTI, skin and soft tissue infection. Joint replacement surgery variable includes a diagnosis of hip, knee, ankle, or shoulder replacement. Any brain outcome variable includes a diagnosis of dementia, delirium, or Parkinson’s disease.

For peer review only - http://bmjopen.bmj.com/site/about/guidelines.xhtml

eTable 7. Hazard ratios of incident disease outcomes in p.C282Y/H63D genotypes in males, excluding a diagnosis of hemochromatosis at baseline

Males	No mutations (Ref group)	H63D +/-		H63D +/+		C282Y +/ H36D +		C282Y +/-		C282Y +/+	
		HR (95% CI)	P	HR (95% CI)	P	HR (95% CI)	P	HR (95% CI)	P	HR (95% CI)	P
Hemochromatosis	1	1.53 (1.08-2.17)	0.02	8.95 (5.87-13.64)	$2.2*10^{-24}$	27.49 (20.50-36.86)	$1.0*10^{-108}$	2.40 (1.66-3.47)	$3.6*10^{-06}$	405.79 (317.34-518.75)	0.00E+00
All-cause mortality	1	1.03 (1.00-1.07)	0.04	1.01 (0.93-1.11)	0.74	1.02 (0.94-1.11)	0.58	1.05 (1.00-1.09)	0.03	1.22 (1.05-1.43)	0.01
**Liver**											
Liver disease (any)	1	1.05 (0.99-1.11)	0.13	1.05 (0.89-1.23)	0.57	1.04 (0.89-1.21)	0.63	1.00 (0.92-1.07)	0.9	2.36 (1.90-2.93)	$5.3*10^{-15}$
Alcoholic liver disease	1	0.95 (0.12-1.10)	0.48	1.07 (0.72-1.60)	0.74	1.04 (0.71-1.53)	0.84	1.15 (0.96-1.38)	0.12	1.59 (0.85-2.97)	0.15
Fibrosis & Cirrhosis	1	1.03 (0.89-1.20)	0.66	0.98 (0.64-1.48)	0.91	0.97 (0.65-1.44)	0.87	1.09 (0.91-1.31)	0.35	4.52 (3.07-6.66)	$2.1*10^{-14}$
Hepatic failure	1	0.99 (0.81-1.21)	0.92	0.75 (0.40-1.40)	0.36	1.05 (0.62-1.76)	0.86	0.75 (0.56-1.00)	0.05	0.31 (0.04-2.19)	0.24
**Cancer**											
Liver cancer	1	1.17 (0.98-1.41)	0.09	0.87 (0.49-1.54)	0.63	1.27 (0.80-2.02)	0.3	1.05 (0.82-1.34)	0.71	4.97 (3.05-8.11)	$1.2*10^{-10}$
Prostate cancer	1	1.00 (0.96-1.05)	0.87	0.97 (0.86-1.09)	0.57	1.04 (0.93-1.16)	0.51	1.05 (0.99-1.11)	0.09	1.27 (1.03-1.57)	0.03
**Musculoskeletal**											
Joint replacement surgery (any)	1	1.03 (0.99-1.07)	0.22	0.98 (0.88-1.10)	0.75	1.10 (0.99-1.22)	0.06	1.06 (1.01-1.12)	0.02	1.75 (1.47-2.09)	$4.8*10^{-10}$
Osteoarthritis	1	1.03 (0.96-1.10)	0.46	0.89 (0.73-1.09)	0.26	1.02 (0.85-1.22)	0.87	1.03 (0.94-1.12)	0.56	1.97 (1.50-2.60)	$1.5*10^{-06}$
Fractures (any)	1	0.96 (0.92-1.01)	0.12	0.92 (0.80-1.06)	0.24	0.94 (0.82-1.07)	0.34	1.01 (0.95-1.07)	0.82	1.17 (0.91-1.49)	0.22
Fragility fractures	1	0.92 (0.86-0.99)	0.03	0.78 (0.63-0.98)	0.03	0.91 (0.74-1.11)	0.34	1.02 (0.93-1.12)	0.68	1.60 (1.17-2.20)	$3.6*10^{-03}$
Osteoporosis	1	1.01 (0.92-1.11)	0.86	0.73 (0.54-0.99)	0.04	1.02 (0.79-1.31)	0.89	1.03 (0.91-1.16)	0.68	1.14 (0.69-1.86)	0.61
Rheumatoid arthritis	1	1.09 (0.98-1.21)	0.13	0.89 (0.64-1.23)	0.48	0.73 (0.52-1.03)	0.07	1.05 (0.91-1.20)	0.52	1.17 (0.66-2.07)	0.58
**Brain**											
Any brain outcome (dementia, delirium, or Parkinson's disease)	1	1.02 (0.97-1.08)	0.4	1.02 (0.88-1.18)	0.8	1.08 (0.93-1.24)	0.31	1.06 (0.99-1.13)	0.1	1.62 (1.27-2.07)	$1.0*10^{-04}$
Dementia	1	1.01 (0.94-1.09)	0.76	1.06 (0.86-1.31)	0.57	1.04 (0.85-1.28)	0.69	1.04 (0.94-1.14)	0.46	1.60 (1.14-2.26)	0.01
Alzheimer's disease	1	0.94 (0.84-1.06)	0.32	0.98 (0.71-1.36)	0.92	1.12 (0.91-1.61)	0.18	0.90 (0.77-1.06)	0.2	1.31 (0.74-2.31)	0.35
Non-Alzheimer's dementia	1	1.06 (0.96-1.17)	0.26	1.11 (0.85-1.46)	0.45	0.89 (0.67-1.20)	0.45	1.14 (1.01-1.30)	0.04	1.81 (1.17-2.78)	0.01
Delirium	1	1.00 (0.93-1.07)	0.98	0.96 (0.78-1.18)	0.7	1.05 (0.87-1.27)	0.59	1.13 (1.04-1.24)	0.01	1.75 (1.28-2.38)	$3.9*10^{-04}$
Parkinson's disease	1	1.07 (0.98-1.16)	0.13	1.05 (0.84-1.31)	0.67	1.06 (0.85-1.33)	0.58	1.01 (0.91-1.13)	0.79	1.44 (1.02-2.03)	0.04

For peer review only

For peer review only - http://bmjopen.bmj.com/site/about/guidelines.xhtml

**Pancreas**											
T1 or T2 diabetes	1	0.99 (0.95-1.02)	0.45	1.02 (0.92-1.14)	0.66	0.94 (0.85-1.05)	0.28	0.95 (0.91-1.00)	0.06	1.17 (0.96-1.43)	0.11
**Infection**											
COVID-19	1	1.05 (0.97-1.13)	0.23	1.09 (0.89-1.34)	0.39	0.81 (0.64-1.01)	0.07	1.11 (1.01-1.23)	0.03	1.51 (1.06-2.15)	0.02
Cholecystitis	1	0.96 (0.88-1.06)	0.44	0.89 (0.68-1.17)	0.4	1.11 (0.88-1.41)	0.37	0.96 (0.85-1.08)	0.51	1.54 (1.02-2.32)	0.04
Pneumonia	1	0.99 (0.95-1.04)	0.8	0.90 (0.80-1.02)	0.09	1.04 (0.93-1.16)	0.52	1.01 (0.96-1.07)	0.66	1.13 (0.91-1.40)	0.27
Sepsis	1	1.01 (0.97-1.04)	0.79	0.98 (0.88-1.09)	0.69	1.01 (0.92-1.12)	0.82	1.01 (0.96-1.06)	0.68	1.12 (0.92-1.36)	0.26
LRTI	1	1.05 (0.99-1.11)	0.1	0.94 (0.80-1.11)	0.48	0.96 (0.82-1.13)	0.63	1.08 (1.00-1.16)	0.04	1.14 (0.85-1.52)	0.4
URTI	1	0.99 (0.85-1.15)	0.85	0.94 (0.61-1.45)	0.78	0.71 (0.44-1.16)	0.17	0.91 (0.74-1.12)	0.37	0.55 (0.18-1.70)	0.3
UTI	1	1.00 (0.95-1.04)	0.86	0.98 (0.86-1.11)	0.71	1.00 (0.89-1.12)	0.96	1.02 (0.96-1.08)	0.51	1.32 (1.07-1.64)	0.01
SSTI	1	1.00 (0.94-1.05)	0.89	1.01 (0.87-1.18)	0.87	1.16 (1.01-1.33)	0.03	1.14 (1.07-1.22)	$1.4*10^{-04}$	1.40 (1.09-1.82)	0.01
**Cardiovascular**											
Arrhythmia	1	0.98 (0.90-1.06)	0.56	0.99 (0.80-1.23)	0.92	0.84 (0.67-1.06)	0.15	0.96 (0.87-1.07)	0.48	0.96 (0.62-1.49)	0.86
Cardiomyopathy	1	0.99 (0.87-1.13)	0.91	0.78 (0.52-1.16)	0.21	0.99 (0.71-1.40)	0.98	1.04 (0.89-1.22)	0.62	0.65 (0.27-1.57)	0.34
CHD	1	1.00 (0.97-1.04)	0.8	0.91 (0.83-1.01)	0.71	1.00 (0.91-1.09)	0.97	1.01 (0.97-1.06)	0.52	1.04 (0.87-1.25)	0.66
Heart failure	1	1.01 (0.96-1.06)	0.69	0.96 (0.84-1.11)	0.61	0.97 (0.85-1.10)	0.62	1.07 (1.01-1.14)	0.03	1.12 (0.87-1.45)	0.38
**Mental Health**											
Depression	1	1.05 (1.00-1.12)	0.07	0.88 (0.74-1.05)	0.17	0.95 (0.81-1.12)	0.55	0.98 (0.91-1.06)	0.6	1.13 (0.82-1.54)	0.46

HR (Hazard ratio) compared to those with neither *HFE* mutation. Cox proportional hazards regression models adjusted for age, assessment centre, and genetic principal components 1–10. Abbreviations: CHD, coronary heart disease; T1, type 1; T2, type 2; LRTI, lower respiratory tract infection; URTI, upper respiratory tract infection; UTI, urinary tract infection SSTI, skin and soft tissue infection; CI, confidence interval. Joint replacement surgery variable includes a diagnosis of hip, knee, ankle, or shoulder replacement. Any brain outcome variable includes a diagnosis of dementia, delirium, or Parkinson's disease.

For peer review only

eTable 8. Cumulative incidence of hemochromatosis from ages 40-80 years by HFE genotypes

	Total Cohort (n=451,270)			No mutations			H63D Heterozygotes			H63D Homozygotes			Compound C282Y/H63D Heterozygotes			p.C282Y Heterozygotes			p.C282Y homozygotes		
	Incident diagnosis			Incident diagnosis			Incident diagnosis			Incident diagnosis			Incident diagnosis			Incident diagnosis			Incident diagnosis		
	%	(95% CI)		%	(95% CI)		%	(95% CI)		%	(95% CI)		%	(95% CI)		%	(95% CI)		%	(95% CI)	
**Male age group (years)**																					
**40 - 45**	0.1	0.0	0.2	0.0	0.0	0.2	0.0	0.0	0.0	0.0	0.0	0.0	0.3	0.0	1.9	0.0	0.0	0.0	6.8	2.8	16.1
**46 - 50**	0.1	0.1	0.2	0.0	0.0	0.2	0.0	0.0	0.1	0.4	0.1	1.3	0.7	0.2	2.0	0.0	0.0	0.0	12.5	7.3	20.8
**51 - 55**	0.2	0.2	0.3	0.0	0.0	0.2	0.0	0.0	0.1	0.6	0.3	1.5	1.5	0.8	2.6	0.0	0.0	0.1	20.4	14.7	27.9
**56 - 60**	0.3	0.3	0.4	0.1	0.0	0.2	0.1	0.0	0.1	0.8	0.4	1.6	2.1	1.3	3.2	0.1	0.1	0.2	28.4	22.7	35.3
**61 - 65**	0.4	0.3	0.5	0.1	0.0	0.2	0.1	0.1	0.2	1.0	0.6	1.8	2.6	1.8	3.8	0.2	0.1	0.3	36.1	30.5	42.4
**66 - 70**	0.5	0.5	0.6	0.1	0.1	0.2	0.2	0.1	0.2	1.2	0.7	2.1	3.6	2.7	4.8	0.3	0.2	0.4	43.0	37.6	48.7
**71 - 75**	0.6	0.6	0.7	0.2	0.1	0.2	0.2	0.1	0.3	1.4	0.9	2.2	4.3	3.4	5.5	0.3	0.2	0.4	48.9	43.8	54.2
**76 - 80**	0.8	0.7	0.9	0.2	0.1	0.3	0.3	0.2	0.4	1.9	1.3	2.9	5.4	4.3	6.8	0.4	0.3	0.6	56.4	51.4	61.6
**Female age group (years)**																					
**40 - 45**	0.0	0.0	0.0	0.0	0.0	0.0	0.0	0.0	0.0	0.0	0.0	0.0	0.2	0.0	1.5	0.0	0.0	0.0	0.0	0.0	0.0
**46 - 50**	0.0	0.0	0.1	0.0	0.0	0.0	0.0	0.0	0.1	0.2	0.1	0.9	0.3	0.1	1.3	0.0	0.0	0.0	3.4	1.7	6.7
**51 - 55**	0.1	0.1	0.1	0.0	0.0	0.0	0.0	0.0	0.1	0.3	0.1	1.0	0.5	0.2	1.3	0.0	0.0	0.1	8.2	5.7	11.7
**56 - 60**	0.1	0.1	0.2	0.0	0.0	0.0	0.1	0.0	0.1	0.4	0.2	1.0	0.7	0.4	1.5	0.1	0.1	0.2	14.5	11.5	18.1
**61 - 65**	0.2	0.2	0.3	0.0	0.0	0.0	0.1	0.0	0.1	0.5	0.2	1.1	1.1	0.6	1.8	0.1	0.1	0.2	21.2	17.9	24.9
**66 - 70**	0.3	0.3	0.3	0.0	0.0	0.1	0.1	0.1	0.2	0.5	0.2	1.1	1.5	1.0	2.2	0.2	0.1	0.3	27.6	24.2	31.3
**71 - 75**	0.4	0.3	0.4	0.1	0.0	0.1	0.1	0.1	0.2	0.6	0.3	1.2	1.9	1.4	2.7	0.2	0.2	0.3	33.5	30.0	37.2
**76 - 80**	0.5	0.5	0.6	0.1	0.1	0.1	0.1	0.1	0.2	0.6	0.3	1.3	2.7	2.0	3.6	0.3	0.2	0.5	40.5	36.7	44.5

For peer review only - http://bmjopen.bmj.com/site/about/guidelines.xhtml

eTable 9. Incident hospital diagnoses in female UK Biobank participants by p.C282Y/H63D genotypes

**Females**	**No mutations**	**H63D +/-**	**H63D +/+**	**C282Y +/ H36D +**	**C282Y +/-**	**C282Y +/+**
Hemochromatosis	44 (0.03)	35 (0.1)	12 (0.2)	57 (1.0)	37 (0.1)	291 (18.8)
All-cause mortality	9,849 (6.8)	3,810 (6.7)	363 (6.5)	393 (6.8)	2,020 (6.9)	129 (8.0)
**Liver**						
Liver disease (any)	3,822 (2.6)	1,512 (2.7)	146 (2.6)	179 (3.1)	825 (2.9)	69 (4.3)
Alcoholic liver disease	181 (0.1)	78 (0.1)	11 (0.2)	6 (0.1)	59 (0.2)	7 (0.4)
Fibrosis & Cirrhosis	475 (0.3)	163 (0.3)	16 (0.3)	21 (0.4)	89 (0.3)	14 (0.9)
Hepatic failure	200 (0.1)	60 (0.1)	7 (0.1)	4 (0.1)	35 (0.1)	5 (0.3)
**Cancer**						
Liver cancer	232 (0.2)	114 (0.2)	10 (0.2)	8 (0.1)	49 (0.2)	3 (0.2)
**Musculoskeletal**						
Joint replacement surgery (any)	12,356 (8.7)	4,772 (8.6)	454 (8.4)	501 (8.9)	2,415 (8.5)	158 (10.3)
Osteoarthritis	4,344 (3.3)	1,681 (3.3)	177 (3.6)	190 (3.7)	893 (3.4)	70 (5.1)
Fractures (any)	10,666 (7.6)	4,204 (7.7)	376 (7.0)	430 (7.7)	2,189 (7.8)	124 (8.0)
Fragility fractures	6,308 (4.4)	2,517 (4.5)	214 (3.9)	275 (4.8)	1,265 (4.4)	63 (4.0)
Osteoporosis	6,990 (5.0)	2,580 (4.7)	254 (4.7)	245 (4.4)	1,390 (4.9)	103 (6.7)
Rheumatoid arthritis	2,189 (1.5)	819 (1.5)	79 (1.4)	87 (1.5)	391 (1.4)	26 (1.6)
**Brain**						
Any brain outcome (dementia, delirium, or Parkinson's disease)	3,876 (2.7)	1,547 (2.7)	142 (2.6)	155 (2.7)	777 (2.7)	61 (3.8)
Dementia	2,159 (1.5)	837 (1.5)	68 (1.2)	84 (1.5)	440 (1.5)	31 (1.9)
Alzheimer's disease	1,072 (0.7)	450 (0.8)	37 (0.7)	51 (0.9)	220 (0.8)	14 (0.9)
Non-Alzheimer's dementia	1,099 (0.8)	395 (0.7)	31 (0.6)	34 (0.6)	227 (0.8)	17 (1.1)
Delirium	1,983 (1.4)	823 (1.4)	81 (1.5)	82 (1.4)	406 (1.4)	36 (2.2)
Parkinson's disease	691 (0.5)	249 (0.4)	26 (0.5)	30 (0.5)	134 (0.5)	8 (0.5)
**Pancreas**						
T1 or T2 diabetes	6,532 (4.6)	2,583 (4.6)	245 (4.5)	278 (4.9)	1,327 (4.6)	87 (5.5)
**Infection**						
Covid-19	1,809 (1.2)	720 (1.3)	72 (1.3)	72 (1.3)	383 (1.3)	21 (1.3)
Cholecystitis	1,749 (1.2)	675 (1.2)	62 (1.1)	69 (1.2)	351 (1.2)	30 (1.9)
Pneumonia	6,130 (4.3)	2,408 (4.3)	242 (4.4)	255 (4.5)	1,276 (4.5)	75 (4.8)

Sepsis	10,026 (7.0)	3,840 (6.9)	379 (7.0)	414 (7.4)	2,103 (7.4)	133 (8.5)
LRTI	3,875 (2.7)	1,439 (2.5)	139 (2.5)	144 (2.5)	821 (2.8)	53 (3.3)
URTI	636 (0.4)	279 (0.5)	21 (0.4)	21 (0.4)	142 (0.5)	2 (0.1)
UTI	9,140 (6.5)	3,493 (6.4)	351 (6.6)	366 (6.7)	1,905 (6.8)	126 (8.2)
SSTI	3,900 (2.7)	1,534 (2.7)	158 (2.9)	153 (2.7)	799 (2.8)	53 (3.3)
**Cardiovascular**						
Arrhythmia	1,655 (1.1)	656 (1.2)	48 (0.9)	67 (1.2)	323 (1.1)	20 (1.3)
Cardiomyopathy	608 (0.4)	230 (0.4)	19 (0.3)	26 (0.5)	130 (0.5)	3 (0.2)
CHD	7,402 (5.3)	2,877 (5.2)	294 (5.4)	293 (5.3)	1,502 (5.3)	82 (5.3)
Heart failure	3,551 (2.4)	1,431 (2.5)	127 (2.3)	155 (2.7)	753 (2.6)	56 (3.5)
**Mental Health**						
Depression	6,415 (4.8)	2430 (4.6)	269 (5.2)	279 (5.2)	1317 (4.9)	64 (4.3)

Incident disease numbers exclude prevalent disease at baseline. Numbers presented are n (%). Abbreviations: CHD, coronary heart disease; T1, type 1; T2, type 2; LRTI, lower respiratory tract infection; URTI, upper respiratory tract infection; UTI, urinary tract infection; SSTI, skin and soft tissue infection. Joint replacement surgery variable includes a diagnosis of hip, knee, ankle, or shoulder replacement. Any brain outcome variable includes a diagnosis of dementia, delirium, or Parkinson's disease.

For peer review only - http://bmjopen.bmj.com/site/about/guidelines.xhtml

eTable 10. Hazard ratios of incident disease outcomes in p.C282Y/H63D genotypes in females

**Females**	**No mutations**	**H63D +/-**		**H63D +/+**		**C282Y +/ H36D +**		**C282Y +/-**		**C282Y +/+**	
		HR (95% CI)	P	HR (95% CI)	P	HR (95% CI)	P	HR (95% CI)	P	HR (95% CI)	P
Hemochromatosis	1	2.04 (1.31-3.17)	$1.70*10^{-03}$	7.10 (3.75-13.45)	$1.80*10^{-09}$	32.78 (22.10-48.63)	$2.20*10^{-67}$	4.15 (2.68-6.44)	$1.80*10^{-10}$	674.10 (489.11-929.05)	$2.47*10^{-346}$
All-cause mortality	1	0.99 (0.96-1.03)	0.68	0.95 (0.85-1.05)	0.3	1.01 (0.91-1.11)	0.9	1.01 (0.96-1.06)	0.62	1.10 (0.92-1.31)	0.28
**Liver**											
Liver disease (any)	1	1.01 (0.95-1.07)	0.72	1.00 (0.84-1.17)	0.96	1.19 (1.02-1.38)	0.03	1.08 (1.00-1.16)	0.06	1.62 (1.27-2.05)	$7.80*10^{-05}$
Alcoholic liver disease	1	1.09 (0.84-1.43)	0.51	1.56 (0.85-2.87)	0.15	0.78 (0.35-1.77)	0.56	1.53 (1.14-2.05)	0.005	3.07 (1.44-6.54)	0.004
Fibrosis & Cirrhosis	1	0.87 (0.73-1.04)	0.13	0.87 (0.53-1.43)	0.58	1.10 (0.71-1.71)	0.67	0.92 (0.74-1.16)	0.49	2.56 (1.50-4.36)	0.001
Hepatic failure	1	0.77 (0.58-1.02)	0.07	0.92 (0.43-1.94)	0.82	0.49 (0.18-1.33)	0.16	0.85 (0.59-1.21)	0.37	2.09 (0.86-5.08)	0.1
**Cancer**											
Liver cancer	1	1.26 (1.01-1.58)	0.04	1.13 (0.60-2.13)	0.71	0.89 (0.44-1.81)	0.75	1.06 (0.78-1.45)	0.69	1.17 (0.37-3.66)	0.79
**Musculoskeletal**											
Joint replacement surgery (any)	1	0.99 (0.96-1.02)	0.55	0.97 (0.88-1.06)	0.47	1.04 (0.95-1.14)	0.34	0.99 (0.94-1.03)	0.51	1.18 (1.01-1.38)	0.04
Osteoarthritis	1	0.99 (0.94-1.05)	0.71	1.06 (0.91-1.23)	0.46	1.10 (0.95-1.27)	0.21	1.02 (0.95-1.10)	0.6	1.44 (1.14-1.82)	0.002
Fractures (any)	1	1.01 (0.97-1.04)	0.65	0.91 (0.82-1.01)	0.07	1.01 (0.92-1.12)	0.79	1.02 (0.97-1.07)	0.42	1.00 (0.84-1.20)	0.96
Fragility fractures	1	1.02 (0.98-1.07)	0.38	0.87 (0.76-1.00)	0.05	1.10 (0.97-1.24)	0.13	0.99 (0.93-1.05)	0.74	0.85 (0.66-1.09)	0.19
Osteoporosis	1	0.94 (0.90-0.99)	0.01	0.94 (0.83-1.07)	0.34	0.89 (0.79-1.01)	0.08	0.99 (0.94-1.05)	0.75	1.28 (1.06-1.56)	0.01
Rheumatoid arthritis	1	0.96 (0.88-1.04)	0.28	0.94 (0.75-1.17)	0.58	1.01 (0.82-1.26)	0.9	0.89 (0.80-0.99)	0.03	1.04 (0.71-1.53)	0.84
**Brain**											
Any brain outcome (dementia, delirium, or Parkinson's disease)	1	1.02 (0.97-1.09)	0.43	0.93 (0.79-1.11)	0.43	1.03 (0.88-1.21)	0.72	1.00 (0.92-1.08)	0.9	1.30 (1.01-1.68)	0.04
Dementia	1	1.00 (0.92-1.08)	0.94	0.80 (0.63-1.02)	0.07	1.00 (0.81-1.25)	0.98	1.01 (0.91-1.12)	0.86	1.16 (0.81-1.66)	0.41
Alzheimer's disease	1	1.08 (0.97-1.20)	0.18	0.87 (0.63-1.20)	0.4	1.22 (0.92-1.61)	0.17	1.02 (0.88-1.17)	0.84	1.04 (0.62-1.77)	0.88

For peer review only

For peer review only - http://bmjopen.bmj.com/site/about/guidelines.xhtml

Non-Alzheimer's dementia	1	0.93 (0.83-1.04)	0.2	0.72 (0.50-1.03)	0.07	0.81 (0.57-1.13)	0.21	1.03 (0.89-1.18)	0.73	1.27 (0.76-2.05)	0.33
Delirium	1	1.06 (0.98-1.15)	0.13	1.05 (0.84-1.31)	0.67	1.06 (0.85-1.32)	0.6	1.01 (0.91-1.13)	0.8	1.50 (1.08-2.08)	0.02
Parkinson's disease	1	0.93 (0.80-1.07)	0.29	0.97 (0.66-1.44)	0.89	1.14 (0.79-1.65)	0.48	0.98 (0.81-1.18)	0.84	1.00 (0.50-2.00)	0.99
**Pancreas**											
T1 or T2 diabetes	1	1.01 (0.97-1.06)	0.55	0.98 (0.86-1.11)	0.77	1.09 (0.97-1.23)	0.17	1.01 (0.96-1.08)	0.6	1.18 (0.95-1.46)	0.13
**Infection**											
COVID-19	1	1.02 (0.94-1.11)	0.66	1.03 (0.81-1.30)	0.83	1.02 (0.80-1.29)	0.9	1.06 (0.94-1.18)	0.34	1.00 (0.65-1.54)	0.99
Cholecystitis	1	0.98 (0.90-1.08)	0.73	0.92 (0.71-1.18)	0.52	0.99 (0.78-1.26)	0.92	0.99 (0.88-1.11)	0.86	1.49 (1.04-2.13)	0.03
Pneumonia	1	1.01 (0.96-1.05)	0.83	1.02 (0.90-1.16)	0.72	1.06 (0.93-1.20)	0.4	1.03 (0.97-1.10)	0.29	1.05 (0.83-1.32)	0.69
Sepsis	1	0.98 (0.94-1.01)	0.23	0.97 (0.88-1.08)	0.61	1.05 (0.95-1.15)	0.36	1.04 (0.99-1.09)	0.12	1.13 (0.96-1.35)	0.15
LRTI	1	0.95 (0.89-1.01)	0.08	0.92 (0.78-1.09)	0.34	0.93 (0.79-1.10)	0.39	1.04 (0.97-1.12)	0.3	1.16 (0.89-1.52)	0.28
URTI	1	1.12 (0.98-1.30)	0.1	0.86 (0.56-1.33)	0.51	0.84 (0.54-1.30)	0.43	1.12 (0.93-1.34)	0.24	0.28 (0.07-1.12)	0.07
UTI	1	0.97 (0.94-1.01)	0.18	0.99 (0.89-1.10)	0.82	1.01 (0.91-1.13)	0.8	1.03 (0.98-1.08)	0.23	1.19 (0.99-1.41)	0.06
SSTI	1	1.01 (0.95-1.07)	0.79	1.05 (0.90-1.23)	0.54	1.00 (0.85-1.18)	0.97	1.03 (0.95-1.11)	0.52	1.20 (0.92-1.58)	0.18
**Cardiovascular**											
Arrhythmia	1	1.02 (0.93-1.11)	0.69	0.76 (0.57-1.01)	0.06	1.04 (0.81-1.33)	0.75	0.98 (0.87-1.11)	0.78	1.07 (0.69-1.67)	0.75
Cardiomyopathy	1	0.97 (0.83-1.13)	0.71	0.82 (0.52-1.30)	0.41	1.11 (0.75-1.64)	0.61	1.08 (0.90-1.31)	0.41	0.44 (0.14-1.37)	0.16
CHD	1	1.00 (0.95-1.04)	0.82	1.03 (0.92-1.16)	0.61	1.01 (0.90-1.14)	0.86	1.01 (0.96-1.07)	0.71	0.94 (0.76-1.17)	0.6
Heart failure	1	1.04 (0.97-1.10)	0.27	0.92 (0.77-1.10)	0.35	1.12 (0.96-1.32)	0.15	1.06 (0.98-1.15)	0.16	1.34 (1.03-1.75)	0.03
**Mental Health**											
Depression	1	0.97 (0.93-1.02)	0.2	1.10 (0.97-1.24)	0.14	1.11 (0.99-1.25)	0.09	1.03 (0.97-1.09)	0.38	0.90 (0.70-1.15)	0.39

HR (Hazard ratio) compared to those with neither *HFE* mutation. Cox proportional hazards regression models adjusted for age, assessment centre, and genetic principal components 1–10. Abbreviations: CHD, coronary heart disease; T1, type 1; T2, type 2; LRTI, lower respiratory tract infection; URTI, upper respiratory tract infection; UTI, urinary tract infection SSTI, skin and soft tissue infection; CI, confidence interval. Joint replacement surgery variable includes a diagnosis of hip, knee, ankle, or shoulder replacement. Any brain outcome variable includes a diagnosis of dementia, delirium, or Parkinson's disease.

For peer review only

For peer review only - http://bmjopen.bmj.com/site/about/guidelines.xhtml

eTable 11. Incident hospital diagnoses in female UK Biobank participants by p.C282Y/H63D genotypes, excluding a diagnosis of hemochromatosis at baseline

**Females**	**No mutations**	**H63D +/-**	**H63D +/+**	**C282Y +/ H36D +**	**C282Y +/-**	**C282Y +/+**
Hemochromatosis	44 (9.24)	35 (7.35)	12 (2.52)	57 (11.97)	37 (7.77)	291 (61.13)
All-cause mortality	9847 (59.47)	3810 (23.01)	363 (2.19)	390 (2.36)	2020 (12.20)	127 (0.77)
**Liver**						
Liver disease (any)	3821 (58.38)	1511 (23.09)	146 (2.23)	177 (2.70)	825 (12.61)	65 (0.99)
Alcoholic liver disease	180 (52.94)	78 (22.94)	11 (3.24)	6 (1.76)	59 (17.35)	6 (1.76)
Fibrosis & Cirrhosis	473 (61.03)	162 (20.90)	16 (2.06)	21 (2.71)	89 (11.48)	14 (1.81)
Hepatic failure	199 (64.40)	60 (19.42)	7 (2.27)	4 (1.29)	35 (11.33)	4 (1.29)
**Cancer**						
Liver cancer	232 (55.77)	114 (27.40)	10 (2.40)	8 (1.92)	49 (11.78)	3 (0.72)
**Musculoskeletal**						
Joint replacement surgery (any)	12355 (59.84)	4771 (23.11)	454 (2.20)	500 (2.42)	2415 (11.70)	150 (0.73)
Osteoarthritis	4343 (59.08)	1681 (22.87)	177 (2.41)	189 (2.57)	893 (12.15)	68 (0.93)
Fractures (any)	10665 (59.31)	4204 (23.38)	375 (2.09)	430 (2.39)	2188 (12.17)	119 (0.66)
Fragility fractures	6308 (59.29)	2517 (23.66)	214 (2.01)	275 (2.58)	1264 (11.88)	61 (0.57)
Osteoporosis	6990 (60.50)	2580 (22.33)	254 (2.20)	244 (2.11)	1389 (12.02)	97 (0.84)
Rheumatoid arthritis	2188 (61.05)	818 (22.82)	79 (2.20)	86 (2.40)	390 (10.88)	23 (0.64)
**Brain**						
Any brain outcome (dementia, delirium, or Parkinson's disease)	3875 (59.16)	1547 (23.62)	142 (2.17)	154 (2.35)	777 (11.86)	55 (0.84)
Dementia	2159 (59.72)	837 (23.15)	68 (1.88)	83 (2.30)	440 (12.17)	28(0.77)
Alzheimer's disease	1072 (58.20)	450 (24.43)	37 (2.01)	50 (2.71)	220 (11.94)	13 (0.71)
Non-Alzheimer's dementia	1099 (61.02)	395 (21.93)	31 (1.72)	34(1.89)	227 (12.60)	15 (0.83)
Delirium	1982 (58.17)	823 (24.16)	81 (2.38)	82 (2.41)	406 (11.92)	33 (0.97)

For peer review only - http://bmjopen.bmj.com/site/about/guidelines.xhtml

Parkinson's disease	691 (60.72)	249 (21.88)	26 (2.28)	30 (2.64)	134 (11.78)	8 (0.70)
**Pancreas**						
T2 diabetes	6538 (59.27)	2575 (23.35)	240 (2.18)	275 (2.49)	1322 (11.99)	80 (0.73)
**Infection**						
Covid-19	1809 (58.83)	719 (23.38)	72 (2.34)	72 (2.34)	383 (12.46)	20 (0.65)
Cholecystitis	1749 (59.61)	675 (23.01)	62 (2.11)	68 (2.32)	351 (11.96)	29 (0.99)
Pneumonia	6127 (59.06)	2406 (23.19)	242 (2.33)	254 (2.45)	1275 (12.29)	70 (0.67)
Sepsis	10024 (59.37)	3837 (22.72)	379 (2.24)	414 (2.45)	2103 (12.45)	128 (0.76)
LRTI	3875 (59.94)	1437 (22.23)	139 (2.15)	143 (2.21)	821 (12.70)	50 (0.77)
URTI	636 (57.87)	277 (25.20)	21 (1.91)	21 (1.91)	142 (12.92)	2 (0.18)
UTI	9137 (59.45)	3490 (22.71)	351 (2.28)	366 (2.38)	1905 (12.40)	119 (0.77)
SSTI	3900 (59.15)	1533 (23.25)	158 (2.40)	152 (2.31)	799 (12.12)	51 (0.77)
**Cardiovascular**						
Arrhythmia	1654 (59.75)	656 (23.70)	48 (1.73)	67 (2.42)	323 (11.67)	20 (0.72)
Cardiomyopathy	608 (59.90)	230 (22.66)	19 (1.87)	25 (2.46)	130 (12.81)	3 (0.30)
CHD	7401 (59.49)	2875 (23.11)	294 (2.36)	291 (2.34)	1502 (12.07)	77 (0.62)
Heart failure	3550 (58.50)	1430 (23.57)	127 (2.09)	154 (2.54)	753 (12.41)	54 (0.89)
**Mental Health**						
Depression	6414 (59.58)	2430 (22.57)	269 (2.50)	278 (2.58)	1317 (12.23)	57 (0.53)

Incident disease numbers exclude prevalent disease at baseline. Numbers presented are n (%). Abbreviations: CHD, coronary heart disease; T1, type 1; T2, type 2; LRTI, lower respiratory tract infection; URTI, upper respiratory tract infection; UTI, urinary tract infection; SSTI, skin and soft tissue infection. Joint replacement surgery variable includes a diagnosis of hip, knee, ankle, or shoulder replacement. Any brain outcome variable includes a diagnosis of dementia, delirium, or Parkinson's disease.

For peer review only - http://bmjopen.bmj.com/site/about/guidelines.xhtml

eTable 12. Hazard ratios of incident disease outcomes in p.C282Y/H63D genotypes in females, excluding a diagnosis of hemochromatosis at baseline

**Females**	**No mutations (Ref group)**	**H63D +/-**		**H63D +/+**		**C282Y +/ H36D +**		**C282Y +/-**		**C282Y +/+**	
		HR (95% CI)	P	HR (95% CI)	P	HR (95% CI)	P	HR (95% CI)	P	HR (95% CI)	P
Hemochromatosis	1	2.04 (1.31-3.17)	**1.70E-03**	7.10 (3.75-13.45)	**1.80E-09**	32.79 (22.10-48.64)	**2.10E-67**	4.15 (2.68-6.44)	**1.80E-10**	675.18 (489.89-930.55)	**0.00E+00**
All-cause mortality	1	0.99 (0.96-1.03)	0.69	0.95 (0.85-1.05)	0.31	1.00 (0.90-1.11)	0.98	1.01 (0.97-1.06)	0.61	1.13 (0.95-1.35)	0.16
**Liver**											
Liver disease (any)	1	1.01 (0.95-1.07)	0.73	1.00 (0.84-1.18)	0.97	1.18 (1.01-1.37)	**0.03**	1.08 (1.00-1.16)	0.06	1.58 (1.23-2.02)	**2.80E-04**
Alcoholic liver disease	1	1.10 (0.84-1.43)	0.48	1.57 (0.85-2.88)	0.15	0.79 (0.35-1.78)	0.57	1.54 (1.14-2.06)	**0.43**	2.75 (1.21-6.20)	**0.02**
Fibrosis & Cirrhosis	1	0.87 (0.73-1.04)	0.13	0.87 (0.53-1.44)	0.60	1.11 (0.72-1.72)	0.65	0.93 (0.74-1.16)	0.51	2.67 (1.57-4.55)	**3.00E-04**
Hepatic failure	1	0.77 (0.58-1.03)	0.08	0.92 (0.43-1.95)	0.83	0.50 (0.18-1.34)	0.17	0.85 (0.59-1.22)	0.38	1.76 (0.65-4.74)	0.26
**Cancer**											
Liver cancer	1	1.26 (1.10-1.58)	**0.04**	1.13 (0.60-2.13)	0.71	0.89 (0.44-1.81)	0.75	1.06 (0.78-1.45)	0.69	1.22 (0.39-3.81)	0.73
**Musculoskeletal**											
Joint replacement surgery (any)	1	0.99 (0.96-1.02)	0.54	0.97 (0.88-1.06)	0.47	1.04 (0.95-1.14)	0.35	0.99 (0.94-1.03)	0.51	1.16 (0.99-1.37)	**0.07**
Osteoarthritis	1	0.99 (0.94-1.05)	0.72	1.06 (0.91-1.23)	0.46	1.09 (0.95-1.27)	0.22	1.02 (0.95-1.10)	0.59	1.46 (1.15-1.85)	**2.10E-03**
Fractures (any)	1	1.01 (0.97-1.05)	0.65	0.91 (0.82-1.01)	0.07	1.02 (0.92-1.12)	0.76	1.02 (0.97-1.07)	0.42	1.01 (0.84-1.20)	0.95
Fragility fractures	1	1.02 (0.98-1.07)	0.37	0.87 (0.76-1.00)	**0.05**	1.10 (0.98-1.24)	0.12	0.99 (0.93-1.05)	0.73	0.86 (0.67-1.10)	0.23
Osteoporosis	1	0.94 (0.90-0.99)	**0.01**	0.94 (0.83-1.07)	0.34	0.89 (0.78-1.01)	0.08	0.99 (0.93-1.05)	0.73	1.26 (1.03-1.54)	**0.02**
Rheumatoid arthritis	1	0.96 (0.88-1.04)	0.28	0.94 (0.75-1.17)	0.58	1.00 (0.81-1.25)	0.97	0.89 (0.80-0.99)	**0.03**	0.96 (0.63-1.44)	0.83
**Brain**											
Any brain outcome (dementia, delirium, or Parkinson's disease)	1	1.02 (0.97-1.09)	0.43	0.93 (0.79-1.11)	0.43	1.03 (0.87-1.20)	0.76	1.00 (0.92-1.08)	0.91	1.23 (0.94-1.61)	0.13
Dementia	1	1.00 (0.92-1.08)	0.94	0.80 (0.63-1.02)	0.07	0.99 (0.80-1.24)	0.95	1.01 (0.91- 1.12)	0.86	1.10 (0.76-1.60)	0.61
Alzheimer's disease	1	1.08 (0.97-1.20)	0.18	0.86 (0.63-1.21)	0.40	1.20 (0.90-1.59)	0.21	1.02 (0.88-1.17)	0.84	1.02 (0.59-1.76)	0.95

Non-Alzheimer's dementia	1	0.93 (0.83-1.04)	0.20	0.72 (0.50-1.03)	0.07	0.81 (0.57-1.14)	0.22	1.03 (0.89-1.18)	0.73	1.17 (0.71-1.96)	0.54
Delirium	1	1.07 (0.98-1.16)	0.13	1.05 (0.84-1.31)	0.67	1.06 (0.85-1.33)	0.58	1.01 (0.91-1.13)	0.79	1.44 (1.02-2.03)	**0.04**
Parkinson's disease	1	0.93 (0.80-1.07)	0.29	0.97 (0.66-1.44)	0.89	1.15 (0.79-1.65)	0.47	0.98 (0.81-1.18)	0.84	1.04 (0.52-2.10)	0.91
**Pancreas**											
T1 or T2 diabetes	1	1.01 (0.97-1.06)	0.54	0.98 (0.86-1.11)	0.72	1.08 (0.96-1.22)	0.20	1.02 (0.96-1.08)	0.59	1.13 (0.90-1.41)	0.28
**Infection**											
COVID-19	1	1.02 (0.93-1.11)	0.68	1.03 (0.81-1.30)	0.83	1.02 (0.80-1.29)	0.88	1.06 (0.94-1.18)	0.34	1.00 (0.64-1.56)	1.00
Cholecystitis	1	0.98 (0.90-1.08)	0.73	0.92 (0.71-1.19)	0.52	0.98 (0.77-1.24)	0.84	0.99 (0.88-1.11)	0.86	1.49 (1.03-2.16)	**0.03**
Pneumonia	1	1.00 (0.96-1.05)	0.84	1.02 (0.90-1.17)	0.71	1.05 (0.93-1.20)	0.41	1.03 (0.97-1.10)	0.29	1.02 (0.81-1.29)	0.85
Sepsis	1	0.98 (0.94-1.01)	0.22	0.97 (0.88-1.08)	0.62	1.05 (0.95-1.16)	0.34	1.04 (0.99-1.09)	0.11	1.14 (0.96-1.36)	0.14
LRTI	1	0.95 (0.89-1.01)	0.07	0.92 (0.78-1.09)	0.34	0.93 (0.78-1.09)	0.36	1.04 (0.97-1.12)	0.29	1.14 (0.86-1.51)	0.35
URTI	1	1.12 (0.97-1.29)	0.13	0.86 (0.56-1.33)	0.51	0.84 (0.54-1.30)	0.43	1.12 (0.93-1.34)	0.24	0.29 (0.07-1.17)	0.08
UTI	1	0.97 (0.94-1.01)	0.17	0.99 (0.89-1.10)	0.82	1.02 (0.92-1.13)	0.76	1.03 (0.98-1.08)	0.22	1.17 (0.97-1.40)	0.10
SSTI	1	1.01 (0.95-1.07)	0.81	1.05 (0.90-1.23)	0.54	1.00 (0.85-1.17)	0.98	1.03 (0.95-1.11)	0.52	1.20 (0.91-1.59)	0.19
**Cardiovascular**											
Arrhythmia	1	1.02 (0.93-1.12)	0.69	0.76 (0.57-1.01)	0.06	1.04 (0.82-1.33)	0.73	0.98 (0.87-1.11)	0.78	1.13 (0.72-1.75)	0.60
Cardiomyopathy	1	0.97 (0.83-1.13)	0.71	0.82 (0.52-1.30)	0.41	1.07 (0.72-1.59)	0.75	1.08 (0.90-1.31)	0.40	0.46 (0.15-1.43)	0.18
CHD	1	0.99 (0.95-1.04)	0.81	1.03 (0.92-1.16)	0.60	1.01 (0.89-1.13)	0.92	1.01 (0.96-1.07)	0.70	0.92 (0.74-1.15)	0.48
Heart failure	1	1.03 (0.97-1.10)	0.28	0.92 (0.77-1.10)	0.36	1.12 (0.95-1.32)	0.17	1.06 (0.98-1.15)	0.15	1.35 (1.03-1.77)	**0.03**
**Mental Health**											
Depression	1	0.97 (0.93-1.02)	0.20	1.10 (0.97-1.23)	0.14	1.11 (0.98-1.25)	0.09	1.03 (0.97-1.09)	0.37	0.83 (0.64-1.08)	0.16

HR (Hazard ratio) compared to those with neither *HFE* mutation. Cox proportional hazards regression models adjusted for age, assessment centre, and genetic principal components 1–10. Abbreviations: CHD, coronary heart disease; T1, type 1; T2, type 2; LRTI, lower respiratory tract infection; URTI, upper respiratory tract infection; UTI, urinary tract infection SSTI, skin and soft tissue infection; CI, confidence interval. Joint replacement surgery variable includes a diagnosis of hip, knee, ankle, or shoulder replacement. Any brain outcome variable includes a diagnosis of dementia, delirium, or Parkinson's disease

For peer review only

For peer review only - http://bmjopen.bmj.com/site/about/guidelines.xhtml

eFigure 1. Kaplan-Meier curves for the cumulative incidence of (a) mortality, (b) liver disease, (c) any joint replacement, and (d) any brain outcome, in male *HFE* p.C282Y homozygotes compared to those with no mutations, excluding a diagnosis of hemochromatosis at baseline

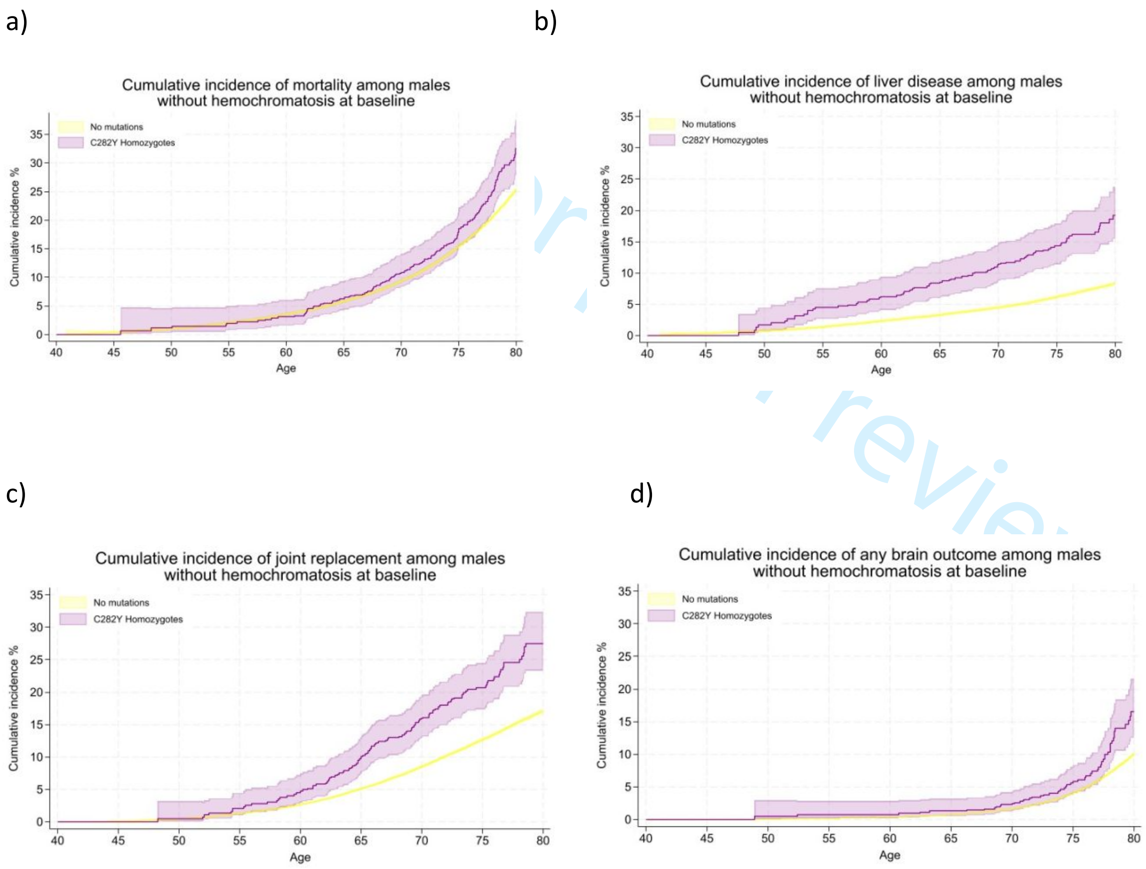

Cumulative incidence (estimated % diagnosed by age 80 years; 95% CIs). Joint replacement surgery includes a diagnosis of hip, knee, ankle, or shoulder replacement. Any brain outcome included a diagnosis of delirium, dementia, or Parkinson's disease.

For peer review only - http://bmjopen.bmj.com/site/about/guidelines.xhtml

eFigure 2. Kaplan-Meier curves for the cumulative incidence of (a) liver disease, and (b) any joint replacement, in female *HFE* p.C282Y homozygotes compared to those with no mutations, excluding a diagnosis of hemochromatosis at baseline

a)

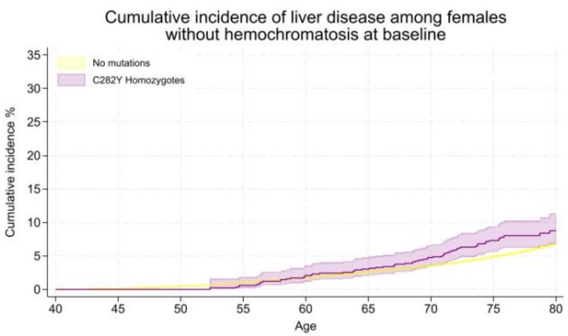

b)

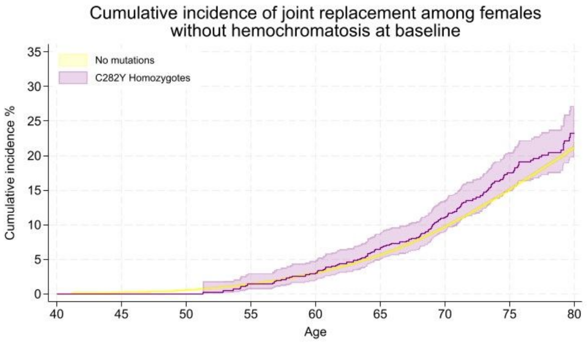

Cumulative incidence (estimated % diagnosed by age 80 years; 95% CIs). Joint replacement surgery includes a diagnosis of hip, knee, ankle, or shoulder replacement.

For peer review only - http://bmjopen.bmj.com/site/about/guidelines.xhtml

STROBE 2007 (v4) Statement—Checklist of items that should be included in reports of *cohort studies*

**Section/Topic**	**Item #**	**Recommendation**	**Reported on page #**
**Title and abstract**	1	(*a*) Indicate the study's design with a commonly used term in the title or the abstract	1-2
		(*b*) Provide in the abstract an informative and balanced summary of what was done and what was found	2
**Introduction**			
Background/rationale	2	Explain the scientific background and rationale for the investigation being reported	4-5
Objectives	3	State specific objectives, including any prespecified hypotheses	5
**Methods**			
Study design	4	Present key elements of study design early in the paper	5
Setting	5	Describe the setting, locations, and relevant dates, including periods of recruitment, exposure, follow-up, and data collection	5-6
Participants	6	(*a*) Give the eligibility criteria, and the sources and methods of selection of participants. Describe methods of follow-up	5-6
		(*b*) For matched studies, give matching criteria and number of exposed and unexposed	N/A
Variables	7	Clearly define all outcomes, exposures, predictors, potential confounders, and effect modifiers. Give diagnostic criteria, if applicable	5-6
Data sources/ measurement	8*	For each variable of interest, give sources of data and details of methods of assessment (measurement). Describe comparability of assessment methods if there is more than one group	*5-6*
Bias	9	Describe any efforts to address potential sources of bias	5
Study size	10	Explain how the study size was arrived at	5
Quantitative variables	11	Explain how quantitative variables were handled in the analyses. If applicable, describe which groupings were chosen and why	5-6
Statistical methods	12	(*a*) Describe all statistical methods, including those used to control for confounding	6
		(*b*) Describe any methods used to examine subgroups and interactions	6
		(*c*) Explain how missing data were addressed	N/A
		(*d*) If applicable, explain how loss to follow-up was addressed	N/A
		(*e*) Describe any sensitivity analyses	6
**Results**			

For peer review only

Participants	13*	(a) Report numbers of individuals at each stage of study—eg numbers potentially eligible, examined for eligibility, confirmed eligible, included in the study, completing follow-up, and analysed	6-7
		(b) Give reasons for non-participation at each stage	N/A
		(c) Consider use of a flow diagram	N/A
Descriptive data	14*	(a) Give characteristics of study participants (eg demographic, clinical, social) and information on exposures and potential confounders	6-7, 18 *Supplement Material* pages: 3-4
		(b) Indicate number of participants with missing data for each variable of interest	N/A
		(c) Summarise follow-up time (eg, average and total amount)	6
Outcome data	15*	Report numbers of outcome events or summary measures over time	*Supplement Material,* pages 5-6, 9-10, 14-15, 18-19
Main results	16	(*a*) Give unadjusted estimates and, if applicable, confounder-adjusted estimates and their precision (eg, 95% confidence interval). Make clear which confounders were adjusted for and why they were included	6-9 *Supplement Material,* pages: 7-8, 11-12, 13, 16-17, 20-21
		(*b*) Report category boundaries when continuous variables were categorized	N/A
		(*c*) If relevant, consider translating estimates of relative risk into absolute risk for a meaningful time period	N/A
Other analyses	17	Report other analyses done—eg analyses of subgroups and interactions, and sensitivity analyses	7
**Discussion**			
Key results	18	Summarise key results with reference to study objectives	10
**Limitations**			10-12
Interpretation	20	Give a cautious overall interpretation of results considering objectives, limitations, multiplicity of analyses, results from similar studies, and other relevant evidence	10-12
Generalisability	21	Discuss the generalisability (external validity) of the study results	10-12
**Other information**			
Funding	22	Give the source of funding and the role of the funders for the present study and, if applicable, for the original study on which the present article is based	13

*Give information separately for cases and controls in case-control studies and, if applicable, for exposed and unexposed groups in cohort and cross-sectional studies.

For peer review only

Note: An Explanation and Elaboration article discusses each checklist item and gives methodological background and published examples of transparent reporting. The STROBE checklist is best used in conjunction with this article (freely available on the Web sites of PLoS Medicine at http://www.plosmedicine.org/, Annals of Internal Medicine at http://www.annals.org/, and Epidemiology at http://www.epidem.com/). Information on the STROBE Initiative is available at www.strobe-statement.org.

For peer review only

HFE genotypes, haemochromatosis diagnosis and clinical outcomes to age 80: a prospective analysis in UK Biobank

Journal:	*BMJ Open*
Manuscript ID	bmjopen-2023-081926.R1
Article Type:	Original research
Date Submitted by the Author:	04-Jan-2024
Complete List of Authors:	Lucas, Mitchell; University of Exeter<br>Atkins, Janice; University of Exeter<br>Pilling, Luke; University of Exeter, Epidemiology and Public Health<br>Shearman, Jeremy; South Warwickshire University NHS Foundation Trust<br>Melzer, David; University of Exeter
**Primary Subject Heading**:	Epidemiology
Secondary Subject Heading:	Genetics and genomics
Keywords:	GENETICS, Other metabolic, e.g. iron, porphyria < DIABETES & ENDOCRINOLOGY, Mortality, Hepatology < INTERNAL MEDICINE

I, the Submitting Author has the right to grant and does grant on behalf of all authors of the Work (as defined in the below author licence), an exclusive licence and/or a non-exclusive licence for contributions from authors who are: i) UK Crown employees; ii) where BMJ has agreed a CC-BY licence shall apply, and/or iii) in accordance with the terms applicable for US Federal Government officers or employees acting as part of their official duties; on a worldwide, perpetual, irrevocable, royalty-free basis to BMJ Publishing Group Ltd ("BMJ") its licensees and where the relevant Journal is co-owned by BMJ to the co-owners of the Journal, to publish the Work in this journal and any other BMJ products and to exploit all rights, as set out in our licence.

The Submitting Author accepts and understands that any supply made under these terms is made by BMJ to the Submitting Author unless you are acting as an employee on behalf of your employer or a postgraduate student of an affiliated institution which is paying any applicable article publishing charge ("APC") for Open Access articles. Where the Submitting Author wishes to make the Work available on an Open Access basis (and intends to pay the relevant APC), the terms of reuse of such Open Access shall be governed by a Creative Commons licence – details of these licences and which Creative Commons licence will apply to this Work are set out in our licence referred to above.

Other than as permitted in any relevant BMJ Author's Self Archiving Policies, I confirm this Work has not been accepted for publication elsewhere, is not being considered for publication elsewhere and does not duplicate material already published. I confirm all authors consent to publication of this Work and authorise the granting of this licence.

For peer review only

HFE genotypes, haemochromatosis diagnosis and clinical outcomes to age 80: a prospective analysis in UK Biobank

Mitchell R Lucas[1]*, Janice L Atkins[1]*, Luke C Pilling[1], Jeremy Shearman[2], David Melzer[1]**

[1]Epidemiology and Public Health Group, Department of Clinical and Biomedical Sciences, Faculty of Health and Life Sciences, University of Exeter, UK

[2]Department of Gastroenterology, South Warwickshire University NHS Foundation Trust, UK

*Contributed equally to this study

Corresponding author: David Melzer, Professor of Epidemiology and Public Health, The Department of Clinical and Biomedical Sciences, Faculty of Health and Life Sciences, University of Exeter, St Luke's Campus, College House, Magdalen Road, Exeter, Devon, EX1 2LU, UK. E-mail: d.melzer@exeter.ac.uk

Keywords: Haemochromatosis; *HFE* p.C282Y / p.H63D genotypes; iron overload; morbidity; mortality; UK Biobank

Word count: 3,950

References: 43

Tables: 1

Figures: 4

For peer review only - http://bmjopen.bmj.com/site/about/guidelines.xhtml

For peer review only

Abstract

Objectives: *HFE* haemochromatosis genetic variants have an uncertain clinical penetrance, especially to older ages and in undiagnosed groups. We estimated p.C282Y and p.H63D variant cumulative incidence of multiple clinical outcomes in a large community cohort.

Design: Prospective cohort study.

Setting:22 assessment centres across England, Scotland, and Wales in the UK Biobank (2006-2010).

Participants:451,270 participants genetically similar to the 1000-Genomes European reference population, with a mean 13.3-year follow-up through hospital inpatient, cancer registries and death certificate data.

Main outcome measures: Cox proportional hazard ratios of incident clinical outcomes and mortality in those with *HFE* p.C282Y-p.H63D mutations compared to those with no variants, stratified by sex and adjusted for age, assessment centre and genetic stratification. Cumulative incidences were estimated from age 40 to 80 years.

Results:12.1% of p.C282Y+/+ males had baseline (mean age 57) haemochromatosis diagnoses, with age 80 cumulative incidence of 56.4%. 33.1% died vs. 25.4% without *HFE* variants (Hazard Ratio [HR] 1.29, 95% CI: 1.12-1.48, $p=4.7*10^{-4}$); 27.9% vs 17.1% had joint replacements, 20.3% vs 8.3% had liver disease, and there was excess delirium, dementia, and Parkinson's disease, but not depression. Associations, including excess mortality, were similar in the group undiagnosed with haemochromatosis. 3.4% of p.C282Y+/+ females had baseline haemochromatosis diagnoses, with cumulative age 80 incidence of 40.5%. There was excess incident liver disease (8.9% vs 6.8%; HR 1.62, 95% CI: 1.27-2.05, $p=7.8*10^{-5}$), joint replacements and delirium, with similar results in the undiagnosed. p.C282Y/p.H63D and p.H63D+/+ men or women had no statistically significant excess fatigue or depression at baseline and no excess incident outcomes.

Conclusions: Male and female p.C282Y homozygotes experienced greater excess morbidity than previously documented, including those undiagnosed with haemochromatosis in the community. As haemochromatosis diagnosis rates were low at baseline despite treatment being considered effective, trials of screening to identify people with p.C282Y homozygosity early appear justified.

For peer review only - http://bmjopen.bmj.com/site/about/guidelines.xhtml

Strengths and limitations of this study

- We analyzed largescale data on community volunteers from the UK Biobank, one of the world's largest *HFE* genotyped cohorts.
- We have analyzed incident disease outcomes during an extended follow-up period of mean 13.3 years.
- We have provided the first clinical outcome data to age 80 years in those with haemochromatosis genotypes, including those undiagnosed with haemochromatosis at baseline, expanding the life-course evidence on *HFE* penetrance.
- UK Biobank participants were somewhat healthier than the general population, but *HFE* allele frequencies were similar to previous UK studies.
- Incident outcomes were from hospital inpatient and cancer registry follow-up, so did not rely on potentially biased patient self-reporting, but community diagnosed conditions may be underestimated.

For peer review only

For peer review only - http://bmjopen.bmj.com/site/about/guidelines.xhtml

Introduction

HFE haemochromatosis is defined by iron overload[1] [2] due to gene variants, which dysregulate intestinal iron absorption. The p.C282Y+/+ (homozygous) group have markedly raised iron measures: e.g. median transferrin saturations were over 80% and near 60% in p.C282Y+/+ males and females in HEIRS study, but below 45% in compound heterozygotes (C282Y+/H36D+), and progressively lower across p.H63D+/+ (homozygote), p.C282Y+/- and p.H63D+/- carriers[3]. Women with each genotype have lower mean iron measures than men[4].

Clinical presentation of haemochromatosis is usually with fatigue, joint pain, raised iron measures or from family screening, or less commonly from direct-to-consumer genotyping. Symptoms usually present after age 40 years[5]. In severely affected p.C282Y+/+ patients (>90% of typical cases[6]), liver iron deposition can lead to liver fibrosis, cirrhosis and cancer[7], especially in the presence of other causes of liver disease. Arthropathy[8], diabetes[9], endocrine dysregulation[10], heart arrhythmias and cardiomyopathies[11], and pneumonia[9] have also been reported.

While clinical cohorts frequently have iron overload complications, disease penetrance in population genotyped groups is uncertain, especially at older ages and those not diagnosed with haemochromatosis[3] [12] [13]. Beutler et al found negligible haemochromatosis symptoms in 152 p.C282Y homozygotes from California health appraisal clinics (excluding diagnosed patients)[12] . The HEIRS Study reported excess liver disease in 299 p.C282Y+/+ males[14]. The Melbourne Collaborative Cohort Study[15] reported that 28.4% (95% Confidence Interval [95% CI], 18.8% to 40.2%) of p.C282Y+/+ males (n=95, mean age 65 at follow-up) had 'documented iron-overload-related disease', with 1.2% of p.C282Y+/+ females affected. Similarly, although excess mortality occurred in clinical patients (especially with liver disease)[16][17], no excess mortality was reported in community-identified p.C282Y+/+ males or other *HFE* genotype groups[15] [18]. Using UK Biobank, we previously examined data on European ancestry community participants with mean 7-year follow-up, finding the 1,294 male p.C282Y homozygotes had increased odds of liver disease and osteoarthritis compared to those without p.C282Y or p.H63D variants[9]. A UK Biobank 8.9-year follow-up quantified excess hepatic malignancies in male p.C282Y homozygotes (HR 10.5; 95% CI: 6.6-16.7; p<0.001 versus no *HFE* variant) and excess all-cause mortality (n=88 deaths; HR 1.2; 95% CI: 1.0-1.5; p=0.046)[7].

For p.C282Y/H63D compound heterozygotes, the Melbourne Collaborative Cohort Study[15] found only one male (of 242 studied) with documented iron overload-related disease, although alcohol was also a

For peer review only - http://bmjopen.bmj.com/site/about/guidelines.xhtml

factor. Similarly, a study of community identified participants with p.H63D variants from the Busselton study (Australia) found none with clinically significant iron overloading[19].

Given the accumulating evidence of significant clinical penetrance with p.C282Y homozygosity (only) and the reported effectiveness of treatment (predominantly venesection), there is renewed interest in screening for those at high risk[1] [20] [21]. *HFE* p.C282Y homozygosity is recommended for reporting to patients when found incidentally[22].

The UK Biobank community cohort includes baseline questionnaires, plus hospital inpatient diagnoses during the mean 13.3-year follow-up. The cohort therefore provides a potential model for community based genetic screening. Participant consent did not allow genotype feedback (see Methods), so outcomes reflect normal clinical care. Here we aimed to estimate risks and cumulative outcomes to age 80 years by genotype and sex for relevant clinical outcomes, including (for the first time) analyses of those undiagnosed with haemochromatosis at baseline.

Methods

Study population

UK Biobank includes community volunteers aged 39 to 73 years at baseline assessments across England, Scotland and Wales, from 2006 to 2010. Participants were somewhat healthier than the general population[23], but *HFE* allele frequencies were similar to previous UK studies[7]. Data cover 451,270 participants, genetically similar to the 1000 Genomes project European reference population[24], with *HFE* p.C282Y (rs1800562) and *HFE* p.H63D (rs1799945) genotypes from whole exome sequencing (Whole Exome Sequence methods were by Regeneron[25]). Participants gave written informed consent and were informed of relevant health related findings at baseline, but consent excluded individual notification of subsequent findings including genotypes. North West Multi-Centre Research Ethics Committee (Research Ethics Committee reference 11/NW/0382) approved UK Biobank. All research was conducted in accordance with both the Declarations of Helsinki and Istanbul.

Baseline variables and incident health outcomes

Baseline questionnaires covered doctor-diagnosed conditions including haemochromatosis. Symptom questions included: “Over the past two weeks, how often have you felt tired or had little energy?”, and responses were coded as ‘fatigue’ combining “more than half the days” and “nearly every day”. Studied

For peer review only - http://bmjopen.bmj.com/site/about/guidelines.xhtml

diagnoses were from *a priori* knowledge (see Supplementary eTable 1 for ascertainment codes for 34 outcomes). England, Wales, and Scotland hospital records were available from April 1996 to October 2022. Prevalent diagnoses were from baseline self-report plus hospital inpatient data from 1996 to baseline. Incident diagnoses and surgical procedures were from hospital inpatient data (baseline to October 2022) plus cancer registries to December 2020 for England and Wales and November 2021 for Scotland. National death records were available to November 2022. Disease ascertainment used International Classification of Diseases 10th revision (ICD-10) codes. Surgical procedures were from OPCS Classification of Interventions and Procedures version 4 (OPCS-4). Having 'any joint replacement surgery' included hip, knee, ankle, or shoulder replacement surgery. The 'any brain outcome' included delirium, dementia, or Parkinson's disease.

Statistical analysis

Cox proportional hazards regression estimated genotype associations with incident outcomes. Models were stratified by sex and adjusted for age, assessment centre and 10 genetic principal components (accounting for population substructure). Main outcome proportional hazards assumptions (tested using 'estat phtest') were met in models adjusted for 5-year age bands. Given the extensive prior evidence for risks in p.C282Y+/+ groups, multiple testing corrections are discussed for lower risk genotypes, using Bonferroni correction of $p<0.001$ (0.05 / 34 disease outcomes). Kaplan-Meier survivor functions estimated the probabilities of cumulative incidence for associated outcomes from age 40 to 80 years within 5-year bands, by *HFE* genotype and by sex. We applied observed incidence rates in each age group to a notional cohort, estimating hypothetical cumulative incident case numbers from age 40 to 80 years. Sensitivity analysis repeated main analyses excluding participants with a haemochromatosis diagnosis at baseline. All analyses were performed in Stata 17.0.

Patient and public involvement

Patients and participants were and are extensively involved in the UK Biobank study itself. We used anonymised data that were already collected and therefore no patients were involved in developing the research question or the outcomes tested. UK Biobank notified participants of relevant health related findings in the baseline assessment, but there is no individual notification of subsequent findings, including genotypes.

For peer review only - http://bmjopen.bmj.com/site/about/guidelines.xhtml
For peer review only

Results

UK Biobank baseline characteristics were previously reported[9] [26] (Table 1, eTables 2-3): 451,270 participants genetically similar to the European 1000 Genomes reference population were followed for a mean 13.3 years. There were 1,298 p.C282Y+/+ (homozygotes), 4,959 p.C282Y/p.H63D compound heterozygote, and 4,673 p.H63D+/+ males: for females 1,604, 5,760 and 5,580 respectively. In the male p.C282Y+/+ group, 12.1% had haemochromatosis diagnoses at baseline, with 6/1,000 diagnosed in p.C282Y/H63D and 2 per 1,000 in the p.H63D+/+ group: the respective rates in females were 3.4%, 2 per 1,000 and 4 per 10,000.

p.C282Y+/+ males aged 60 plus reported baseline excess fatigue (11.8% vs 8.2%; Odds Ratio (OR): 1.43, 95% CI: 1.11-1.85, p=0.01), but there was no statistically significant excess fatigue with other genotypes, except a marginal association in p.H63D+/- males (OR: 1.06, CI 1.00-1.12, p=0.05) which became non-significant after multiple testing correction. There were no differences in depression prevalence at baseline (Table 1, eTables 2-3).

Males with p.C282Y homozygote genotypes: excess mortality and morbidity versus without HFE variants

Figure 1 shows the Hazard ratios (HRs) for studied incident outcomes by sex and genotype, with cumulative incidence (with confidence intervals) to age 80 years presented in Figure 2 for those outcomes which had significant HRs. p.C282Y+/+ males had increased rates of mortality versus those without p.C282Y or p.H63D variants (HR 1.29, 95% CI: 1.12-1.48, $p=4.7*10^{-4}$; Figure 1; eTables 4 and 5). Cumulative incidence of death was 33.1% (95% CI: 28.9% to 37.8%) versus 25.4% (Figure 2 and 3a). Excess mortality in those undiagnosed with haemochromatosis at UK Biobank baseline (eTables 6 and 7) was similar (HR=1.22 95% CI: 1.05-1.43, $p=1.0*10^{-2}$) with a cumulative death rate of 32.5% (95% CI: 27.9 to 37.6%) (eFigure 1a).

Haemochromatosis diagnosis cumulative incidence at age 80 in p.C282Y+/+ males was 56.4% (Figure 2, eTable 8). Cumulative incidence of 'any liver disease' was 20.3% vs 8.3% without variants (HR 2.56, 95% CI: 2.10-3.12, $p=8.70*10^{-21}$) and 7.7% developed liver fibrosis or cirrhosis vs 1.3%. There was a raised HR for alcoholic liver disease (Figure 1) with cumulative incidence 3.3% of p.C282Y+/+ males vs 1.4%. Liver cancers cumulative incidence was 5.5% (95% CI: 3.8% to 8.0%, vs 0.8% without variants). p.C282Y+/+ males also had raised cumulative incidence of prostate cancer: 17.2% vs 14.8%. The cumulative incidence graphs show excess liver disease clearly apparent by age 55, but the excess mortality becoming significant at older ages (Figure 3a and 3b). Notably, 81.5% of deaths, 66.3% of 'any liver diseases', and 71.8% of joint replacements in p.C282Y+/+ males occurred after age 65.

For peer review only - http://bmjopen.bmj.com/site/about/guidelines.xhtml

Joint replacement cumulative incidence in p.C282Y+/+ males was 27.9% vs 17.1% without variants (Figure 3c): this remained after excluding those with fractures within 5 days before surgery (n=53; HR 1.74, 95% CI: 1.45-2.09; p=1.6*10^{-9}). p.C282Y+/+ males also had excess osteoarthritis, fragility fractures, and osteoporosis.

Brain outcome (dementia, delirium, or Parkinson's disease) cumulative incidence in p.C282Y+/+ males was 16.3% versus 10.0% without variants (HR 1.65 95% CI: 1.31-2.06, p=1.70*10^{-5}) (Figure 3d); for delirium 12.4% vs. 5.8% (HR 1.69, 95% CI: 1.26-2.27; p=4.80*10^{-4}); non-Alzheimer's dementia 6.0% vs 3.0% (HR 2.05, 95% CI: 1.40-3.00; p=2.40*10^{-4}), and Parkinson's disease (HR 1.86, 95% CI: 1.21-2.87 p=0.005).

Diabetes (type 1 or 2) cumulative incidence was 23.5% in p.C282Y+/+ males (vs. 19.1%). p.C282Y+/+ males had excess hospital diagnosed COVID-19 (8% vs 4.8%, HR 1.51, 95% CI: 1.08-2.11; p=0.02, missing significance in earlier follow-up data[27]), urinary tract infections (UTI) and skin and soft tissue infections (SSTI). Associations were not significant for cardiac outcomes (arrhythmia, cardiomyopathy, CHD, heart failure) or depression.

Estimates of excess morbidity in the male p.C282Y+/+ group without haemochromatosis diagnoses in the community at UK Biobank baseline (n=1,141) were mostly similar to those in the overall sample (Figures 2 and eFigure 1a-d, eTables 6 and 7). Notably, however, point HR estimates for liver fibrosis or cirrhosis, and liver cancers risk appeared marginally lower than in the whole group, but with wide confidence intervals including the whole study estimates HR point estimates (e.g. for liver fibrosis and cirrhosis HR 4.52, 95% CI: 3.07-6.66, p=2.10*10^{-14} without diagnosis, versus HR 5.36 95% CI: 3.83-7.52; p=2.00*10^{-22} in the whole p.C282Y+/+ group). In addition, alcoholic liver disease and osteoporosis lost statistical significance, but cholecystitis became nominally statistically significant (HR 1.54, 95% CI: 1.02-2.32, p=0.04).

Females with p.C282Y homozygote genotypes: excess morbidity versus without HFE variants

Diagnosed haemochromatosis cumulative incidence in p.C282Y+/+ females was 40.5% by age 80. There was no excess mortality, but this group experienced excess 'any liver disease' (8.9% vs 6.75%; HR 1.62, 95% CI: 1.27-2.05; p=7.80*10^{-5}) (Figure 4a), liver fibrosis or cirrhosis (1.9% vs 0.8%; HR 2.56, 95% CI: 1.50-4.36; p=0.001), alcoholic liver disease (1.0% vs 0.3%; HR 3.07, 95% CI: 1.44-6.54; p=0.004), plus cholecystitis, versus women without *HFE* variants (Figures 1 and 2; eTables 8, 9 and 10).

Joint replacement surgery was more common in female p.C282Y homozygotes (23.2% vs 21.1%) (Figure 4b), as were osteoarthritis and osteoporosis. Cumulative incidence of brain outcomes was raised (8.6% vs

For peer review only - http://bmjopen.bmj.com/site/about/guidelines.xhtml

7.4%; HR 1.30, 95% CI: 1.01-1.68; p=0.04), including delirium (5.9% vs 3.9%; HR 1.50. 95% CI: 1.08-2.08; p=0.02).

There was a nominally significant excess of heart failure (HR 1.34, 95% CI:1.03-1.75, p=0.03) but no associations with other studied cardiac outcomes. There were no associations with liver cancer, fragility fractures, diabetes, dementia, Parkinson's disease, or infections (SSTIs, UTIs).

Estimates of excess morbidity in the female p.C282Y+/+ group without haemochromatosis diagnoses in the community at UK Biobank baseline (n=1,550) were similar to those in the overall sample (Figures 2 and eFigure 2a-b, eTables 11 and 12).

Male and female p.C282Y/p.H63D compound heterozygotes versus without HFE variants

Haemochromatosis diagnosis cumulative incidence in p.C282Y/H63D males was 5.4% by age 80. However, there was no statistically significant increase in mortality, incidence of 'any liver disease' or liver cancers, joint replacements, or diabetes (Figure 1). p.C282Y/H63D males were modestly more likely to develop SSTIs (HR 1.16, 95% CI: 1.01-1.33, p=0.03) but this lost statistical significance after multiple testing correction.

Haemochromatosis cumulative incidence in p.C282Y/H63D females was 2.7% by age 80 but there were similarly no excess mortality, musculoskeletal diagnoses, diabetes, or brain outcomes (Figure 1). However, there was a modest association with 'any liver disease' (HR 1.19, 95% CI: 1.02-1.38, p=0.03), but associations with specific liver diagnoses were non-significant, including for liver fibrosis and cirrhosis, the most common form of liver disease with iron overload (Figures 1-2; eTables 4, 5, 8, 9, 10).

Males and females with p.H63D homozygote genotypes vs. no HFE variants

Diagnosed haemochromatosis cumulative incidence in p.H63D/H63D males was 1.9% by age 80, but with no excess mortality, liver disease diagnoses, liver cancers, joint replacement surgery, diabetes, or depression. There were protective associations between p.H63D homozygosity and fragility fractures (HR 0.78 95% CI: 0.62-0.98 p=0.03) and osteoporosis (HR 0.73 95% CI: 0.53-0.99, p=0.04) (eTables 4, 5 and 8).

Diagnosed haemochromatosis cumulative incidence p.H63D/H63D females was 0.6% by age 80. There was no excess mortality, liver disease or liver cancers, joint replacement surgery, diabetes, or depression. However, females also had a nominally significant (uncorrected $p<0.05$) protective association with fragility fractures (HR 0.87 95% CI: 0.76-1.00 p=0.05), echoing the similar protective associations in p.H63D/H63D males (eTables 8, 9 and 10).

For peer review only - http://bmjopen.bmj.com/site/about/guidelines.xhtml

Associations for p.C282Y+/- and p.H63D+/- (heterozygote carrier) males and females

There were a small number of nominally significant protective and risk associations in carrier groups (eTables 4 and 5), in p.C282Y+/- males including for excess delirium (HR 1.13 95% CI: 1.04-1.24 p=0.006) and skin and soft tissue infections (HR 1.14 95% CI: 1.06-1.22 p=1.80*10^{-4}) and for p.H63D+/- males a protective association with fragility fractures (HR 0.92 95% CI: 0.86-0.99 p=0.03). For p.C282Y+/- females there were no associations, but for p.H63D+/- females a nominally significant excess of liver cancers (HR 1.26 95% CI: 1.01-1.58 p=0.04) was present, plus a protective association for osteoporosis (eTables 9 and 10). Only the skin and soft tissue infection association was significant after multiple testing correction.

Discussion

Clinical penetrance by *HFE* genotypes in community samples has been uncertain, especially for those undiagnosed for haemochromatosis, the potential beneficiaries of population screening. Our results show greater excess morbidity and mortality in p.C282Y+/+ groups than previously documented, much occurring at older ages. p.C282Y+/+ males had marked excess mortality (33.1% dead by age 80 vs. 25.4% without p.C282Y or p.H63D variants), plus substantial excess joint replacements, liver disease, brain outcomes and some infections. Estimates of excess morbidity in p.C282Y+/+ groups undiagnosed for haemochromatosis were similar (eTables 7 and 12): perhaps most crucially, the p.C282Y homozygote male undiagnosed group had similar excess mortality, with an estimated cumulative death rate to age 80 of 32.5% (95% CI: 27.9 to 37.6%) versus 25.4% in those without *HFE* variants (eFigure 1a and eTable 7). Of p.C282Y+/+ females (n=1,604), diagnosed haemochromatosis cumulative incidence by 80 was 40.5%, with excess 'any liver disease', joint replacements and delirium present, suggesting more clinical penetrance than previously documented. In p.C282Y/p.H63D and H63D homozygote groups and carriers, we found no excess fatigue or depression at baseline and no excess mortality or major outcomes during follow-up.

Comparison to previous fatigue and depression studies

Baseline characteristics in UK Biobank have been reported before,[26], except for fatigue and depression. Fatigue and depression in haemochromatosis is important to patients and their carers, but the causal role of iron overload in these symptoms is unclear. p.C282Y+/+ males aged 60 plus reported excess fatigue (11.8% versus 8.2% without *HFE* variants) at baseline, but no excess depression at baseline or follow-up. Rates of both conditions were similar in the other studied genotype groups to those without *HFE* variants (except in p.H63D+/- males, with very modest excess fatigue, non-significant with multiple statistical testing correction). A blinded randomized trial of erythrocytapheresis[28] in p.C282Y+/+ groups with moderately raised ferritin levels (300 and 1000 µg/L) did find reduced fatigue scores with transferrin

For peer review only - http://bmjopen.bmj.com/site/about/guidelines.xhtml

saturations falling from mean 63.5% to 45.4% in the treatment group, although the mean fatigue score was below diagnosis level, making clinical interpretation unclear. Also, recent observational studies have cast doubt on relationships between fatigue, quality of life measures and iron parameters in haemochromatosis patients[29] [30], and fatigue might also occur with venesection. A UK biobank analysis found tiredness genetically linked to factors including increased adiposity, blood lipids and inflammatory markers[31].

Comparison to previous mortality-and morbidity studies

Rates of haemochromatosis diagnosis at baseline (mean age 57) were low even in p.C282Y+/+ groups (12.1% in males, 3.4% in females), but cumulative estimates to age 80 indicated much higher diagnosis rates at older ages. These baseline rates are similar to cumulative rates from the US hospitals genetics collaboration eMERGE study[32] for p.C282Y+/+ group at age 60. The eMERGE study[32] figures also show the majority of haemochromatosis diagnoses occurring later in life, reaching nearly 50% in males and 25% in females after age 80.

Earlier reports suggested no excess mortality in community-identified p.C282Y+/+[15] [18], despite higher death rates in clinical cohorts with liver disease[33], [15]. In the UK Biobank much larger sample, we found clearly increased all-cause mortality in p.C282Y+/+ males only (194 p.C282Y+/+ deaths, HR 1.29, 95% CI: 1.12 to 1.48, $p=4.70*10^{-4}$). A Swedish sample of 2,273 p.C282Y homozygotes found similar increased mortality (HR 1.30, 95% CI: 95%: 1.12-1.50, versus males with no mutations), with no excess in female homozygotes (HR 0.98, 95% CI: 0.82-1.18)[34]. These studies found no excess mortality in p.C282Y/p.H63D or p.H63D/p.H63D groups.

Estimates of disease penetrance in community genotyped groups differ widely [3][12] [13], with the highest estimate from The Melbourne Collaborative Cohort Study[15] , of 28.4% (95% CI: 18.8% to 40.2%) of p.C282Y+/+ males (n=95, mean age 65 at follow-up) having 'documented iron-overload-related disease', and 1.2% of female p.C282Y homozygotes affected. In UK Biobank we found that diagnosed haemochromatosis cumulative incidence was 36.1% in p.C282Y+/+ males and 21.2% of p.C282Y+/+ females by age 65 (eTable 8), suggesting a larger burden of iron overload disease especially in p.C282Y+/+ women. Liver cancers occurred in 5.5% (95% CI: 3.8% to 8.0%) of p.C282Y+/+ males by age 80, a slightly lower estimate than at age 75[7] (7.2% (95% CI: 3.9%-13.1%), but within the earlier confidence intervals. The estimate remains comparable to a meta-analysis[13] estimated lifetime incidence of severe liver disease (cirrhosis or hepatocellular carcinoma) of 9% (95% CI: 2.6%-15.3%) in untreated male *HFE* p.C282Y+/+ homozygotes.

For peer review only - http://bmjopen.bmj.com/site/about/guidelines.xhtml

Several studies have reported excess arthritis in p.C282Y homozygote men and to a lesser extent, in female homozygotes[9]. Our results extend a previous 11.5-year follow-up[35], now showing the full extent of later-life joint replacements in male p.C282Y homozygotes and providing a robust measure for severe joint damage in haemochromatosis.

Excess diabetes has previously been reported in p.C282Y homozygous men (9), but other studies have suggested that *HFE* mutations do not have important pathophysiological consequences in patients with type 2 diabetes[36]. We found a modest diabetes excess in p.C282Y+/+ individuals (Figures 1 and 2). Overall, as cumulative diabetes incidence was 23.5% in p.C282Y+/+ males vs. 19.1% without *HFE* variants, non-iron factors appear to now contribute the majority of diabetes even in p.C282Y+/+ males. Although cardiac complications are often noted haemochromatosis reviews[11][37], there is little evidence linking community-identified p.C282Y homozygosity to these outcomes[38] [39]: our finding of excess of heart failure in p.C282Y homozygote women only (HR 1.34 95% CI: 1.03-1.75, p=0.03) needs further study.

Our previous UK Biobank report on brain outcomes (mean 10.5 years follow-up) found p.C282Y+/+ males had excess dementia diagnoses and iron deposition in key brain areas on MRI[40]. The current analysis also found higher incidence especially of non-Alzheimer's dementia in p.C282Y+/+ males plus more delirium. Loughnan et al[41] reported that p.C282Y+/+ males had more Parkinson's disease (OR 1.83; 95% CI: 1.19-2.80; p=0.006) in cross-sectional analyses of UK Biobank: our incident analyses provide a similar estimate for Parkinson's disease in p.C282Y+/+ males (HR 1.86, 95% CI: 1.21-2.87 p=0.005).

In the HEIRS study[3] community genotyped p.C282Y/p.H63D (compound heterozygote) males and females had substantially lower transferrin saturation levels compared to p.C282Y+/+ males or females. In line with this, cumulative incidence of haemochromatosis diagnosed by age 80 was relatively low (men 5.4%, women 2.7%), and no excess mortality, liver fibrosis and cirrhosis, joint replacements, diabetes, or depression in these groups. We did find that p.C282Y/p.H63D females had nominally significant small excess of any liver disease (HR 1.19, 95% CI: 1.02-1.38; p=0.03) although with no excess liver fibrosis and cirrhosis, no similar association in p.C282Y/p.H63D males, and with a non-significant multiple testing p-value. p.C282Y/p.H63D males had a small excess of skin and soft tissue infections (HR 1.16, 95% CI: 1.01-1.33, uncorrected p=0.03), again multiple testing non-significant.

Strengths and limitations

We analyzed largescale cohort data on community volunteers, providing the first outcome data to age 80. *HFE* allele frequencies were similar to other UK studies[7] but UK Biobank did recruit somewhat healthier

For peer review only - http://bmjopen.bmj.com/site/about/guidelines.xhtml

participants than the general population[23], so we have focused on incident outcomes during the mean 13.3 year follow-up: we found no deviations from proportional hazards assumptions in Cox models. Outcomes for both the overall genotype groups and those undiagnosed with haemochromatosis at UK Biobank baseline are presented. Previous studies have suggested that treated p.C282Y homozygous patients do not have an increased mortality rate compared to the general population [42], but numbers with haemochromatosis diagnoses at baseline were small in the current study and so outcomes in this group were not presented. Also, there was no data available on the diverse routes to diagnosis, so modelling treated outcomes would likely be confounded. Further work is needed on the effect so early diagnosis and treatment. Most outcomes were from hospital inpatient care, so community diagnosed conditions may be underestimated, but findings are similar to analyses including primary care records (available to 2017 only)[7] and baseline data collected at interview[9]. Information on the criteria used to establish a diagnosis of fibrosis/cirrhosis in the hospital follow-up data (e.g. non-invasive biomarker panels or liver biopsy) were not available.

Implications for early diagnosis and screening

We found greater excess morbidity than previously reported in both p.C282Y+/+ males and females, especially at older ages, and in those undiagnosed with haemochromatosis. Despite treatment (predominantly venesection) being considered effective for preventing liver disease progression[1][43][44] rates of haemochromatosis diagnosis were low at baseline: for example, of 31 p.C282Y+/+ males with incident liver cancer, 17 were undiagnosed with haemochromatosis at baseline. Genotyping earlier in life could be a powerful preventive tool to identify p.C282Y+/+ individuals who are clearly at major risk of related excess disease. A recent cross-sectional genotyping study within a US health provider found 72% (144/201) of p.C282Y homozygous patients (mean age 62) had not been diagnosed with haemochromatosis, 36% of whom had iron overload [21]. As cumulative diagnosed haemochromatosis in UK Biobank cohort was estimated 56.4% in p.C282Y+/+ males and 40.5% females by age 80, p.C282Y+/+ genotyping with follow-up iron studies would likely have a high yield. However, the prevention potential following up p.C282Y/p.H63D, H63D+/+ groups will likely be very low, as there was no significant excess fatigue or depression at baseline, and no excess mortality, incident liver fibrosis or cirrhosis, joint replacements, or depression.

Conclusion

Male and female p.C282Y homozygotes in this community cohort experienced greater excess morbidity than previously documented. Much of this excess liver, musculoskeletal, diabetes, brain and morbidity

For peer review only - http://bmjopen.bmj.com/site/about/guidelines.xhtml

occurred after age 60. In those not diagnosed with haemochromatosis at study baseline, risks were broadly similar to those in the overall group, notably including excess mortality in p.C282Y+/+ males. Trials of targeted or community genotyping to diagnose haemochromatosis earlier appear justified, especially to identify people with the p.C282Y homozygote variants. The potential for iron related preventive treatment in the p.C282Y/H63D, p.H63D+/+ and other *HFE* genotype groups appears very limited, as these genotype groups were associated with no statistically significant excess morbidity or mortality.

Data sharing

Data are available on application to the UK Biobank (www.ukbiobank.ac.uk/register-apply).

Funding

University of Exeter supports ML, LP and DM; JA has a National Institute for Health and Care Research (NIHR) Advanced Fellowship (NIHR301844).

Authors contributions

ML performed the analysis, interpreted results, created the figures, and drafted the manuscript. JA contributed to the design and analysis of the study, data interpretation and drafting of the manuscript. LP contributed to the design of the study, data interpretation, creation of figures and contributed to the manuscript. JS provided expert clinical interpretation of the data and contributed to the manuscript. DM oversaw design of the study, data analysis, interpretation of results, and led the writing of the manuscript. All authors approved the final version of the manuscript.

Acknowledgements

This research was conducted using the UK Biobank resource, under application 14631. We thank the UK Biobank participants and coordinators. This work used data provided by patients and collected by the NHS as part of their care and support. Copyright © (2023), NHS England. Re-used with the permission of the NHS England and UK Biobank. All rights reserved. This research also used data assets made available by National Safe Haven as part of the Data and Connectivity National Core Study, led by Health Data Research UK in partnership with the Office for National Statistics and funded by UK Research and Innovation. This study was supported by the National Institute for Health and Care Research Exeter Biomedical Research Centre. The views expressed are those of the authors and not necessarily those of the NIHR or the Department of Health and Social Care.

For peer review only - http://bmjopen.bmj.com/site/about/guidelines.xhtml

Competing interests

None declared.

For peer review only

For peer review only - http://bmjopen.bmj.com/site/about/guidelines.xhtml

Table 1. Baseline characteristics of male and female UK Biobank participants by selected p.C282Y/H63D genotypes

	**No** p.C282Y or p.H63D **variants**	**H63D +/+**	**C282Y +/ H36D +**	**C282Y +/+**
**MALES**				
Total participants	122,841	4,673	4,959	1,298
Mean age, years (SD)	56.99 (8.1)	56.99 (8.1)	56.97 (8.1)	56.84 (8.2)
Hemochromatosis diagnosis, n (%)	29 (0.02)	8 (0.2)	29 (0.6)	157 (12.1)
Self-reported fatigue, n (%)	12,094 (10.1)	451 (9.9)	523 (10.9)	148 (11.8)
Self-reported fatigue (60+ years), n (%),	4,475 (8.2)	173 (8.2)	177 (8.0)	68 (11.8)
Prevalent depression, n (%)	5,883 (4.8)	220 (4.7)	188 (3.8)	71 (5.5)
**FEMALES**				
Total participants	145,694	5,580	5,760	1,604
Mean age, years (SD)	56.62 (7.9)	56.58 (8.1)	56.46 (7.9)	56.92 (8.0)
Hemochromatosis diagnosis, n (%)	8 (0.01)	2 (0.04)	12 (0.2)	54 (3.4)
Self-reported fatigue, n (%)	19,110 (13.5)	760 (14.1)	785 (14.1)	220 (14.4)
Self-reported fatigue (60+ years), n (%),	6,055 (10.0)	253 (10.9)	229 (9.9)	77 (11.4)
Prevalent depression, n (%)	10,734 (7.4)	387 (6.9)	432 (7.5)	123 (7.7)

A total of 451,270 male and female participants genetically-similar to the 1000 Genomes project European reference population with *HFE* genotypic data available in the UK Biobank. Numbers presented are mean (SD) for continuous variables and n (%) for categorical variables. Fatigue = tiredness in more than half the days in past 2 weeks. See supplementary eTable 2 and 3 for all p.C282Y/H63D genotype groups.

For peer review only - http://bmjopen.bmj.com/site/about/guidelines.xhtml

Figure 1. Hazard ratios of incident disease outcomes (95% CI) in selected p.C282Y/H63D genotypes compared to those with no mutations. Hazard ratios compared to those with neither *HFE* mutation. Cox proportional hazards regression models adjusted for age, assessment centre, and genetic principal components 1–10. Abbreviations: UTI, urinary tract infection; SSTI, skin and soft tissue infection; CI, confidence interval. Joint replacement surgery includes a diagnosis of hip, knee, ankle, or shoulder replacement. Any brain outcome included a diagnosis of delirium, dementia, or Parkinson's disease. See supplementary eTables 4, 5, 9 and 10 for incident numbers and HRs for all p.C282Y/H63D genotype groups.

Figure 2. Cumulative incidence of outcomes from ages 40-80 years by *HFE* genotypes (no p.C282Y or p.H63D variants vs p.C282Y homozygotes and p.C282Y homozygotes undiagnosed with hemochromatosis at baseline). Cumulative incidence (estimated % diagnosed by age 80 years; 95% CIs) for significant outcomes ($p<0.05$) from Cox proportional hazards regression models (Figure 1; eTables 5, 7, 10 and 12). Abbreviations: UTI, urinary tract infection; SSTI, skin and soft tissue infection; CI, confidence interval. Joint replacement surgery includes a diagnosis of hip, knee, ankle, or shoulder replacement. Any brain outcome included a diagnosis of delirium, dementia, or Parkinson's disease.

Figure 3. Kaplan-Meier curves for the cumulative incidence of (a) mortality, (b) liver disease, (c) any joint replacement, and (d) any brain outcome, in male *HFE* p.C282Y homozygotes compared to those with no mutations. Cumulative incidence (estimated % diagnosed by age 80 years; 95% CIs). Joint replacement surgery includes a diagnosis of hip, knee, ankle, or shoulder replacement. Any brain outcome included a diagnosis of delirium, dementia, or Parkinson's disease.

Figure 4. Kaplan-Meier curves for the cumulative incidence of (a) liver disease, and (b) any joint replacement, in female *HFE* p.C282Y homozygotes compared to those with no mutations. Cumulative incidence (estimated % diagnosed by age 80 years; 95% CIs). Joint replacement surgery includes a diagnosis of hip, knee, ankle, or shoulder replacement.

For peer review only - http://bmjopen.bmj.com/site/about/guidelines.xhtml

References

1. Adams PC, Jeffrey G, Ryan J. Haemochromatosis. Lancet. 2023;1811–21.
2. Katsarou MS, Papasavva M, Latsi R, Drakoulis N. Hemochromatosis: Hereditary hemochromatosis and HFE gene. Vitam Horm. 2019 Jan 1;110:201–22.
3. Adams PC, Reboussin DM, Barton JC, McLaren CE, Eckfeldt JH, McLaren GD, et al. Hemochromatosis and iron-overload screening in a racially diverse population. N Engl J Med. 2005 Apr 28;352(17):1769–78.
4. Bomford A. Genetics of haemochromatosis. Lancet. 2002 Nov 23;360(9346):1673–81.
5. Adams P. Classic Hereditary Hemochromatosis [Internet]. National Organization for Rare Disorders. 2019. p. 1–3. Available from: https://rarediseases.org/rare-diseases/classic-hereditary-hemochromatosis/
6. Adams PC. Epidemiology and diagnostic testing for hemochromatosis and iron overload. Int J Lab Hematol. 2015;37(S1):25–30.
7. Atkins JL, Pilling LC, Masoli JAH, Kuo CL, Shearman JD, Adams PC, et al. Association of Hemochromatosis HFE p.C282Y Homozygosity with Hepatic Malignancy. JAMA - Journal of the American Medical Association. 2020 Nov 24;324(20):2048–57.
8. Wijarnpreecha K, Aby ES, Panjawatanan P, Kroner PT, Harnois DM, Palmer WC, et al. Hereditary hemochromatosis and risk of joint replacement surgery: A systematic review and meta-analysis. Eur J Gastroenterol Hepatol. 2020;33(1):96–101.
9. Pilling LC, Tamosauskaite J, Jones G, Wood AR, Jones L, Kuo C ling L, et al. Common conditions associated with hereditary haemochromatosis genetic variants: cohort study in UK Biobank. BMJ. 2019 Jan 16;364:k5222.
10. Pelusi C, Gasparini DI, Bianchi N, Pasquali R. Endocrine dysfunction in hereditary hemochromatosis. Vol. 39, Journal of Endocrinological Investigation. Springer International Publishing; 2016. p. 837–47.
11. Joshi PK, Patel SC, Shreya D, Zamora DI, Patel GS, Rodriguez K, et al. Hereditary Hemochromatosis : A Cardiac Perspective. 2021;13(11).
12. Beutler E, Felitti VJ, Koziol JA, Ho NJ, Gelbart T. Penetrance of 845G → A (C282Y) HFE hereditary haemochromatosis mutation in the USA. Lancet. 2002;359(9302):211–8.
13. Grosse SD, Gurrin LC, Bertalli NA, Allen KJ. Clinical penetrance in hereditary hemochromatosis: estimates of the cumulative incidence of severe liver disease among HFE C282Y homozygotes. Genetics in Medicine. 2017;
14. McLaren CE, Barton JC, Adams PC, Harris EL, Acton RT, Press N, et al. Hemochromatosis and Iron Overload Screening (HEIRS) study design for an evaluation of 100,000 primary care-based adults. Am J Med Sci. 2003 Feb;325(2):53–62.
15. Allen KJ, Gurrin LC, Constantine CC, Osborne NJ, Delatycki MB, Nicoll AJ, et al. Iron-Overload–Related Disease in *HFE* Hereditary Hemochromatosis. New England Journal of Medicine. 2008 Jan 17;358(3):221–30.

For peer review only - http://bmjopen.bmj.com/site/about/guidelines.xhtml

16. Niederau C, Fischer R, Purschel A, Purschel P, Stremmel W, Ha¨ussinger D, et al. Long-term Survival in Patients With Hereditary Hemochromatosis. Vol. 110, Gastroenterology. W.B. Saunders; 1996 Apr.

17. Adams PC, Richard L, Weir M, Speechley M. Survival and development of health conditions after iron depletion therapy in C282Y-linked hemochromatosis patients. Canadian Liver Journal. 2021;4(4):381–90.

18. Van Aken MO, De Craen AJM, Gussekloo J, Moghaddam PH, Vandenbroucke JP, Heijmans BT, et al. No increase in mortality and morbidity among carriers of the C282Y mutation of the hereditary haemochromatosis gene in the oldest old: The Leiden 85-plus Study. Eur J Clin Invest. 2002;32(10):750–4.

19. Gochee PA, Powell LW, Cullen DJ, Du Sart D, Rossi E, Olynyk JK. A population-based study of the biochemical and clinical expression of the H63D hemochromatosis mutation. Gastroenterology. 2002 Mar;122(3):646–51.

20. Schmidtke J. Twenty-Five Years of Contemplating Genotype-Based Hereditary Hemochromatosis Population Screening. Genes (Basel). 2022;13(9):1622.

21. Savatt JM, Johns A, Schwartz MLB, McDonald WS, Salvati ZM, Oritz NM, et al. Testing and Management of Iron Overload After Genetic Screening–Identified Hemochromatosis. JAMA Netw Open. 2023 Oct 23;6(10):e2338995.

22. Miller DT, Lee K, Chung WK, Gordon AS, Herman GE, Klein TE, et al. ACMG SF v3.0 list for reporting of secondary findings in clinical exome and genome sequencing: a policy statement of the American College of Medical Genetics and Genomics (ACMG). Genetics in Medicine. 2021;23(8):1381–90.

23. Fry A, Littlejohns TJ, Sudlow C, Doherty N, Adamska L, Sprosen T, et al. Comparison of Sociodemographic and Health-Related Characteristics of UK Biobank Participants With Those of the General Population. Am J Epidemiol. 2017 Nov 1;186(9):1026–34.

24. National Academies of Sciences, Engineering, and Medicine, [Internet]. Committee on the Use of Race, Ethnicity, and Ancestry as Population Descriptors in Genomics Research, Board on Health Sciences Policy, Committee on Population, Health and Medicine Division, Division of Behavioral and Social Sciences and Education, Nationa. National Academies Press, Washington, D.C; 2023. Available from: https://www.nap.edu/catalog/26902

25. Van Hout C V., Tachmazidou I, Backman JD, Hoffman JD, Liu D, Pandey AK, et al. Exome sequencing and characterization of 49,960 individuals in the UK Biobank. Nature. 2020;586(7831):749–56.

26. Tamosauskaite J, Atkins JL, Pilling LC, Kuo CL, Kuchel GA, Ferrucci L, et al. Hereditary Hemochromatosis Associations with Frailty, Sarcopenia and Chronic Pain: Evidence from 200,975 Older UK Biobank Participants. J Gerontol A Biol Sci Med Sci. 2019 Jan 15;74(3):337–42.

27. Atkins JL, Lucas M, Pilling LC, Melzer D. Letter to the editor regarding: “A haemochromatosis-causing HFE mutation is associated with SARS-CoV-2 susceptibility in the Czech population”. Clinica Chimica Acta 538 (2023) 211–215. Clinica Chimica Acta. 2023;542(February):117271.

For peer review only - http://bmjopen.bmj.com/site/about/guidelines.xhtml

28. Ong SY, Gurrin LC, Dolling L, Dixon J, Nicoll AJ, Wolthuizen M, et al. Reduction of body iron in HFE-related haemochromatosis and moderate iron overload (Mi-Iron): a multicentre, participant-blinded, randomised controlled trial. Lancet Haematol. 2017 Dec 1;4(12):e607–14.

29. Acevedo LAU, Alvarenga AM, Fonseca PFS, da Silva NK, Cançado RD, Naoum FA, et al. Quality of Life Scores Remained Different among the Genotypic Groups of Patients with Suspected Hemochromatosis, Even after Treatment Period. Genes (Basel). 2022 Jan 1;13(1).

30. Belhomme N, Morcet J, Lescoat A, Robin F, Deugnier Y, Bardou-Jacquet E, et al. Patient-reported outcomes and their relation with iron parameters in HFE haemochromatosis during maintenance therapy: A prospective cohort study. Liver Int. 2022 Jun 30;

31. Deary V, Hagenaars SP, Harris SE, Hill WD, Davies G, Liewald DCM, et al. Genetic contributions to self-reported tiredness. Mol Psychiatry. 2018 Mar;23(3):609–20.

32. Gallego CJ, Burt A, Sundaresan AS, Ye Z, Shaw C, Crosslin DR, et al. Penetrance of Hemochromatosis in HFE Genotypes Resulting in p.Cys282Tyr and p.[Cys282Tyr];[His63Asp] in the eMERGE Network. Am J Hum Genet. 2015 Oct 1;97(4):512–20.

33. Adams PC, Speechley M, Kertesz AE. Long-term survival analysis in hereditary hemochromatosis. Gastroenterology. 1991;101(2):368–72.

34. Hagström H, Ndegwa N, Jalmeus M, Ekstedt M, Posserud I, Rorsman F, et al. Morbidity, risk of cancer and mortality in 3645 HFE mutations carriers. Liver Int. 2021 Mar 1;41(3):545–53.

35. Banfield LR, Knapp KM, Pilling LC, Melzer D, Atkins JL. Hemochromatosis Genetic Variants and Musculoskeletal Outcomes: 11.5-Year Follow-Up in the UK Biobank Cohort Study. JBMR Plus. 2023;00(00):1–11.

36. Davis TME, Beilby J, Davis WA, Olynyk JK, Jeffrey GP, Rossi E, et al. Prevalence, Characteristics, and Prognostic Significance of *HFE* Gene Mutations in Type 2 Diabetes. Diabetes Care. 2008 Sep 1;31(9):1795–801.

37. Kowdley K V., Brown KE, Ahn J, Sundaram V. ACG Clinical Guideline. Am J Gastroenterol. 2019;114(August):1.

38. Fox CJ, Cullen DJ, Knuiman MW, Cumpston GN, Divitini ML, Rossi E, et al. Effects of body iron stores and haemochromatosis genotypes on coronary heart disease outcomes in the Busselton health study. J Cardiovasc Risk. 2002 Oct;9(5):287–93.

39. Waalen J, Felitti V, Gelbart T, Ho NJ, Beutler E. Prevalence of coronary heart disease associated with HFE mutations in adults attending a health appraisal center. Am J Med. 2002 Oct;113(6):472–9.

40. Atkins JL, Pilling LC, Heales CJ, Savage S, Kuo CL, Kuchel GA, et al. Hemochromatosis mutations, brain iron imaging, and dementia in the UK Biobank cohort. Journal of Alzheimer's Disease. 2021;79(3):1203–11.

41. Loughnan R, Ahern J, Tompkins C, Palmer CE, Iversen J, Thompson WK, et al. Association of Genetic Variant Linked to Hemochromatosis With Brain Magnetic Resonance Imaging Measures of Iron and Movement Disorders. 2022;74136:1–10.

For peer review only

For peer review only - http://bmjopen.bmj.com/site/about/guidelines.xhtml

42. Bardou-Jacquet E, Morcet J, Manet G, Lainé F, Perrin M, Jouanolle AM, et al. Decreased cardiovascular and extrahepatic cancer-related mortality in treated patients with mild HFE hemochromatosis. J Hepatol. 2015 Mar;62(3):682–9.

43. Bardou-Jacquet E, Morandeau E, Anderson GJ, Ramm GA, Ramm LE, Morcet J, et al. Regression of Fibrosis Stage With Treatment Reduces Long-Term Risk of Liver Cancer in Patients With Hemochromatosis Caused by Mutation in HFE. Clin Gastroenterol Hepatol. 2020 Jul;18(8):1851–7.

44. Powell LW, Dixon JL, Ramm GA, Purdie DM, Lincoln DJ, Anderson GJ, et al. Screening for hemochromatosis in asymptomatic subjects with or without a family history. Arch Intern Med. 2006 Feb 13;166(3):294–301.

For peer review only

For peer review only - http://bmjopen.bmj.com/site/about/guidelines.xhtml

For peer review only

For peer review only - http://bmjopen.bmj.com/site/about/guidelines.xhtml

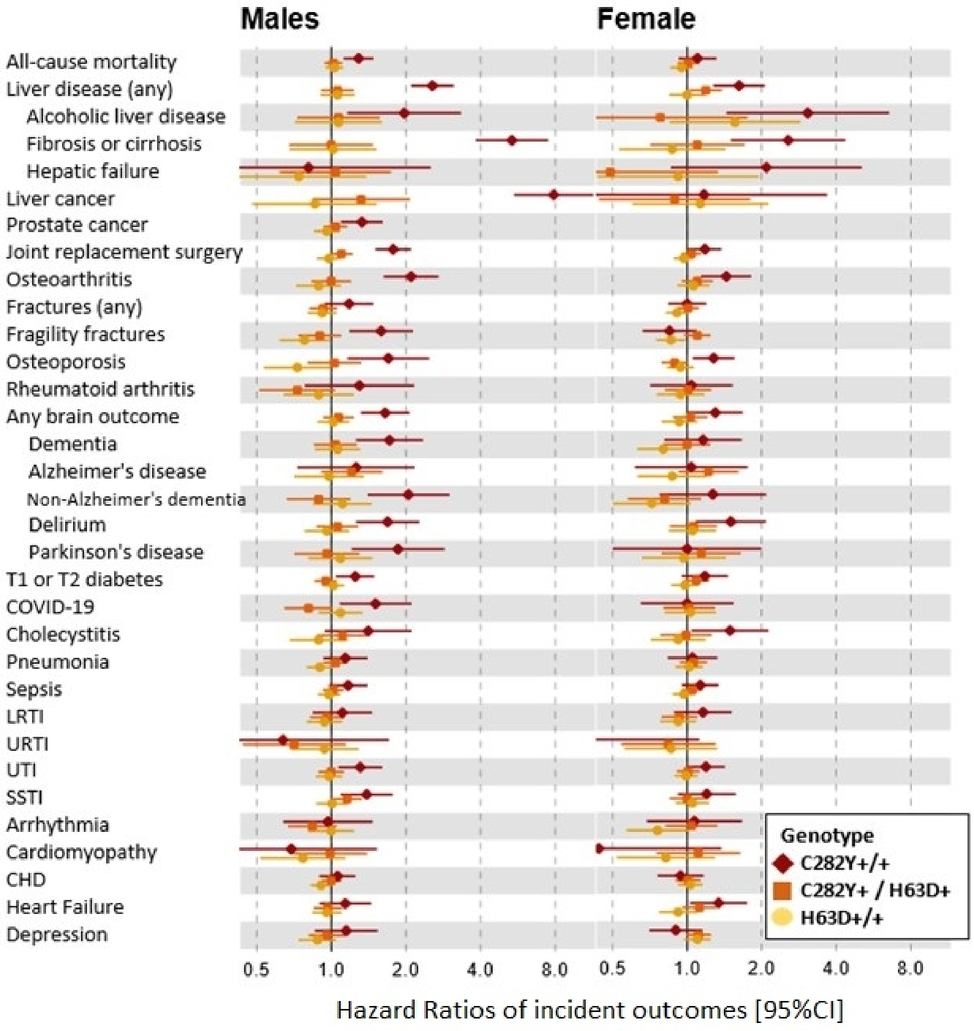

Figure 1

53x56mm (300 x 300 DPI)

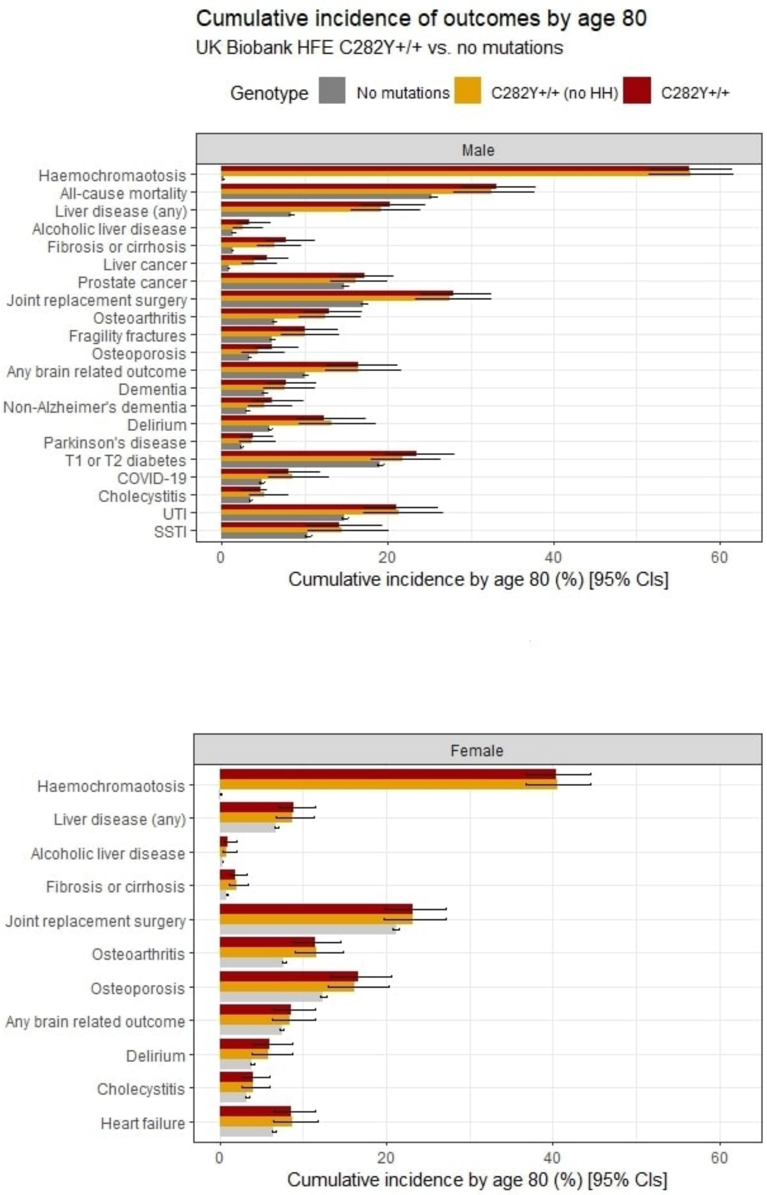

Figure 2

52x79mm (300 x 300 DPI)

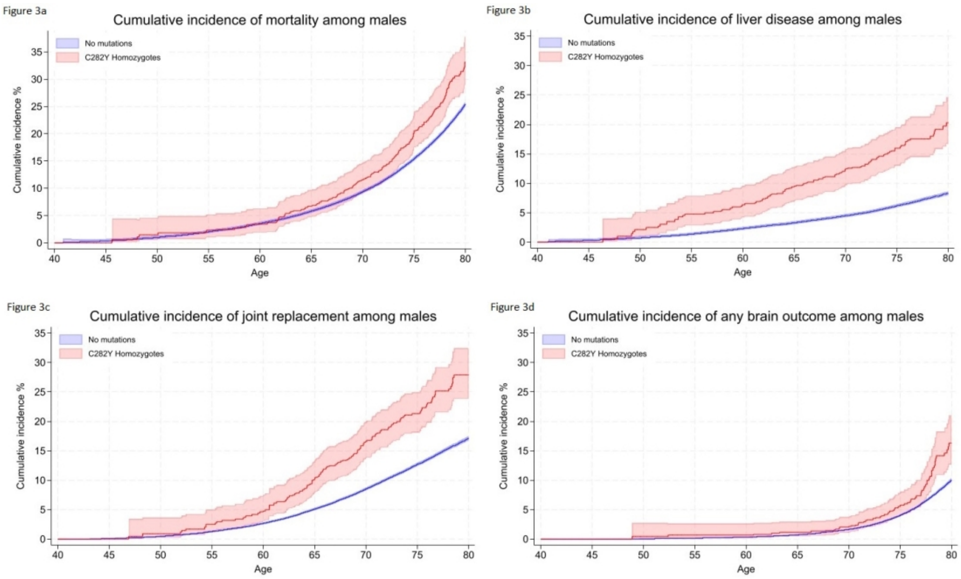

127x76mm (300 x 300 DPI)

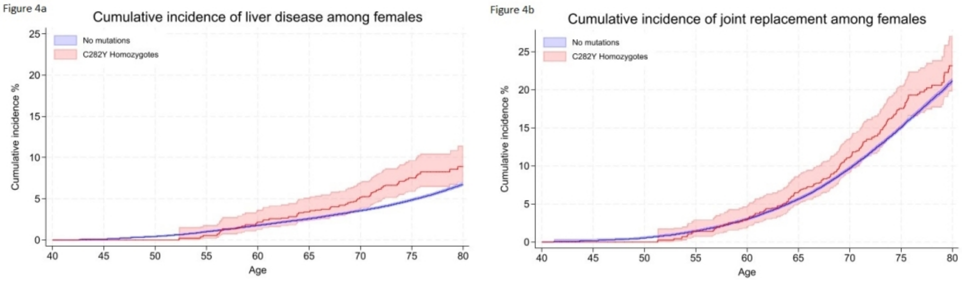

127x36mm (300 x 300 DPI)

Supplementary Material

eTable 1. Incident hospital diagnosed outcomes or procedures with associated ICD-10/OPCS codes

Disease	ICD-10 code
Arrhythmia	I49
Cardiomyopathy	I42
CHD	I20; I21; I22; I23; I24; I25
Cholecystitis	K800; K804; K81
COVID-19	U07.1; U07.2
Delirium	F05
Dementia	F00; F01; F02; F03; G30
Alzheimer's disease	G30
Non-Alzheimer's dementia	F00; F01; F02; F03
Depression	F32; F33; F34.1
Fractures (any)	S02; S12; S22; S32; S42; S52; S62; S72; S82; S92; T02; T08; T10; T12; T14.2
Fragility fractures	S220; S32; S325; S328; S422; S423; S424; S524; S525; S720; S721; S722; S582; S5823; T08
Hemochromatosis	E83.1
Heart failure	I50; J81
Liver disease (any)	K70; K71; K72; K73; K74; K75; K76; K77
Alcoholic liver disease	K70
Fibrosis & Cirrhosis	K74
Hepatic failure	K72
Liver cancer	C22
Lower respiratory tract infection	J20; J21; J22
Osteoarthritis	M15.0; M15.1; M15.2; M15.9; M16.0; M16.1; M17.0; M17.1; M18.0; M18.1; M19.0
Osteoporosis	M80; M81; M811; M812; M813; M814; M815; M816; M818; M819
Parkinson's disease	G20; F02.3
Pneumonia	J13; J14; J15; J16; J17; J18
Prostate cancer	C61
Rheumatoid arthritis	M05; M06
Sepsis	A021; A039; A207; A241; A217; A227; A239; A267; A282; A327; A392; A393; A394; A40; A41; A427; A548; B007; B377; H440; J950; N390; O85; P36; R651; T814; T880
Skin soft tissue infection	L00; L01; L02; L03; L04; L05; L06; L07; L08
Type 1 or Type 2 diabetes	E10; E11
Urinary tract infection	N30; N34; N39
Upper respiratory tract infection	J39; J06; J04

Procedure	OPCS Code
Ankle replacement	O32; O320; O321; O322; O323; O324; O325
Hip replacement	W37;W370;W371;W372;W373;W374;W38;W380;W381;W382;W383;W384; W46;W460;W461;W462;W463;W47;W470;W471;W472;W473;W93;W930; W931;W932;W933;W94;W940;W941;W942;W943;O171;O172;O173;W580; W581;W582
Knee replacement	O18*; W40*; W41*; W42*
Shoulder replacement	O06; +A11O060; O061; O062; O063; O068; O069; O07; O070; O071; O072; O073; O078; O079; O08; O080; O081; O082; O083; O084; O088; O089; O09; O091; O098; O099; O10; O101; O108; O109

ICD-10 = International Classification of Diseases 10th revision codes; OPCS-4 = OPCS Classification of Interventions and Procedures version 4. Joint replacement surgery variable includes a diagnosis of hip, knee, ankle, or shoulder replacement. Any brain outcome variable includes a diagnosis of dementia, delirium, or Parkinson's disease.

For peer review only

For peer review only - http://bmjopen.bmj.com/site/about/guidelines.xhtml

eTable 2. Baseline characteristics of male UK Biobank participants by p.C282Y/H63D genotypes

	No mutations	H63D+/-	H63D+/+	C282Y+/H63+	C282Y+/-	C282Y +/+	Total
**Total participants**	122,841	47,983	4,673	4,959	24,636	1,298	206,390
Mean age, years (SD)	56.99 (8.1)	57.02 (8.1)	56.99 (8.1)	56.97 (8.1)	57.02 (8.1)	56.84 (8.2)	57.00 (8.1)
Hemochromatosis diagnosis, n (%)	29 (0.02)	17 (0.04)	8 (0.2)	29 (0.6)	27 (0.1)	157 (12.1)	267 (0.1)
Self-reported fatigue, n (%)	12,094 (10.1)	4,865 (10.4)	451 (9.9)	523 (10.9)	2,476 (10.3)	148 (11.8)	20,557 (10.2)
Self-reported fatigue (60+ years), n (%),	4,475 (8.2)	1,858 (8.6)	173 (8.2)	177 (8.0)	924 (8.4)	68 (11.8)	7,675 (8.3)
Depression diagnosis, n (%)	5,883 (4.8)	2,232 (4.7)	220 (4.7)	188 (3.8)	1,146 (4.7)	71 (5.5)	9,740 (4.7)

A total of 206,390 male participants genetically similar to the 1000 Genomes project European reference population with *HFE* genotypic data available in the UK Biobank. Numbers presented are mean (SD) for continuous variables and n (%) for categorical variables. Fatigue = tiredness in more than half the days in past 2 weeks.

For peer review only - http://bmjopen.bmj.com/site/about/guidelines.xhtml

eTable 3. Baseline characteristics of female UK Biobank participants by p.C282Y/H63D genotypes

	No mutations	H63D+/-	H63D+/+	C282Y+/H63+	C282Y+/-	C282Y +/+	Total
**Total participants**	145,694	57,021	5,580	5,760	29,221	1,604	244,880
Mean age, years (SD)	56.62 (7.9)	56.58 (7.9)	56.58 (8.1)	56.46 (7.9)	56.49 (8.0)	56.92 (8.0)	56.60 (7.9)
Hemochromatosis diagnosis, n (%)	8 (0.01)	6 (0.01)	2 (0.04)	12 (0.2)	5 (0.02)	54 (3.4)	87 (0.04)
Self-reported fatigue, n (%)	19,110 (13.5)	7,449 (13.5)	760 (14.1)	785 (14.1)	3,802 (13.4)	220 (14.4)	32,126 (13.5)
Self-reported fatigue (60+ years), n (%),	6,055 (10.0)	2,417 (10.2)	253 (10.9)	229 (9.9)	1,157 (9.6)	77 (11.4)	10,188 (10.0)
Depression diagnosis, n (%)	10,734 (7.4)	4,266 (7.5)	387 (6.9)	432 (7.5)	2,271 (7.8)	123 (7.7)	18,213 (7.4)

A total of 244,880 female participants genetically similar to the 1000 Genomes project European reference population with *HFE* genotypic data available in the UK Biobank. Numbers presented are mean (SD) for continuous variables and n (%) for categorical variables. Fatigue = tiredness in more than half the days in past 2 weeks.

For peer review only - http://bmjopen.bmj.com/site/about/guidelines.xhtml

eTable 4. Incident hospital diagnoses in male UK Biobank participants by p.C282Y/H63D genotypes

**Males**	**No mutations**	**H63D +/-**	**H63D +/+**	**C282Y +/ H36D +**	**C282Y +/-**	**C282Y +/+**
Hemochromatosis	85 (0.1)	51 (0.1)	29 (0.6)	95 (1.9)	42 (0.2)	288 (25.2)
All-cause mortality	13,976 (11.4)	5,658 (11.8)	540 (11.6)	591 (11.9)	2,996 (12.2)	194 (15.0)
**Liver**						
Liver disease (any)	3,969 (3.3)	1,621 (3.4)	160 (3.5)	171 (3.5)	803 (3.3)	102 (8.1)
Alcoholic liver disease	608 (0.5)	227 (0.5)	25 (0.5)	28 (0.6)	150 (0.6)	14 (1.1)
Fibrosis & Cirrhosis	618 (0.5)	252 (0.5)	24 (0.5)	26 (0.5)	142 (0.6)	36 (2.8)
Hepatic failure	355 (0.3)	137 (0.3)	10 (0.2)	15 (0.3)	54 (0.2)	3 (0.2)
**Cancer**						
Liver cancer	363 (0.3)	167 (0.4)	12 (0.3)	20 (0.4)	80 (0.3)	31 (2.4)
Prostate cancer	7,723 (6.4)	3,025 (6.4)	283 (6.2)	319 (6.5)	1,613 (6.7)	104 (8.1)
**Musculoskeletal**						
Joint replacement surgery (any)	8,429 (7.1)	3,379 (7.3)	315 (7.0)	376 (7.8)	1,801 (7.5)	144 (11.8)
Osteoarthritis	2,873 (2.5)	1,155 (2.6)	97 (2.2)	120 (2.6)	601 (2.6)	61 (5.5)
Fractures (any)	6,092 (5.2)	2,298 (5.1)	212 (4.8)	231 (4.9)	1,243 (5.3)	75 (6.2)
Fragility fractures	2,666 (2.2)	963 (2.0)	79 (1.7)	98 (2.0)	551 (2.3)	44 (3.4)
Osteoporosis	1,522 (1.3)	602 (1.3)	42 (0.9)	64 (1.3)	319 (1.3)	27 (2.1)
Rheumatoid arthritis	1,131 (0.9)	479 (1.0)	38 (0.8)	33 (0.7)	238 (1.0)	15 (1.2)
**Brain**						
Any brain outcome (dementia, delirium, or Parkinson's disease)	4,619 (3.8)	1,852 (3.9)	177 (3.8)	201 (4.1)	993 (4.0)	76 (5.9)
Dementia	2,306 (1.9)	916 (1.9)	92 (2.0)	97 (2.0)	487 (2.0)	40 (3.1)
Alzheimer's disease	1,026 (0.8)	380 (0.8)	38 (0.8)	50 (1.0)	189 (0.8)	13 (1.0)
Non-Alzheimer's dementia	1,299 (1.1)	540 (1.1)	54 (1.2)	47 (1.0)	303 (1.2)	27 (2.1)
Delirium	2,621 (2.1)	1,028 (2.1)	95 (2.0)	112 (2.3)	603 (2.5)	45 (3.5)
Parkinson's disease	1,116 (0.9)	454 (1.0)	46 (1.0)	43 (0.9)	202 (0.8)	21 (1.6)
**Pancreas**						
T1 or T2 diabetes	9,515 (8.0)	3,665 (7.9)	368 (8.1)	367 (7.6)	1,822 (7.7)	119 (9.6)
**Infection**						
Covid-19	2,332 (1.9)	956 (2.0)	96 (2.1)	77 (1.6)	522 (2.1)	35 (2.7)
Cholecystitis	1,624 (1.3)	613 (1.3)	55 (1.2)	73 (1.5)	318 (1.3)	24 (1.9)

For peer review only - http://bmjopen.bmj.com/site/about/guidelines.xhtml

Pneumonia	8,019 (6.7)	3,128 (6.7)	273 (6.0)	342 (7.1)	1,651 (6.9)	95 (7.6)
Sepsis	10,081 (8.4)	3,955 (8.4)	374 (8.2)	416 (8.6)	2,065 (8.6)	122 (9.7)
LRTI	4,113 (3.4)	1,694 (3.6)	148 (3.2)	164 (3.3)	914 (3.7)	50 (3.9)
URTI	588 (0.5)	226 (0.5)	21 (0.5)	17 (0.3)	109 (0.4)	4 (0.3)
UTI	7,099 (5.9)	2,773 (5.9)	263 (5.7)	290 (6.0)	1,472 (6.1)	96 (7.6)
SSTI	4,604 (3.8)	1,792 (3.8)	176 (3.8)	216 (4.5)	1,053 (4.4)	66 (5.2)
**Cardiovascular**						
Arrhythmia	2,283 (1.9)	871 (1.8)	86 (1.9)	77 (1.6)	445 (1.8)	23 (1.8)
Cardiomyopathy	855 (0.7)	330 (0.7)	25 (0.5)	34 (0.7)	179 (0.7)	6 (0.5)
CHD	11,796 (10.5)	4,626 (10.5)	412 (9.6)	485 (10.5)	2,422 (10.7)	133 (11.0)
Heart failure	5,779 (4.7)	2,289 (4.8)	211 (4.6)	229 (4.7)	1,256 (5.1)	68 (5.3)
**Mental Health**						
Depression	3,929 (3.4)	1,615 (3.5)	132 (3.0)	152 (3.2)	772 (3.3)	46 (3.8)

Incident disease numbers exclude prevalent disease at baseline. Numbers presented are n (%). Abbreviations: CHD, coronary heart disease; T1, type 1; T2, type 2; LRTI, lower respiratory tract infection; URTI, upper respiratory tract infection; UTI, urinary tract infection; SSTI, skin and soft tissue infection. Joint replacement surgery variable includes a diagnosis of hip, knee, ankle, or shoulder replacement. Any brain outcome variable includes a diagnosis of dementia, delirium, or Parkinson's disease.

For peer review only - http://bmjopen.bmj.com/site/about/guidelines.xhtml

eTable 5. Hazard ratios of incident disease outcomes in p.C282Y/H63D genotypes in males

**Males**	**No mutations**	**H63D +/-**		**H63D +/+**		**C282Y +/ H36D +**		**C282Y +/-**		**C282Y +/+**	
		HR (95% CI)	P	HR (95% CI)	P	HR (95% CI)	P	HR (95% CI)	P	HR (95% CI)	P
Hemochromatosis	1	1.53 (1.08-2.17)	0.02	8.95 (5.87-13.64)	$2.20*10^{-24}$	27.47 (20.49-36.84)	$1.00*10^{-108}$	2.40 (1.66-3.47)	$3.70*10^{-06}$	405.29 (317.06-518.11)	$2.97*10^{-501}$
All-cause mortality	1	1.03 (1.00-1.06)	0.05	1.01 (0.93-1.11)	0.75	1.02 (0.94-1.11)	0.58	1.05 (1.00-1.09)	0.03	1.29 (1.12-1.48)	$4.70*10^{-04}$
**Liver**											
Liver disease (any)	1	1.05 (0.99-1.11)	0.12	1.06 (0.90-1.24)	0.48	1.06 (0.91-1.23)	0.46	1.00 (0.93-1.78)	0.96	2.56 (2.10-3.12)	$8.70*10^{-21}$
Alcoholic liver disease	1	0.95 (0.81-1.11)	0.50	1.07 (0.71-1.59)	0.76	1.07 (0.73-1.57)	0.72	1.17 (0.98-1.40)	0.09	1.97 (1.16-3.35)	0.01
Fibrosis & Cirrhosis	1	1.04 (0.90-1.21)	0.58	1.02 (0.68-1.53)	0.93	1.00 (0.68-1.48)	0.99	1.11 (0.92-1.33)	0.28	5.36 (3.83-7.52)	$2.00*10^{-22}$
Hepatic failure	1	0.99 (0.81-1.21)	0.92	0.74 (0.40-1.40)	0.36	1.04 (0.62-1.74)	0.88	0.75 (0.56-1.00)	0.05	0.81 (0.26-2.52)	0.72
**Cancer**											
Liver cancer	1	1.17 (0.98-1.41)	0.09	0.86 (0.48-1.53)	0.61	1.32 (0.84-2.08)	0.22	1.07 (0.84-1.36)	0.61	7.90 (5.46-11.43)	$5.50*10^{-28}$
Prostate cancer	1	1.00 (0.96-1.05)	0.86	0.96 (0.86-1.09)	0.56	1.03 (0.93-1.16)	0.55	1.05 (1.00-1.11)	0.09	1.33 (1.09-1.61)	0.004
**Musculoskeletal**											
Joint replacement surgery (any)	1	1.03 (0.99-1.07)	0.21	0.98 (0.88-1.10)	0.74	1.10 (1.00-1.22)	0.08	1.06 (1.01-1.12)	0.02	1.78 (1.51-2.10)	$6.40*10^{-12}$
Osteoarthritis	1	1.03 (0.96-1.10)	0.44	0.89 (0.72-1.09)	0.25	1.01 (0.84-1.21)	0.99	1.03 (0.94-1.12)	0.55	2.10 (1.63-2.71)	$1.10*10^{-08}$
Fractures (any)	1	0.96 (0.92-1.01)	0.13	0.92 (0.80-1.05)	0.23	0.93 (0.82-1.06)	0.29	1.01 (0.95-1.07)	0.85	1.18 (0.94-1.48)	0.16
Fragility fractures	1	0.92 (0.86-0.99)	0.03	0.78 (0.62-0.98)	0.03	0.90 (0.74-1.10)	0.31	1.02 (0.93-1.12)	0.68	1.59 (1.18-2.14)	0.002
Osteoporosis	1	1.01 (0.92-1.11)	0.85	0.73 (0.53-0.99)	0.04	1.03 (0.80-1.32)	0.82	1.02 (0.91-1.16)	0.70	1.70 (1.16-2.48)	0.007
Rheumatoid arthritis	1	1.08 (0.97-1.21)	0.13	0.89 (0.64-1.23)	0.48	0.73 (0.51-1.03)	0.07	1.05 (0.91-1.20)	0.53	1.30 (0.78-2.16)	0.31
**Brain**											
Any brain outcome (dementia, delirium, or Parkinson's disease)	1	1.02 (0.97-1.08)	0.39	1.02 (0.88-1.18)	0.83	1.07 (0.93-1.24)	0.32	1.06 (0.99-1.13)	0.11	1.65 (1.31-2.06)	$1.70*10^{-05}$
Dementia	1	1.01 (0.94-1.09)	0.76	1.06 (0.86-1.31)	0.58	1.04 (0.85-1.27)	0.73	1.04 (0.94-1.14)	0.48	1.72 (1.26-2.35)	0.001
Alzheimer's disease	1	0.94 (0.84-1.06)	0.32	0.98 (0.71-1.36)	0.91	1.21 (0.91-1.60)	0.19	0.90 (0.77-1.05)	0.20	1.26 (0.73-2.17)	0.42
Non-Alzheimer's dementia	1	1.06 (0.96-1.17)	0.25	1.11 (0.84-1.45)	0.46	0.89 (0.66-1.19)	0.43	1.14 (1.01-1.30)	0.04	2.05 (1.40-3.00)	$2.40*10^{-04}$
Delirium	1	1.00 (0.93-1.08)	0.98	0.96 (0.78-1.18)	0.69	1.06 (0.87-1.28)	0.57	1.13 (1.04-1.24)	0.006	1.69 (1.26-2.27)	$4.80*10^{-04}$
Parkinson's disease	1	1.04 (0.93-1.16)	0.47	1.09 (0.81-1.47)	0.55	0.96 (0.71-1.30)	0.77	0.90 (0.77-1.04)	0.16	1.86 (1.21-2.87)	0.005
**Pancreas**											

For peer review only

T1 or T2 diabetes	1	0.98 (0.95-1.02)	0.44	1.02 (0.92-1.13)	0.69	0.95 (0.86-1.06)	0.35	0.95 (0.91-1.00)	0.06	1.25 (1.05-1.50)	0.01
**Infection**											
COVID-19	1	1.05 (0.97-1.13)	0.21	1.09 (0.89-1.34)	0.4	0.81 (0.65-1.02)	0.07	1.11 (1.01-1.22)	0.03	1.51 (1.08-2.11)	0.02
Cholecystitis	1	0.96 (0.88-1.06)	0.45	0.89 (0.68-1.16)	0.39	1.11 (0.87-1.40)	0.4	0.96 (0.86-1.09)	0.56	1.41 (0.94-2.11)	0.09
Pneumonia	1	1.00 (0.96-1.04)	0.82	0.90 (0.80-1.02)	0.09	1.04 (0.93-1.16)	0.51	1.01 (0.96-1.07)	0.68	1.14 (0.93-1.40)	0.21
Sepsis	1	1.01 (0.97-1.04)	0.78	0.98 (0.88-1.08)	0.66	1.02 (0.92-1.12)	0.7	1.01 (0.96-1.06)	0.70	1.17 (0.98-1.40)	0.09
LRTI	1	1.05 (0.99-1.11)	0.10	0.94 (0.80-1.11)	0.46	0.96 (0.82-1.12)	0.58	1.08 (1.00-1.16)	0.48	1.11 (0.84-1.46)	0.48
URTI	1	0.99 (0.84-1.15)	0.85	0.94 (0.61-1.45)	0.77	0.71 (0.44-1.15)	0.16	0.92 (0.75-1.13)	0.41	0.64 (0.24-1.71)	0.38
UTI	1	1.00 (0.95-1.04)	0.90	0.98 (0.86-1.10)	0.69	1.00 (0.89-1.13)	0.98	1.02 (0.96-1.08)	0.51	1.31 (1.07-1.61)	0.008
SSTI	1	1.00 (0.94-1.05)	0.88	1.01 (0.87-1.17)	0.90	1.16 (1.01-1.33)	0.03	1.14 (1.06-1.22)	$1.80*10^{-04}$	1.39 (1.09-1.77)	0.008
**Cardiovascular**											
Arrhythmia	1	0.98 (0.90-1.06)	0.57	1.00 (0.81-1.24)	0.99	0.84 (0.67-1.05)	0.13	0.96 (0.87-1.07)	0.49	0.97 (0.64-1.47)	0.89
Cardiomyopathy	1	0.99 (0.87-1.12)	0.88	0.77 (0.52-1.15)	0.21	0.99 (0.70-1.39)	0.94	1.04 (0.89-1.23)	0.60	0.69 (0.31-1.53)	0.36
CHD	1	1.01 (0.97-1.04)	0.77	0.91 (0.83-1.01)	0.08	1.00 (0.91-1.10)	0.99	1.02 (0.97-1.06)	0.45	1.06 (0.90-1.26)	0.49
Heart failure	1	1.01 (0.96-1.06)	0.67	0.96 (0.84-1.10)	0.59	0.97 (0.85-1.11)	0.65	1.07 (1.01-1.14)	0.03	1.14 (0.89-1.44)	0.29
**Mental Health**											
Depression	1	1.05 (0.99-1.12)	0.07	0.88 (0.74-1.05)	0.16	0.96 (0.81-1.13)	0.60	0.98 (0.91-1.06)	0.59	1.15 (0.86-1.54)	0.34

HR (Hazard ratio) compared to those with neither *HFE* mutation. Cox proportional hazards regression models adjusted for age, assessment centre, and genetic principal components 1–10. Abbreviations: CHD, coronary heart disease; T1, type 1; T2, type 2; LRTI, lower respiratory tract infection; URTI, upper respiratory tract infection; UTI, urinary tract infection; SSTI, skin and soft tissue infection; CI, confidence interval. Joint replacement surgery variable includes a diagnosis of hip, knee, ankle, or shoulder replacement. Any brain outcome variable includes a diagnosis of dementia, delirium, or Parkinson's disease.

For peer review only

eTable 6. Incident hospital diagnoses in male UK Biobank participants by p.C282Y/H63D genotypes, excluding a diagnosis of hemochromatosis at baseline

**Males**	**No mutations**	**H63D +/-**	**H63D +/+**	**C282Y +/ H36D +**	**C282Y +/-**	**C282Y +/+**
Hemochromatosis	85 (14.41)	51 (8.64)	29 (4.92)	95 (16.10)	42 (7.12)	288 (48.81)
All-cause mortality	13959 (58.44)	5653 (23.66)	539 (2.26)	587 (2.46)	2990 (12.52)	160 (0.67)
**Liver**						
Liver disease (any)	3969 (58.38)	1619 (23.82)	158 (2.32)	167 (2.46)	800 (11.77)	85 (1.25)
Alcoholic liver disease	607 (58.20)	226 (21.67)	25 (2.40)	27 (2.59)	148 (14.19)	10 (0.96)
Fibrosis & Cirrhosis	618 (57.06)	250 (23.08)	23 (2.12)	25 (2.31)	140 (12.93)	27 (2.49)
Hepatic failure	355 (62.06)	137 (23.95)	10 (1.75)	15 (2.65)	54 (9.44)	1 (0.17)
**Cancer**						
Liver cancer	361 (55.28)	166 (25.42)	12 (1.84)	19 (2.91)	78 (11.94)	17 (2.60)
Prostate cancer	7722 (59.20)	3023 (23.18)	283 (2.17)	318 (2.44)	1610 (12.34)	87 (0.67)
**Musculoskeletal**						
Joint replacement surgery (any)	8427 (58.44)	3377 (23.42)	315 (2.18)	375 (2.60)	1801 (12.49)	126 (0.87)
Osteoarthritis	2872 (58.70)	1153 (23.56)	97 (1.98)	120 (2.45)	600 (12.26)	51 (1.04)
Fractures (any)	6090 (60.07)	2296 (22.65)	212 (2.09)	231 (2.28)	1243 (12.26)	66 (0.65)
Fragility fractures	2663 (60.63)	963 (21.93)	79 (1.80)	98 (2.23)	550 (12.52)	39 (0.89)
Osteoporosis	1521 (59.37)	601 (23.46)	42 (1.64)	63 (2.46)	319 (12.45)	16 (0.62)
Rheumatoid arthritis	1130 (58.55)	479 (24.82)	38 (1.97)	33 (1.71)	238 (12.33)	12 (0.62)
**Brain**						
Any brain outcome (dementia, delirium, or Parkinson's disease)	4616 (58.43)	1849 (23.41)	177 (2.24)	200 (2.53)	992 (12.56)	66 (0.84)
Dementia	2305 (58.67)	915 (23.29)	92 (2.34)	97 (2.47)	487 (12.40)	33 (0.84)
Alzheimer's disease	1026 (60.53)	380 (22.42)	38 (2.24)	50 (2.95)	189 (11.15)	12 (0.71)
Non-Alzheimer's dementia	1298 (57.38)	539 (23.83)	54 (2.39)	47 (2.08)	303 (13.40)	21 (0.93)
Delirium	2619 (58.29)	1025 (22.81)	95 (2.11)	111 (2.47)	602 (13.40)	41 (0.91)
Parkinson's disease	1115 (59.34)	454 (24.16)	46 (2.45)	43 (2.29)	202 (10.75)	19 (1.01)
**Pancreas**						

For peer review only - http://bmjopen.bmj.com/site/about/guidelines.xhtml

T2 diabetes	9570 (60.17)	3673 (23.09)	365 (2.30)	368 (2.31)	1831 (11.51)	97 (0.61)
**Infection**						
Covid-19	2331 (58.14)	953 (23.77)	96 (2.39)	76 (1.90)	522 (13.02)	31 (0.77)
Cholecystitis	1623 (60.07)	612 (22.65)	55 (2.04)	73 (2.70)	316 (11.70)	23 (0.85)
Pneumonia	8014 (59.44)	3124 (23.17)	273 (2.02)	340 (2.52)	1649 (12.23)	83 (0.62)
Sepsis	10075 (59.34)	3951 (23.27)	374 (2.20)	412 (2.43)	2063(12.15)	103 (0.61)
LRTI	4110 (58.12)	1691 (23.91)	148 (2.09)	164 (2.32)	913 (12.91)	45 (0.64)
URTI	588 (61.06)	226 (23.47)	21 (2.18)	17 (1.77)	108 (11.21)	3 (0.31)
UTI	7097 (59.29)	2769 (23.13)	263 (2.20)	286 (2.39)	1470 (12.28)	85 (0.71)
SSTI	4599 (58.27)	1790 (22.68)	176 (2.23)	215 (2.72)	1053 (13.34)	59 (0.75)
**Cardiovascular**						
Arrhythmia	2282 (60.40)	870 (23.03)	85 (2.25)	77 (2.04)	444 (11.75)	20 (0.53)
Cardiomyopathy	853 (59.86)	330 (23.16)	25 (1.75)	34 (2.39)	178 (12.49)	5 (0.35)
CHD	11793 (59.45)	4622 (23.30)	411 (2.07)	481 (2.42)	2414 (12.17)	115 (0.58)
Heart failure	5775 (58.87)	2285 (23.29)	211 (2.15)	227 (2.31)	1253 (12.77)	59 (0.60)
**Mental Health**						
Depression	3925 (59.18)	1614 (24.34)	132 (1.99)	150 (2.26)	771 (11.63)	40 (0.60)

Incident disease numbers exclude prevalent disease at baseline. Numbers presented are n (%). Abbreviations: CHD, coronary heart disease; T1, type 1; T2, type 2; LRTI, lower respiratory tract infection; URTI, upper respiratory tract infection; UTI, urinary tract infection; SSTI, skin and soft tissue infection. Joint replacement surgery variable includes a diagnosis of hip, knee, ankle, or shoulder replacement. Any brain outcome variable includes a diagnosis of dementia, delirium, or Parkinson's disease.

For peer review only - http://bmjopen.bmj.com/site/about/guidelines.xhtml

eTable 7. Hazard ratios of incident disease outcomes in p.C282Y/H63D genotypes in males, excluding a diagnosis of hemochromatosis at baseline

Males	No mutations (Ref group)	H63D +/-		H63D +/+		C282Y +/ H36D +		C282Y +/-		C282Y +/+	
		HR (95% CI)	P	HR (95% CI)	P	HR (95% CI)	P	HR (95% CI)	P	HR (95% CI)	P
Hemochromatosis	1	1.53 (1.08-2.17)	0.02	8.95 (5.87-13.64)	$2.2*10^{-24}$	27.49 (20.50-36.86)	$1.0*10^{-108}$	2.40 (1.66-3.47)	$3.6*10^{-06}$	405.79 (317.34-518.75)	0.00E+00
All-cause mortality	1	1.03 (1.00-1.07)	0.04	1.01 (0.93-1.11)	0.74	1.02 (0.94-1.11)	0.58	1.05 (1.00-1.09)	0.03	1.22 (1.05-1.43)	0.01
**Liver**											
Liver disease (any)	1	1.05 (0.99-1.11)	0.13	1.05 (0.89-1.23)	0.57	1.04 (0.89-1.21)	0.63	1.00 (0.92-1.07)	0.9	2.36 (1.90-2.93)	$5.3*10^{-15}$
Alcoholic liver disease	1	0.95 (0.12-1.10)	0.48	1.07 (0.72-1.60)	0.74	1.04 (0.71-1.53)	0.84	1.15 (0.96-1.38)	0.12	1.59 (0.85-2.97)	0.15
Fibrosis & Cirrhosis	1	1.03 (0.89-1.20)	0.66	0.98 (0.64-1.48)	0.91	0.97 (0.65-1.44)	0.87	1.09 (0.91-1.31)	0.35	4.52 (3.07-6.66)	$2.1*10^{-14}$
Hepatic failure	1	0.99 (0.81-1.21)	0.92	0.75 (0.40-1.40)	0.36	1.05 (0.62-1.76)	0.86	0.75 (0.56-1.00)	0.05	0.31 (0.04-2.19)	0.24
**Cancer**											
Liver cancer	1	1.17 (0.98-1.41)	0.09	0.87 (0.49-1.54)	0.63	1.27 (0.80-2.02)	0.3	1.05 (0.82-1.34)	0.71	4.97 (3.05-8.11)	$1.2*10^{-10}$
Prostate cancer	1	1.00 (0.96-1.05)	0.87	0.97 (0.86-1.09)	0.57	1.04 (0.93-1.16)	0.51	1.05 (0.99-1.11)	0.09	1.27 (1.03-1.57)	0.03
**Musculoskeletal**											
Joint replacement surgery (any)	1	1.03 (0.99-1.07)	0.22	0.98 (0.88-1.10)	0.75	1.10 (0.99-1.22)	0.06	1.06 (1.01-1.12)	0.02	1.75 (1.47-2.09)	$4.8*10^{-10}$
Osteoarthritis	1	1.03 (0.96-1.10)	0.46	0.89 (0.73-1.09)	0.26	1.02 (0.85-1.22)	0.87	1.03 (0.94-1.12)	0.56	1.97 (1.50-2.60)	$1.5*10^{-06}$
Fractures (any)	1	0.96 (0.92-1.01)	0.12	0.92 (0.80-1.06)	0.24	0.94 (0.82-1.07)	0.34	1.01 (0.95-1.07)	0.82	1.17 (0.91-1.49)	0.22
Fragility fractures	1	0.92 (0.86-0.99)	0.03	0.78 (0.63-0.98)	0.03	0.91 (0.74-1.11)	0.34	1.02 (0.93-1.12)	0.68	1.60 (1.17-2.20)	$3.6*10^{-03}$
Osteoporosis	1	1.01 (0.92-1.11)	0.86	0.73 (0.54-0.99)	0.04	1.02 (0.79-1.31)	0.89	1.03 (0.91-1.16)	0.68	1.14 (0.69-1.86)	0.61
Rheumatoid arthritis	1	1.09 (0.98-1.21)	0.13	0.89 (0.64-1.23)	0.48	0.73 (0.52-1.03)	0.07	1.05 (0.91-1.20)	0.52	1.17 (0.66-2.07)	0.58
**Brain**											
Any brain outcome (dementia, delirium, or Parkinson's disease)	1	1.02 (0.97-1.08)	0.4	1.02 (0.88-1.18)	0.8	1.08 (0.93-1.24)	0.31	1.06 (0.99-1.13)	0.1	1.62 (1.27-2.07)	$1.0*10^{-04}$
Dementia	1	1.01 (0.94-1.09)	0.76	1.06 (0.86-1.31)	0.57	1.04 (0.85-1.28)	0.69	1.04 (0.94-1.14)	0.46	1.60 (1.14-2.26)	0.01
Alzheimer's disease	1	0.94 (0.84-1.06)	0.32	0.98 (0.71-1.36)	0.92	1.12 (0.91-1.61)	0.18	0.90 (0.77-1.06)	0.2	1.31 (0.74-2.31)	0.35
Non-Alzheimer's dementia	1	1.06 (0.96-1.17)	0.26	1.11 (0.85-1.46)	0.45	0.89 (0.67-1.20)	0.45	1.14 (1.01-1.30)	0.04	1.81 (1.17-2.78)	0.01
Delirium	1	1.00 (0.93-1.07)	0.98	0.96 (0.78-1.18)	0.7	1.05 (0.87-1.27)	0.59	1.13 (1.04-1.24)	0.01	1.75 (1.28-2.38)	$3.9*10^{-04}$
Parkinson's disease	1	1.07 (0.98-1.16)	0.13	1.05 (0.84-1.31)	0.67	1.06 (0.85-1.33)	0.58	1.01 (0.91-1.13)	0.79	1.44 (1.02-2.03)	0.04

For peer review only

For peer review only - http://bmjopen.bmj.com/site/about/guidelines.xhtml

**Pancreas**											
T1 or T2 diabetes	1	0.99 (0.95-1.02)	0.45	1.02 (0.92-1.14)	0.66	0.94 (0.85-1.05)	0.28	0.95 (0.91-1.00)	0.06	1.17 (0.96-1.43)	0.11
**Infection**											
COVID-19	1	1.05 (0.97-1.13)	0.23	1.09 (0.89-1.34)	0.39	0.81 (0.64-1.01)	0.07	1.11 (1.01-1.23)	0.03	1.51 (1.06-2.15)	0.02
Cholecystitis	1	0.96 (0.88-1.06)	0.44	0.89 (0.68-1.17)	0.4	1.11 (0.88-1.41)	0.37	0.96 (0.85-1.08)	0.51	1.54 (1.02-2.32)	0.04
Pneumonia	1	0.99 (0.95-1.04)	0.8	0.90 (0.80-1.02)	0.09	1.04 (0.93-1.16)	0.52	1.01 (0.96-1.07)	0.66	1.13 (0.91-1.40)	0.27
Sepsis	1	1.01 (0.97-1.04)	0.79	0.98 (0.88-1.09)	0.69	1.01 (0.92-1.12)	0.82	1.01 (0.96-1.06)	0.68	1.12 (0.92-1.36)	0.26
LRTI	1	1.05 (0.99-1.11)	0.1	0.94 (0.80-1.11)	0.48	0.96 (0.82-1.13)	0.63	1.08 (1.00-1.16)	0.04	1.14 (0.85-1.52)	0.4
URTI	1	0.99 (0.85-1.15)	0.85	0.94 (0.61-1.45)	0.78	0.71 (0.44-1.16)	0.17	0.91 (0.74-1.12)	0.37	0.55 (0.18-1.70)	0.3
UTI	1	1.00 (0.95-1.04)	0.86	0.98 (0.86-1.11)	0.71	1.00 (0.89-1.12)	0.96	1.02 (0.96-1.08)	0.51	1.32 (1.07-1.64)	0.01
SSTI	1	1.00 (0.94-1.05)	0.89	1.01 (0.87-1.18)	0.87	1.16 (1.01-1.33)	0.03	1.14 (1.07-1.22)	$1.4*10^{-04}$	1.40 (1.09-1.82)	0.01
**Cardiovascular**											
Arrhythmia	1	0.98 (0.90-1.06)	0.56	0.99 (0.80-1.23)	0.92	0.84 (0.67-1.06)	0.15	0.96 (0.87-1.07)	0.48	0.96 (0.62-1.49)	0.86
Cardiomyopathy	1	0.99 (0.87-1.13)	0.91	0.78 (0.52-1.16)	0.21	0.99 (0.71-1.40)	0.98	1.04 (0.89-1.22)	0.62	0.65 (0.27-1.57)	0.34
CHD	1	1.00 (0.97-1.04)	0.8	0.91 (0.83-1.01)	0.71	1.00 (0.91-1.09)	0.97	1.01 (0.97-1.06)	0.52	1.04 (0.87-1.25)	0.66
Heart failure	1	1.01 (0.96-1.06)	0.69	0.96 (0.84-1.11)	0.61	0.97 (0.85-1.10)	0.62	1.07 (1.01-1.14)	0.03	1.12 (0.87-1.45)	0.38
**Mental Health**											
Depression	1	1.05 (1.00-1.12)	0.07	0.88 (0.74-1.05)	0.17	0.95 (0.81-1.12)	0.55	0.98 (0.91-1.06)	0.6	1.13 (0.82-1.54)	0.46

HR (Hazard ratio) compared to those with neither *HFE* mutation. Cox proportional hazards regression models adjusted for age, assessment centre, and genetic principal components 1–10. Abbreviations: CHD, coronary heart disease; T1, type 1; T2, type 2; LRTI, lower respiratory tract infection; URTI, upper respiratory tract infection; UTI, urinary tract infection SSTI, skin and soft tissue infection; CI, confidence interval. Joint replacement surgery variable includes a diagnosis of hip, knee, ankle, or shoulder replacement. Any brain outcome variable includes a diagnosis of dementia, delirium, or Parkinson's disease.

For peer review only

For peer review only - http://bmjopen.bmj.com/site/about/guidelines.xhtml

eTable 8. Cumulative incidence of hemochromatosis from ages 40-80 years by HFE genotypes

	Total Cohort (n=451,270)			No mutations			H63D Heterozygotes			H63D Homozygotes			Compound C282Y/H63D Heterozygotes			p.C282Y Heterozygotes			p.C282Y homozygotes		
	Incident diagnosis			Incident diagnosis			Incident diagnosis			Incident diagnosis			Incident diagnosis			Incident diagnosis			Incident diagnosis		
	%	(95% CI)		%	(95% CI)		%	(95% CI)		%	(95% CI)		%	(95% CI)		%	(95% CI)		%	(95% CI)	
**Male age group (years)**																					
**40 - 45**	0.1	0.0	0.2	0.0	0.0	0.2	0.0	0.0	0.0	0.0	0.0	0.0	0.3	0.0	1.9	0.0	0.0	0.0	6.8	2.8	16.1
**46 - 50**	0.1	0.1	0.2	0.0	0.0	0.2	0.0	0.0	0.1	0.4	0.1	1.3	0.7	0.2	2.0	0.0	0.0	0.0	12.5	7.3	20.8
**51 - 55**	0.2	0.2	0.3	0.0	0.0	0.2	0.0	0.0	0.1	0.6	0.3	1.5	1.5	0.8	2.6	0.0	0.0	0.1	20.4	14.7	27.9
**56 - 60**	0.3	0.3	0.4	0.1	0.0	0.2	0.1	0.0	0.1	0.8	0.4	1.6	2.1	1.3	3.2	0.1	0.1	0.2	28.4	22.7	35.3
**61 - 65**	0.4	0.3	0.5	0.1	0.0	0.2	0.1	0.1	0.2	1.0	0.6	1.8	2.6	1.8	3.8	0.2	0.1	0.3	36.1	30.5	42.4
**66 - 70**	0.5	0.5	0.6	0.1	0.1	0.2	0.2	0.1	0.2	1.2	0.7	2.1	3.6	2.7	4.8	0.3	0.2	0.4	43.0	37.6	48.7
**71 - 75**	0.6	0.6	0.7	0.2	0.1	0.2	0.2	0.1	0.3	1.4	0.9	2.2	4.3	3.4	5.5	0.3	0.2	0.4	48.9	43.8	54.2
**76 - 80**	0.8	0.7	0.9	0.2	0.1	0.3	0.3	0.2	0.4	1.9	1.3	2.9	5.4	4.3	6.8	0.4	0.3	0.6	56.4	51.4	61.6
**Female age group (years)**																					
**40 - 45**	0.0	0.0	0.0	0.0	0.0	0.0	0.0	0.0	0.0	0.0	0.0	0.0	0.2	0.0	1.5	0.0	0.0	0.0	0.0	0.0	0.0
**46 - 50**	0.0	0.0	0.1	0.0	0.0	0.0	0.0	0.0	0.1	0.2	0.1	0.9	0.3	0.1	1.3	0.0	0.0	0.0	3.4	1.7	6.7
**51 - 55**	0.1	0.1	0.1	0.0	0.0	0.0	0.0	0.0	0.1	0.3	0.1	1.0	0.5	0.2	1.3	0.0	0.0	0.1	8.2	5.7	11.7
**56 - 60**	0.1	0.1	0.2	0.0	0.0	0.0	0.1	0.0	0.1	0.4	0.2	1.0	0.7	0.4	1.5	0.1	0.1	0.2	14.5	11.5	18.1
**61 - 65**	0.2	0.2	0.3	0.0	0.0	0.0	0.1	0.0	0.1	0.5	0.2	1.1	1.1	0.6	1.8	0.1	0.1	0.2	21.2	17.9	24.9
**66 - 70**	0.3	0.3	0.3	0.0	0.0	0.1	0.1	0.1	0.2	0.5	0.2	1.1	1.5	1.0	2.2	0.2	0.1	0.3	27.6	24.2	31.3
**71 - 75**	0.4	0.3	0.4	0.1	0.0	0.1	0.1	0.1	0.2	0.6	0.3	1.2	1.9	1.4	2.7	0.2	0.2	0.3	33.5	30.0	37.2
**76 - 80**	0.5	0.5	0.6	0.1	0.1	0.1	0.1	0.1	0.2	0.6	0.3	1.3	2.7	2.0	3.6	0.3	0.2	0.5	40.5	36.7	44.5

For peer review only - http://bmjopen.bmj.com/site/about/guidelines.xhtml

eTable 9. Incident hospital diagnoses in female UK Biobank participants by p.C282Y/H63D genotypes

**Females**	**No mutations**	**H63D +/-**	**H63D +/+**	**C282Y +/ H36D +**	**C282Y +/-**	**C282Y +/+**
Hemochromatosis	44 (0.03)	35 (0.1)	12 (0.2)	57 (1.0)	37 (0.1)	291 (18.8)
All-cause mortality	9,849 (6.8)	3,810 (6.7)	363 (6.5)	393 (6.8)	2,020 (6.9)	129 (8.0)
**Liver**						
Liver disease (any)	3,822 (2.6)	1,512 (2.7)	146 (2.6)	179 (3.1)	825 (2.9)	69 (4.3)
Alcoholic liver disease	181 (0.1)	78 (0.1)	11 (0.2)	6 (0.1)	59 (0.2)	7 (0.4)
Fibrosis & Cirrhosis	475 (0.3)	163 (0.3)	16 (0.3)	21 (0.4)	89 (0.3)	14 (0.9)
Hepatic failure	200 (0.1)	60 (0.1)	7 (0.1)	4 (0.1)	35 (0.1)	5 (0.3)
**Cancer**						
Liver cancer	232 (0.2)	114 (0.2)	10 (0.2)	8 (0.1)	49 (0.2)	3 (0.2)
**Musculoskeletal**						
Joint replacement surgery (any)	12,356 (8.7)	4,772 (8.6)	454 (8.4)	501 (8.9)	2,415 (8.5)	158 (10.3)
Osteoarthritis	4,344 (3.3)	1,681 (3.3)	177 (3.6)	190 (3.7)	893 (3.4)	70 (5.1)
Fractures (any)	10,666 (7.6)	4,204 (7.7)	376 (7.0)	430 (7.7)	2,189 (7.8)	124 (8.0)
Fragility fractures	6,308 (4.4)	2,517 (4.5)	214 (3.9)	275 (4.8)	1,265 (4.4)	63 (4.0)
Osteoporosis	6,990 (5.0)	2,580 (4.7)	254 (4.7)	245 (4.4)	1,390 (4.9)	103 (6.7)
Rheumatoid arthritis	2,189 (1.5)	819 (1.5)	79 (1.4)	87 (1.5)	391 (1.4)	26 (1.6)
**Brain**						
Any brain outcome (dementia, delirium, or Parkinson's disease)	3,876 (2.7)	1,547 (2.7)	142 (2.6)	155 (2.7)	777 (2.7)	61 (3.8)
Dementia	2,159 (1.5)	837 (1.5)	68 (1.2)	84 (1.5)	440 (1.5)	31 (1.9)
Alzheimer's disease	1,072 (0.7)	450 (0.8)	37 (0.7)	51 (0.9)	220 (0.8)	14 (0.9)
Non-Alzheimer's dementia	1,099 (0.8)	395 (0.7)	31 (0.6)	34 (0.6)	227 (0.8)	17 (1.1)
Delirium	1,983 (1.4)	823 (1.4)	81 (1.5)	82 (1.4)	406 (1.4)	36 (2.2)
Parkinson's disease	691 (0.5)	249 (0.4)	26 (0.5)	30 (0.5)	134 (0.5)	8 (0.5)
**Pancreas**						
T1 or T2 diabetes	6,532 (4.6)	2,583 (4.6)	245 (4.5)	278 (4.9)	1,327 (4.6)	87 (5.5)
**Infection**						
Covid-19	1,809 (1.2)	720 (1.3)	72 (1.3)	72 (1.3)	383 (1.3)	21 (1.3)
Cholecystitis	1,749 (1.2)	675 (1.2)	62 (1.1)	69 (1.2)	351 (1.2)	30 (1.9)
Pneumonia	6,130 (4.3)	2,408 (4.3)	242 (4.4)	255 (4.5)	1,276 (4.5)	75 (4.8)

For peer review only - http://bmjopen.bmj.com/site/about/guidelines.xhtml

Sepsis	10,026 (7.0)	3,840 (6.9)	379 (7.0)	414 (7.4)	2,103 (7.4)	133 (8.5)
LRTI	3,875 (2.7)	1,439 (2.5)	139 (2.5)	144 (2.5)	821 (2.8)	53 (3.3)
URTI	636 (0.4)	279 (0.5)	21 (0.4)	21 (0.4)	142 (0.5)	2 (0.1)
UTI	9,140 (6.5)	3,493 (6.4)	351 (6.6)	366 (6.7)	1,905 (6.8)	126 (8.2)
SSTI	3,900 (2.7)	1,534 (2.7)	158 (2.9)	153 (2.7)	799 (2.8)	53 (3.3)
**Cardiovascular**						
Arrhythmia	1,655 (1.1)	656 (1.2)	48 (0.9)	67 (1.2)	323 (1.1)	20 (1.3)
Cardiomyopathy	608 (0.4)	230 (0.4)	19 (0.3)	26 (0.5)	130 (0.5)	3 (0.2)
CHD	7,402 (5.3)	2,877 (5.2)	294 (5.4)	293 (5.3)	1,502 (5.3)	82 (5.3)
Heart failure	3,551 (2.4)	1,431 (2.5)	127 (2.3)	155 (2.7)	753 (2.6)	56 (3.5)
**Mental Health**						
Depression	6,415 (4.8)	2430 (4.6)	269 (5.2)	279 (5.2)	1317 (4.9)	64 (4.3)

Incident disease numbers exclude prevalent disease at baseline. Numbers presented are n (%). Abbreviations: CHD, coronary heart disease; T1, type 1; T2, type 2; LRTI, lower respiratory tract infection; URTI, upper respiratory tract infection; UTI, urinary tract infection; SSTI, skin and soft tissue infection. Joint replacement surgery variable includes a diagnosis of hip, knee, ankle, or shoulder replacement. Any brain outcome variable includes a diagnosis of dementia, delirium, or Parkinson's disease.

For peer review only - http://bmjopen.bmj.com/site/about/guidelines.xhtml

eTable 10. Hazard ratios of incident disease outcomes in p.C282Y/H63D genotypes in females

Females	No mutations	H63D +/-		H63D +/+		C282Y +/ H36D +		C282Y +/-		C282Y +/+	
		HR (95% CI)	P	HR (95% CI)	P	HR (95% CI)	P	HR (95% CI)	P	HR (95% CI)	P
Hemochromatosis	1	2.04 (1.31-3.17)	$1.70*10^{-03}$	7.10 (3.75-13.45)	$1.80*10^{-09}$	32.78 (22.10-48.63)	$2.20*10^{-67}$	4.15 (2.68-6.44)	$1.80*10^{-10}$	674.10 (489.11-929.05)	$2.47*10^{-346}$
All-cause mortality	1	0.99 (0.96-1.03)	0.68	0.95 (0.85-1.05)	0.3	1.01 (0.91-1.11)	0.9	1.01 (0.96-1.06)	0.62	1.10 (0.92-1.31)	0.28
**Liver**											
Liver disease (any)	1	1.01 (0.95-1.07)	0.72	1.00 (0.84-1.17)	0.96	1.19 (1.02-1.38)	0.03	1.08 (1.00-1.16)	0.06	1.62 (1.27-2.05)	$7.80*10^{-05}$
Alcoholic liver disease	1	1.09 (0.84-1.43)	0.51	1.56 (0.85-2.87)	0.15	0.78 (0.35-1.77)	0.56	1.53 (1.14-2.05)	0.005	3.07 (1.44-6.54)	0.004
Fibrosis & Cirrhosis	1	0.87 (0.73-1.04)	0.13	0.87 (0.53-1.43)	0.58	1.10 (0.71-1.71)	0.67	0.92 (0.74-1.16)	0.49	2.56 (1.50-4.36)	0.001
Hepatic failure	1	0.77 (0.58-1.02)	0.07	0.92 (0.43-1.94)	0.82	0.49 (0.18-1.33)	0.16	0.85 (0.59-1.21)	0.37	2.09 (0.86-5.08)	0.1
**Cancer**											
Liver cancer	1	1.26 (1.01-1.58)	0.04	1.13 (0.60-2.13)	0.71	0.89 (0.44-1.81)	0.75	1.06 (0.78-1.45)	0.69	1.17 (0.37-3.66)	0.79
**Musculoskeletal**											
Joint replacement surgery (any)	1	0.99 (0.96-1.02)	0.55	0.97 (0.88-1.06)	0.47	1.04 (0.95-1.14)	0.34	0.99 (0.94-1.03)	0.51	1.18 (1.01-1.38)	0.04
Osteoarthritis	1	0.99 (0.94-1.05)	0.71	1.06 (0.91-1.23)	0.46	1.10 (0.95-1.27)	0.21	1.02 (0.95-1.10)	0.6	1.44 (1.14-1.82)	0.002
Fractures (any)	1	1.01 (0.97-1.04)	0.65	0.91 (0.82-1.01)	0.07	1.01 (0.92-1.12)	0.79	1.02 (0.97-1.07)	0.42	1.00 (0.84-1.20)	0.96
Fragility fractures	1	1.02 (0.98-1.07)	0.38	0.87 (0.76-1.00)	0.05	1.10 (0.97-1.24)	0.13	0.99 (0.93-1.05)	0.74	0.85 (0.66-1.09)	0.19
Osteoporosis	1	0.94 (0.90-0.99)	0.01	0.94 (0.83-1.07)	0.34	0.89 (0.79-1.01)	0.08	0.99 (0.94-1.05)	0.75	1.28 (1.06-1.56)	0.01
Rheumatoid arthritis	1	0.96 (0.88-1.04)	0.28	0.94 (0.75-1.17)	0.58	1.01 (0.82-1.26)	0.9	0.89 (0.80-0.99)	0.03	1.04 (0.71-1.53)	0.84
**Brain**											
Any brain outcome (dementia, delirium, or Parkinson's disease)	1	1.02 (0.97-1.09)	0.43	0.93 (0.79-1.11)	0.43	1.03 (0.88-1.21)	0.72	1.00 (0.92-1.08)	0.9	1.30 (1.01-1.68)	0.04
Dementia	1	1.00 (0.92-1.08)	0.94	0.80 (0.63-1.02)	0.07	1.00 (0.81-1.25)	0.98	1.01 (0.91-1.12)	0.86	1.16 (0.81-1.66)	0.41
Alzheimer's disease	1	1.08 (0.97-1.20)	0.18	0.87 (0.63-1.20)	0.4	1.22 (0.92-1.61)	0.17	1.02 (0.88-1.17)	0.84	1.04 (0.62-1.77)	0.88

For peer review only

For peer review only - http://bmjopen.bmj.com/site/about/guidelines.xhtml

Non-Alzheimer's dementia	1	0.93 (0.83-1.04)	0.2	0.72 (0.50-1.03)	0.07	0.81 (0.57-1.13)	0.21	1.03 (0.89-1.18)	0.73	1.27 (0.76-2.05)	0.33
Delirium	1	1.06 (0.98-1.15)	0.13	1.05 (0.84-1.31)	0.67	1.06 (0.85-1.32)	0.6	1.01 (0.91-1.13)	0.8	1.50 (1.08-2.08)	0.02
Parkinson's disease	1	0.93 (0.80-1.07)	0.29	0.97 (0.66-1.44)	0.89	1.14 (0.79-1.65)	0.48	0.98 (0.81-1.18)	0.84	1.00 (0.50-2.00)	0.99
**Pancreas**											
T1 or T2 diabetes	1	1.01 (0.97-1.06)	0.55	0.98 (0.86-1.11)	0.77	1.09 (0.97-1.23)	0.17	1.01 (0.96-1.08)	0.6	1.18 (0.95-1.46)	0.13
**Infection**											
COVID-19	1	1.02 (0.94-1.11)	0.66	1.03 (0.81-1.30)	0.83	1.02 (0.80-1.29)	0.9	1.06 (0.94-1.18)	0.34	1.00 (0.65-1.54)	0.99
Cholecystitis	1	0.98 (0.90-1.08)	0.73	0.92 (0.71-1.18)	0.52	0.99 (0.78-1.26)	0.92	0.99 (0.88-1.11)	0.86	1.49 (1.04-2.13)	0.03
Pneumonia	1	1.01 (0.96-1.05)	0.83	1.02 (0.90-1.16)	0.72	1.06 (0.93-1.20)	0.4	1.03 (0.97-1.10)	0.29	1.05 (0.83-1.32)	0.69
Sepsis	1	0.98 (0.94-1.01)	0.23	0.97 (0.88-1.08)	0.61	1.05 (0.95-1.15)	0.36	1.04 (0.99-1.09)	0.12	1.13 (0.96-1.35)	0.15
LRTI	1	0.95 (0.89-1.01)	0.08	0.92 (0.78-1.09)	0.34	0.93 (0.79-1.10)	0.39	1.04 (0.97-1.12)	0.3	1.16 (0.89-1.52)	0.28
URTI	1	1.12 (0.98-1.30)	0.1	0.86 (0.56-1.33)	0.51	0.84 (0.54-1.30)	0.43	1.12 (0.93-1.34)	0.24	0.28 (0.07-1.12)	0.07
UTI	1	0.97 (0.94-1.01)	0.18	0.99 (0.89-1.10)	0.82	1.01 (0.91-1.13)	0.8	1.03 (0.98-1.08)	0.23	1.19 (0.99-1.41)	0.06
SSTI	1	1.01 (0.95-1.07)	0.79	1.05 (0.90-1.23)	0.54	1.00 (0.85-1.18)	0.97	1.03 (0.95-1.11)	0.52	1.20 (0.92-1.58)	0.18
**Cardiovascular**											
Arrhythmia	1	1.02 (0.93-1.11)	0.69	0.76 (0.57-1.01)	0.06	1.04 (0.81-1.33)	0.75	0.98 (0.87-1.11)	0.78	1.07 (0.69-1.67)	0.75
Cardiomyopathy	1	0.97 (0.83-1.13)	0.71	0.82 (0.52-1.30)	0.41	1.11 (0.75-1.64)	0.61	1.08 (0.90-1.31)	0.41	0.44 (0.14-1.37)	0.16
CHD	1	1.00 (0.95-1.04)	0.82	1.03 (0.92-1.16)	0.61	1.01 (0.90-1.14)	0.86	1.01 (0.96-1.07)	0.71	0.94 (0.76-1.17)	0.6
Heart failure	1	1.04 (0.97-1.10)	0.27	0.92 (0.77-1.10)	0.35	1.12 (0.96-1.32)	0.15	1.06 (0.98-1.15)	0.16	1.34 (1.03-1.75)	0.03
**Mental Health**											
Depression	1	0.97 (0.93-1.02)	0.2	1.10 (0.97-1.24)	0.14	1.11 (0.99-1.25)	0.09	1.03 (0.97-1.09)	0.38	0.90 (0.70-1.15)	0.39

HR (Hazard ratio) compared to those with neither *HFE* mutation. Cox proportional hazards regression models adjusted for age, assessment centre, and genetic principal components 1–10. Abbreviations: CHD, coronary heart disease; T1, type 1; T2, type 2; LRTI, lower respiratory tract infection; URTI, upper respiratory tract infection; UTI, urinary tract infection SSTI, skin and soft tissue infection; CI, confidence interval. Joint replacement surgery variable includes a diagnosis of hip, knee, ankle, or shoulder replacement. Any brain outcome variable includes a diagnosis of dementia, delirium, or Parkinson's disease.

For peer review only

For peer review only - http://bmjopen.bmj.com/site/about/guidelines.xhtml

eTable 11. Incident hospital diagnoses in female UK Biobank participants by p.C282Y/H63D genotypes, excluding a diagnosis of hemochromatosis at baseline

Females	No mutations	H63D +/-	H63D +/+	C282Y +/ H36D +	C282Y +/-	C282Y +/+
Hemochromatosis	44 (9.24)	35 (7.35)	12 (2.52)	57 (11.97)	37 (7.77)	291 (61.13)
All-cause mortality	9847 (59.47)	3810 (23.01)	363 (2.19)	390 (2.36)	2020 (12.20)	127 (0.77)
**Liver**						
Liver disease (any)	3821 (58.38)	1511 (23.09)	146 (2.23)	177 (2.70)	825 (12.61)	65 (0.99)
Alcoholic liver disease	180 (52.94)	78 (22.94)	11 (3.24)	6 (1.76)	59 (17.35)	6 (1.76)
Fibrosis & Cirrhosis	473 (61.03)	162 (20.90)	16 (2.06)	21 (2.71)	89 (11.48)	14 (1.81)
Hepatic failure	199 (64.40)	60 (19.42)	7 (2.27)	4 (1.29)	35 (11.33)	4 (1.29)
**Cancer**						
Liver cancer	232 (55.77)	114 (27.40)	10 (2.40)	8 (1.92)	49 (11.78)	3 (0.72)
**Musculoskeletal**						
Joint replacement surgery (any)	12355 (59.84)	4771 (23.11)	454 (2.20)	500 (2.42)	2415 (11.70)	150 (0.73)
Osteoarthritis	4343 (59.08)	1681 (22.87)	177 (2.41)	189 (2.57)	893 (12.15)	68 (0.93)
Fractures (any)	10665 (59.31)	4204 (23.38)	375 (2.09)	430 (2.39)	2188 (12.17)	119 (0.66)
Fragility fractures	6308 (59.29)	2517 (23.66)	214 (2.01)	275 (2.58)	1264 (11.88)	61 (0.57)
Osteoporosis	6990 (60.50)	2580 (22.33)	254 (2.20)	244 (2.11)	1389 (12.02)	97 (0.84)
Rheumatoid arthritis	2188 (61.05)	818 (22.82)	79 (2.20)	86 (2.40)	390 (10.88)	23 (0.64)
**Brain**						
Any brain outcome (dementia, delirium, or Parkinson's disease)	3875 (59.16)	1547 (23.62)	142 (2.17)	154 (2.35)	777 (11.86)	55 (0.84)
Dementia	2159 (59.72)	837 (23.15)	68 (1.88)	83 (2.30)	440 (12.17)	28(0.77)
Alzheimer's disease	1072 (58.20)	450 (24.43)	37 (2.01)	50 (2.71)	220 (11.94)	13 (0.71)
Non-Alzheimer's dementia	1099 (61.02)	395 (21.93)	31 (1.72)	34(1.89)	227 (12.60)	15 (0.83)
Delirium	1982 (58.17)	823 (24.16)	81 (2.38)	82 (2.41)	406 (11.92)	33 (0.97)

Parkinson's disease	691 (60.72)	249 (21.88)	26 (2.28)	30 (2.64)	134 (11.78)	8 (0.70)
**Pancreas**						
T2 diabetes	6538 (59.27)	2575 (23.35)	240 (2.18)	275 (2.49)	1322 (11.99)	80 (0.73)
**Infection**						
Covid-19	1809 (58.83)	719 (23.38)	72 (2.34)	72 (2.34)	383 (12.46)	20 (0.65)
Cholecystitis	1749 (59.61)	675 (23.01)	62 (2.11)	68 (2.32)	351 (11.96)	29 (0.99)
Pneumonia	6127 (59.06)	2406 (23.19)	242 (2.33)	254 (2.45)	1275 (12.29)	70 (0.67)
Sepsis	10024 (59.37)	3837 (22.72)	379 (2.24)	414 (2.45)	2103 (12.45)	128 (0.76)
LRTI	3875 (59.94)	1437 (22.23)	139 (2.15)	143 (2.21)	821 (12.70)	50 (0.77)
URTI	636 (57.87)	277 (25.20)	21 (1.91)	21 (1.91)	142 (12.92)	2 (0.18)
UTI	9137 (59.45)	3490 (22.71)	351 (2.28)	366 (2.38)	1905 (12.40)	119 (0.77)
SSTI	3900 (59.15)	1533 (23.25)	158 (2.40)	152 (2.31)	799 (12.12)	51 (0.77)
**Cardiovascular**						
Arrhythmia	1654 (59.75)	656 (23.70)	48 (1.73)	67 (2.42)	323 (11.67)	20 (0.72)
Cardiomyopathy	608 (59.90)	230 (22.66)	19 (1.87)	25 (2.46)	130 (12.81)	3 (0.30)
CHD	7401 (59.49)	2875 (23.11)	294 (2.36)	291 (2.34)	1502 (12.07)	77 (0.62)
Heart failure	3550 (58.50)	1430 (23.57)	127 (2.09)	154 (2.54)	753 (12.41)	54 (0.89)
**Mental Health**						
Depression	6414 (59.58)	2430 (22.57)	269 (2.50)	278 (2.58)	1317 (12.23)	57 (0.53)

Incident disease numbers exclude prevalent disease at baseline. Numbers presented are n (%). Abbreviations: CHD, coronary heart disease; T1, type 1; T2, type 2; LRTI, lower respiratory tract infection; URTI, upper respiratory tract infection; UTI, urinary tract infection; SSTI, skin and soft tissue infection. Joint replacement surgery variable includes a diagnosis of hip, knee, ankle, or shoulder replacement. Any brain outcome variable includes a diagnosis of dementia, delirium, or Parkinson's disease.

For peer review only - http://bmjopen.bmj.com/site/about/guidelines.xhtml

eTable 12. Hazard ratios of incident disease outcomes in p.C282Y/H63D genotypes in females, excluding a diagnosis of hemochromatosis at baseline

**Females**	**No mutations (Ref group)**	**H63D +/-**		**H63D +/+**		**C282Y +/ H36D +**		**C282Y +/-**		**C282Y +/+**	
		HR (95% CI)	P	HR (95% CI)	P	HR (95% CI)	P	HR (95% CI)	P	HR (95% CI)	P
Hemochromatosis	1	2.04 (1.31-3.17)	**1.70E-03**	7.10 (3.75-13.45)	**1.80E-09**	32.79 (22.10-48.64)	**2.10E-67**	4.15 (2.68-6.44)	**1.80E-10**	675.18 (489.89-930.55)	**0.00E+00**
All-cause mortality	1	0.99 (0.96-1.03)	0.69	0.95 (0.85-1.05)	0.31	1.00 (0.90-1.11)	0.98	1.01 (0.97-1.06)	0.61	1.13 (0.95-1.35)	0.16
**Liver**											
Liver disease (any)	1	1.01 (0.95-1.07)	0.73	1.00 (0.84-1.18)	0.97	1.18 (1.01-1.37)	**0.03**	1.08 (1.00-1.16)	0.06	1.58 (1.23-2.02)	**2.80E-04**
Alcoholic liver disease	1	1.10 (0.84-1.43)	0.48	1.57 (0.85-2.88)	0.15	0.79 (0.35-1.78)	0.57	1.54 (1.14-2.06)	**0.43**	2.75 (1.21-6.20)	**0.02**
Fibrosis & Cirrhosis	1	0.87 (0.73-1.04)	0.13	0.87 (0.53-1.44)	0.60	1.11 (0.72-1.72)	0.65	0.93 (0.74-1.16)	0.51	2.67 (1.57-4.55)	**3.00E-04**
Hepatic failure	1	0.77 (0.58-1.03)	0.08	0.92 (0.43-1.95)	0.83	0.50 (0.18-1.34)	0.17	0.85 (0.59-1.22)	0.38	1.76 (0.65-4.74)	0.26
**Cancer**											
Liver cancer	1	1.26 (1.10-1.58)	**0.04**	1.13 (0.60-2.13)	0.71	0.89 (0.44-1.81)	0.75	1.06 (0.78-1.45)	0.69	1.22 (0.39-3.81)	0.73
**Musculoskeletal**											
Joint replacement surgery (any)	1	0.99 (0.96-1.02)	0.54	0.97 (0.88-1.06)	0.47	1.04 (0.95-1.14)	0.35	0.99 (0.94-1.03)	0.51	1.16 (0.99-1.37)	**0.07**
Osteoarthritis	1	0.99 (0.94-1.05)	0.72	1.06 (0.91-1.23)	0.46	1.09 (0.95-1.27)	0.22	1.02 (0.95-1.10)	0.59	1.46 (1.15-1.85)	**2.10E-03**
Fractures (any)	1	1.01 (0.97-1.05)	0.65	0.91 (0.82-1.01)	0.07	1.02 (0.92-1.12)	0.76	1.02 (0.97-1.07)	0.42	1.01 (0.84-1.20)	0.95
Fragility fractures	1	1.02 (0.98-1.07)	0.37	0.87 (0.76-1.00)	**0.05**	1.10 (0.98-1.24)	0.12	0.99 (0.93-1.05)	0.73	0.86 (0.67-1.10)	0.23
Osteoporosis	1	0.94 (0.90-0.99)	**0.01**	0.94 (0.83-1.07)	0.34	0.89 (0.78-1.01)	0.08	0.99 (0.93-1.05)	0.73	1.26 (1.03-1.54)	**0.02**
Rheumatoid arthritis	1	0.96 (0.88-1.04)	0.28	0.94 (0.75-1.17)	0.58	1.00 (0.81-1.25)	0.97	0.89 (0.80-0.99)	**0.03**	0.96 (0.63-1.44)	0.83
**Brain**											
Any brain outcome (dementia, delirium, or Parkinson's disease)	1	1.02 (0.97-1.09)	0.43	0.93 (0.79-1.11)	0.43	1.03 (0.87-1.20)	0.76	1.00 (0.92-1.08)	0.91	1.23 (0.94-1.61)	0.13
Dementia	1	1.00 (0.92-1.08)	0.94	0.80 (0.63-1.02)	0.07	0.99 (0.80-1.24)	0.95	1.01 (0.91- 1.12)	0.86	1.10 (0.76-1.60)	0.61
Alzheimer's disease	1	1.08 (0.97-1.20)	0.18	0.86 (0.63-1.21)	0.40	1.20 (0.90-1.59)	0.21	1.02 (0.88-1.17)	0.84	1.02 (0.59-1.76)	0.95

For peer review only - http://bmjopen.bmj.com/site/about/guidelines.xhtml

Non-Alzheimer's dementia	1	0.93 (0.83-1.04)	0.20	0.72 (0.50-1.03)	0.07	0.81 (0.57-1.14)	0.22	1.03 (0.89-1.18)	0.73	1.17 (0.71-1.96)	0.54
Delirium	1	1.07 (0.98-1.16)	0.13	1.05 (0.84-1.31)	0.67	1.06 (0.85-1.33)	0.58	1.01 (0.91-1.13)	0.79	1.44 (1.02-2.03)	**0.04**
Parkinson's disease	1	0.93 (0.80-1.07)	0.29	0.97 (0.66-1.44)	0.89	1.15 (0.79-1.65)	0.47	0.98 (0.81-1.18)	0.84	1.04 (0.52-2.10)	0.91
**Pancreas**											
T1 or T2 diabetes	1	1.01 (0.97-1.06)	0.54	0.98 (0.86-1.11)	0.72	1.08 (0.96-1.22)	0.20	1.02 (0.96-1.08)	0.59	1.13 (0.90-1.41)	0.28
**Infection**											
COVID-19	1	1.02 (0.93-1.11)	0.68	1.03 (0.81-1.30)	0.83	1.02 (0.80-1.29)	0.88	1.06 (0.94-1.18)	0.34	1.00 (0.64-1.56)	1.00
Cholecystitis	1	0.98 (0.90-1.08)	0.73	0.92 (0.71-1.19)	0.52	0.98 (0.77-1.24)	0.84	0.99 (0.88-1.11)	0.86	1.49 (1.03-2.16)	**0.03**
Pneumonia	1	1.00 (0.96-1.05)	0.84	1.02 (0.90-1.17)	0.71	1.05 (0.93-1.20)	0.41	1.03 (0.97-1.10)	0.29	1.02 (0.81-1.29)	0.85
Sepsis	1	0.98 (0.94-1.01)	0.22	0.97 (0.88-1.08)	0.62	1.05 (0.95-1.16)	0.34	1.04 (0.99-1.09)	0.11	1.14 (0.96-1.36)	0.14
LRTI	1	0.95 (0.89-1.01)	0.07	0.92 (0.78-1.09)	0.34	0.93 (0.78-1.09)	0.36	1.04 (0.97-1.12)	0.29	1.14 (0.86-1.51)	0.35
URTI	1	1.12 (0.97-1.29)	0.13	0.86 (0.56-1.33)	0.51	0.84 (0.54-1.30)	0.43	1.12 (0.93-1.34)	0.24	0.29 (0.07-1.17)	0.08
UTI	1	0.97 (0.94-1.01)	0.17	0.99 (0.89-1.10)	0.82	1.02 (0.92-1.13)	0.76	1.03 (0.98-1.08)	0.22	1.17 (0.97-1.40)	0.10
SSTI	1	1.01 (0.95-1.07)	0.81	1.05 (0.90-1.23)	0.54	1.00 (0.85-1.17)	0.98	1.03 (0.95-1.11)	0.52	1.20 (0.91-1.59)	0.19
**Cardiovascular**											
Arrhythmia	1	1.02 (0.93-1.12)	0.69	0.76 (0.57-1.01)	0.06	1.04 (0.82-1.33)	0.73	0.98 (0.87-1.11)	0.78	1.13 (0.72-1.75)	0.60
Cardiomyopathy	1	0.97 (0.83-1.13)	0.71	0.82 (0.52-1.30)	0.41	1.07 (0.72-1.59)	0.75	1.08 (0.90-1.31)	0.40	0.46 (0.15-1.43)	0.18
CHD	1	0.99 (0.95-1.04)	0.81	1.03 (0.92-1.16)	0.60	1.01 (0.89-1.13)	0.92	1.01 (0.96-1.07)	0.70	0.92 (0.74-1.15)	0.48
Heart failure	1	1.03 (0.97-1.10)	0.28	0.92 (0.77-1.10)	0.36	1.12 (0.95-1.32)	0.17	1.06 (0.98-1.15)	0.15	1.35 (1.03-1.77)	**0.03**
**Mental Health**											
Depression	1	0.97 (0.93-1.02)	0.20	1.10 (0.97-1.23)	0.14	1.11 (0.98-1.25)	0.09	1.03 (0.97-1.09)	0.37	0.83 (0.64-1.08)	0.16

HR (Hazard ratio) compared to those with neither *HFE* mutation. Cox proportional hazards regression models adjusted for age, assessment centre, and genetic principal components 1–10. Abbreviations: CHD, coronary heart disease; T1, type 1; T2, type 2; LRTI, lower respiratory tract infection; URTI, upper respiratory tract infection; UTI, urinary tract infection SSTI, skin and soft tissue infection; CI, confidence interval. Joint replacement surgery variable includes a diagnosis of hip, knee, ankle, or shoulder replacement. Any brain outcome variable includes a diagnosis of dementia, delirium, or Parkinson's disease

For peer review only - http://bmjopen.bmj.com/site/about/guidelines.xhtml

eFigure 1. Kaplan-Meier curves for the cumulative incidence of (a) mortality, (b) liver disease, (c) any joint replacement, and (d) any brain outcome, in male *HFE* p.C282Y homozygotes compared to those with no mutations, excluding a diagnosis of hemochromatosis at baseline

a)

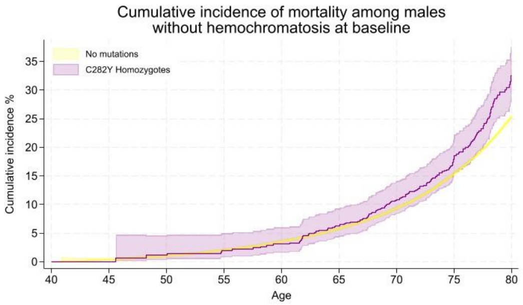

b)

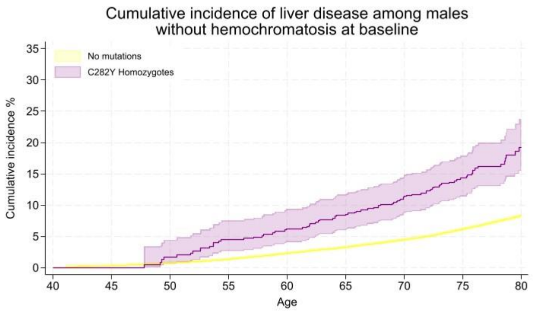

c)

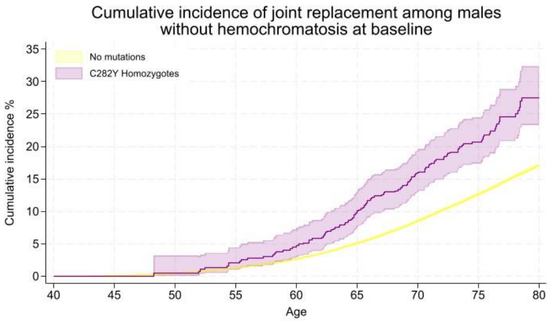

d)

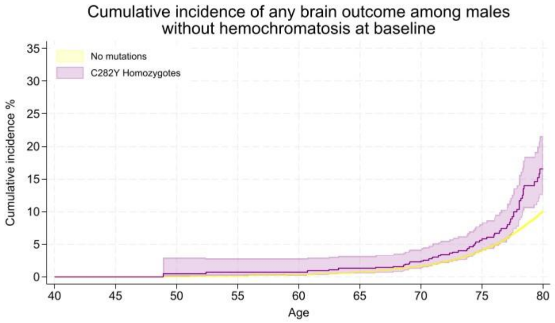

Cumulative incidence (estimated % diagnosed by age 80 years; 95% CIs). Joint replacement surgery includes a diagnosis of hip, knee, ankle, or shoulder replacement. Any brain outcome included a diagnosis of delirium, dementia, or Parkinson's disease.

For peer review only - http://bmjopen.bmj.com/site/about/guidelines.xhtml

eFigure 2. Kaplan-Meier curves for the cumulative incidence of (a) liver disease, and (b) any joint replacement, in female *HFE* p.C282Y homozygotes compared to those with no mutations, excluding a diagnosis of hemochromatosis at baseline

a) b)

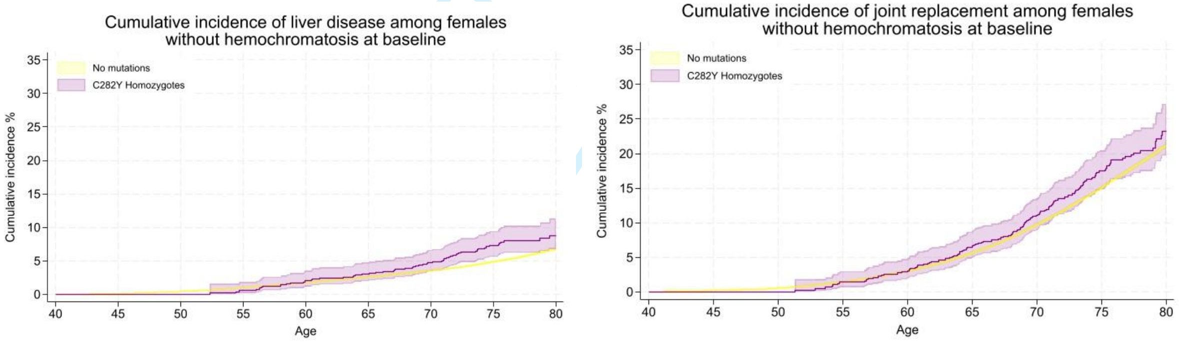

Cumulative incidence (estimated % diagnosed by age 80 years; 95% CIs). Joint replacement surgery includes a diagnosis of hip, knee, ankle, or shoulder replacement.

STROBE 2007 (v4) Statement—Checklist of items that should be included in reports of *cohort studies*

Section/Topic	Item #	Recommendation	Reported on page #
**Title and abstract**	1	(*a*) Indicate the study's design with a commonly used term in the title or the abstract	1-2
		(*b*) Provide in the abstract an informative and balanced summary of what was done and what was found	2
**Introduction**			
Background/rationale	2	Explain the scientific background and rationale for the investigation being reported	4-5
Objectives	3	State specific objectives, including any prespecified hypotheses	5
**Methods**			
Study design	4	Present key elements of study design early in the paper	5
Setting	5	Describe the setting, locations, and relevant dates, including periods of recruitment, exposure, follow-up, and data collection	5-6
Participants	6	(*a*) Give the eligibility criteria, and the sources and methods of selection of participants. Describe methods of follow-up	5-6
		(*b*) For matched studies, give matching criteria and number of exposed and unexposed	N/A
Variables	7	Clearly define all outcomes, exposures, predictors, potential confounders, and effect modifiers. Give diagnostic criteria, if applicable	5-6
Data sources/ measurement	8*	For each variable of interest, give sources of data and details of methods of assessment (measurement). Describe comparability of assessment methods if there is more than one group	*5-6*
Bias	9	Describe any efforts to address potential sources of bias	5
Study size	10	Explain how the study size was arrived at	5
Quantitative variables	11	Explain how quantitative variables were handled in the analyses. If applicable, describe which groupings were chosen and why	5-6
Statistical methods	12	(*a*) Describe all statistical methods, including those used to control for confounding	6
		(*b*) Describe any methods used to examine subgroups and interactions	6
		(*c*) Explain how missing data were addressed	N/A
		(*d*) If applicable, explain how loss to follow-up was addressed	N/A
		(*e*) Describe any sensitivity analyses	6
**Results**			

For peer review only

Participants	13*	(a) Report numbers of individuals at each stage of study—eg numbers potentially eligible, examined for eligibility, confirmed eligible, included in the study, completing follow-up, and analysed	6-7
		(b) Give reasons for non-participation at each stage	N/A
		(c) Consider use of a flow diagram	N/A
Descriptive data	14*	(a) Give characteristics of study participants (eg demographic, clinical, social) and information on exposures and potential confounders	6-7, 18<br>*Supplement Material* pages: 3-4
		(b) Indicate number of participants with missing data for each variable of interest	N/A
		(c) Summarise follow-up time (eg, average and total amount)	6
Outcome data	15*	Report numbers of outcome events or summary measures over time	*Supplement Material,* pages 5-6, 9-10, 14-15, 18-19
Main results	16	(*a*) Give unadjusted estimates and, if applicable, confounder-adjusted estimates and their precision (eg, 95% confidence interval). Make clear which confounders were adjusted for and why they were included	6-9<br>*Supplement Material,* pages: 7-8, 11-12, 13, 16-17, 20-21
		(*b*) Report category boundaries when continuous variables were categorized	N/A
		(*c*) If relevant, consider translating estimates of relative risk into absolute risk for a meaningful time period	N/A
Other analyses	17	Report other analyses done—eg analyses of subgroups and interactions, and sensitivity analyses	7
**Discussion**			
Key results	18	Summarise key results with reference to study objectives	10
**Limitations**			10-12
Interpretation	20	Give a cautious overall interpretation of results considering objectives, limitations, multiplicity of analyses, results from similar studies, and other relevant evidence	10-12
Generalisability	21	Discuss the generalisability (external validity) of the study results	10-12
**Other information**			
Funding	22	Give the source of funding and the role of the funders for the present study and, if applicable, for the original study on which the present article is based	13

*Give information separately for cases and controls in case-control studies and, if applicable, for exposed and unexposed groups in cohort and cross-sectional studies.

For peer review only

Note: An Explanation and Elaboration article discusses each checklist item and gives methodological background and published examples of transparent reporting. The STROBE checklist is best used in conjunction with this article (freely available on the Web sites of PLoS Medicine at http://www.plosmedicine.org/, Annals of Internal Medicine at http://www.annals.org/, and Epidemiology at http://www.epidem.com/). Information on the STROBE Initiative is available at www.strobe-statement.org.

For peer review only

BMJ Open

HFE genotypes, haemochromatosis diagnosis and clinical outcomes to age 80: a prospective cohort study in UK Biobank

Journal:	*BMJ Open*
Manuscript ID	bmjopen-2023-081926.R2
Article Type:	Original research
Date Submitted by the Author:	17-Jan-2024
Complete List of Authors:	Lucas, Mitchell; University of Exeter<br>Atkins, Janice; University of Exeter<br>Pilling, Luke; University of Exeter, Epidemiology and Public Health<br>Shearman, Jeremy; South Warwickshire University NHS Foundation Trust<br>Melzer, David; University of Exeter
**Primary Subject Heading**:	Epidemiology
Secondary Subject Heading:	Genetics and genomics
Keywords:	GENETICS, Other metabolic, e.g. iron, porphyria < DIABETES & ENDOCRINOLOGY, Mortality, Hepatology < INTERNAL MEDICINE

SCHOLARONE™
Manuscripts

I, the Submitting Author has the right to grant and does grant on behalf of all authors of the Work (as defined in the below author licence), an exclusive licence and/or a non-exclusive licence for contributions from authors who are: i) UK Crown employees; ii) where BMJ has agreed a CC-BY licence shall apply, and/or iii) in accordance with the terms applicable for US Federal Government officers or employees acting as part of their official duties; on a worldwide, perpetual, irrevocable, royalty-free basis to BMJ Publishing Group Ltd ("BMJ") its licensees and where the relevant Journal is co-owned by BMJ to the co-owners of the Journal, to publish the Work in this journal and any other BMJ products and to exploit all rights, as set out in our licence.

The Submitting Author accepts and understands that any supply made under these terms is made by BMJ to the Submitting Author unless you are acting as an employee on behalf of your employer or a postgraduate student of an affiliated institution which is paying any applicable article publishing charge ("APC") for Open Access articles. Where the Submitting Author wishes to make the Work available on an Open Access basis (and intends to pay the relevant APC), the terms of reuse of such Open Access shall be governed by a Creative Commons licence – details of these licences and which Creative Commons licence will apply to this Work are set out in our licence referred to above.

Other than as permitted in any relevant BMJ Author's Self Archiving Policies, I confirm this Work has not been accepted for publication elsewhere, is not being considered for publication elsewhere and does not duplicate material already published. I confirm all authors consent to publication of this Work and authorise the granting of this licence.

For peer review only

HFE genotypes, haemochromatosis diagnosis and clinical outcomes to age 80: a prospective cohort study in UK Biobank

Mitchell R Lucas[1]*, Janice L Atkins[1]*, Luke C Pilling[1], Jeremy Shearman[2], David Melzer[1]**

[1]Epidemiology and Public Health Group, Department of Clinical and Biomedical Sciences, Faculty of Health and Life Sciences, University of Exeter, UK

[2]Department of Gastroenterology, South Warwickshire University NHS Foundation Trust, UK

*Contributed equally to this study

Corresponding author: David Melzer, Professor of Epidemiology and Public Health, The Department of Clinical and Biomedical Sciences, Faculty of Health and Life Sciences, University of Exeter, St Luke's Campus, College House, Magdalen Road, Exeter, Devon, EX1 2LU, UK. E-mail: d.melzer@exeter.ac.uk

Keywords: Haemochromatosis; *HFE* p.C282Y / p.H63D genotypes; iron overload; morbidity; mortality; UK Biobank

Word count: 3,950

References: 43

Tables: 1

Figures: 4

For peer review only - http://bmjopen.bmj.com/site/about/guidelines.xhtml

For peer review only

Abstract

Objectives: *HFE* haemochromatosis genetic variants have an uncertain clinical penetrance, especially to older ages and in undiagnosed groups. We estimated p.C282Y and p.H63D variant cumulative incidence of multiple clinical outcomes in a large community cohort.

Design: Prospective cohort study.

Setting: 22 assessment centres across England, Scotland, and Wales in the UK Biobank (2006-2010).

Participants: 451,270 participants genetically similar to the 1000-Genomes European reference population, with a mean 13.3-year follow-up through hospital inpatient, cancer registries and death certificate data.

Main outcome measures: Cox proportional hazard ratios of incident clinical outcomes and mortality in those with *HFE* p.C282Y-p.H63D mutations compared to those with no variants, stratified by sex and adjusted for age, assessment centre and genetic stratification. Cumulative incidences were estimated from age 40 to 80 years.

Results: 12.1% of p.C282Y+/+ males had baseline (mean age 57) haemochromatosis diagnoses, with age 80 cumulative incidence of 56.4%. 33.1% died vs. 25.4% without *HFE* variants (Hazard Ratio [HR] 1.29, 95% CI: 1.12-1.48, $p=4.7*10^{-4}$); 27.9% vs 17.1% had joint replacements, 20.3% vs 8.3% had liver disease, and there was excess delirium, dementia, and Parkinson's disease, but not depression. Associations, including excess mortality, were similar in the group undiagnosed with haemochromatosis. 3.4% of p.C282Y+/+ females had baseline haemochromatosis diagnoses, with cumulative age 80 incidence of 40.5%. There was excess incident liver disease (8.9% vs 6.8%; HR 1.62, 95% CI: 1.27-2.05, $p=7.8*10^{-5}$), joint replacements and delirium, with similar results in the undiagnosed. p.C282Y/p.H63D and p.H63D+/+ men or women had no statistically significant excess fatigue or depression at baseline and no excess incident outcomes.

Conclusions: Male and female p.C282Y homozygotes experienced greater excess morbidity than previously documented, including those undiagnosed with haemochromatosis in the community. As haemochromatosis diagnosis rates were low at baseline despite treatment being considered effective, trials of screening to identify people with p.C282Y homozygosity early appear justified.

For peer review only - http://bmjopen.bmj.com/site/about/guidelines.xhtml

Strengths and limitations of this study

- We analyzed largescale data on community volunteers from the UK Biobank, one of the world's largest *HFE* genotyped cohorts.
- We have analyzed incident disease outcomes during an extended follow-up period of mean 13.3 years.
- We have provided the first clinical outcome data to age 80 years in those with haemochromatosis genotypes, including those undiagnosed with haemochromatosis at baseline, expanding the life-course evidence on *HFE* penetrance.
- UK Biobank participants were somewhat healthier than the general population, but *HFE* allele frequencies were similar to previous UK studies.
- Incident outcomes were from hospital inpatient and cancer registry follow-up, so did not rely on potentially biased patient self-reporting, but community diagnosed conditions may be underestimated.

For peer review only

For peer review only - http://bmjopen.bmj.com/site/about/guidelines.xhtml

Introduction

HFE haemochromatosis is defined by iron overload[1] [2] due to gene variants, which dysregulate intestinal iron absorption. The p.C282Y+/+ (homozygous) group have markedly raised iron measures: e.g. median transferrin saturations were over 80% and near 60% in p.C282Y+/+ males and females in HEIRS study, but below 45% in compound heterozygotes (C282Y+/H36D+), and progressively lower across p.H63D+/+ (homozygote), p.C282Y+/- and p.H63D+/- carriers[3]. Women with each genotype have lower mean iron measures than men[4].

Clinical presentation of haemochromatosis is usually with fatigue, joint pain, raised iron measures or from family screening, or less commonly from direct-to-consumer genotyping. Symptoms usually present after age 40 years[5]. In severely affected p.C282Y+/+ patients (>90% of typical cases[6]), liver iron deposition can lead to liver fibrosis, cirrhosis and cancer[7], especially in the presence of other causes of liver disease. Arthropathy[8], diabetes[9], endocrine dysregulation[10], heart arrhythmias and cardiomyopathies[11], and pneumonia[9] have also been reported.

While clinical cohorts frequently have iron overload complications, disease penetrance in population genotyped groups is uncertain, especially at older ages and those not diagnosed with haemochromatosis[3] [12] [13]. Beutler et al found negligible haemochromatosis symptoms in 152 p.C282Y homozygotes from California health appraisal clinics (excluding diagnosed patients)[12] . The HEIRS Study reported excess liver disease in 299 p.C282Y+/+ males[14]. The Melbourne Collaborative Cohort Study[15] reported that 28.4% (95% Confidence Interval [95% CI], 18.8% to 40.2%) of p.C282Y+/+ males (n=95, mean age 65 at follow-up) had 'documented iron-overload-related disease', with 1.2% of p.C282Y+/+ females affected. Similarly, although excess mortality occurred in clinical patients (especially with liver disease)[16][17], no excess mortality was reported in community-identified p.C282Y+/+ males or other *HFE* genotype groups[15] [18]. Using UK Biobank, we previously examined data on European ancestry community participants with mean 7-year follow-up, finding the 1,294 male p.C282Y homozygotes had increased odds of liver disease and osteoarthritis compared to those without p.C282Y or p.H63D variants[9]. A UK Biobank 8.9-year follow-up quantified excess hepatic malignancies in male p.C282Y homozygotes (HR 10.5; 95% CI: 6.6-16.7; p<0.001 versus no *HFE* variant) and excess all-cause mortality (n=88 deaths; HR 1.2; 95% CI: 1.0-1.5; p=0.046)[7].

For p.C282Y/H63D compound heterozygotes, the Melbourne Collaborative Cohort Study[15] found only one male (of 242 studied) with documented iron overload-related disease, although alcohol was also a

For peer review only - http://bmjopen.bmj.com/site/about/guidelines.xhtml

factor. Similarly, a study of community identified participants with p.H63D variants from the Busselton study (Australia) found none with clinically significant iron overloading[19].

Given the accumulating evidence of significant clinical penetrance with p.C282Y homozygosity (only) and the reported effectiveness of treatment (predominantly venesection), there is renewed interest in screening for those at high risk[1] [20] [21]. *HFE* p.C282Y homozygosity is recommended for reporting to patients when found incidentally[22].

The UK Biobank community cohort includes baseline questionnaires, plus hospital inpatient diagnoses during the mean 13.3-year follow-up. The cohort therefore provides a potential model for community based genetic screening. Participant consent did not allow genotype feedback (see Methods), so outcomes reflect normal clinical care. Here we aimed to estimate risks and cumulative outcomes to age 80 years by genotype and sex for relevant clinical outcomes, including (for the first time) analyses of those undiagnosed with haemochromatosis at baseline.

Methods

Study population

UK Biobank includes community volunteers aged 39 to 73 years at baseline assessments across England, Scotland and Wales, from 2006 to 2010. Participants were somewhat healthier than the general population[23], but *HFE* allele frequencies were similar to previous UK studies[7]. Data cover 451,270 participants, genetically similar to the 1000 Genomes project European reference population[24], with *HFE* p.C282Y (rs1800562) and *HFE* p.H63D (rs1799945) genotypes from whole exome sequencing (Whole Exome Sequence methods were by Regeneron[25]). Participants gave written informed consent and were informed of relevant health related findings at baseline, but consent excluded individual notification of subsequent findings including genotypes. North West Multi-Centre Research Ethics Committee (Research Ethics Committee reference 11/NW/0382) approved UK Biobank. All research was conducted in accordance with both the Declarations of Helsinki and Istanbul.

Baseline variables and incident health outcomes

Baseline questionnaires covered doctor-diagnosed conditions including haemochromatosis. Symptom questions included: “Over the past two weeks, how often have you felt tired or had little energy?”, and responses were coded as ‘fatigue’ combining “more than half the days” and “nearly every day”. Studied

For peer review only - http://bmjopen.bmj.com/site/about/guidelines.xhtml

diagnoses were from *a priori* knowledge (see Supplementary eTable 1 for ascertainment codes for 34 outcomes). England, Wales, and Scotland hospital records were available from April 1996 to October 2022. Prevalent diagnoses were from baseline self-report plus hospital inpatient data from 1996 to baseline. Incident diagnoses and surgical procedures were from hospital inpatient data (baseline to October 2022) plus cancer registries to December 2020 for England and Wales and November 2021 for Scotland. National death records were available to November 2022. Disease ascertainment used International Classification of Diseases 10th revision (ICD-10) codes. Surgical procedures were from OPCS Classification of Interventions and Procedures version 4 (OPCS-4). Having 'any joint replacement surgery' included hip, knee, ankle, or shoulder replacement surgery. The 'any brain outcome' included delirium, dementia, or Parkinson's disease.

Statistical analysis

Cox proportional hazards regression estimated genotype associations with incident outcomes. Models were stratified by sex and adjusted for age, assessment centre and 10 genetic principal components (accounting for population substructure). Main outcome proportional hazards assumptions (tested using 'estat phtest') were met in models adjusted for 5-year age bands. Given the extensive prior evidence for risks in p.C282Y+/+ groups, multiple testing corrections are discussed for lower risk genotypes, using Bonferroni correction of $p<0.001$ (0.05 / 34 disease outcomes). Kaplan-Meier survivor functions estimated the probabilities of cumulative incidence for associated outcomes from age 40 to 80 years within 5-year bands, by *HFE* genotype and by sex. We applied observed incidence rates in each age group to a notional cohort, estimating hypothetical cumulative incident case numbers from age 40 to 80 years. Sensitivity analysis repeated main analyses excluding participants with a haemochromatosis diagnosis at baseline. All analyses were performed in Stata 17.0.

Patient and public involvement

Patients and participants were and are extensively involved in the UK Biobank study itself. We used anonymised data that were already collected and therefore no patients were involved in developing the research question or the outcomes tested. UK Biobank notified participants of relevant health related findings in the baseline assessment, but there is no individual notification of subsequent findings, including genotypes.

For peer review only - http://bmjopen.bmj.com/site/about/guidelines.xhtml

Results

UK Biobank baseline characteristics were previously reported[9] [26] (Table 1, eTables 2-3): 451,270 participants genetically similar to the European 1000 Genomes reference population were followed for a mean 13.3 years. There were 1,298 p.C282Y+/+ (homozygotes), 4,959 p.C282Y/p.H63D compound heterozygote, and 4,673 p.H63D+/+ males: for females 1,604, 5,760 and 5,580 respectively. In the male p.C282Y+/+ group, 12.1% had haemochromatosis diagnoses at baseline, with 6/1,000 diagnosed in p.C282Y/H63D and 2 per 1,000 in the p.H63D+/+ group: the respective rates in females were 3.4%, 2 per 1,000 and 4 per 10,000.

p.C282Y+/+ males aged 60 plus reported baseline excess fatigue (11.8% vs 8.2%; Odds Ratio (OR): 1.43, 95% CI: 1.11-1.85, p=0.01), but there was no statistically significant excess fatigue with other genotypes, except a marginal association in p.H63D+/- males (OR: 1.06, CI 1.00-1.12, p=0.05) which became non-significant after multiple testing correction. There were no differences in depression prevalence at baseline (Table 1, eTables 2-3).

Males with p.C282Y homozygote genotypes: excess mortality and morbidity versus without HFE variants

Figure 1 shows the Hazard ratios (HRs) for studied incident outcomes by sex and genotype, with cumulative incidence (with confidence intervals) to age 80 years presented in Figure 2 for those outcomes which had significant HRs. p.C282Y+/+ males had increased rates of mortality versus those without p.C282Y or p.H63D variants (HR 1.29, 95% CI: 1.12-1.48, $p=4.7*10^{-4}$; Figure 1; eTables 4 and 5). Cumulative incidence of death was 33.1% (95% CI: 28.9% to 37.8%) versus 25.4% (Figure 2 and 3a). Excess mortality in those undiagnosed with haemochromatosis at UK Biobank baseline (eTables 6 and 7) was similar (HR=1.22 95% CI: 1.05-1.43, $p=1.0*10^{-2}$) with a cumulative death rate of 32.5% (95% CI: 27.9 to 37.6%) (eFigure 1a).

Haemochromatosis diagnosis cumulative incidence at age 80 in p.C282Y+/+ males was 56.4% (Figure 2, eTable 8). Cumulative incidence of 'any liver disease' was 20.3% vs 8.3% without variants (HR 2.56, 95% CI: 2.10-3.12, $p=8.70*10^{-21}$) and 7.7% developed liver fibrosis or cirrhosis vs 1.3%. There was a raised HR for alcoholic liver disease (Figure 1) with cumulative incidence 3.3% of p.C282Y+/+ males vs 1.4%. Liver cancers cumulative incidence was 5.5% (95% CI: 3.8% to 8.0%, vs 0.8% without variants). p.C282Y+/+ males also had raised cumulative incidence of prostate cancer: 17.2% vs 14.8%. The cumulative incidence graphs show excess liver disease clearly apparent by age 55, but the excess mortality becoming significant at older ages (Figure 3a and 3b). Notably, 81.5% of deaths, 66.3% of 'any liver diseases', and 71.8% of joint replacements in p.C282Y+/+ males occurred after age 65.

For peer review only - http://bmjopen.bmj.com/site/about/guidelines.xhtml

Joint replacement cumulative incidence in p.C282Y+/+ males was 27.9% vs 17.1% without variants (Figure 3c): this remained after excluding those with fractures within 5 days before surgery (n=53; HR 1.74, 95% CI: 1.45-2.09; $p=1.6*10^{-9}$). p.C282Y+/+ males also had excess osteoarthritis, fragility fractures, and osteoporosis.

Brain outcome (dementia, delirium, or Parkinson's disease) cumulative incidence in p.C282Y+/+ males was 16.3% versus 10.0% without variants (HR 1.65 95% CI: 1.31-2.06, $p=1.70*10^{-5}$) (Figure 3d); for delirium 12.4% vs. 5.8% (HR 1.69, 95% CI: 1.26-2.27; $p=4.80*10^{-4}$); non-Alzheimer's dementia 6.0% vs 3.0% (HR 2.05, 95% CI: 1.40-3.00; $p=2.40*10^{-4}$), and Parkinson's disease (HR 1.86, 95% CI: 1.21-2.87 p=0.005).

Diabetes (type 1 or 2) cumulative incidence was 23.5% in p.C282Y+/+ males (vs. 19.1%). p.C282Y+/+ males had excess hospital diagnosed COVID-19 (8% vs 4.8%, HR 1.51, 95% CI: 1.08-2.11; p=0.02, missing significance in earlier follow-up data[27]), urinary tract infections (UTI) and skin and soft tissue infections (SSTI). Associations were not significant for cardiac outcomes (arrhythmia, cardiomyopathy, CHD, heart failure) or depression.

Estimates of excess morbidity in the male p.C282Y+/+ group without haemochromatosis diagnoses in the community at UK Biobank baseline (n=1,141) were mostly similar to those in the overall sample (Figures 2 and eFigure 1a-d, eTables 6 and 7). Notably, however, point HR estimates for liver fibrosis or cirrhosis, and liver cancers risk appeared marginally lower than in the whole group, but with wide confidence intervals including the whole study estimates HR point estimates (e.g. for liver fibrosis and cirrhosis HR 4.52, 95% CI: 3.07-6.66, $p=2.10*10^{-14}$ without diagnosis, versus HR 5.36 95% CI: 3.83-7.52; $p=2.00*10^{-22}$ in the whole p.C282Y+/+ group). In addition, alcoholic liver disease and osteoporosis lost statistical significance, but cholecystitis became nominally statistically significant (HR 1.54, 95% CI: 1.02-2.32, p=0.04).

Females with p.C282Y homozygote genotypes: excess morbidity versus without HFE variants

Diagnosed haemochromatosis cumulative incidence in p.C282Y+/+ females was 40.5% by age 80. There was no excess mortality, but this group experienced excess 'any liver disease' (8.9% vs 6.75%; HR 1.62, 95% CI: 1.27-2.05; $p=7.80*10^{-5}$) (Figure 4a), liver fibrosis or cirrhosis (1.9% vs 0.8%; HR 2.56, 95% CI: 1.50-4.36; p=0.001), alcoholic liver disease (1.0% vs 0.3%; HR 3.07, 95% CI: 1.44-6.54; p=0.004), plus cholecystitis, versus women without *HFE* variants (Figures 1 and 2; eTables 8, 9 and 10).

Joint replacement surgery was more common in female p.C282Y homozygotes (23.2% vs 21.1%) (Figure 4b), as were osteoarthritis and osteoporosis. Cumulative incidence of brain outcomes was raised (8.6% vs

For peer review only - http://bmjopen.bmj.com/site/about/guidelines.xhtml

7.4%; HR 1.30, 95% CI: 1.01-1.68; p=0.04), including delirium (5.9% vs 3.9%; HR 1.50. 95% CI: 1.08-2.08; p=0.02).

There was a nominally significant excess of heart failure (HR 1.34, 95% CI:1.03-1.75, p=0.03) but no associations with other studied cardiac outcomes. There were no associations with liver cancer, fragility fractures, diabetes, dementia, Parkinson's disease, or infections (SSTIs, UTIs).

Estimates of excess morbidity in the female p.C282Y+/+ group without haemochromatosis diagnoses in the community at UK Biobank baseline (n=1,550) were similar to those in the overall sample (Figures 2 and eFigure 2a-b, eTables 11 and 12).

Male and female p.C282Y/p.H63D compound heterozygotes versus without HFE variants

Haemochromatosis diagnosis cumulative incidence in p.C282Y/H63D males was 5.4% by age 80. However, there was no statistically significant increase in mortality, incidence of 'any liver disease' or liver cancers, joint replacements, or diabetes (Figure 1). p.C282Y/H63D males were modestly more likely to develop SSTIs (HR 1.16, 95% CI: 1.01-1.33, p=0.03) but this lost statistical significance after multiple testing correction.

Haemochromatosis cumulative incidence in p.C282Y/H63D females was 2.7% by age 80 but there were similarly no excess mortality, musculoskeletal diagnoses, diabetes, or brain outcomes (Figure 1). However, there was a modest association with 'any liver disease' (HR 1.19, 95% CI: 1.02-1.38, p=0.03), but associations with specific liver diagnoses were non-significant, including for liver fibrosis and cirrhosis, the most common form of liver disease with iron overload (Figures 1-2; eTables 4, 5, 8, 9, 10).

Males and females with p.H63D homozygote genotypes vs. no HFE variants

Diagnosed haemochromatosis cumulative incidence in p.H63D/H63D males was 1.9% by age 80, but with no excess mortality, liver disease diagnoses, liver cancers, joint replacement surgery, diabetes, or depression. There were protective associations between p.H63D homozygosity and fragility fractures (HR 0.78 95% CI: 0.62-0.98 p=0.03) and osteoporosis (HR 0.73 95% CI: 0.53-0.99, p=0.04) (eTables 4, 5 and 8).

Diagnosed haemochromatosis cumulative incidence p.H63D/H63D females was 0.6% by age 80. There was no excess mortality, liver disease or liver cancers, joint replacement surgery, diabetes, or depression. However, females also had a nominally significant (uncorrected $p<0.05$) protective association with fragility fractures (HR 0.87 95% CI: 0.76-1.00 p=0.05), echoing the similar protective associations in p.H63D/H63D males (eTables 8, 9 and 10).

For peer review only - http://bmjopen.bmj.com/site/about/guidelines.xhtml

Associations for p.C282Y+/- and p.H63D+/- (heterozygote carrier) males and females

There were a small number of nominally significant protective and risk associations in carrier groups (eTables 4 and 5), in p.C282Y+/- males including for excess delirium (HR 1.13 95% CI: 1.04-1.24 p=0.006) and skin and soft tissue infections (HR 1.14 95% CI: 1.06-1.22 p=1.80*10^{-4}) and for p.H63D+/- males a protective association with fragility fractures (HR 0.92 95% CI: 0.86-0.99 p=0.03). For p.C282Y+/- females there were no associations, but for p.H63D+/- females a nominally significant excess of liver cancers (HR 1.26 95% CI: 1.01-1.58 p=0.04) was present, plus a protective association for osteoporosis (eTables 9 and 10). Only the skin and soft tissue infection association was significant after multiple testing correction.

Discussion

Clinical penetrance by *HFE* genotypes in community samples has been uncertain, especially for those undiagnosed for haemochromatosis, the potential beneficiaries of population screening. Our results show greater excess morbidity and mortality in p.C282Y+/+ groups than previously documented, much occurring at older ages. p.C282Y+/+ males had marked excess mortality (33.1% dead by age 80 vs. 25.4% without p.C282Y or p.H63D variants), plus substantial excess joint replacements, liver disease, brain outcomes and some infections. Estimates of excess morbidity in p.C282Y+/+ groups undiagnosed for haemochromatosis were similar (eTables 7 and 12): perhaps most crucially, the p.C282Y homozygote male undiagnosed group had similar excess mortality, with an estimated cumulative death rate to age 80 of 32.5% (95% CI: 27.9 to 37.6%) versus 25.4% in those without *HFE* variants (eFigure 1a and eTable 7). Of p.C282Y+/+ females (n=1,604), diagnosed haemochromatosis cumulative incidence by 80 was 40.5%, with excess ‘any liver disease’, joint replacements and delirium present, suggesting more clinical penetrance than previously documented. In p.C282Y/p.H63D and H63D homozygote groups and carriers, we found no excess fatigue or depression at baseline and no excess mortality or major outcomes during follow-up.

Comparison to previous fatigue and depression studies

Baseline characteristics in UK Biobank have been reported before,[26], except for fatigue and depression. Fatigue and depression in haemochromatosis is important to patients and their carers, but the causal role of iron overload in these symptoms is unclear. p.C282Y+/+ males aged 60 plus reported excess fatigue (11.8% versus 8.2% without *HFE* variants) at baseline, but no excess depression at baseline or follow-up. Rates of both conditions were similar in the other studied genotype groups to those without *HFE* variants (except in p.H63D+/- males, with very modest excess fatigue, non-significant with multiple statistical testing correction). A blinded randomized trial of erythrocytapheresis[28] in p.C282Y+/+ groups with moderately raised ferritin levels (300 and 1000 μg/L) did find reduced fatigue scores with transferrin

For peer review only - http://bmjopen.bmj.com/site/about/guidelines.xhtml

saturations falling from mean 63.5% to 45.4% in the treatment group, although the mean fatigue score was below diagnosis level, making clinical interpretation unclear. Also, recent observational studies have cast doubt on relationships between fatigue, quality of life measures and iron parameters in haemochromatosis patients[29] [30], and fatigue might also occur with venesection. A UK biobank analysis found tiredness genetically linked to factors including increased adiposity, blood lipids and inflammatory markers[31].

Comparison to previous mortality-and morbidity studies

Rates of haemochromatosis diagnosis at baseline (mean age 57) were low even in p.C282Y+/+ groups (12.1% in males, 3.4% in females), but cumulative estimates to age 80 indicated much higher diagnosis rates at older ages. These baseline rates are similar to cumulative rates from the US hospitals genetics collaboration eMERGE study[32] for p.C282Y+/+ group at age 60. The eMERGE study[32] figures also show the majority of haemochromatosis diagnoses occurring later in life, reaching nearly 50% in males and 25% in females after age 80.

Earlier reports suggested no excess mortality in community-identified p.C282Y+/+[15] [18], despite higher death rates in clinical cohorts with liver disease[33], [15]. In the UK Biobank much larger sample, we found clearly increased all-cause mortality in p.C282Y+/+ males only (194 p.C282Y+/+ deaths, HR 1.29, 95% CI: 1.12 to 1.48, p=4.70*10^{-4}). A Swedish sample of 2,273 p.C282Y homozygotes found similar increased mortality (HR 1.30, 95% CI: 95%: 1.12-1.50, versus males with no mutations), with no excess in female homozygotes (HR 0.98, 95% CI: 0.82-1.18)[34]. These studies found no excess mortality in p.C282Y/p.H63D or p.H63D/p.H63D groups.

Estimates of disease penetrance in community genotyped groups differ widely [3][12] [13], with the highest estimate from The Melbourne Collaborative Cohort Study[15] , of 28.4% (95% CI: 18.8% to 40.2%) of p.C282Y+/+ males (n=95, mean age 65 at follow-up) having 'documented iron-overload-related disease', and 1.2% of female p.C282Y homozygotes affected. In UK Biobank we found that diagnosed haemochromatosis cumulative incidence was 36.1% in p.C282Y+/+ males and 21.2% of p.C282Y+/+ females by age 65 (eTable 8), suggesting a larger burden of iron overload disease especially in p.C282Y+/+ women. Liver cancers occurred in 5.5% (95% CI: 3.8% to 8.0%) of p.C282Y+/+ males by age 80, a slightly lower estimate than at age 75[7] (7.2% (95% CI: 3.9%-13.1%), but within the earlier confidence intervals. The estimate remains comparable to a meta-analysis[13] estimated lifetime incidence of severe liver disease (cirrhosis or hepatocellular carcinoma) of 9% (95% CI: 2.6%-15.3%) in untreated male *HFE* p.C282Y+/+ homozygotes.

For peer review only - http://bmjopen.bmj.com/site/about/guidelines.xhtml

Several studies have reported excess arthritis in p.C282Y homozygote men and to a lesser extent, in female homozygotes[9]. Our results extend a previous 11.5-year follow-up[35], now showing the full extent of later-life joint replacements in male p.C282Y homozygotes and providing a robust measure for severe joint damage in haemochromatosis.

Excess diabetes has previously been reported in p.C282Y homozygous men (9), but other studies have suggested that *HFE* mutations do not have important pathophysiological consequences in patients with type 2 diabetes[36]. We found a modest diabetes excess in p.C282Y+/+ individuals (Figures 1 and 2). Overall, as cumulative diabetes incidence was 23.5% in p.C282Y+/+ males vs. 19.1% without *HFE* variants, non-iron factors appear to now contribute the majority of diabetes even in p.C282Y+/+ males. Although cardiac complications are often noted haemochromatosis reviews[11][37], there is little evidence linking community-identified p.C282Y homozygosity to these outcomes[38] [39]: our finding of excess of heart failure in p.C282Y homozygote women only (HR 1.34 95% CI: 1.03-1.75, p=0.03) needs further study.

Our previous UK Biobank report on brain outcomes (mean 10.5 years follow-up) found p.C282Y+/+ males had excess dementia diagnoses and iron deposition in key brain areas on MRI[40]. The current analysis also found higher incidence especially of non-Alzheimer's dementia in p.C282Y+/+ males plus more delirium. Loughnan et al[41] reported that p.C282Y+/+ males had more Parkinson's disease (OR 1.83; 95% CI: 1.19-2.80; p=0.006) in cross-sectional analyses of UK Biobank: our incident analyses provide a similar estimate for Parkinson's disease in p.C282Y+/+ males (HR 1.86, 95% CI: 1.21-2.87 p=0.005).

In the HEIRS study[3] community genotyped p.C282Y/p.H63D (compound heterozygote) males and females had substantially lower transferrin saturation levels compared to p.C282Y+/+ males or females. In line with this, cumulative incidence of haemochromatosis diagnosed by age 80 was relatively low (men 5.4%, women 2.7%), and no excess mortality, liver fibrosis and cirrhosis, joint replacements, diabetes, or depression in these groups. We did find that p.C282Y/p.H63D females had nominally significant small excess of any liver disease (HR 1.19, 95% CI: 1.02-1.38; p=0.03) although with no excess liver fibrosis and cirrhosis, no similar association in p.C282Y/p.H63D males, and with a non-significant multiple testing p-value. p.C282Y/p.H63D males had a small excess of skin and soft tissue infections (HR 1.16, 95% CI: 1.01-1.33, uncorrected p=0.03), again multiple testing non-significant.

Strengths and limitations

We analyzed largescale cohort data on community volunteers, providing the first outcome data to age 80. *HFE* allele frequencies were similar to other UK studies[7] but UK Biobank did recruit somewhat healthier

For peer review only - http://bmjopen.bmj.com/site/about/guidelines.xhtml

participants than the general population[23], so we have focused on incident outcomes during the mean 13.3 year follow-up: we found no deviations from proportional hazards assumptions in Cox models. Outcomes for both the overall genotype groups and those undiagnosed with haemochromatosis at UK Biobank baseline are presented. Previous studies have suggested that treated p.C282Y homozygous patients do not have an increased mortality rate compared to the general population [42], but numbers with haemochromatosis diagnoses at baseline were small in the current study and so outcomes in this group were not presented. Also, there was no data available on the diverse routes to diagnosis, so modelling treated outcomes would likely be confounded. Further work is needed on the effect so early diagnosis and treatment. Most outcomes were from hospital inpatient care, so community diagnosed conditions may be underestimated, but findings are similar to analyses including primary care records (available to 2017 only)[7] and baseline data collected at interview[9]. Information on the criteria used to establish a diagnosis of fibrosis/cirrhosis in the hospital follow-up data (e.g. non-invasive biomarker panels or liver biopsy) were not available.

Implications for early diagnosis and screening

We found greater excess morbidity than previously reported in both p.C282Y+/+ males and females, especially at older ages, and in those undiagnosed with haemochromatosis. Despite treatment (predominantly venesection) being considered effective for preventing liver disease progression[1][43][44] rates of haemochromatosis diagnosis were low at baseline: for example, of 31 p.C282Y+/+ males with incident liver cancer, 17 were undiagnosed with haemochromatosis at baseline. Genotyping earlier in life could be a powerful preventive tool to identify p.C282Y+/+ individuals who are clearly at major risk of related excess disease. A recent cross-sectional genotyping study within a US health provider found 72% (144/201) of p.C282Y homozygous patients (mean age 62) had not been diagnosed with haemochromatosis, 36% of whom had iron overload [21]. As cumulative diagnosed haemochromatosis in UK Biobank cohort was estimated 56.4% in p.C282Y+/+ males and 40.5% females by age 80, p.C282Y+/+ genotyping with follow-up iron studies would likely have a high yield. However, the prevention potential following up p.C282Y/p.H63D, H63D+/+ groups will likely be very low, as there was no significant excess fatigue or depression at baseline, and no excess mortality, incident liver fibrosis or cirrhosis, joint replacements, or depression.

Conclusion

Male and female p.C282Y homozygotes in this community cohort experienced greater excess morbidity than previously documented. Much of this excess liver, musculoskeletal, diabetes, brain and morbidity

For peer review only - http://bmjopen.bmj.com/site/about/guidelines.xhtml

occurred after age 60. In those not diagnosed with haemochromatosis at study baseline, risks were broadly similar to those in the overall group, notably including excess mortality in p.C282Y+/+ males. Trials of targeted or community genotyping to diagnose haemochromatosis earlier appear justified, especially to identify people with the p.C282Y homozygote variants. The potential for iron related preventive treatment in the p.C282Y/H63D, p.H63D+/+ and other *HFE* genotype groups appears very limited, as these genotype groups were associated with no statistically significant excess morbidity or mortality.

Data sharing

Data are available on application to the UK Biobank (www.ukbiobank.ac.uk/register-apply).

Funding

University of Exeter supports ML, LP and DM; JA has a National Institute for Health and Care Research (NIHR) Advanced Fellowship (NIHR301844).

Authors contributions

ML performed the analysis, interpreted results, created the figures, and drafted the manuscript. JA contributed to the design and analysis of the study, data interpretation and drafting of the manuscript. LP contributed to the design of the study, data interpretation, creation of figures and contributed to the manuscript. JS provided expert clinical interpretation of the data and contributed to the manuscript. DM oversaw design of the study, data analysis, interpretation of results, and led the writing of the manuscript. All authors approved the final version of the manuscript.

Acknowledgements

This research was conducted using the UK Biobank resource, under application 14631. We thank the UK Biobank participants and coordinators. This work used data provided by patients and collected by the NHS as part of their care and support. Copyright © (2023), NHS England. Re-used with the permission of the NHS England and UK Biobank. All rights reserved. This research also used data assets made available by National Safe Haven as part of the Data and Connectivity National Core Study, led by Health Data Research UK in partnership with the Office for National Statistics and funded by UK Research and Innovation. This study was supported by the National Institute for Health and Care Research Exeter Biomedical Research Centre. The views expressed are those of the authors and not necessarily those of the NIHR or the Department of Health and Social Care.

For peer review only - http://bmjopen.bmj.com/site/about/guidelines.xhtml

Competing interests

None declared.

For peer review only

For peer review only - http://bmjopen.bmj.com/site/about/guidelines.xhtml

Table 1. Baseline characteristics of male and female UK Biobank participants by selected p.C282Y/H63D genotypes

	**No** p.C282Y or p.H63D **variants**	**H63D +/+**	**C282Y +/ H36D +**	**C282Y +/+**
**MALES**				
Total participants	122,841	4,673	4,959	1,298
Mean age, years (SD)	56.99 (8.1)	56.99 (8.1)	56.97 (8.1)	56.84 (8.2)
Hemochromatosis diagnosis, n (%)	29 (0.02)	8 (0.2)	29 (0.6)	157 (12.1)
Self-reported fatigue, n (%)	12,094 (10.1)	451 (9.9)	523 (10.9)	148 (11.8)
Self-reported fatigue (60+ years), n (%),	4,475 (8.2)	173 (8.2)	177 (8.0)	68 (11.8)
Prevalent depression, n (%)	5,883 (4.8)	220 (4.7)	188 (3.8)	71 (5.5)
**FEMALES**				
Total participants	145,694	5,580	5,760	1,604
Mean age, years (SD)	56.62 (7.9)	56.58 (8.1)	56.46 (7.9)	56.92 (8.0)
Hemochromatosis diagnosis, n (%)	8 (0.01)	2 (0.04)	12 (0.2)	54 (3.4)
Self-reported fatigue, n (%)	19,110 (13.5)	760 (14.1)	785 (14.1)	220 (14.4)
Self-reported fatigue (60+ years), n (%),	6,055 (10.0)	253 (10.9)	229 (9.9)	77 (11.4)
Prevalent depression, n (%)	10,734 (7.4)	387 (6.9)	432 (7.5)	123 (7.7)

A total of 451,270 male and female participants genetically-similar to the 1000 Genomes project European reference population with *HFE* genotypic data available in the UK Biobank. Numbers presented are mean (SD) for continuous variables and n (%) for categorical variables. Fatigue = tiredness in more than half the days in past 2 weeks. See supplementary eTable 2 and 3 for all p.C282Y/H63D genotype groups.

For peer review only - http://bmjopen.bmj.com/site/about/guidelines.xhtml

For peer review only

Figure 1. Hazard ratios of incident disease outcomes (95% CI) in selected p.C282Y/H63D genotypes compared to those with no mutations. Hazard ratios compared to those with neither *HFE* mutation. Cox proportional hazards regression models adjusted for age, assessment centre, and genetic principal components 1–10. Abbreviations: UTI, urinary tract infection; SSTI, skin and soft tissue infection; CI, confidence interval. Joint replacement surgery includes a diagnosis of hip, knee, ankle, or shoulder replacement. Any brain outcome included a diagnosis of delirium, dementia, or Parkinson's disease. See supplementary eTables 4, 5, 9 and 10 for incident numbers and HRs for all p.C282Y/H63D genotype groups.

Figure 2. Cumulative incidence of outcomes from ages 40-80 years by *HFE* genotypes (no p.C282Y or p.H63D variants vs p.C282Y homozygotes and p.C282Y homozygotes undiagnosed with hemochromatosis at baseline). Cumulative incidence (estimated % diagnosed by age 80 years; 95% CIs) for significant outcomes ($p<0.05$) from Cox proportional hazards regression models (Figure 1; eTables 5, 7, 10 and 12). Abbreviations: UTI, urinary tract infection; SSTI, skin and soft tissue infection; CI, confidence interval. Joint replacement surgery includes a diagnosis of hip, knee, ankle, or shoulder replacement. Any brain outcome included a diagnosis of delirium, dementia, or Parkinson's disease.

Figure 3. Kaplan-Meier curves for the cumulative incidence of (a) mortality, (b) liver disease, (c) any joint replacement, and (d) any brain outcome, in male *HFE* p.C282Y homozygotes compared to those with no mutations. Cumulative incidence (estimated % diagnosed by age 80 years; 95% CIs). Joint replacement surgery includes a diagnosis of hip, knee, ankle, or shoulder replacement. Any brain outcome included a diagnosis of delirium, dementia, or Parkinson's disease.

Figure 4. Kaplan-Meier curves for the cumulative incidence of (a) liver disease, and (b) any joint replacement, in female *HFE* p.C282Y homozygotes compared to those with no mutations. Cumulative incidence (estimated % diagnosed by age 80 years; 95% CIs). Joint replacement surgery includes a diagnosis of hip, knee, ankle, or shoulder replacement.

For peer review only - http://bmjopen.bmj.com/site/about/guidelines.xhtml

References

1. Adams PC, Jeffrey G, Ryan J. Haemochromatosis. Lancet. 2023;1811–21.

2. Katsarou MS, Papasavva M, Latsi R, Drakoulis N. Hemochromatosis: Hereditary hemochromatosis and HFE gene. Vitam Horm. 2019 Jan 1;110:201–22.

3. Adams PC, Reboussin DM, Barton JC, McLaren CE, Eckfeldt JH, McLaren GD, et al. Hemochromatosis and iron-overload screening in a racially diverse population. N Engl J Med. 2005 Apr 28;352(17):1769–78.

4. Bomford A. Genetics of haemochromatosis. Lancet. 2002 Nov 23;360(9346):1673–81.

5. Adams P. Classic Hereditary Hemochromatosis [Internet]. National Organization for Rare Disorders. 2019. p. 1–3. Available from: https://rarediseases.org/rare-diseases/classic-hereditary-hemochromatosis/

6. Adams PC. Epidemiology and diagnostic testing for hemochromatosis and iron overload. Int J Lab Hematol. 2015;37(S1):25–30.

7. Atkins JL, Pilling LC, Masoli JAH, Kuo CL, Shearman JD, Adams PC, et al. Association of Hemochromatosis HFE p.C282Y Homozygosity with Hepatic Malignancy. JAMA - Journal of the American Medical Association. 2020 Nov 24;324(20):2048–57.

8. Wijarnpreecha K, Aby ES, Panjawatanan P, Kroner PT, Harnois DM, Palmer WC, et al. Hereditary hemochromatosis and risk of joint replacement surgery: A systematic review and meta-analysis. Eur J Gastroenterol Hepatol. 2020;33(1):96–101.

9. Pilling LC, Tamosauskaite J, Jones G, Wood AR, Jones L, Kuo C ling L, et al. Common conditions associated with hereditary haemochromatosis genetic variants: cohort study in UK Biobank. BMJ. 2019 Jan 16;364:k5222.

10. Pelusi C, Gasparini DI, Bianchi N, Pasquali R. Endocrine dysfunction in hereditary hemochromatosis. Vol. 39, Journal of Endocrinological Investigation. Springer International Publishing; 2016. p. 837–47.

11. Joshi PK, Patel SC, Shreya D, Zamora DI, Patel GS, Rodriguez K, et al. Hereditary Hemochromatosis : A Cardiac Perspective. 2021;13(11).

12. Beutler E, Felitti VJ, Koziol JA, Ho NJ, Gelbart T. Penetrance of 845G → A (C282Y) HFE hereditary haemochromatosis mutation in the USA. Lancet. 2002;359(9302):211–8.

13. Grosse SD, Gurrin LC, Bertalli NA, Allen KJ. Clinical penetrance in hereditary hemochromatosis: estimates of the cumulative incidence of severe liver disease among HFE C282Y homozygotes. Genetics in Medicine. 2017;

14. McLaren CE, Barton JC, Adams PC, Harris EL, Acton RT, Press N, et al. Hemochromatosis and Iron Overload Screening (HEIRS) study design for an evaluation of 100,000 primary care-based adults. Am J Med Sci. 2003 Feb;325(2):53–62.

15. Allen KJ, Gurrin LC, Constantine CC, Osborne NJ, Delatycki MB, Nicoll AJ, et al. Iron-Overload–Related Disease in *HFE* Hereditary Hemochromatosis. New England Journal of Medicine. 2008 Jan 17;358(3):221–30.

For peer review only - http://bmjopen.bmj.com/site/about/guidelines.xhtml

16. Niederau C, Fischer R, Purschel A, Purschel P, Stremmel W, Ha¨ussinger D, et al. Long-term Survival in Patients With Hereditary Hemochromatosis. Vol. 110, Gastroenterology. W.B. Saunders; 1996 Apr.

17. Adams PC, Richard L, Weir M, Speechley M. Survival and development of health conditions after iron depletion therapy in C282Y-linked hemochromatosis patients. Canadian Liver Journal. 2021;4(4):381–90.

18. Van Aken MO, De Craen AJM, Gussekloo J, Moghaddam PH, Vandenbroucke JP, Heijmans BT, et al. No increase in mortality and morbidity among carriers of the C282Y mutation of the hereditary haemochromatosis gene in the oldest old: The Leiden 85-plus Study. Eur J Clin Invest. 2002;32(10):750–4.

19. Gochee PA, Powell LW, Cullen DJ, Du Sart D, Rossi E, Olynyk JK. A population-based study of the biochemical and clinical expression of the H63D hemochromatosis mutation. Gastroenterology. 2002 Mar;122(3):646–51.

20. Schmidtke J. Twenty-Five Years of Contemplating Genotype-Based Hereditary Hemochromatosis Population Screening. Genes (Basel). 2022;13(9):1622.

21. Savatt JM, Johns A, Schwartz MLB, McDonald WS, Salvati ZM, Oritz NM, et al. Testing and Management of Iron Overload After Genetic Screening–Identified Hemochromatosis. JAMA Netw Open. 2023 Oct 23;6(10):e2338995.

22. Miller DT, Lee K, Chung WK, Gordon AS, Herman GE, Klein TE, et al. ACMG SF v3.0 list for reporting of secondary findings in clinical exome and genome sequencing: a policy statement of the American College of Medical Genetics and Genomics (ACMG). Genetics in Medicine. 2021;23(8):1381–90.

23. Fry A, Littlejohns TJ, Sudlow C, Doherty N, Adamska L, Sprosen T, et al. Comparison of Sociodemographic and Health-Related Characteristics of UK Biobank Participants With Those of the General Population. Am J Epidemiol. 2017 Nov 1;186(9):1026–34.

24. National Academies of Sciences, Engineering, and Medicine, [Internet]. Committee on the Use of Race, Ethnicity, and Ancestry as Population Descriptors in Genomics Research, Board on Health Sciences Policy, Committee on Population, Health and Medicine Division, Division of Behavioral and Social Sciences and Education, Nationa. National Academies Press, Washington, D.C; 2023. Available from: https://www.nap.edu/catalog/26902

25. Van Hout C V., Tachmazidou I, Backman JD, Hoffman JD, Liu D, Pandey AK, et al. Exome sequencing and characterization of 49,960 individuals in the UK Biobank. Nature. 2020;586(7831):749–56.

26. Tamosauskaite J, Atkins JL, Pilling LC, Kuo CL, Kuchel GA, Ferrucci L, et al. Hereditary Hemochromatosis Associations with Frailty, Sarcopenia and Chronic Pain: Evidence from 200,975 Older UK Biobank Participants. J Gerontol A Biol Sci Med Sci. 2019 Jan 15;74(3):337–42.

27. Atkins JL, Lucas M, Pilling LC, Melzer D. Letter to the editor regarding: “A haemochromatosis-causing HFE mutation is associated with SARS-CoV-2 susceptibility in the Czech population”. Clinica Chimica Acta 538 (2023) 211–215. Clinica Chimica Acta. 2023;542(February):117271.

For peer review only - http://bmjopen.bmj.com/site/about/guidelines.xhtml

28. Ong SY, Gurrin LC, Dolling L, Dixon J, Nicoll AJ, Wolthuizen M, et al. Reduction of body iron in HFE-related haemochromatosis and moderate iron overload (Mi-Iron): a multicentre, participant-blinded, randomised controlled trial. Lancet Haematol. 2017 Dec 1;4(12):e607–14.

29. Acevedo LAU, Alvarenga AM, Fonseca PFS, da Silva NK, Cançado RD, Naoum FA, et al. Quality of Life Scores Remained Different among the Genotypic Groups of Patients with Suspected Hemochromatosis, Even after Treatment Period. Genes (Basel). 2022 Jan 1;13(1).

30. Belhomme N, Morcet J, Lescoat A, Robin F, Deugnier Y, Bardou-Jacquet E, et al. Patient-reported outcomes and their relation with iron parameters in HFE haemochromatosis during maintenance therapy: A prospective cohort study. Liver Int. 2022 Jun 30;

31. Deary V, Hagenaars SP, Harris SE, Hill WD, Davies G, Liewald DCM, et al. Genetic contributions to self-reported tiredness. Mol Psychiatry. 2018 Mar;23(3):609–20.

32. Gallego CJ, Burt A, Sundaresan AS, Ye Z, Shaw C, Crosslin DR, et al. Penetrance of Hemochromatosis in HFE Genotypes Resulting in p.Cys282Tyr and p.[Cys282Tyr];[His63Asp] in the eMERGE Network. Am J Hum Genet. 2015 Oct 1;97(4):512–20.

33. Adams PC, Speechley M, Kertesz AE. Long-term survival analysis in hereditary hemochromatosis. Gastroenterology. 1991;101(2):368–72.

34. Hagström H, Ndegwa N, Jalmeus M, Ekstedt M, Posserud I, Rorsman F, et al. Morbidity, risk of cancer and mortality in 3645 HFE mutations carriers. Liver Int. 2021 Mar 1;41(3):545–53.

35. Banfield LR, Knapp KM, Pilling LC, Melzer D, Atkins JL. Hemochromatosis Genetic Variants and Musculoskeletal Outcomes: 11.5-Year Follow-Up in the UK Biobank Cohort Study. JBMR Plus. 2023;00(00):1–11.

36. Davis TME, Beilby J, Davis WA, Olynyk JK, Jeffrey GP, Rossi E, et al. Prevalence, Characteristics, and Prognostic Significance of *HFE* Gene Mutations in Type 2 Diabetes. Diabetes Care. 2008 Sep 1;31(9):1795–801.

37. Kowdley K V., Brown KE, Ahn J, Sundaram V. ACG Clinical Guideline. Am J Gastroenterol. 2019;114(August):1.

38. Fox CJ, Cullen DJ, Knuiman MW, Cumpston GN, Divitini ML, Rossi E, et al. Effects of body iron stores and haemochromatosis genotypes on coronary heart disease outcomes in the Busselton health study. J Cardiovasc Risk. 2002 Oct;9(5):287–93.

39. Waalen J, Felitti V, Gelbart T, Ho NJ, Beutler E. Prevalence of coronary heart disease associated with HFE mutations in adults attending a health appraisal center. Am J Med. 2002 Oct;113(6):472–9.

40. Atkins JL, Pilling LC, Heales CJ, Savage S, Kuo CL, Kuchel GA, et al. Hemochromatosis mutations, brain iron imaging, and dementia in the UK Biobank cohort. Journal of Alzheimer's Disease. 2021;79(3):1203–11.

41. Loughnan R, Ahern J, Tompkins C, Palmer CE, Iversen J, Thompson WK, et al. Association of Genetic Variant Linked to Hemochromatosis With Brain Magnetic Resonance Imaging Measures of Iron and Movement Disorders. 2022;74136:1–10.

For peer review only

For peer review only - http://bmjopen.bmj.com/site/about/guidelines.xhtml

42. Bardou-Jacquet E, Morcet J, Manet G, Lainé F, Perrin M, Jouanolle AM, et al. Decreased cardiovascular and extrahepatic cancer-related mortality in treated patients with mild HFE hemochromatosis. J Hepatol. 2015 Mar;62(3):682–9.

43. Bardou-Jacquet E, Morandeau E, Anderson GJ, Ramm GA, Ramm LE, Morcet J, et al. Regression of Fibrosis Stage With Treatment Reduces Long-Term Risk of Liver Cancer in Patients With Hemochromatosis Caused by Mutation in HFE. Clin Gastroenterol Hepatol. 2020 Jul;18(8):1851–7.

44. Powell LW, Dixon JL, Ramm GA, Purdie DM, Lincoln DJ, Anderson GJ, et al. Screening for hemochromatosis in asymptomatic subjects with or without a family history. Arch Intern Med. 2006 Feb 13;166(3):294–301.

For peer review only

For peer review only

For peer review only - http://bmjopen.bmj.com/site/about/guidelines.xhtml

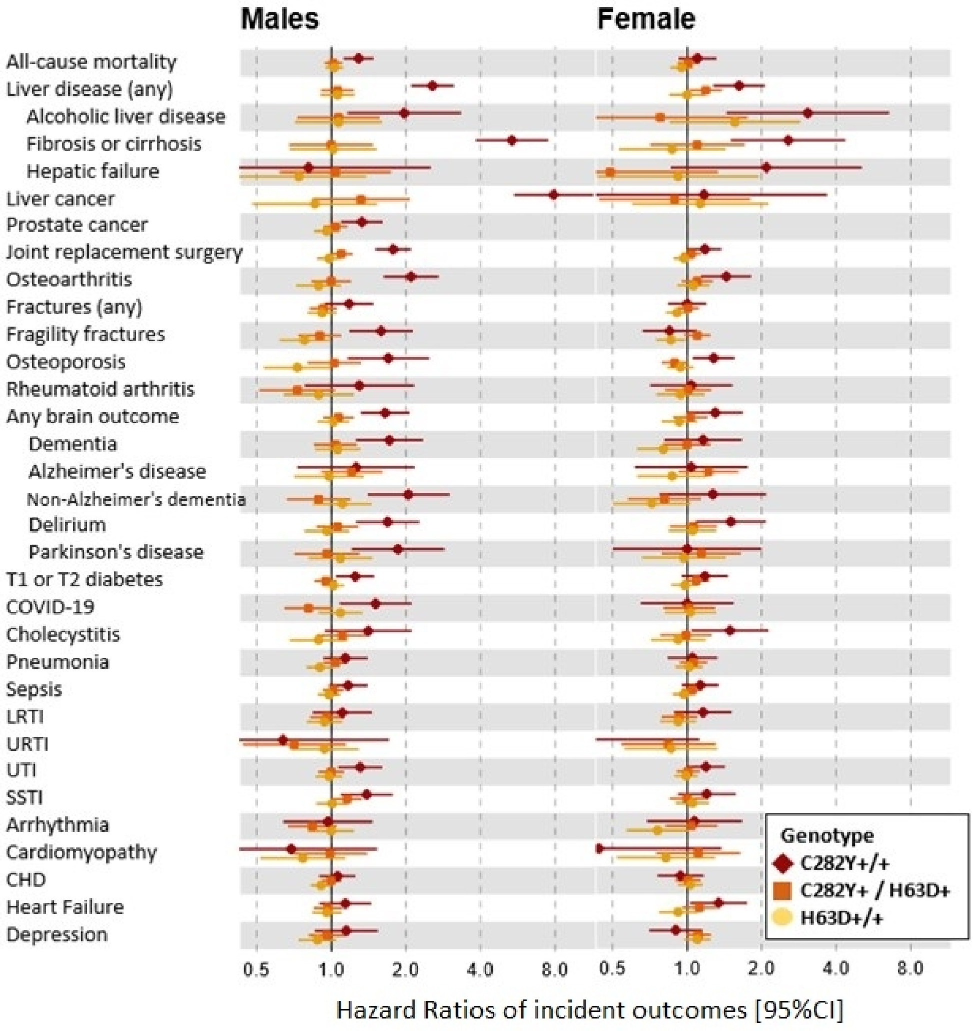

Figure 1

53x56mm (300 x 300 DPI)

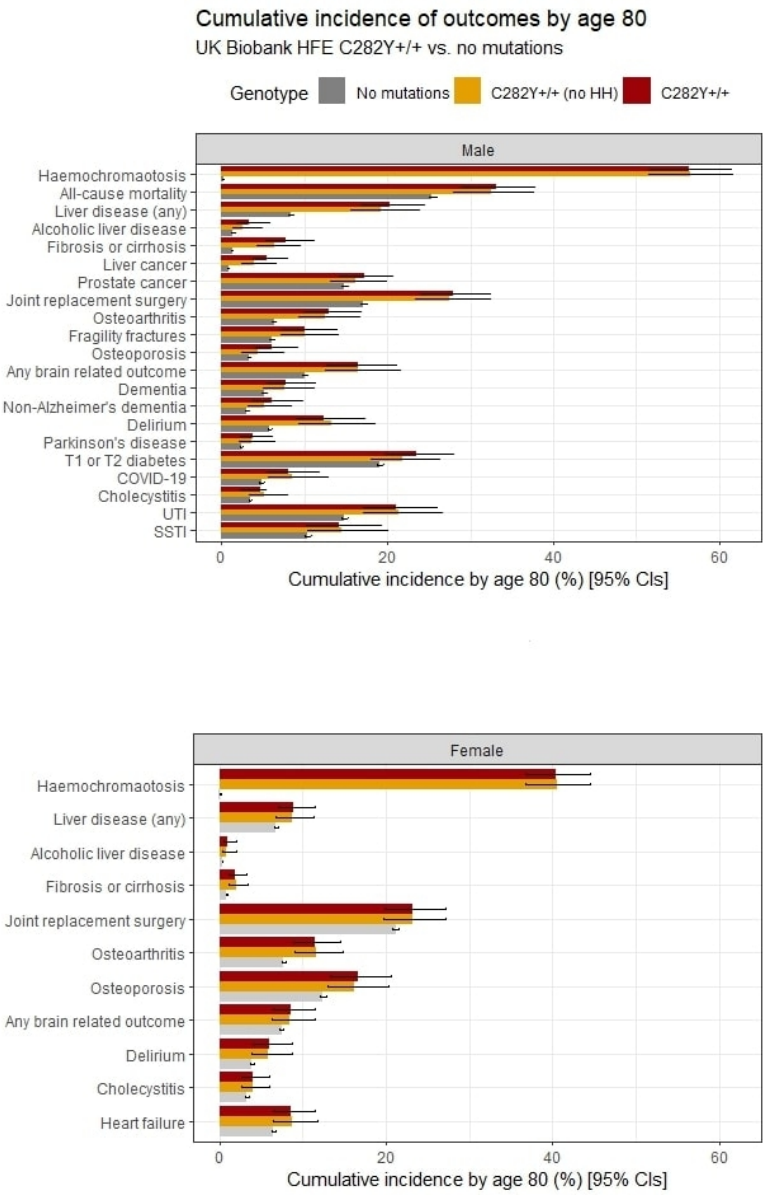

Figure 2

52x79mm (300 x 300 DPI)

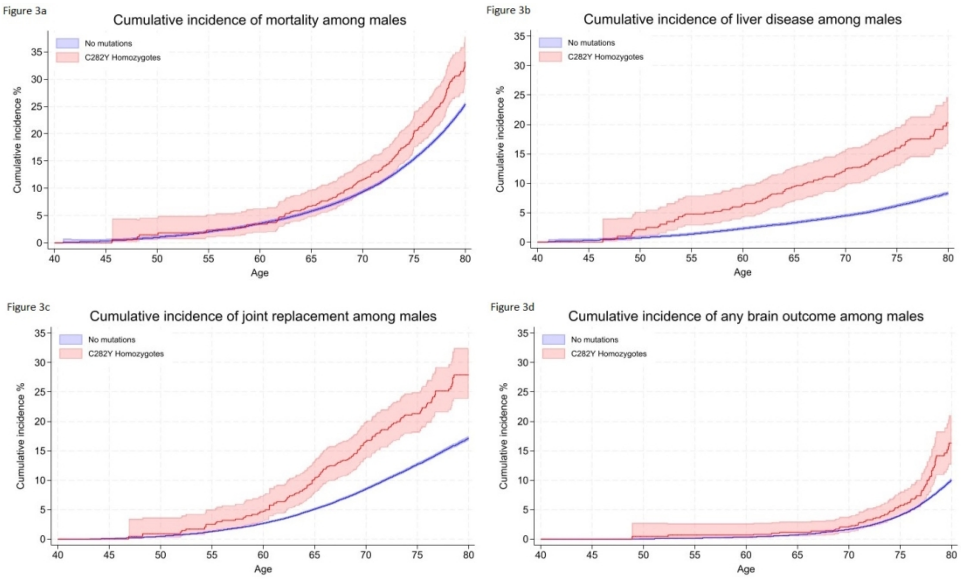

127x76mm (300 x 300 DPI)

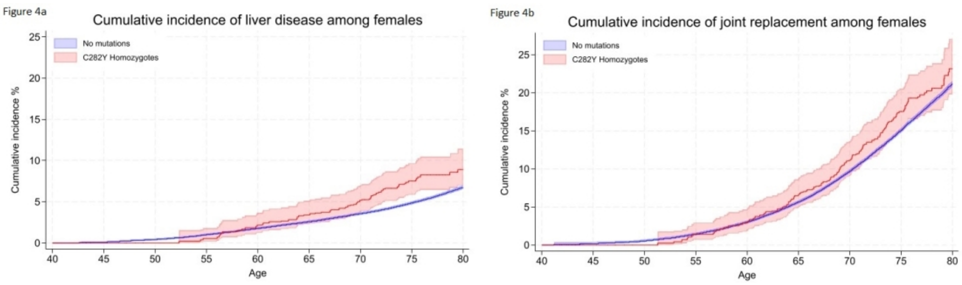

127x36mm (300 x 300 DPI)

Supplementary Material

eTable 1. Incident hospital diagnosed outcomes or procedures with associated ICD-10/OPCS codes

Disease	ICD-10 code
Arrhythmia	I49
Cardiomyopathy	I42
CHD	I20; I21; I22; I23; I24; I25
Cholecystitis	K800; K804; K81
COVID-19	U07.1; U07.2
Delirium	F05
Dementia	F00; F01; F02; F03; G30
Alzheimer's disease	G30
Non-Alzheimer's dementia	F00; F01; F02; F03
Depression	F32; F33; F34.1
Fractures (any)	S02; S12; S22; S32; S42; S52; S62; S72; S82; S92; T02; T08; T10; T12; T14.2
Fragility fractures	S220; S32; S325; S328; S422; S423; S424; S524; S525; S720; S721; S722; S582; S5823; T08
Hemochromatosis	E83.1
Heart failure	I50; J81
Liver disease (any)	K70; K71; K72; K73; K74; K75; K76; K77
Alcoholic liver disease	K70
Fibrosis & Cirrhosis	K74
Hepatic failure	K72
Liver cancer	C22
Lower respiratory tract infection	J20; J21; J22
Osteoarthritis	M15.0; M15.1; M15.2; M15.9; M16.0; M16.1; M17.0; M17.1; M18.0; M18.1; M19.0
Osteoporosis	M80; M81; M811; M812; M813; M814; M815; M816; M818; M819
Parkinson's disease	G20; F02.3
Pneumonia	J13; J14; J15; J16; J17; J18
Prostate cancer	C61
Rheumatoid arthritis	M05; M06
Sepsis	A021; A039; A207; A241; A217; A227; A239; A267; A282; A327; A392; A393; A394; A40; A41; A427; A548; B007; B377; H440; J950; N390; O85; P36; R651; T814; T880
Skin soft tissue infection	L00; L01; L02; L03; L04; L05; L06; L07; L08
Type 1 or Type 2 diabetes	E10; E11
Urinary tract infection	N30; N34; N39
Upper respiratory tract infection	J39; J06; J04

Procedure	OPCS Code
Ankle replacement	O32; O320; O321; O322; O323; O324; O325
Hip replacement	W37;W370;W371;W372;W373;W374;W38;W380;W381;W382;W383;W384;W46;W460;W461;W462;W463;W47;W470;W471;W472;W473;W93;W930;W931;W932;W933;W94;W940;W941;W942;W943;O171;O172;O173;W580;W581;W582
Knee replacement	O18*; W40*; W41*; W42*
Shoulder replacement	O06; +A11O060; O061; O062; O063; O068; O069; O07; O070; O071; O072; O073; O078; O079; O08; O080; O081; O082; O083; O084; O088; O089; O09; O091; O098; O099; O10; O101; O108; O109

For peer review only - http://bmjopen.bmj.com/site/about/guidelines.xhtml

ICD-10 = International Classification of Diseases 10th revision codes; OPCS-4 = OPCS Classification of Interventions and Procedures version 4. Joint replacement surgery variable includes a diagnosis of hip, knee, ankle, or shoulder replacement. Any brain outcome variable includes a diagnosis of dementia, delirium, or Parkinson's disease.

For peer review only

For peer review only - http://bmjopen.bmj.com/site/about/guidelines.xhtml

eTable 2. Baseline characteristics of male UK Biobank participants by p.C282Y/H63D genotypes

	No mutations	H63D+/-	H63D+/+	C282Y+/H63+	C282Y+/-	C282Y +/+	Total
**Total participants**	122,841	47,983	4,673	4,959	24,636	1,298	206,390
Mean age, years (SD)	56.99 (8.1)	57.02 (8.1)	56.99 (8.1)	56.97 (8.1)	57.02 (8.1)	56.84 (8.2)	57.00 (8.1)
Hemochromatosis diagnosis, n (%)	29 (0.02)	17 (0.04)	8 (0.2)	29 (0.6)	27 (0.1)	157 (12.1)	267 (0.1)
Self-reported fatigue, n (%)	12,094 (10.1)	4,865 (10.4)	451 (9.9)	523 (10.9)	2,476 (10.3)	148 (11.8)	20,557 (10.2)
Self-reported fatigue (60+ years), n (%),	4,475 (8.2)	1,858 (8.6)	173 (8.2)	177 (8.0)	924 (8.4)	68 (11.8)	7,675 (8.3)
Depression diagnosis, n (%)	5,883 (4.8)	2,232 (4.7)	220 (4.7)	188 (3.8)	1,146 (4.7)	71 (5.5)	9,740 (4.7)

A total of 206,390 male participants genetically similar to the 1000 Genomes project European reference population with *HFE* genotypic data available in the UK Biobank. Numbers presented are mean (SD) for continuous variables and n (%) for categorical variables. Fatigue = tiredness in more than half the days in past 2 weeks.

For peer review only - http://bmjopen.bmj.com/site/about/guidelines.xhtml

eTable 3. Baseline characteristics of female UK Biobank participants by p.C282Y/H63D genotypes

	No mutations	H63D+/-	H63D+/+	C282Y+/H63+	C282Y+/-	C282Y +/+	Total
**Total participants**	145,694	57,021	5,580	5,760	29,221	1,604	244,880
Mean age, years (SD)	56.62 (7.9)	56.58 (7.9)	56.58 (8.1)	56.46 (7.9)	56.49 (8.0)	56.92 (8.0)	56.60 (7.9)
Hemochromatosis diagnosis, n (%)	8 (0.01)	6 (0.01)	2 (0.04)	12 (0.2)	5 (0.02)	54 (3.4)	87 (0.04)
Self-reported fatigue, n (%)	19,110 (13.5)	7,449 (13.5)	760 (14.1)	785 (14.1)	3,802 (13.4)	220 (14.4)	32,126 (13.5)
Self-reported fatigue (60+ years), n (%),	6,055 (10.0)	2,417 (10.2)	253 (10.9)	229 (9.9)	1,157 (9.6)	77 (11.4)	10,188 (10.0)
Depression diagnosis, n (%)	10,734 (7.4)	4,266 (7.5)	387 (6.9)	432 (7.5)	2,271 (7.8)	123 (7.7)	18,213 (7.4)

A total of 244,880 female participants genetically similar to the 1000 Genomes project European reference population with *HFE* genotypic data available in the UK Biobank. Numbers presented are mean (SD) for continuous variables and n (%) for categorical variables. Fatigue = tiredness in more than half the days in past 2 weeks.

For peer review only - http://bmjopen.bmj.com/site/about/guidelines.xhtml

eTable 4. Incident hospital diagnoses in male UK Biobank participants by p.C282Y/H63D genotypes

Males	No mutations	H63D +/-	H63D +/+	C282Y +/ H36D +	C282Y +/-	C282Y +/+
Hemochromatosis	85 (0.1)	51 (0.1)	29 (0.6)	95 (1.9)	42 (0.2)	288 (25.2)
All-cause mortality	13,976 (11.4)	5,658 (11.8)	540 (11.6)	591 (11.9)	2,996 (12.2)	194 (15.0)
**Liver**						
Liver disease (any)	3,969 (3.3)	1,621 (3.4)	160 (3.5)	171 (3.5)	803 (3.3)	102 (8.1)
Alcoholic liver disease	608 (0.5)	227 (0.5)	25 (0.5)	28 (0.6)	150 (0.6)	14 (1.1)
Fibrosis & Cirrhosis	618 (0.5)	252 (0.5)	24 (0.5)	26 (0.5)	142 (0.6)	36 (2.8)
Hepatic failure	355 (0.3)	137 (0.3)	10 (0.2)	15 (0.3)	54 (0.2)	3 (0.2)
**Cancer**						
Liver cancer	363 (0.3)	167 (0.4)	12 (0.3)	20 (0.4)	80 (0.3)	31 (2.4)
Prostate cancer	7,723 (6.4)	3,025 (6.4)	283 (6.2)	319 (6.5)	1,613 (6.7)	104 (8.1)
**Musculoskeletal**						
Joint replacement surgery (any)	8,429 (7.1)	3,379 (7.3)	315 (7.0)	376 (7.8)	1,801 (7.5)	144 (11.8)
Osteoarthritis	2,873 (2.5)	1,155 (2.6)	97 (2.2)	120 (2.6)	601 (2.6)	61 (5.5)
Fractures (any)	6,092 (5.2)	2,298 (5.1)	212 (4.8)	231 (4.9)	1,243 (5.3)	75 (6.2)
Fragility fractures	2,666 (2.2)	963 (2.0)	79 (1.7)	98 (2.0)	551 (2.3)	44 (3.4)
Osteoporosis	1,522 (1.3)	602 (1.3)	42 (0.9)	64 (1.3)	319 (1.3)	27 (2.1)
Rheumatoid arthritis	1,131 (0.9)	479 (1.0)	38 (0.8)	33 (0.7)	238 (1.0)	15 (1.2)
**Brain**						
Any brain outcome (dementia, delirium, or Parkinson's disease)	4,619 (3.8)	1,852 (3.9)	177 (3.8)	201 (4.1)	993 (4.0)	76 (5.9)
Dementia	2,306 (1.9)	916 (1.9)	92 (2.0)	97 (2.0)	487 (2.0)	40 (3.1)
Alzheimer's disease	1,026 (0.8)	380 (0.8)	38 (0.8)	50 (1.0)	189 (0.8)	13 (1.0)
Non-Alzheimer's dementia	1,299 (1.1)	540 (1.1)	54 (1.2)	47 (1.0)	303 (1.2)	27 (2.1)
Delirium	2,621 (2.1)	1,028 (2.1)	95 (2.0)	112 (2.3)	603 (2.5)	45 (3.5)
Parkinson's disease	1,116 (0.9)	454 (1.0)	46 (1.0)	43 (0.9)	202 (0.8)	21 (1.6)
**Pancreas**						
T1 or T2 diabetes	9,515 (8.0)	3,665 (7.9)	368 (8.1)	367 (7.6)	1,822 (7.7)	119 (9.6)
**Infection**						
Covid-19	2,332 (1.9)	956 (2.0)	96 (2.1)	77 (1.6)	522 (2.1)	35 (2.7)
Cholecystitis	1,624 (1.3)	613 (1.3)	55 (1.2)	73 (1.5)	318 (1.3)	24 (1.9)

Pneumonia	8,019 (6.7)	3,128 (6.7)	273 (6.0)	342 (7.1)	1,651 (6.9)	95 (7.6)
Sepsis	10,081 (8.4)	3,955 (8.4)	374 (8.2)	416 (8.6)	2,065 (8.6)	122 (9.7)
LRTI	4,113 (3.4)	1,694 (3.6)	148 (3.2)	164 (3.3)	914 (3.7)	50 (3.9)
URTI	588 (0.5)	226 (0.5)	21 (0.5)	17 (0.3)	109 (0.4)	4 (0.3)
UTI	7,099 (5.9)	2,773 (5.9)	263 (5.7)	290 (6.0)	1,472 (6.1)	96 (7.6)
SSTI	4,604 (3.8)	1,792 (3.8)	176 (3.8)	216 (4.5)	1,053 (4.4)	66 (5.2)
**Cardiovascular**						
Arrhythmia	2,283 (1.9)	871 (1.8)	86 (1.9)	77 (1.6)	445 (1.8)	23 (1.8)
Cardiomyopathy	855 (0.7)	330 (0.7)	25 (0.5)	34 (0.7)	179 (0.7)	6 (0.5)
CHD	11,796 (10.5)	4,626 (10.5)	412 (9.6)	485 (10.5)	2,422 (10.7)	133 (11.0)
Heart failure	5,779 (4.7)	2,289 (4.8)	211 (4.6)	229 (4.7)	1,256 (5.1)	68 (5.3)
**Mental Health**						
Depression	3,929 (3.4)	1,615 (3.5)	132 (3.0)	152 (3.2)	772 (3.3)	46 (3.8)

Incident disease numbers exclude prevalent disease at baseline. Numbers presented are n (%). Abbreviations: CHD, coronary heart disease; T1, type 1; T2, type 2; LRTI, lower respiratory tract infection; URTI, upper respiratory tract infection; UTI, urinary tract infection; SSTI, skin and soft tissue infection. Joint replacement surgery variable includes a diagnosis of hip, knee, ankle, or shoulder replacement. Any brain outcome variable includes a diagnosis of dementia, delirium, or Parkinson's disease.

For peer review only - http://bmjopen.bmj.com/site/about/guidelines.xhtml

eTable 5. Hazard ratios of incident disease outcomes in p.C282Y/H63D genotypes in males

**Males**	**No mutations**	**H63D +/-**		**H63D +/+**		**C282Y +/ H36D +**		**C282Y +/-**		**C282Y +/+**	
		HR (95% CI)	P	HR (95% CI)	P	HR (95% CI)	P	HR (95% CI)	P	HR (95% CI)	P
Hemochromatosis	1	1.53 (1.08-2.17)	0.02	8.95 (5.87-13.64)	$2.20*10^{-24}$	27.47 (20.49-36.84)	$1.00*10^{-108}$	2.40 (1.66-3.47)	$3.70*10^{-06}$	405.29 (317.06-518.11)	$2.97*10^{-501}$
All-cause mortality	1	1.03 (1.00-1.06)	0.05	1.01 (0.93-1.11)	0.75	1.02 (0.94-1.11)	0.58	1.05 (1.00-1.09)	0.03	1.29 (1.12-1.48)	$4.70*10^{-04}$
**Liver**											
Liver disease (any)	1	1.05 (0.99-1.11)	0.12	1.06 (0.90-1.24)	0.48	1.06 (0.91-1.23)	0.46	1.00 (0.93-1.78)	0.96	2.56 (2.10-3.12)	$8.70*10^{-21}$
Alcoholic liver disease	1	0.95 (0.81-1.11)	0.50	1.07 (0.71-1.59)	0.76	1.07 (0.73-1.57)	0.72	1.17 (0.98-1.40)	0.09	1.97 (1.16-3.35)	0.01
Fibrosis & Cirrhosis	1	1.04 (0.90-1.21)	0.58	1.02 (0.68-1.53)	0.93	1.00 (0.68-1.48)	0.99	1.11 (0.92-1.33)	0.28	5.36 (3.83-7.52)	$2.00*10^{-22}$
Hepatic failure	1	0.99 (0.81-1.21)	0.92	0.74 (0.40-1.40)	0.36	1.04 (0.62-1.74)	0.88	0.75 (0.56-1.00)	0.05	0.81 (0.26-2.52)	0.72
**Cancer**											
Liver cancer	1	1.17 (0.98-1.41)	0.09	0.86 (0.48-1.53)	0.61	1.32 (0.84-2.08)	0.22	1.07 (0.84-1.36)	0.61	7.90 (5.46-11.43)	$5.50*10^{-28}$
Prostate cancer	1	1.00 (0.96-1.05)	0.86	0.96 (0.86-1.09)	0.56	1.03 (0.93-1.16)	0.55	1.05 (1.00-1.11)	0.09	1.33 (1.09-1.61)	0.004
**Musculoskeletal**											
Joint replacement surgery (any)	1	1.03 (0.99-1.07)	0.21	0.98 (0.88-1.10)	0.74	1.10 (1.00-1.22)	0.08	1.06 (1.01-1.12)	0.02	1.78 (1.51-2.10)	$6.40*10^{-12}$
Osteoarthritis	1	1.03 (0.96-1.10)	0.44	0.89 (0.72-1.09)	0.25	1.01 (0.84-1.21)	0.99	1.03 (0.94-1.12)	0.55	2.10 (1.63-2.71)	$1.10*10^{-08}$
Fractures (any)	1	0.96 (0.92-1.01)	0.13	0.92 (0.80-1.05)	0.23	0.93 (0.82-1.06)	0.29	1.01 (0.95-1.07)	0.85	1.18 (0.94-1.48)	0.16
Fragility fractures	1	0.92 (0.86-0.99)	0.03	0.78 (0.62-0.98)	0.03	0.90 (0.74-1.10)	0.31	1.02 (0.93-1.12)	0.68	1.59 (1.18-2.14)	0.002
Osteoporosis	1	1.01 (0.92-1.11)	0.85	0.73 (0.53-0.99)	0.04	1.03 (0.80-1.32)	0.82	1.02 (0.91-1.16)	0.70	1.70 (1.16-2.48)	0.007
Rheumatoid arthritis	1	1.08 (0.97-1.21)	0.13	0.89 (0.64-1.23)	0.48	0.73 (0.51-1.03)	0.07	1.05 (0.91-1.20)	0.53	1.30 (0.78-2.16)	0.31
**Brain**											
Any brain outcome (dementia, delirium, or Parkinson's disease)	1	1.02 (0.97-1.08)	0.39	1.02 (0.88-1.18)	0.83	1.07 (0.93-1.24)	0.32	1.06 (0.99-1.13)	0.11	1.65 (1.31-2.06)	$1.70*10^{-05}$
Dementia	1	1.01 (0.94-1.09)	0.76	1.06 (0.86-1.31)	0.58	1.04 (0.85-1.27)	0.73	1.04 (0.94-1.14)	0.48	1.72 (1.26-2.35)	0.001
Alzheimer's disease	1	0.94 (0.84-1.06)	0.32	0.98 (0.71-1.36)	0.91	1.21 (0.91-1.60)	0.19	0.90 (0.77-1.05)	0.20	1.26 (0.73-2.17)	0.42
Non-Alzheimer's dementia	1	1.06 (0.96-1.17)	0.25	1.11 (0.84-1.45)	0.46	0.89 (0.66-1.19)	0.43	1.14 (1.01-1.30)	0.04	2.05 (1.40-3.00)	$2.40*10^{-04}$
Delirium	1	1.00 (0.93-1.08)	0.98	0.96 (0.78-1.18)	0.69	1.06 (0.87-1.28)	0.57	1.13 (1.04-1.24)	0.006	1.69 (1.26-2.27)	$4.80*10^{-04}$
Parkinson's disease	1	1.04 (0.93-1.16)	0.47	1.09 (0.81-1.47)	0.55	0.96 (0.71-1.30)	0.77	0.90 (0.77-1.04)	0.16	1.86 (1.21-2.87)	0.005
**Pancreas**											

For peer review only

For peer review only - http://bmjopen.bmj.com/site/about/guidelines.xhtml

T1 or T2 diabetes	1	0.98 (0.95-1.02)	0.44	1.02 (0.92-1.13)	0.69	0.95 (0.86-1.06)	0.35	0.95 (0.91-1.00)	0.06	1.25 (1.05-1.50)	0.01
**Infection**											
COVID-19	1	1.05 (0.97-1.13)	0.21	1.09 (0.89-1.34)	0.4	0.81 (0.65-1.02)	0.07	1.11 (1.01-1.22)	0.03	1.51 (1.08-2.11)	0.02
Cholecystitis	1	0.96 (0.88-1.06)	0.45	0.89 (0.68-1.16)	0.39	1.11 (0.87-1.40)	0.4	0.96 (0.86-1.09)	0.56	1.41 (0.94-2.11)	0.09
Pneumonia	1	1.00 (0.96-1.04)	0.82	0.90 (0.80-1.02)	0.09	1.04 (0.93-1.16)	0.51	1.01 (0.96-1.07)	0.68	1.14 (0.93-1.40)	0.21
Sepsis	1	1.01 (0.97-1.04)	0.78	0.98 (0.88-1.08)	0.66	1.02 (0.92-1.12)	0.7	1.01 (0.96-1.06)	0.70	1.17 (0.98-1.40)	0.09
LRTI	1	1.05 (0.99-1.11)	0.10	0.94 (0.80-1.11)	0.46	0.96 (0.82-1.12)	0.58	1.08 (1.00-1.16)	0.48	1.11 (0.84-1.46)	0.48
URTI	1	0.99 (0.84-1.15)	0.85	0.94 (0.61-1.45)	0.77	0.71 (0.44-1.15)	0.16	0.92 (0.75-1.13)	0.41	0.64 (0.24-1.71)	0.38
UTI	1	1.00 (0.95-1.04)	0.90	0.98 (0.86-1.10)	0.69	1.00 (0.89-1.13)	0.98	1.02 (0.96-1.08)	0.51	1.31 (1.07-1.61)	0.008
SSTI	1	1.00 (0.94-1.05)	0.88	1.01 (0.87-1.17)	0.90	1.16 (1.01-1.33)	0.03	1.14 (1.06-1.22)	$1.80*10^{-04}$	1.39 (1.09-1.77)	0.008
**Cardiovascular**											
Arrhythmia	1	0.98 (0.90-1.06)	0.57	1.00 (0.81-1.24)	0.99	0.84 (0.67-1.05)	0.13	0.96 (0.87-1.07)	0.49	0.97 (0.64-1.47)	0.89
Cardiomyopathy	1	0.99 (0.87-1.12)	0.88	0.77 (0.52-1.15)	0.21	0.99 (0.70-1.39)	0.94	1.04 (0.89-1.23)	0.60	0.69 (0.31-1.53)	0.36
CHD	1	1.01 (0.97-1.04)	0.77	0.91 (0.83-1.01)	0.08	1.00 (0.91-1.10)	0.99	1.02 (0.97-1.06)	0.45	1.06 (0.90-1.26)	0.49
Heart failure	1	1.01 (0.96-1.06)	0.67	0.96 (0.84-1.10)	0.59	0.97 (0.85-1.11)	0.65	1.07 (1.01-1.14)	0.03	1.14 (0.89-1.44)	0.29
**Mental Health**											
Depression	1	1.05 (0.99-1.12)	0.07	0.88 (0.74-1.05)	0.16	0.96 (0.81-1.13)	0.60	0.98 (0.91-1.06)	0.59	1.15 (0.86-1.54)	0.34

HR (Hazard ratio) compared to those with neither *HFE* mutation. Cox proportional hazards regression models adjusted for age, assessment centre, and genetic principal components 1–10. Abbreviations: CHD, coronary heart disease; T1, type 1; T2, type 2; LRTI, lower respiratory tract infection; URTI, upper respiratory tract infection; UTI, urinary tract infection; SSTI, skin and soft tissue infection; CI, confidence interval. Joint replacement surgery variable includes a diagnosis of hip, knee, ankle, or shoulder replacement. Any brain outcome variable includes a diagnosis of dementia, delirium, or Parkinson's disease.

For peer review only

For peer review only - http://bmjopen.bmj.com/site/about/guidelines.xhtml

eTable 6. Incident hospital diagnoses in male UK Biobank participants by p.C282Y/H63D genotypes, excluding a diagnosis of hemochromatosis at baseline

**Males**	**No mutations**	**H63D +/-**	**H63D +/+**	**C282Y +/ H36D +**	**C282Y +/-**	**C282Y +/+**
Hemochromatosis	85 (14.41)	51 (8.64)	29 (4.92)	95 (16.10)	42 (7.12)	288 (48.81)
All-cause mortality	13959 (58.44)	5653 (23.66)	539 (2.26)	587 (2.46)	2990 (12.52)	160 (0.67)
**Liver**						
Liver disease (any)	3969 (58.38)	1619 (23.82)	158 (2.32)	167 (2.46)	800 (11.77)	85 (1.25)
Alcoholic liver disease	607 (58.20)	226 (21.67)	25 (2.40)	27 (2.59)	148 (14.19)	10 (0.96)
Fibrosis & Cirrhosis	618 (57.06)	250 (23.08)	23 (2.12)	25 (2.31)	140 (12.93)	27 (2.49)
Hepatic failure	355 (62.06)	137 (23.95)	10 (1.75)	15 (2.65)	54 (9.44)	1 (0.17)
**Cancer**						
Liver cancer	361 (55.28)	166 (25.42)	12 (1.84)	19 (2.91)	78 (11.94)	17 (2.60)
Prostate cancer	7722 (59.20)	3023 (23.18)	283 (2.17)	318 (2.44)	1610 (12.34)	87 (0.67)
**Musculoskeletal**						
Joint replacement surgery (any)	8427 (58.44)	3377 (23.42)	315 (2.18)	375 (2.60)	1801 (12.49)	126 (0.87)
Osteoarthritis	2872 (58.70)	1153 (23.56)	97 (1.98)	120 (2.45)	600 (12.26)	51 (1.04)
Fractures (any)	6090 (60.07)	2296 (22.65)	212 (2.09)	231 (2.28)	1243 (12.26)	66 (0.65)
Fragility fractures	2663 (60.63)	963 (21.93)	79 (1.80)	98 (2.23)	550 (12.52)	39 (0.89)
Osteoporosis	1521 (59.37)	601 (23.46)	42 (1.64)	63 (2.46)	319 (12.45)	16 (0.62)
Rheumatoid arthritis	1130 (58.55)	479 (24.82)	38 (1.97)	33 (1.71)	238 (12.33)	12 (0.62)
**Brain**						
Any brain outcome (dementia, delirium, or Parkinson's disease)	4616 (58.43)	1849 (23.41)	177 (2.24)	200 (2.53)	992 (12.56)	66 (0.84)
Dementia	2305 (58.67)	915 (23.29)	92 (2.34)	97 (2.47)	487 (12.40)	33 (0.84)
Alzheimer's disease	1026 (60.53)	380 (22.42)	38 (2.24)	50 (2.95)	189 (11.15)	12 (0.71)
Non-Alzheimer's dementia	1298 (57.38)	539 (23.83)	54 (2.39)	47 (2.08)	303 (13.40)	21 (0.93)
Delirium	2619 (58.29)	1025 (22.81)	95 (2.11)	111 (2.47)	602 (13.40)	41 (0.91)
Parkinson's disease	1115 (59.34)	454 (24.16)	46 (2.45)	43 (2.29)	202 (10.75)	19 (1.01)
**Pancreas**						

T2 diabetes	9570 (60.17)	3673 (23.09)	365 (2.30)	368 (2.31)	1831 (11.51)	97 (0.61)
**Infection**						
Covid-19	2331 (58.14)	953 (23.77)	96 (2.39)	76 (1.90)	522 (13.02)	31 (0.77)
Cholecystitis	1623 (60.07)	612 (22.65)	55 (2.04)	73 (2.70)	316 (11.70)	23 (0.85)
Pneumonia	8014 (59.44)	3124 (23.17)	273 (2.02)	340 (2.52)	1649 (12.23)	83 (0.62)
Sepsis	10075 (59.34)	3951 (23.27)	374 (2.20)	412 (2.43)	2063(12.15)	103 (0.61)
LRTI	4110 (58.12)	1691 (23.91)	148 (2.09)	164 (2.32)	913 (12.91)	45 (0.64)
URTI	588 (61.06)	226 (23.47)	21 (2.18)	17 (1.77)	108 (11.21)	3 (0.31)
UTI	7097 (59.29)	2769 (23.13)	263 (2.20)	286 (2.39)	1470 (12.28)	85 (0.71)
SSTI	4599 (58.27)	1790 (22.68)	176 (2.23)	215 (2.72)	1053 (13.34)	59 (0.75)
**Cardiovascular**						
Arrhythmia	2282 (60.40)	870 (23.03)	85 (2.25)	77 (2.04)	444 (11.75)	20 (0.53)
Cardiomyopathy	853 (59.86)	330 (23.16)	25 (1.75)	34 (2.39)	178 (12.49)	5 (0.35)
CHD	11793 (59.45)	4622 (23.30)	411 (2.07)	481 (2.42)	2414 (12.17)	115 (0.58)
Heart failure	5775 (58.87)	2285 (23.29)	211 (2.15)	227 (2.31)	1253 (12.77)	59 (0.60)
**Mental Health**						
Depression	3925 (59.18)	1614 (24.34)	132 (1.99)	150 (2.26)	771 (11.63)	40 (0.60)

Incident disease numbers exclude prevalent disease at baseline. Numbers presented are n (%). Abbreviations: CHD, coronary heart disease; T1, type 1; T2, type 2; LRTI, lower respiratory tract infection; URTI, upper respiratory tract infection; UTI, urinary tract infection; SSTI, skin and soft tissue infection. Joint replacement surgery variable includes a diagnosis of hip, knee, ankle, or shoulder replacement. Any brain outcome variable includes a diagnosis of dementia, delirium, or Parkinson's disease.

For peer review only

eTable 7. Hazard ratios of incident disease outcomes in p.C282Y/H63D genotypes in males, excluding a diagnosis of hemochromatosis at baseline

Males	No mutations (Ref group)	H63D +/-		H63D +/+		C282Y +/ H36D +		C282Y +/-		C282Y +/+	
		HR (95% CI)	P	HR (95% CI)	P	HR (95% CI)	P	HR (95% CI)	P	HR (95% CI)	P
Hemochromatosis	1	1.53 (1.08-2.17)	0.02	8.95 (5.87-13.64)	$2.2*10^{-24}$	27.49 (20.50-36.86)	$1.0*10^{-108}$	2.40 (1.66-3.47)	$3.6*10^{-06}$	405.79 (317.34-518.75)	0.00E+00
All-cause mortality	1	1.03 (1.00-1.07)	0.04	1.01 (0.93-1.11)	0.74	1.02 (0.94-1.11)	0.58	1.05 (1.00-1.09)	0.03	1.22 (1.05-1.43)	0.01
**Liver**											
Liver disease (any)	1	1.05 (0.99-1.11)	0.13	1.05 (0.89-1.23)	0.57	1.04 (0.89-1.21)	0.63	1.00 (0.92-1.07)	0.9	2.36 (1.90-2.93)	$5.3*10^{-15}$
Alcoholic liver disease	1	0.95 (0.12-1.10)	0.48	1.07 (0.72-1.60)	0.74	1.04 (0.71-1.53)	0.84	1.15 (0.96-1.38)	0.12	1.59 (0.85-2.97)	0.15
Fibrosis & Cirrhosis	1	1.03 (0.89-1.20)	0.66	0.98 (0.64-1.48)	0.91	0.97 (0.65-1.44)	0.87	1.09 (0.91-1.31)	0.35	4.52 (3.07-6.66)	$2.1*10^{-14}$
Hepatic failure	1	0.99 (0.81-1.21)	0.92	0.75 (0.40-1.40)	0.36	1.05 (0.62-1.76)	0.86	0.75 (0.56-1.00)	0.05	0.31 (0.04-2.19)	0.24
**Cancer**											
Liver cancer	1	1.17 (0.98-1.41)	0.09	0.87 (0.49-1.54)	0.63	1.27 (0.80-2.02)	0.3	1.05 (0.82-1.34)	0.71	4.97 (3.05-8.11)	$1.2*10^{-10}$
Prostate cancer	1	1.00 (0.96-1.05)	0.87	0.97 (0.86-1.09)	0.57	1.04 (0.93-1.16)	0.51	1.05 (0.99-1.11)	0.09	1.27 (1.03-1.57)	0.03
**Musculoskeletal**											
Joint replacement surgery (any)	1	1.03 (0.99-1.07)	0.22	0.98 (0.88-1.10)	0.75	1.10 (0.99-1.22)	0.06	1.06 (1.01-1.12)	0.02	1.75 (1.47-2.09)	$4.8*10^{-10}$
Osteoarthritis	1	1.03 (0.96-1.10)	0.46	0.89 (0.73-1.09)	0.26	1.02 (0.85-1.22)	0.87	1.03 (0.94-1.12)	0.56	1.97 (1.50-2.60)	$1.5*10^{-06}$
Fractures (any)	1	0.96 (0.92-1.01)	0.12	0.92 (0.80-1.06)	0.24	0.94 (0.82-1.07)	0.34	1.01 (0.95-1.07)	0.82	1.17 (0.91-1.49)	0.22
Fragility fractures	1	0.92 (0.86-0.99)	0.03	0.78 (0.63-0.98)	0.03	0.91 (0.74-1.11)	0.34	1.02 (0.93-1.12)	0.68	1.60 (1.17-2.20)	$3.6*10^{-03}$
Osteoporosis	1	1.01 (0.92-1.11)	0.86	0.73 (0.54-0.99)	0.04	1.02 (0.79-1.31)	0.89	1.03 (0.91-1.16)	0.68	1.14 (0.69-1.86)	0.61
Rheumatoid arthritis	1	1.09 (0.98-1.21)	0.13	0.89 (0.64-1.23)	0.48	0.73 (0.52-1.03)	0.07	1.05 (0.91-1.20)	0.52	1.17 (0.66-2.07)	0.58
**Brain**											
Any brain outcome (dementia, delirium, or Parkinson's disease)	1	1.02 (0.97-1.08)	0.4	1.02 (0.88-1.18)	0.8	1.08 (0.93-1.24)	0.31	1.06 (0.99-1.13)	0.1	1.62 (1.27-2.07)	$1.0*10^{-04}$
Dementia	1	1.01 (0.94-1.09)	0.76	1.06 (0.86-1.31)	0.57	1.04 (0.85-1.28)	0.69	1.04 (0.94-1.14)	0.46	1.60 (1.14-2.26)	0.01
Alzheimer's disease	1	0.94 (0.84-1.06)	0.32	0.98 (0.71-1.36)	0.92	1.12 (0.91-1.61)	0.18	0.90 (0.77-1.06)	0.2	1.31 (0.74-2.31)	0.35
Non-Alzheimer's dementia	1	1.06 (0.96-1.17)	0.26	1.11 (0.85-1.46)	0.45	0.89 (0.67-1.20)	0.45	1.14 (1.01-1.30)	0.04	1.81 (1.17-2.78)	0.01
Delirium	1	1.00 (0.93-1.07)	0.98	0.96 (0.78-1.18)	0.7	1.05 (0.87-1.27)	0.59	1.13 (1.04-1.24)	0.01	1.75 (1.28-2.38)	$3.9*10^{-04}$
Parkinson's disease	1	1.07 (0.98-1.16)	0.13	1.05 (0.84-1.31)	0.67	1.06 (0.85-1.33)	0.58	1.01 (0.91-1.13)	0.79	1.44 (1.02-2.03)	0.04

For peer review only

**Pancreas**											
T1 or T2 diabetes	1	0.99 (0.95-1.02)	0.45	1.02 (0.92-1.14)	0.66	0.94 (0.85-1.05)	0.28	0.95 (0.91-1.00)	0.06	1.17 (0.96-1.43)	0.11
**Infection**											
COVID-19	1	1.05 (0.97-1.13)	0.23	1.09 (0.89-1.34)	0.39	0.81 (0.64-1.01)	0.07	1.11 (1.01-1.23)	0.03	1.51 (1.06-2.15)	0.02
Cholecystitis	1	0.96 (0.88-1.06)	0.44	0.89 (0.68-1.17)	0.4	1.11 (0.88-1.41)	0.37	0.96 (0.85-1.08)	0.51	1.54 (1.02-2.32)	0.04
Pneumonia	1	0.99 (0.95-1.04)	0.8	0.90 (0.80-1.02)	0.09	1.04 (0.93-1.16)	0.52	1.01 (0.96-1.07)	0.66	1.13 (0.91-1.40)	0.27
Sepsis	1	1.01 (0.97-1.04)	0.79	0.98 (0.88-1.09)	0.69	1.01 (0.92-1.12)	0.82	1.01 (0.96-1.06)	0.68	1.12 (0.92-1.36)	0.26
LRTI	1	1.05 (0.99-1.11)	0.1	0.94 (0.80-1.11)	0.48	0.96 (0.82-1.13)	0.63	1.08 (1.00-1.16)	0.04	1.14 (0.85-1.52)	0.4
URTI	1	0.99 (0.85-1.15)	0.85	0.94 (0.61-1.45)	0.78	0.71 (0.44-1.16)	0.17	0.91 (0.74-1.12)	0.37	0.55 (0.18-1.70)	0.3
UTI	1	1.00 (0.95-1.04)	0.86	0.98 (0.86-1.11)	0.71	1.00 (0.89-1.12)	0.96	1.02 (0.96-1.08)	0.51	1.32 (1.07-1.64)	0.01
SSTI	1	1.00 (0.94-1.05)	0.89	1.01 (0.87-1.18)	0.87	1.16 (1.01-1.33)	0.03	1.14 (1.07-1.22)	$1.4*10^{-04}$	1.40 (1.09-1.82)	0.01
**Cardiovascular**											
Arrhythmia	1	0.98 (0.90-1.06)	0.56	0.99 (0.80-1.23)	0.92	0.84 (0.67-1.06)	0.15	0.96 (0.87-1.07)	0.48	0.96 (0.62-1.49)	0.86
Cardiomyopathy	1	0.99 (0.87-1.13)	0.91	0.78 (0.52-1.16)	0.21	0.99 (0.71-1.40)	0.98	1.04 (0.89-1.22)	0.62	0.65 (0.27-1.57)	0.34
CHD	1	1.00 (0.97-1.04)	0.8	0.91 (0.83-1.01)	0.71	1.00 (0.91-1.09)	0.97	1.01 (0.97-1.06)	0.52	1.04 (0.87-1.25)	0.66
Heart failure	1	1.01 (0.96-1.06)	0.69	0.96 (0.84-1.11)	0.61	0.97 (0.85-1.10)	0.62	1.07 (1.01-1.14)	0.03	1.12 (0.87-1.45)	0.38
**Mental Health**											
Depression	1	1.05 (1.00-1.12)	0.07	0.88 (0.74-1.05)	0.17	0.95 (0.81-1.12)	0.55	0.98 (0.91-1.06)	0.6	1.13 (0.82-1.54)	0.46

HR (Hazard ratio) compared to those with neither *HFE* mutation. Cox proportional hazards regression models adjusted for age, assessment centre, and genetic principal components 1–10. Abbreviations: CHD, coronary heart disease; T1, type 1; T2, type 2; LRTI, lower respiratory tract infection; URTI, upper respiratory tract infection; UTI, urinary tract infection SSTI, skin and soft tissue infection; CI, confidence interval. Joint replacement surgery variable includes a diagnosis of hip, knee, ankle, or shoulder replacement. Any brain outcome variable includes a diagnosis of dementia, delirium, or Parkinson's disease.

For peer review only - http://bmjopen.bmj.com/site/about/guidelines.xhtml

eTable 8. Cumulative incidence of hemochromatosis from ages 40-80 years by HFE genotypes

	Total Cohort (n=451,270)			No mutations			H63D Heterozygotes			H63D Homozygotes			Compound C282Y/H63D Heterozygotes			p.C282Y Heterozygotes			p.C282Y homozygotes		
	Incident diagnosis			Incident diagnosis			Incident diagnosis			Incident diagnosis			Incident diagnosis			Incident diagnosis			Incident diagnosis		
	%	(95% CI)		%	(95% CI)		%	(95% CI)		%	(95% CI)		%	(95% CI)		%	(95% CI)		%	(95% CI)	
**Male age group (years)**																					
**40 - 45**	0.1	0.0	0.2	0.0	0.0	0.2	0.0	0.0	0.0	0.0	0.0	0.0	0.3	0.0	1.9	0.0	0.0	0.0	6.8	2.8	16.1
**46 - 50**	0.1	0.1	0.2	0.0	0.0	0.2	0.0	0.0	0.1	0.4	0.1	1.3	0.7	0.2	2.0	0.0	0.0	0.0	12.5	7.3	20.8
**51 - 55**	0.2	0.2	0.3	0.0	0.0	0.2	0.0	0.0	0.1	0.6	0.3	1.5	1.5	0.8	2.6	0.0	0.0	0.1	20.4	14.7	27.9
**56 - 60**	0.3	0.3	0.4	0.1	0.0	0.2	0.1	0.0	0.1	0.8	0.4	1.6	2.1	1.3	3.2	0.1	0.1	0.2	28.4	22.7	35.3
**61 - 65**	0.4	0.3	0.5	0.1	0.0	0.2	0.1	0.1	0.2	1.0	0.6	1.8	2.6	1.8	3.8	0.2	0.1	0.3	36.1	30.5	42.4
**66 - 70**	0.5	0.5	0.6	0.1	0.1	0.2	0.2	0.1	0.2	1.2	0.7	2.1	3.6	2.7	4.8	0.3	0.2	0.4	43.0	37.6	48.7
**71 - 75**	0.6	0.6	0.7	0.2	0.1	0.2	0.2	0.1	0.3	1.4	0.9	2.2	4.3	3.4	5.5	0.3	0.2	0.4	48.9	43.8	54.2
**76 - 80**	0.8	0.7	0.9	0.2	0.1	0.3	0.3	0.2	0.4	1.9	1.3	2.9	5.4	4.3	6.8	0.4	0.3	0.6	56.4	51.4	61.6
**Female age group (years)**																					
**40 - 45**	0.0	0.0	0.0	0.0	0.0	0.0	0.0	0.0	0.0	0.0	0.0	0.0	0.2	0.0	1.5	0.0	0.0	0.0	0.0	0.0	0.0
**46 - 50**	0.0	0.0	0.1	0.0	0.0	0.0	0.0	0.0	0.1	0.2	0.1	0.9	0.3	0.1	1.3	0.0	0.0	0.0	3.4	1.7	6.7
**51 - 55**	0.1	0.1	0.1	0.0	0.0	0.0	0.0	0.0	0.1	0.3	0.1	1.0	0.5	0.2	1.3	0.0	0.0	0.1	8.2	5.7	11.7
**56 - 60**	0.1	0.1	0.2	0.0	0.0	0.0	0.1	0.0	0.1	0.4	0.2	1.0	0.7	0.4	1.5	0.1	0.1	0.2	14.5	11.5	18.1
**61 - 65**	0.2	0.2	0.3	0.0	0.0	0.0	0.1	0.0	0.1	0.5	0.2	1.1	1.1	0.6	1.8	0.1	0.1	0.2	21.2	17.9	24.9
**66 - 70**	0.3	0.3	0.3	0.0	0.0	0.1	0.1	0.1	0.2	0.5	0.2	1.1	1.5	1.0	2.2	0.2	0.1	0.3	27.6	24.2	31.3
**71 - 75**	0.4	0.3	0.4	0.1	0.0	0.1	0.1	0.1	0.2	0.6	0.3	1.2	1.9	1.4	2.7	0.2	0.2	0.3	33.5	30.0	37.2
**76 - 80**	0.5	0.5	0.6	0.1	0.1	0.1	0.1	0.1	0.2	0.6	0.3	1.3	2.7	2.0	3.6	0.3	0.2	0.5	40.5	36.7	44.5

For peer review only - http://bmjopen.bmj.com/site/about/guidelines.xhtml

eTable 9. Incident hospital diagnoses in female UK Biobank participants by p.C282Y/H63D genotypes

Females	No mutations	H63D +/-	H63D +/+	C282Y +/ H36D +	C282Y +/-	C282Y +/+
Hemochromatosis	44 (0.03)	35 (0.1)	12 (0.2)	57 (1.0)	37 (0.1)	291 (18.8)
All-cause mortality	9,849 (6.8)	3,810 (6.7)	363 (6.5)	393 (6.8)	2,020 (6.9)	129 (8.0)
**Liver**						
Liver disease (any)	3,822 (2.6)	1,512 (2.7)	146 (2.6)	179 (3.1)	825 (2.9)	69 (4.3)
Alcoholic liver disease	181 (0.1)	78 (0.1)	11 (0.2)	6 (0.1)	59 (0.2)	7 (0.4)
Fibrosis & Cirrhosis	475 (0.3)	163 (0.3)	16 (0.3)	21 (0.4)	89 (0.3)	14 (0.9)
Hepatic failure	200 (0.1)	60 (0.1)	7 (0.1)	4 (0.1)	35 (0.1)	5 (0.3)
**Cancer**						
Liver cancer	232 (0.2)	114 (0.2)	10 (0.2)	8 (0.1)	49 (0.2)	3 (0.2)
**Musculoskeletal**						
Joint replacement surgery (any)	12,356 (8.7)	4,772 (8.6)	454 (8.4)	501 (8.9)	2,415 (8.5)	158 (10.3)
Osteoarthritis	4,344 (3.3)	1,681 (3.3)	177 (3.6)	190 (3.7)	893 (3.4)	70 (5.1)
Fractures (any)	10,666 (7.6)	4,204 (7.7)	376 (7.0)	430 (7.7)	2,189 (7.8)	124 (8.0)
Fragility fractures	6,308 (4.4)	2,517 (4.5)	214 (3.9)	275 (4.8)	1,265 (4.4)	63 (4.0)
Osteoporosis	6,990 (5.0)	2,580 (4.7)	254 (4.7)	245 (4.4)	1,390 (4.9)	103 (6.7)
Rheumatoid arthritis	2,189 (1.5)	819 (1.5)	79 (1.4)	87 (1.5)	391 (1.4)	26 (1.6)
**Brain**						
Any brain outcome (dementia, delirium, or Parkinson's disease)	3,876 (2.7)	1,547 (2.7)	142 (2.6)	155 (2.7)	777 (2.7)	61 (3.8)
Dementia	2,159 (1.5)	837 (1.5)	68 (1.2)	84 (1.5)	440 (1.5)	31 (1.9)
Alzheimer's disease	1,072 (0.7)	450 (0.8)	37 (0.7)	51 (0.9)	220 (0.8)	14 (0.9)
Non-Alzheimer's dementia	1,099 (0.8)	395 (0.7)	31 (0.6)	34 (0.6)	227 (0.8)	17 (1.1)
Delirium	1,983 (1.4)	823 (1.4)	81 (1.5)	82 (1.4)	406 (1.4)	36 (2.2)
Parkinson's disease	691 (0.5)	249 (0.4)	26 (0.5)	30 (0.5)	134 (0.5)	8 (0.5)
**Pancreas**						
T1 or T2 diabetes	6,532 (4.6)	2,583 (4.6)	245 (4.5)	278 (4.9)	1,327 (4.6)	87 (5.5)
**Infection**						
Covid-19	1,809 (1.2)	720 (1.3)	72 (1.3)	72 (1.3)	383 (1.3)	21 (1.3)
Cholecystitis	1,749 (1.2)	675 (1.2)	62 (1.1)	69 (1.2)	351 (1.2)	30 (1.9)
Pneumonia	6,130 (4.3)	2,408 (4.3)	242 (4.4)	255 (4.5)	1,276 (4.5)	75 (4.8)

For peer review only - http://bmjopen.bmj.com/site/about/guidelines.xhtml

Sepsis	10,026 (7.0)	3,840 (6.9)	379 (7.0)	414 (7.4)	2,103 (7.4)	133 (8.5)
LRTI	3,875 (2.7)	1,439 (2.5)	139 (2.5)	144 (2.5)	821 (2.8)	53 (3.3)
URTI	636 (0.4)	279 (0.5)	21 (0.4)	21 (0.4)	142 (0.5)	2 (0.1)
UTI	9,140 (6.5)	3,493 (6.4)	351 (6.6)	366 (6.7)	1,905 (6.8)	126 (8.2)
SSTI	3,900 (2.7)	1,534 (2.7)	158 (2.9)	153 (2.7)	799 (2.8)	53 (3.3)
**Cardiovascular**						
Arrhythmia	1,655 (1.1)	656 (1.2)	48 (0.9)	67 (1.2)	323 (1.1)	20 (1.3)
Cardiomyopathy	608 (0.4)	230 (0.4)	19 (0.3)	26 (0.5)	130 (0.5)	3 (0.2)
CHD	7,402 (5.3)	2,877 (5.2)	294 (5.4)	293 (5.3)	1,502 (5.3)	82 (5.3)
Heart failure	3,551 (2.4)	1,431 (2.5)	127 (2.3)	155 (2.7)	753 (2.6)	56 (3.5)
**Mental Health**						
Depression	6,415 (4.8)	2430 (4.6)	269 (5.2)	279 (5.2)	1317 (4.9)	64 (4.3)

Incident disease numbers exclude prevalent disease at baseline. Numbers presented are n (%). Abbreviations: CHD, coronary heart disease; T1, type 1; T2, type 2; LRTI, lower respiratory tract infection; URTI, upper respiratory tract infection; UTI, urinary tract infection; SSTI, skin and soft tissue infection. Joint replacement surgery variable includes a diagnosis of hip, knee, ankle, or shoulder replacement. Any brain outcome variable includes a diagnosis of dementia, delirium, or Parkinson's disease.

For peer review only - http://bmjopen.bmj.com/site/about/guidelines.xhtml

eTable 10. Hazard ratios of incident disease outcomes in p.C282Y/H63D genotypes in females

**Females**	**No mutations**	**H63D +/-**		**H63D +/+**		**C282Y +/ H36D +**		**C282Y +/-**		**C282Y +/+**	
		HR (95% CI)	P	HR (95% CI)	P	HR (95% CI)	P	HR (95% CI)	P	HR (95% CI)	P
Hemochromatosis	1	2.04 (1.31-3.17)	$1.70*10^{-03}$	7.10 (3.75-13.45)	$1.80*10^{-09}$	32.78 (22.10-48.63)	$2.20*10^{-67}$	4.15 (2.68-6.44)	$1.80*10^{-10}$	674.10 (489.11-929.05)	$2.47*10^{-346}$
All-cause mortality	1	0.99 (0.96-1.03)	0.68	0.95 (0.85-1.05)	0.3	1.01 (0.91-1.11)	0.9	1.01 (0.96-1.06)	0.62	1.10 (0.92-1.31)	0.28
**Liver**											
Liver disease (any)	1	1.01 (0.95-1.07)	0.72	1.00 (0.84-1.17)	0.96	1.19 (1.02-1.38)	0.03	1.08 (1.00-1.16)	0.06	1.62 (1.27-2.05)	$7.80*10^{-05}$
Alcoholic liver disease	1	1.09 (0.84-1.43)	0.51	1.56 (0.85-2.87)	0.15	0.78 (0.35-1.77)	0.56	1.53 (1.14-2.05)	0.005	3.07 (1.44-6.54)	0.004
Fibrosis & Cirrhosis	1	0.87 (0.73-1.04)	0.13	0.87 (0.53-1.43)	0.58	1.10 (0.71-1.71)	0.67	0.92 (0.74-1.16)	0.49	2.56 (1.50-4.36)	0.001
Hepatic failure	1	0.77 (0.58-1.02)	0.07	0.92 (0.43-1.94)	0.82	0.49 (0.18-1.33)	0.16	0.85 (0.59-1.21)	0.37	2.09 (0.86-5.08)	0.1
**Cancer**											
Liver cancer	1	1.26 (1.01-1.58)	0.04	1.13 (0.60-2.13)	0.71	0.89 (0.44-1.81)	0.75	1.06 (0.78-1.45)	0.69	1.17 (0.37-3.66)	0.79
**Musculoskeletal**											
Joint replacement surgery (any)	1	0.99 (0.96-1.02)	0.55	0.97 (0.88-1.06)	0.47	1.04 (0.95-1.14)	0.34	0.99 (0.94-1.03)	0.51	1.18 (1.01-1.38)	0.04
Osteoarthritis	1	0.99 (0.94-1.05)	0.71	1.06 (0.91-1.23)	0.46	1.10 (0.95-1.27)	0.21	1.02 (0.95-1.10)	0.6	1.44 (1.14-1.82)	0.002
Fractures (any)	1	1.01 (0.97-1.04)	0.65	0.91 (0.82-1.01)	0.07	1.01 (0.92-1.12)	0.79	1.02 (0.97-1.07)	0.42	1.00 (0.84-1.20)	0.96
Fragility fractures	1	1.02 (0.98-1.07)	0.38	0.87 (0.76-1.00)	0.05	1.10 (0.97-1.24)	0.13	0.99 (0.93-1.05)	0.74	0.85 (0.66-1.09)	0.19
Osteoporosis	1	0.94 (0.90-0.99)	0.01	0.94 (0.83-1.07)	0.34	0.89 (0.79-1.01)	0.08	0.99 (0.94-1.05)	0.75	1.28 (1.06-1.56)	0.01
Rheumatoid arthritis	1	0.96 (0.88-1.04)	0.28	0.94 (0.75-1.17)	0.58	1.01 (0.82-1.26)	0.9	0.89 (0.80-0.99)	0.03	1.04 (0.71-1.53)	0.84
**Brain**											
Any brain outcome (dementia, delirium, or Parkinson's disease)	1	1.02 (0.97-1.09)	0.43	0.93 (0.79-1.11)	0.43	1.03 (0.88-1.21)	0.72	1.00 (0.92-1.08)	0.9	1.30 (1.01-1.68)	0.04
Dementia	1	1.00 (0.92-1.08)	0.94	0.80 (0.63-1.02)	0.07	1.00 (0.81-1.25)	0.98	1.01 (0.91-1.12)	0.86	1.16 (0.81-1.66)	0.41
Alzheimer's disease	1	1.08 (0.97-1.20)	0.18	0.87 (0.63-1.20)	0.4	1.22 (0.92-1.61)	0.17	1.02 (0.88-1.17)	0.84	1.04 (0.62-1.77)	0.88

For peer review only

Non-Alzheimer's dementia	1	0.93 (0.83-1.04)	0.2	0.72 (0.50-1.03)	0.07	0.81 (0.57-1.13)	0.21	1.03 (0.89-1.18)	0.73	1.27 (0.76-2.05)	0.33
Delirium	1	1.06 (0.98-1.15)	0.13	1.05 (0.84-1.31)	0.67	1.06 (0.85-1.32)	0.6	1.01 (0.91-1.13)	0.8	1.50 (1.08-2.08)	0.02
Parkinson's disease	1	0.93 (0.80-1.07)	0.29	0.97 (0.66-1.44)	0.89	1.14 (0.79-1.65)	0.48	0.98 (0.81-1.18)	0.84	1.00 (0.50-2.00)	0.99
**Pancreas**											
T1 or T2 diabetes	1	1.01 (0.97-1.06)	0.55	0.98 (0.86-1.11)	0.77	1.09 (0.97-1.23)	0.17	1.01 (0.96-1.08)	0.6	1.18 (0.95-1.46)	0.13
**Infection**											
COVID-19	1	1.02 (0.94-1.11)	0.66	1.03 (0.81-1.30)	0.83	1.02 (0.80-1.29)	0.9	1.06 (0.94-1.18)	0.34	1.00 (0.65-1.54)	0.99
Cholecystitis	1	0.98 (0.90-1.08)	0.73	0.92 (0.71-1.18)	0.52	0.99 (0.78-1.26)	0.92	0.99 (0.88-1.11)	0.86	1.49 (1.04-2.13)	0.03
Pneumonia	1	1.01 (0.96-1.05)	0.83	1.02 (0.90-1.16)	0.72	1.06 (0.93-1.20)	0.4	1.03 (0.97-1.10)	0.29	1.05 (0.83-1.32)	0.69
Sepsis	1	0.98 (0.94-1.01)	0.23	0.97 (0.88-1.08)	0.61	1.05 (0.95-1.15)	0.36	1.04 (0.99-1.09)	0.12	1.13 (0.96-1.35)	0.15
LRTI	1	0.95 (0.89-1.01)	0.08	0.92 (0.78-1.09)	0.34	0.93 (0.79-1.10)	0.39	1.04 (0.97-1.12)	0.3	1.16 (0.89-1.52)	0.28
URTI	1	1.12 (0.98-1.30)	0.1	0.86 (0.56-1.33)	0.51	0.84 (0.54-1.30)	0.43	1.12 (0.93-1.34)	0.24	0.28 (0.07-1.12)	0.07
UTI	1	0.97 (0.94-1.01)	0.18	0.99 (0.89-1.10)	0.82	1.01 (0.91-1.13)	0.8	1.03 (0.98-1.08)	0.23	1.19 (0.99-1.41)	0.06
SSTI	1	1.01 (0.95-1.07)	0.79	1.05 (0.90-1.23)	0.54	1.00 (0.85-1.18)	0.97	1.03 (0.95-1.11)	0.52	1.20 (0.92-1.58)	0.18
**Cardiovascular**											
Arrhythmia	1	1.02 (0.93-1.11)	0.69	0.76 (0.57-1.01)	0.06	1.04 (0.81-1.33)	0.75	0.98 (0.87-1.11)	0.78	1.07 (0.69-1.67)	0.75
Cardiomyopathy	1	0.97 (0.83-1.13)	0.71	0.82 (0.52-1.30)	0.41	1.11 (0.75-1.64)	0.61	1.08 (0.90-1.31)	0.41	0.44 (0.14-1.37)	0.16
CHD	1	1.00 (0.95-1.04)	0.82	1.03 (0.92-1.16)	0.61	1.01 (0.90-1.14)	0.86	1.01 (0.96-1.07)	0.71	0.94 (0.76-1.17)	0.6
Heart failure	1	1.04 (0.97-1.10)	0.27	0.92 (0.77-1.10)	0.35	1.12 (0.96-1.32)	0.15	1.06 (0.98-1.15)	0.16	1.34 (1.03-1.75)	0.03
**Mental Health**											
Depression	1	0.97 (0.93-1.02)	0.2	1.10 (0.97-1.24)	0.14	1.11 (0.99-1.25)	0.09	1.03 (0.97-1.09)	0.38	0.90 (0.70-1.15)	0.39

HR (Hazard ratio) compared to those with neither *HFE* mutation. Cox proportional hazards regression models adjusted for age, assessment centre, and genetic principal components 1–10. Abbreviations: CHD, coronary heart disease; T1, type 1; T2, type 2; LRTI, lower respiratory tract infection; URTI, upper respiratory tract infection; UTI, urinary tract infection SSTI, skin and soft tissue infection; CI, confidence interval. Joint replacement surgery variable includes a diagnosis of hip, knee, ankle, or shoulder replacement. Any brain outcome variable includes a diagnosis of dementia, delirium, or Parkinson's disease.

For peer review only - http://bmjopen.bmj.com/site/about/guidelines.xhtml

eTable 11. Incident hospital diagnoses in female UK Biobank participants by p.C282Y/H63D genotypes, excluding a diagnosis of hemochromatosis at baseline

**Females**	**No mutations**	**H63D +/-**	**H63D +/+**	**C282Y +/ H36D +**	**C282Y +/-**	**C282Y +/+**
Hemochromatosis	44 (9.24)	35 (7.35)	12 (2.52)	57 (11.97)	37 (7.77)	291 (61.13)
All-cause mortality	9847 (59.47)	3810 (23.01)	363 (2.19)	390 (2.36)	2020 (12.20)	127 (0.77)
**Liver**						
Liver disease (any)	3821 (58.38)	1511 (23.09)	146 (2.23)	177 (2.70)	825 (12.61)	65 (0.99)
Alcoholic liver disease	180 (52.94)	78 (22.94)	11 (3.24)	6 (1.76)	59 (17.35)	6 (1.76)
Fibrosis & Cirrhosis	473 (61.03)	162 (20.90)	16 (2.06)	21 (2.71)	89 (11.48)	14 (1.81)
Hepatic failure	199 (64.40)	60 (19.42)	7 (2.27)	4 (1.29)	35 (11.33)	4 (1.29)
**Cancer**						
Liver cancer	232 (55.77)	114 (27.40)	10 (2.40)	8 (1.92)	49 (11.78)	3 (0.72)
**Musculoskeletal**						
Joint replacement surgery (any)	12355 (59.84)	4771 (23.11)	454 (2.20)	500 (2.42)	2415 (11.70)	150 (0.73)
Osteoarthritis	4343 (59.08)	1681 (22.87)	177 (2.41)	189 (2.57)	893 (12.15)	68 (0.93)
Fractures (any)	10665 (59.31)	4204 (23.38)	375 (2.09)	430 (2.39)	2188 (12.17)	119 (0.66)
Fragility fractures	6308 (59.29)	2517 (23.66)	214 (2.01)	275 (2.58)	1264 (11.88)	61 (0.57)
Osteoporosis	6990 (60.50)	2580 (22.33)	254 (2.20)	244 (2.11)	1389 (12.02)	97 (0.84)
Rheumatoid arthritis	2188 (61.05)	818 (22.82)	79 (2.20)	86 (2.40)	390 (10.88)	23 (0.64)
**Brain**						
Any brain outcome (dementia, delirium, or Parkinson's disease)	3875 (59.16)	1547 (23.62)	142 (2.17)	154 (2.35)	777 (11.86)	55 (0.84)
Dementia	2159 (59.72)	837 (23.15)	68 (1.88)	83 (2.30)	440 (12.17)	28(0.77)
Alzheimer's disease	1072 (58.20)	450 (24.43)	37 (2.01)	50 (2.71)	220 (11.94)	13 (0.71)
Non-Alzheimer's dementia	1099 (61.02)	395 (21.93)	31 (1.72)	34(1.89)	227 (12.60)	15 (0.83)
Delirium	1982 (58.17)	823 (24.16)	81 (2.38)	82 (2.41)	406 (11.92)	33 (0.97)

For peer review only - http://bmjopen.bmj.com/site/about/guidelines.xhtml

Parkinson’s disease	691 (60.72)	249 (21.88)	26 (2.28)	30 (2.64)	134 (11.78)	8 (0.70)
**Pancreas**						
T2 diabetes	6538 (59.27)	2575 (23.35)	240 (2.18)	275 (2.49)	1322 (11.99)	80 (0.73)
**Infection**						
Covid-19	1809 (58.83)	719 (23.38)	72 (2.34)	72 (2.34)	383 (12.46)	20 (0.65)
Cholecystitis	1749 (59.61)	675 (23.01)	62 (2.11)	68 (2.32)	351 (11.96)	29 (0.99)
Pneumonia	6127 (59.06)	2406 (23.19)	242 (2.33)	254 (2.45)	1275 (12.29)	70 (0.67)
Sepsis	10024 (59.37)	3837 (22.72)	379 (2.24)	414 (2.45)	2103 (12.45)	128 (0.76)
LRTI	3875 (59.94)	1437 (22.23)	139 (2.15)	143 (2.21)	821 (12.70)	50 (0.77)
URTI	636 (57.87)	277 (25.20)	21 (1.91)	21 (1.91)	142 (12.92)	2 (0.18)
UTI	9137 (59.45)	3490 (22.71)	351 (2.28)	366 (2.38)	1905 (12.40)	119 (0.77)
SSTI	3900 (59.15)	1533 (23.25)	158 (2.40)	152 (2.31)	799 (12.12)	51 (0.77)
**Cardiovascular**						
Arrhythmia	1654 (59.75)	656 (23.70)	48 (1.73)	67 (2.42)	323 (11.67)	20 (0.72)
Cardiomyopathy	608 (59.90)	230 (22.66)	19 (1.87)	25 (2.46)	130 (12.81)	3 (0.30)
CHD	7401 (59.49)	2875 (23.11)	294 (2.36)	291 (2.34)	1502 (12.07)	77 (0.62)
Heart failure	3550 (58.50)	1430 (23.57)	127 (2.09)	154 (2.54)	753 (12.41)	54 (0.89)
**Mental Health**						
Depression	6414 (59.58)	2430 (22.57)	269 (2.50)	278 (2.58)	1317 (12.23)	57 (0.53)

Incident disease numbers exclude prevalent disease at baseline. Numbers presented are n (%). Abbreviations: CHD, coronary heart disease; T1, type 1; T2, type 2; LRTI, lower respiratory tract infection; URTI, upper respiratory tract infection; UTI, urinary tract infection; SSTI, skin and soft tissue infection. Joint replacement surgery variable includes a diagnosis of hip, knee, ankle, or shoulder replacement. Any brain outcome variable includes a diagnosis of dementia, delirium, or Parkinson’s disease.

For peer review only - http://bmjopen.bmj.com/site/about/guidelines.xhtml

eTable 12. Hazard ratios of incident disease outcomes in p.C282Y/H63D genotypes in females, excluding a diagnosis of hemochromatosis at baseline

Females	No mutations (Ref group)	H63D +/-		H63D +/+		C282Y +/ H36D +		C282Y +/-		C282Y +/+	
		HR (95% CI)	P	HR (95% CI)	P	HR (95% CI)	P	HR (95% CI)	P	HR (95% CI)	P
Hemochromatosis	1	2.04 (1.31-3.17)	**1.70E-03**	7.10 (3.75-13.45)	**1.80E-09**	32.79 (22.10-48.64)	**2.10E-67**	4.15 (2.68-6.44)	**1.80E-10**	675.18 (489.89-930.55)	**0.00E+00**
All-cause mortality	1	0.99 (0.96-1.03)	0.69	0.95 (0.85-1.05)	0.31	1.00 (0.90-1.11)	0.98	1.01 (0.97-1.06)	0.61	1.13 (0.95-1.35)	0.16
**Liver**											
Liver disease (any)	1	1.01 (0.95-1.07)	0.73	1.00 (0.84-1.18)	0.97	1.18 (1.01-1.37)	**0.03**	1.08 (1.00-1.16)	0.06	1.58 (1.23-2.02)	**2.80E-04**
Alcoholic liver disease	1	1.10 (0.84-1.43)	0.48	1.57 (0.85-2.88)	0.15	0.79 (0.35-1.78)	0.57	1.54 (1.14-2.06)	**0.43**	2.75 (1.21-6.20)	**0.02**
Fibrosis & Cirrhosis	1	0.87 (0.73-1.04)	0.13	0.87 (0.53-1.44)	0.60	1.11 (0.72-1.72)	0.65	0.93 (0.74-1.16)	0.51	2.67 (1.57-4.55)	**3.00E-04**
Hepatic failure	1	0.77 (0.58-1.03)	0.08	0.92 (0.43-1.95)	0.83	0.50 (0.18-1.34)	0.17	0.85 (0.59-1.22)	0.38	1.76 (0.65-4.74)	0.26
**Cancer**											
Liver cancer	1	1.26 (1.10-1.58)	**0.04**	1.13 (0.60-2.13)	0.71	0.89 (0.44-1.81)	0.75	1.06 (0.78-1.45)	0.69	1.22 (0.39-3.81)	0.73
**Musculoskeletal**											
Joint replacement surgery (any)	1	0.99 (0.96-1.02)	0.54	0.97 (0.88-1.06)	0.47	1.04 (0.95-1.14)	0.35	0.99 (0.94-1.03)	0.51	1.16 (0.99-1.37)	**0.07**
Osteoarthritis	1	0.99 (0.94-1.05)	0.72	1.06 (0.91-1.23)	0.46	1.09 (0.95-1.27)	0.22	1.02 (0.95-1.10)	0.59	1.46 (1.15-1.85)	**2.10E-03**
Fractures (any)	1	1.01 (0.97-1.05)	0.65	0.91 (0.82-1.01)	0.07	1.02 (0.92-1.12)	0.76	1.02 (0.97-1.07)	0.42	1.01 (0.84-1.20)	0.95
Fragility fractures	1	1.02 (0.98-1.07)	0.37	0.87 (0.76-1.00)	**0.05**	1.10 (0.98-1.24)	0.12	0.99 (0.93-1.05)	0.73	0.86 (0.67-1.10)	0.23
Osteoporosis	1	0.94 (0.90-0.99)	**0.01**	0.94 (0.83-1.07)	0.34	0.89 (0.78-1.01)	0.08	0.99 (0.93-1.05)	0.73	1.26 (1.03-1.54)	**0.02**
Rheumatoid arthritis	1	0.96 (0.88-1.04)	0.28	0.94 (0.75-1.17)	0.58	1.00 (0.81-1.25)	0.97	0.89 (0.80-0.99)	**0.03**	0.96 (0.63-1.44)	0.83
**Brain**											
Any brain outcome (dementia, delirium, or Parkinson's disease)	1	1.02 (0.97-1.09)	0.43	0.93 (0.79-1.11)	0.43	1.03 (0.87-1.20)	0.76	1.00 (0.92-1.08)	0.91	1.23 (0.94-1.61)	0.13
Dementia	1	1.00 (0.92-1.08)	0.94	0.80 (0.63-1.02)	0.07	0.99 (0.80-1.24)	0.95	1.01 (0.91- 1.12)	0.86	1.10 (0.76-1.60)	0.61
Alzheimer's disease	1	1.08 (0.97-1.20)	0.18	0.86 (0.63-1.21)	0.40	1.20 (0.90-1.59)	0.21	1.02 (0.88-1.17)	0.84	1.02 (0.59-1.76)	0.95

For peer review only - http://bmjopen.bmj.com/site/about/guidelines.xhtml

Non-Alzheimer's dementia	1	0.93 (0.83-1.04)	0.20	0.72 (0.50-1.03)	0.07	0.81 (0.57-1.14)	0.22	1.03 (0.89-1.18)	0.73	1.17 (0.71-1.96)	0.54
Delirium	1	1.07 (0.98-1.16)	0.13	1.05 (0.84-1.31)	0.67	1.06 (0.85-1.33)	0.58	1.01 (0.91-1.13)	0.79	1.44 (1.02-2.03)	**0.04**
Parkinson's disease	1	0.93 (0.80-1.07)	0.29	0.97 (0.66-1.44)	0.89	1.15 (0.79-1.65)	0.47	0.98 (0.81-1.18)	0.84	1.04 (0.52-2.10)	0.91
**Pancreas**											
T1 or T2 diabetes	1	1.01 (0.97-1.06)	0.54	0.98 (0.86-1.11)	0.72	1.08 (0.96-1.22)	0.20	1.02 (0.96-1.08)	0.59	1.13 (0.90-1.41)	0.28
**Infection**											
COVID-19	1	1.02 (0.93-1.11)	0.68	1.03 (0.81-1.30)	0.83	1.02 (0.80-1.29)	0.88	1.06 (0.94-1.18)	0.34	1.00 (0.64-1.56)	1.00
Cholecystitis	1	0.98 (0.90-1.08)	0.73	0.92 (0.71-1.19)	0.52	0.98 (0.77-1.24)	0.84	0.99 (0.88-1.11)	0.86	1.49 (1.03-2.16)	**0.03**
Pneumonia	1	1.00 (0.96-1.05)	0.84	1.02 (0.90-1.17)	0.71	1.05 (0.93-1.20)	0.41	1.03 (0.97-1.10)	0.29	1.02 (0.81-1.29)	0.85
Sepsis	1	0.98 (0.94-1.01)	0.22	0.97 (0.88-1.08)	0.62	1.05 (0.95-1.16)	0.34	1.04 (0.99-1.09)	0.11	1.14 (0.96-1.36)	0.14
LRTI	1	0.95 (0.89-1.01)	0.07	0.92 (0.78-1.09)	0.34	0.93 (0.78-1.09)	0.36	1.04 (0.97-1.12)	0.29	1.14 (0.86-1.51)	0.35
URTI	1	1.12 (0.97-1.29)	0.13	0.86 (0.56-1.33)	0.51	0.84 (0.54-1.30)	0.43	1.12 (0.93-1.34)	0.24	0.29 (0.07-1.17)	0.08
UTI	1	0.97 (0.94-1.01)	0.17	0.99 (0.89-1.10)	0.82	1.02 (0.92-1.13)	0.76	1.03 (0.98-1.08)	0.22	1.17 (0.97-1.40)	0.10
SSTI	1	1.01 (0.95-1.07)	0.81	1.05 (0.90-1.23)	0.54	1.00 (0.85-1.17)	0.98	1.03 (0.95-1.11)	0.52	1.20 (0.91-1.59)	0.19
**Cardiovascular**											
Arrhythmia	1	1.02 (0.93-1.12)	0.69	0.76 (0.57-1.01)	0.06	1.04 (0.82-1.33)	0.73	0.98 (0.87-1.11)	0.78	1.13 (0.72-1.75)	0.60
Cardiomyopathy	1	0.97 (0.83-1.13)	0.71	0.82 (0.52-1.30)	0.41	1.07 (0.72-1.59)	0.75	1.08 (0.90-1.31)	0.40	0.46 (0.15-1.43)	0.18
CHD	1	0.99 (0.95-1.04)	0.81	1.03 (0.92-1.16)	0.60	1.01 (0.89-1.13)	0.92	1.01 (0.96-1.07)	0.70	0.92 (0.74-1.15)	0.48
Heart failure	1	1.03 (0.97-1.10)	0.28	0.92 (0.77-1.10)	0.36	1.12 (0.95-1.32)	0.17	1.06 (0.98-1.15)	0.15	1.35 (1.03-1.77)	**0.03**
**Mental Health**											
Depression	1	0.97 (0.93-1.02)	0.20	1.10 (0.97-1.23)	0.14	1.11 (0.98-1.25)	0.09	1.03 (0.97-1.09)	0.37	0.83 (0.64-1.08)	0.16

HR (Hazard ratio) compared to those with neither *HFE* mutation. Cox proportional hazards regression models adjusted for age, assessment centre, and genetic principal components 1–10. Abbreviations: CHD, coronary heart disease; T1, type 1; T2, type 2; LRTI, lower respiratory tract infection; URTI, upper respiratory tract infection; UTI, urinary tract infection SSTI, skin and soft tissue infection; CI, confidence interval. Joint replacement surgery variable includes a diagnosis of hip, knee, ankle, or shoulder replacement. Any brain outcome variable includes a diagnosis of dementia, delirium, or Parkinson's disease

For peer review only

For peer review only - http://bmjopen.bmj.com/site/about/guidelines.xhtml

eFigure 1. Kaplan-Meier curves for the cumulative incidence of (a) mortality, (b) liver disease, (c) any joint replacement, and (d) any brain outcome, in male *HFE* p.C282Y homozygotes compared to those with no mutations, excluding a diagnosis of hemochromatosis at baseline

a)

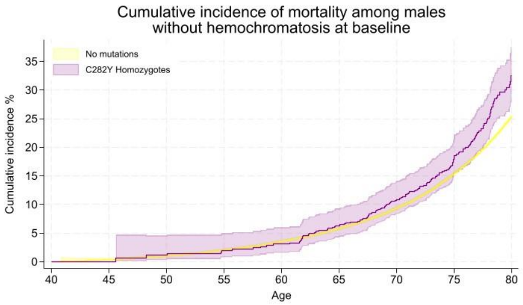

b)

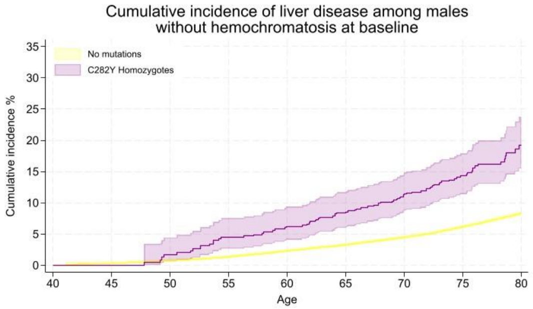

c)

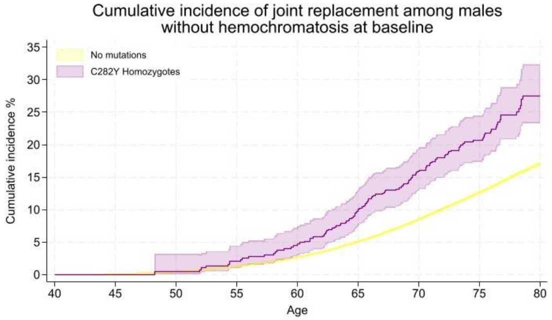

d)

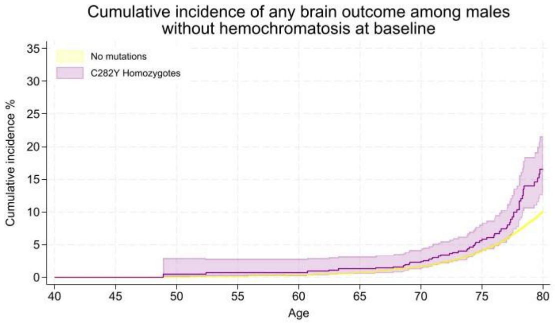

Cumulative incidence (estimated % diagnosed by age 80 years; 95% CIs). Joint replacement surgery includes a diagnosis of hip, knee, ankle, or shoulder replacement. Any brain outcome included a diagnosis of delirium, dementia, or Parkinson's disease.

For peer review only - http://bmjopen.bmj.com/site/about/guidelines.xhtml

eFigure 2. Kaplan-Meier curves for the cumulative incidence of (a) liver disease, and (b) any joint replacement, in female *HFE* p.C282Y homozygotes compared to those with no mutations, excluding a diagnosis of hemochromatosis at baseline

a) b)

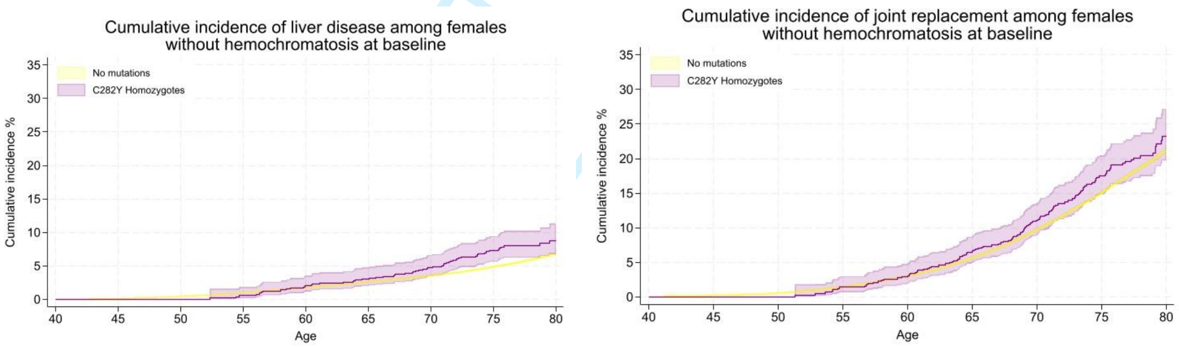

Cumulative incidence (estimated % diagnosed by age 80 years; 95% CIs). Joint replacement surgery includes a diagnosis of hip, knee, ankle, or shoulder replacement.

STROBE 2007 (v4) Statement—Checklist of items that should be included in reports of *cohort studies*

Section/Topic	Item #	Recommendation	Reported on page #
**Title and abstract**	1	(*a*) Indicate the study's design with a commonly used term in the title or the abstract	1-2
		(*b*) Provide in the abstract an informative and balanced summary of what was done and what was found	2
**Introduction**			
Background/rationale	2	Explain the scientific background and rationale for the investigation being reported	4-5
Objectives	3	State specific objectives, including any prespecified hypotheses	5
**Methods**			
Study design	4	Present key elements of study design early in the paper	5
Setting	5	Describe the setting, locations, and relevant dates, including periods of recruitment, exposure, follow-up, and data collection	5-6
Participants	6	(*a*) Give the eligibility criteria, and the sources and methods of selection of participants. Describe methods of follow-up	5-6
		(*b*) For matched studies, give matching criteria and number of exposed and unexposed	N/A
Variables	7	Clearly define all outcomes, exposures, predictors, potential confounders, and effect modifiers. Give diagnostic criteria, if applicable	5-6
Data sources/ measurement	8*	For each variable of interest, give sources of data and details of methods of assessment (measurement). Describe comparability of assessment methods if there is more than one group	*5-6*
Bias	9	Describe any efforts to address potential sources of bias	5
Study size	10	Explain how the study size was arrived at	5
Quantitative variables	11	Explain how quantitative variables were handled in the analyses. If applicable, describe which groupings were chosen and why	5-6
Statistical methods	12	(*a*) Describe all statistical methods, including those used to control for confounding	6
		(*b*) Describe any methods used to examine subgroups and interactions	6
		(*c*) Explain how missing data were addressed	N/A
		(*d*) If applicable, explain how loss to follow-up was addressed	N/A
		(*e*) Describe any sensitivity analyses	6
**Results**			

For peer review only

Participants	13*	(a) Report numbers of individuals at each stage of study—eg numbers potentially eligible, examined for eligibility, confirmed eligible, included in the study, completing follow-up, and analysed	6-7
		(b) Give reasons for non-participation at each stage	N/A
		(c) Consider use of a flow diagram	N/A
Descriptive data	14*	(a) Give characteristics of study participants (eg demographic, clinical, social) and information on exposures and potential confounders	6-7, 18 *Supplement Material* pages: 3-4
		(b) Indicate number of participants with missing data for each variable of interest	N/A
		(c) Summarise follow-up time (eg, average and total amount)	6
Outcome data	15*	Report numbers of outcome events or summary measures over time	*Supplement Material,* pages 5-6, 9-10, 14-15, 18-19
Main results	16	(*a*) Give unadjusted estimates and, if applicable, confounder-adjusted estimates and their precision (eg, 95% confidence interval). Make clear which confounders were adjusted for and why they were included	6-9 *Supplement Material,* pages: 7-8, 11-12, 13, 16-17, 20-21
		(*b*) Report category boundaries when continuous variables were categorized	N/A
		(*c*) If relevant, consider translating estimates of relative risk into absolute risk for a meaningful time period	N/A
Other analyses	17	Report other analyses done—eg analyses of subgroups and interactions, and sensitivity analyses	7
**Discussion**			
Key results	18	Summarise key results with reference to study objectives	10
**Limitations**			10-12
Interpretation	20	Give a cautious overall interpretation of results considering objectives, limitations, multiplicity of analyses, results from similar studies, and other relevant evidence	10-12
Generalisability	21	Discuss the generalisability (external validity) of the study results	10-12
**Other information**			
Funding	22	Give the source of funding and the role of the funders for the present study and, if applicable, for the original study on which the present article is based	13

*Give information separately for cases and controls in case-control studies and, if applicable, for exposed and unexposed groups in cohort and cross-sectional studies.

For peer review only

Note: An Explanation and Elaboration article discusses each checklist item and gives methodological background and published examples of transparent reporting. The STROBE checklist is best used in conjunction with this article (freely available on the Web sites of PLoS Medicine at http://www.plosmedicine.org/, Annals of Internal Medicine at http://www.annals.org/, and Epidemiology at http://www.epidem.com/). Information on the STROBE Initiative is available at www.strobe-statement.org.

For peer review only
